# Novel Sulfonamide Derivatives Containing a Piperidine Moiety as New Bactericide Leads for Managing Plant Bacterial Diseases

**DOI:** 10.3390/ijms24065861

**Published:** 2023-03-20

**Authors:** Jiao Xie, Zhou-Qing Long, Ai-Qun Chen, Ying-Guo Ding, Shi-Tao Liu, Xiang Zhou, Li-Wei Liu, Song Yang

**Affiliations:** National Key Laboratory of Green Pesticide, Key Laboratory of Green Pesticide and Agricultural Bioengineering, Ministry of Education, Center for R&D of Fine Chemicals of Guizhou University, Guiyang 550025, China

**Keywords:** antibacterial activity, dihydropteroate synthase, cell membrane, plant bacterial disease

## Abstract

Plant bacterial diseases are an intractable problem due to the fact that phytopathogens have acquired strong resistances for traditional pesticides, resulting in restricting the quality and yield of agricultural products around the world. To develop new agrochemical alternatives, we prepared a novel series of sulfanilamide derivatives containing piperidine fragments and assessed their antibacterial potency. The bioassay results revealed that most molecules displayed excellent in vitro antibacterial potency towards *Xanthomonas oryzae* pv. *oryzae* (*Xoo*) and *Xanthomonas axonopodis* pv. *citri* (*Xac*). In particular, molecule **C_4_** exhibited outstanding inhibitory activity toward *Xoo* with EC_50_ value of 2.02 µg mL^−1^, which was significantly better than those of the commercial agents bismerthiazol (EC_50_ = 42.38 µg mL^−1^) and thiodiazole copper (EC_50_ = 64.50 µg mL^−1^). A series of biochemical assays confirmed that compound **C_4_** interacted with dihydropteroate synthase, and irreversibly damaged the cell membrane. In vivo assays showed that the molecule **C_4_** presented acceptable curative and protection activities of 34.78% and 39.83%, respectively, at 200 µg mL^−1^, which were greater than those of thiodiazole and bismerthiazol. This study highlights the valuable insights for the excavation and development of new bactericides that can concurrently target dihydropteroate synthase and bacterial cell membranes.

## 1. Introduction

The world population is increasing and should reach about 9.2 billion by 2050. Meeting the growing population’s demand for food will require increasing food crops’ production levels. In particular, since access to additional agricultural land is very limited, it is particularly important to produce food crops with higher quality and yield on the same land area [1]. Unfortunately, the presence of plant pathogens has severely disrupted the growth of food crops around the globe. For example, *Xanthomonas*, a large genus of Gram-negative bacteria, contains the major pathogenic bacteria for plants. The genus comprises 27 species with various virulence factors and pathogenicity for nearly 400 different plant hosts, including rice, citrus, tomatoes, cabbage, bananas, peppers, and wheat [2,3,4]. Currently, bacterial leaf blight caused by refractory *Xanthomonas oryzae* pv. *oryzae* (*Xoo*) is the most devastating bacterial disease for rice and one of the main obstacles to rice production. *Xoo* enters wounds in the leaves or roots, broken trichomes, or hydathodes and invades the xylem vessels. Rice leaves then wither and etiolate, which hinders photosynthesis, resulting in yield reduction and economic losses of of 10–50% [5,6,7,8]. Chemical antibacterial agents have been widely used since the 1960s and remain a major pathogen control method in current agriculture. However, the irregular use of traditional chemical antibacterial agents including bismerthiazol (BT) and thiodiazole copper (TC) increases the resistance of plant pathogens, reducing the control efficiency of these agents, which are also toxic for humans and the environment [9,10]. Therefore, it is urgent to develop new, low-risk, green agrochemicals with high efficiency and high selectivity to manage plant diseases, thereby improving production levels and meeting the population’s needs.

Dihydropteroate synthase (DHPS) is a critical enzyme of the de novo folate biosynthesis pathway. This enzyme catalyzes the condensation of 6-hydroxymethyl-7,8-dihydropterin-pyrophosphate (DHPPP) with *p*-aminobenzoic acid to form 7,8-dihydropteroate (DHF). DHF is then converted into tetrahydrofolate, which is involved in the process of DNA synthesis and the biosynthesis of several amino acids. Therefore, inhibiting DHPS interrupts folate biosynthesis, thereby inhibiting DNA replication, significantly hindering normal bacterial growth and proliferation [11,12,13,14]. Sulfonamides are one of the earliest discovered synthetic antibiotics; their chemical structures are similar to those of *p*-aminobenzoic acid and thus they competitively bind to the active site of DHPS. Sulfonamide drugs have been widely used in biological systems to cure bacterial infections since the initial discovery of the sulfonamide class of antibiotics [15]. Moreover, sulfonamide compounds have a broad range of applications in medicine due to their wide spectrum of bioactivities, which include antibacterial [16], antifungal [17], antiviral [18], anticancer [18], anti-inflammatory [19], anti-tumoral [19] and antimalarial [20] effects. Interestingly, some sulfonamide derivatives can effectively kill these pathogens by damaging cell membrane integrity [21,22,23]. Given these factors, low-cost sulfonamides with rational molecular modifications might be reconsidered for treating plant pathogenic bacteria.

Furthermore, the piperidine link is a very important active fragment in drugs, pesticides, and natural products. It is the most prevalent nitrogen ring system in nitrogen-containing heterocyclic drugs approved by the U.S. FDA [24]. Many commercialized agents contain a piperidine link, such as the antifungal fenpropidin, the anti-*oomycete* agent oxathiapiprolin, the anti-Alzheimer drug donepezil, and the anticonvulsant agent ifenprodil [25]. In addition, some piperidine derivatives containing with sulfonamide unit displayed outstanding bioactivity [26,27]. On the other hand, saturated medium-length alkyl chains have high affinities for various types of cellular membranes thanks to their high lipophilicity [28,29,30]. A study on anacardic acids, saturated fatty acids, and alkyl gallic esters possessing different side-chain lengths showed that alkyl chain lengths between C-8 and C-12 provided optimal antibacterial activities, and the optimal alkyl tail length depended on the species of pathogen and the parent structure of the compound [31,32,33,34].

**Figure 1:**
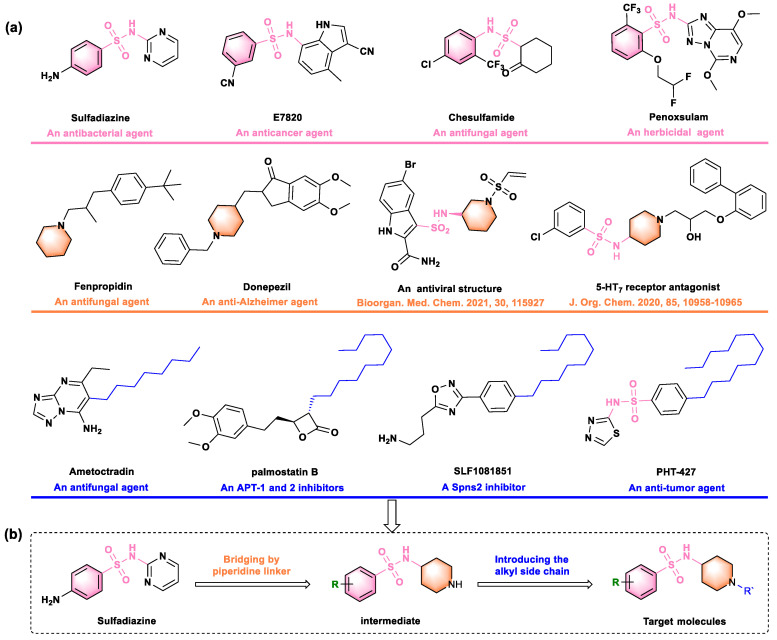
(**a**) Some commercial and reported bioactive compounds containing the sulfonamide, piperidine, or alkyl side chain unit. (**b**) The design strategy for title molecules [26,27].

Based on these considerations and our foregoing experience on pesticide development, we prepared a series of new sulfonamide compounds linked with an alkyl tail through a piperidine moiety based on a molecular hybridization strategy (Figure 1). We then evaluated the antibacterial activities of all the designed molecules towards the intractable pathogenic strains *Xoo* and *Xac* in vitro using the classical turbidimetric method and growth curves assay. In addition, we assessed the in vivo control effect on rice bacterial blight of the most active compound **C_4_**. Finally, we performed scanning electron microscope (SEM) imaging, membrane permeabilization assays, and a molecular docking study with DHPS to preliminarily explore the possible antibacterial mechanisms of these designed structures.

## 2. Results and Discussion

### 2.1. Synthesis of the Target Molecules

The compounds **A_1_**–**A_24_** and **B_1_**–**B_2_** were prepared via the reactions of substitution, deprotection, and substitution, with the yield of reaction from 24.5% to 72.8%. The reactive acyl chloride, sulfonyl chloride, may be reacted with water to form the by-products carboxylic acid and benzenesulfonic acid during the reaction. Moreover, the by-products *N*, *N*-disubstituted amides, and sulfonamides may also be formed. The compounds **B_3_**–**B_6_** were prepared via the reaction of substitution, deprotection, and condensation, with the yield of reaction from 31.3% to 67.7%. EDCI and HOBt were used as catalysts for the condensation reaction. Analogously, the compounds **C_1_**–**C_6_** were prepared via the reaction of substitution, deprotection, and substitution, with the yield of reaction from 45.6% to 74.4%. Haloalkanes and intermediate **4** undergo the Hofmann alkylation reaction to produce ammonium impurity. The state, yield, melting point and spectra data of all target compounds were summarized in supporting information. 

### 2.2. In Vitro Antibacterial Activities and Structure−Activity Relationship (SAR) Analysis

**Scheme 1:**
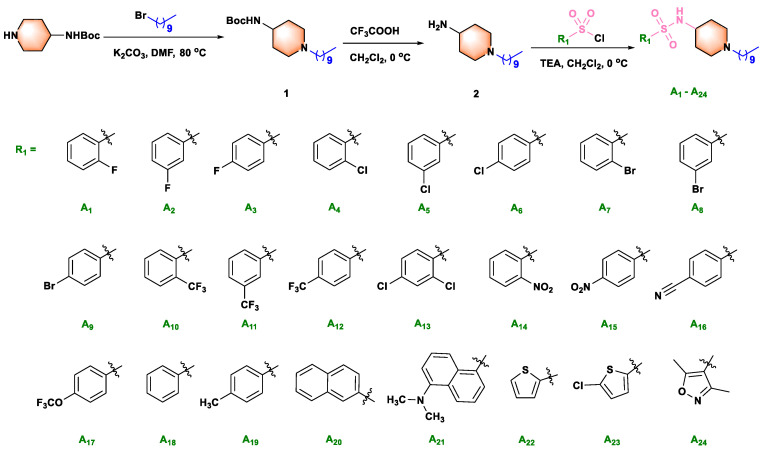
Synthetic route for title molecules **A_1_**–**A_24_**.

First, as displayed in Scheme 1, a series of sulfonamide derivatives (**A_1_**–**A_24_**) was prepared and their bioactivity was evaluated using a classical turbidimetric method [35]. Commercialized BT, TC, and sulfadiazine served as the positive controls. The bioassay results revealed that all target compounds displayed fantastic antibacterial potency against the plant pathogens *Xoo* and *Xac*, with EC_50_ values ranging from 2.65 to 11.83 µg mL^−1^ (against *Xoo*) and from 4.74 to 21.26 µg mL^−1^ (against *Xac*) (Table 1). These values were strikingly superior to those of the commercial agents BT (EC_50_ = 42.38 and 110.54 µg mL^−1^), TC (EC_50_ = 64.50 and 121.40 µg mL^−1^), and sulfadiazine (>150 µg mL^−1^). These results demonstrated that the design of sulfonamide derivatives containing medium alkyl chains could accelerate the discovery of highly effective antibacterial agents for application in agriculture.

Based on the above EC_50_ values, we deduced the following structure−activity relationships (SAR): (1) The position of the halogen on the benzene ring dramatically affected the antibacterial potency; having the substituent on the *meta*-position yielded vastly superior antibacterial activity to having it on the *para-* or *ortho*-position. This was demonstrated in the following compound activity rankings: 3-F (**A_2_**, 4.20 µg mL^−1^) > 2-F (**A_1_**, 4.79 µg mL^−1^) and 4-F (**A_3_**, 4.69 µg mL^−1^) against *Xoo*, and 3-F (**A_2_**, 5.34 µg mL^−1^) > 2-F (**A_1_**, 7.76 µg mL^−1^) and 4-F (**A_3_**, 7.90 µg mL^−1^) against *Xac*. We observed similar antibacterial activity patterns for Cl and Br atom substitutions against *Xoo* and *Xac*. (2) Introducing a disubstituted halogen to the benzene ring reduced the biological activity. Compound **A_13_** (2,4-diCl, 6.84 µg mL^−1^ toward *Xoo*) was a little less potent than compounds (**A_4_**, 2-Cl, 6.10 µg mL^−1^; **A_5_**, 3-Cl, 3.18 µg mL^−1^; and **A_6_**, 4-Cl, 3.80 µg mL^−1^ toward *Xoo*). Additionally, we observed a similar tendency against *Xac*. (3) The electron-withdrawing and electron-donating of the para-position on the benzene rings differently affected the activities toward the two strains, except for -OCF_3_. Introducing an electron-donating group at the *para*-position was more favorable in enhancing the anti-*Xoo* activity than an electron-withdrawing group. This is seen in **A_19_** (4-CH_3_, 3.62 µg mL^−1^) > **A_3_** (4-F, 4.69 µg mL^−1^), **A_6_** (4-Cl, 3.80 µg mL^−1^), **A_9_** (4-Br, 5.16 µg mL^−1^), **A_12_** (4-CF_3_, 5.95 µg mL^−1^), **A_15_** (4-NO_2_, 4.73 µg mL^−1^), and **A_16_** (4-CN, 4.98 µg mL^−1^). By contrast, introducing an electron-withdrawing group at the *para*-position had better anti-*Xac* activity than an electron-donating group: **A_3_** (4-F, 7.90 µg mL^−1^), **A_6_** (4-Cl, 7.49 µg mL^−1^), **A_9_** (4-Br, 7.04 µg mL^−1^), **A_12_** (4-CF_3_, 6.76 µg mL^−1^), **A_15_** (4-NO_2_, 7.29 µg mL^−1^), and **A_16_** (4-CN, 6.81 µg mL^−1^) > **A_19_** (4-CH_3_, 12.71 µg mL^−1^). (4) Replacing the benzene ring with a heterocycle dramatically decreased the antibacterial activity, with EC_50_ values augmenting from 2.65–6.84 to 10.98–11.83 µg mL^−1^ toward *Xoo* and from 4.74–14.73 to 15.27–21.26 µg mL^−1^ against *Xac*, showing that the benzene ring group was crucial to biological activity. Particularly, compounds **A_10_** (2-CF_3_, EC_50_ value = 2.65 µg mL^−1^ for *Xoo*) and **A_8_** (3-Br, EC_50_ value = 4.74 µg mL^−1^ for *Xac*) exhibited the highest antibacterial activity.

**Scheme 2:**
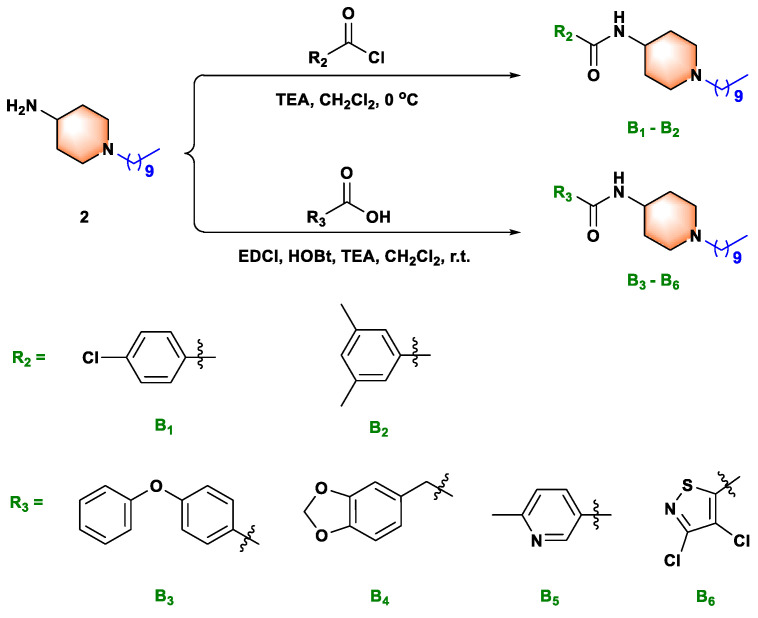
Synthesis route of title molecules **B_1_**−**B_6_**.

We next synthesized compounds **B_1_**–**B_6_** (see Scheme 2) to explore whether the sulfonamide bond was necessary for biological activity. As shown in Table 2, compared with compounds **A_1_**–**A_21_**, compounds **B_1_**–**B_6_** exhibited weak to moderate biological activities against anti-*Xoo* and anti-*Xac* in vitro. Therefore, neither the small substituted benzamide (**B_1_** and **B_2_**), the large substituted benzamide (**B_3_** and **B_4_**), nor the heterocyclic amide (**B_5_** and **B_6_**) improved the biological activity, confirming that the sulfonamide bond played an essential role in the antibacterial capacity. These outcomes provided a theoretical basis for the design of sulfonamide compounds as putative DHPS inhibitors.

**Scheme 3:**
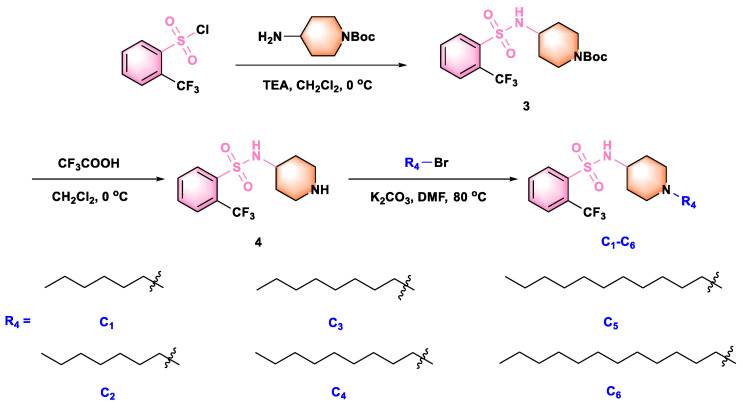
Synthesis route of title molecules **C_1_**−**C_6_**.

**Figure 2:**
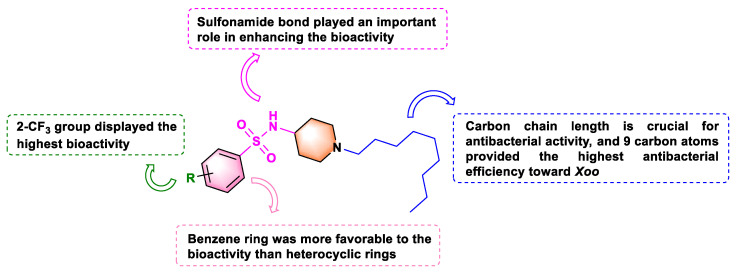
Diagram for structure−activity relationship (SAR) analysis.

The optimal alkyl side chain length depends on parent structures and the tested pathogen species [31]. To find the optimal chain length against *Xoo* and *Xac*, we synthesized compounds **C_1_**–**C_6_** (See Scheme 3) with different alkyl chain lengths based on the structure of the highly active compound **A_10_**. As Table 3 illustrated, increasing the chain length first increased the antibacterial activity and then decreased it. Interestingly, compound **C_4_** (with nine carbon atoms) displayed the highest antibacterial efficiency toward *Xoo*, with an EC_50_ value of 2.02 µg mL^−1^. However, compound **C_3_** (with eight carbon atoms) had the highest antibacterial potency toward *Xac*, with an EC_50_ value of 8.60 µg mL^−1^. This difference might be due to the different hydrophobic interactions between these compounds and the membrane lipids of the two bacteria [31]. Eventually, the preliminary structure–activity relationships of title compounds were summarized as Figure 2.

### 2.3. Effect of Molecule ***C_4_*** on the Growth of Xoo Cells

**Figure 3:**
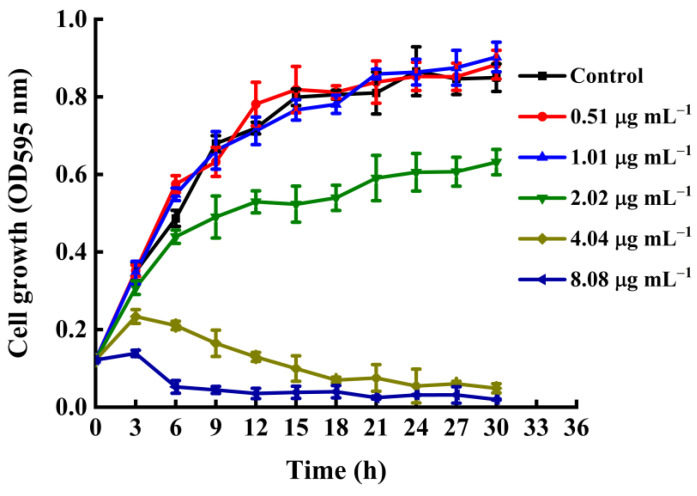
Growth curves of *Xoo* cells treated with compound **C_4_** at various dosages (0.51, 1.01, 2.02, 4.04, 8.08 µg mL^−1^) in nutrient broth medium.

Since compound **C_4_** exhibited the most potent activity against *Xoo*, we selected it to further study the antibacterial mechanism. First, we performed a growth curve assay to explore the bactericidal performance of compound **C_4_** against *Xoo*. In detail, we treated a *Xoo* suspension with compound **C_4_** at different concentrations (0.51, 1.01, 2.02, 4.04, and 8.08 µg mL^−1^) and recorded the absorbance of the culture at 595 nm every 3 h for 30 h. As performed in Figure 3, *Xoo* cells incubated with 0.51 or 1.01 µg mL^−1^ of **C_4_** grew normally. Meanwhile, at 2.02 µg mL^−1^, compound **C_4_** clearly inhibited cell growth after 9 h of incubation. In addition, at 4.04 and 8.08 µg mL^−1^, compound **C_4_** started inhibiting *Xoo* cell growth after 3 h of incubation. These results showed that compound **C_4_** had remarkable bactericidal potency, and it could decrease the probability of bacterial resistance development.

### 2.4. Molecular Docking Simulation of ***C_4_*** with XooDHPS

**Figure 4:**
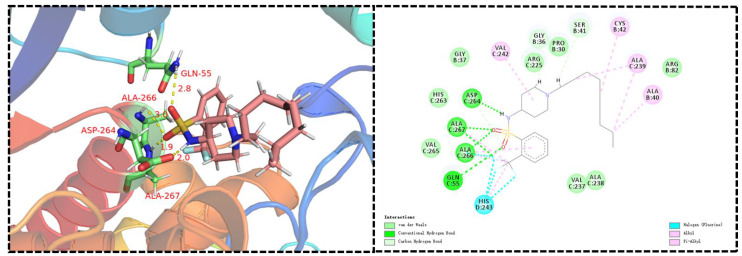
Predicted binding mode and interactions. Molecular docking of compound **C_4_** with DHPS. (PDB: 6DAY).

**Figure 5:**
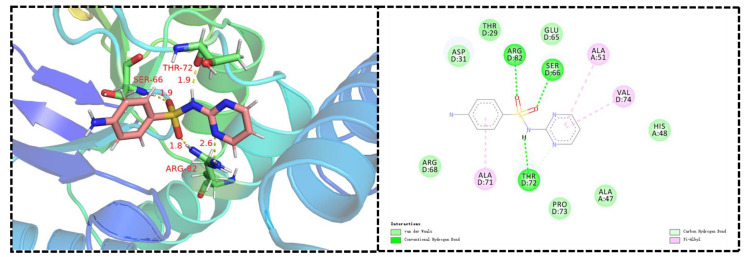
Predicted binding mode and interactions. Molecular docking of sulfadiazine with DHPS. (PDB: 6DAY).

DHPS is an indispensable enzyme in the biosynthetic machinery of folic acid, and the folate cyclic pathway is crucial for the biosynthesis of nucleotides in bacteria. Sulfanilamides are the first-discovered synthetic antifolic drugs. They bind to the active site of DHPS, thereby interrupting folate biosynthesis and impeding DNA replication; these are the main mechanisms of action of sulfonamide derivatives against bacteria [11,12,13,14]. To confirm whether our novel sulfonamide derivatives could also target DHPS, we produced molecular docking images showing the binding modes between compounds **C_4_** and *Xoo*DHPS (Figure 4), assessed the quality resulting of the structure model using the SAVES v5.0 server, and validated the 3D structures using the Ramachandran plot (Figure S1). Firstly, the molecular docking scores revealed that the highly active compound **C_4_** (EC_50_ = 2.02 µg mL^−1^) had a significantly higher binding score than the commercial sulfadiazine (EC_50_ > 150 µg mL^−1^) (total score 7.89 (compound **C_4_**) vs. 4.84 (sulfadiazine)). This result was in accordance with the EC_50_ values, suggesting that compound **C_4_** had a higher binding affinity for *Xoo*DHPS than sulfadiazine. The molecular docking revealed that the O atom of the sulfonamido fragment in compound **C_4_** could form a 2.8 Å hydrogen bond with the H atom of the amino group in the GLN-55 residue and 3.0 and 1.9 Å hydrogen bonds with the H atoms of the amino groups of ALA-266 and ALA-267. Additionally, the H atoms of the sulfonyl moiety and the O atom of carbonyl group in the ASP-264 residue could form a 2.0 Å hydrogen bond. These observations revealed that the sulfonamido group was a beneficial and vital structural fragment in compound **C_4_**. In addition, strong van der Waals, halogen (fluorine), carbon–hydrogen, pi-alkyl, and hydrophobic interactions could be observed between compound **C_4_** and various residues. All these interactions might stabilize the *Xoo*DHPS-**C_4_** complex. In contrast, although we observed four hydrogen bonds between sulfadiazine and *Xoo*DHPS (Figure 5), they displayed a low binding score, indicating a relatively weak interaction and a different binding mode.

### 2.5. SEM Morphological Study of Xoo Cells Treated with ***C_4_***

**Figure 6:**
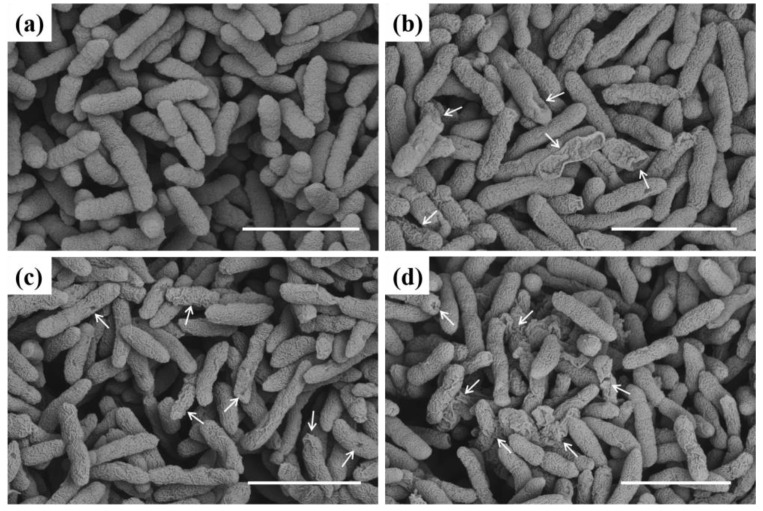
SEM images of *Xoo* cells incubated with compound **C_4_**. (**a**) Control, (**b**) 4.04 µg mL^−1^, (**c**) 8.08 µg mL^−1^, and (**d**) 16.16 µg mL^−1^. Scale bars: 2 µm. Arrow represents damaged *Xoo*.

To further probe the mechanism of action of the designed molecules on bacteria, we produced SEM images (Figure 6) showing the morphology of bacterial cells with various concentrations of compound **C_4_**. As indicated in Figure 6, untreated *Xoo* cells exhibited a smooth, intact, round surface and regular morphology. Meanwhile, most cells treated with 4.04 µg mL^−1^ of **C_4_** had lost their normal shape (Figure 6b) and displayed a partially sunken, massively wrinkled, highly shriveled surface and irregular morphology. Additionally, at 8.08 and 16.16 µg mL^−1^, this phenomenon was noticeably exacerbated (Figure 6c,d), potentially indicating protein, DNA, and RNA leakage. This outcome confirmed that this type of molecule showed strong interactions with plant pathogenic bacteria, affecting various physiological processes, possibly by altering and increasing the cell membranes’ permeability, thereby leading to bacteria death. The strong destructive effect of this compound on cell membrane integrity might be closely related to the hydrophobic nature of the alkyl tail.

### 2.6. Assessing the Cell Membrane Integrity by Fluorescence Microscopy

**Figure 7:**
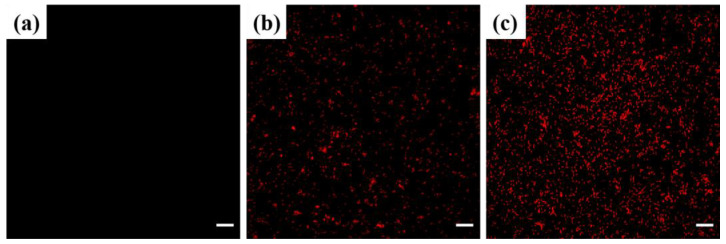
Fluorescence images of **C_4_**-treated *Xoo* cells stained with PI: (**a**) Control, (**b**) 4.04 µg mL^−1^, and (**c**) 16.16 µg mL^−1^. Scale bars: 10 µm.

The highly organized cell membrane structure of Gram-negative bacteria protects them from foreign antibacterial agents and toxic environmental factors. It is a vital barrier maintaining the stability of the intracellular environment and normal physiological processes [25,35,36]. Therefore, the discovery of antibacterial agents that disrupt cell membrane integrity is crucial for the management of bacterial diseases. Here, we assessed the membrane integrity of **C_4_**-treated *Xoo* cells by performing PI staining. PI is a non-fluorescent dye that does not cross the intact membrane of living cells but can enter dead cells or cells with a compromised membrane. It binds to single- and double-stranded DNA to form PI-DNA complexes emitting intense red fluorescence [37,38]. As shown in Figure 7 and Figure S2, we observed no red fluorescence in the negative controls (Figure 7a). In contrast, compound **C_4_** dose-dependently increased the red fluorescence intensity (Figure 7b,c), confirming that **C_4_** increased cell membrane permeability by damaging the bacterial membrane integrity, resulting in bacterial cell death.

### 2.7. Effect of ***C_4_*** on Electrical Conductivity

**Figure 8:**
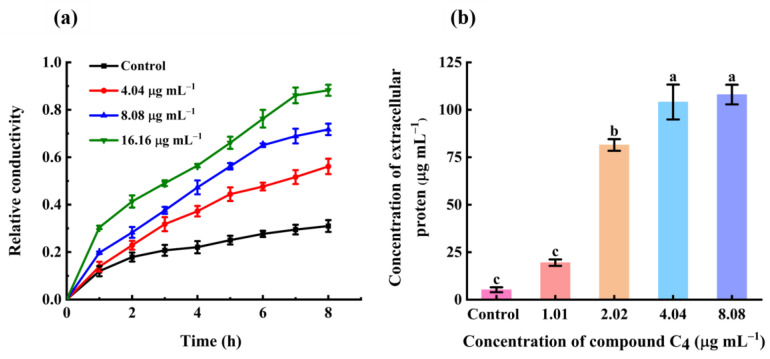
(**a**) Relative conductivity of *Xoo* cells treated with **C_4_** (Control, 4.04, 8.08, and 16.16 µg mL^−1^). (**b**) Extracellular protein leakage in *Xoo* cells treated with **C_4_** (Control, 1.01, 2.02, 4.04, and 8.08 µg mL^−1^). Bars indicate the mean ± SD. Lowercase letters indicate significant differences between the different treatment groups, as calculated using one-way ANOVA (*p* < 0.05).

The cytoplasmic membrane of bacteria provides a permeability barrier to small ions, including H^+^, Na^+^, and K^+^, which is necessary to maintain proper enzyme activity and maintain metabolism. Maintaining ion homeostasis is crucial to the energy status of the cell [39,40]. To further ascertain the membrane damage capacity of compound **C_4_**, we assessed electrolyte leakage by recording the electrical conductivity of cells using a DDS-307 conductivity meter (Figure 8a). The relative electrical conductivity of control cells slightly increased over time, which might be due to regular bacteria cytolysis and death [39]. Meanwhile, compound **C_4_** dose-dependently increased the relative electric conductivity of bacterial suspensions over time. At the end of the incubation (8 h), the relative conductivity reached 56% at 4.04 µg mL^−1^, 72% at 8.08 µg mL^−1^, and 88% at 16.16 µg mL^−1^. This outcome clearly disclosed that the compound **C_4_**-induced cell membrane damage caused massive bacterial intracellular electrolyte leakage. The loss of electrolytes impaired the balance of physiological processes, eventually causing the death of *Xoo* cells.

### 2.8. Effect of ***C_4_*** on Protein Concentrations

Physical damage to the bacterial lipid bilayer results in cytoplasmic content spillage [40]. We measured protein leakage in *Xoo* using a standard Bradford assay to confirm the irreversible **C_4_**-induced damage to the *Xoo* cell membrane [41]. The extracellular protein concentrations appear in Figure 8b. Compound **C_4_** dose-dependently increased extracellular protein content (Figure 8b). Accordingly, compound **C_4_** decreased the intracellular protein content of *Xoo* cells in a concentration-dependent manner (See Figure S3), confirming that molecule **C_4_** could damage the cell membrane and disrupt membrane integrity. This outcome was in accordance with the PI staining, SEM, and relative electric conductivity assays, suggesting that compound **C_4_** irreversibly damaged the *Xoo* cell membrane and that, as well as small ions, biological macromolecular proteins also leaked from bacterial cells.

### 2.9. Phytotoxicity Assay and In Vivo Bioassay against Rice Bacterial Leaf Blight

**Figure 9:**
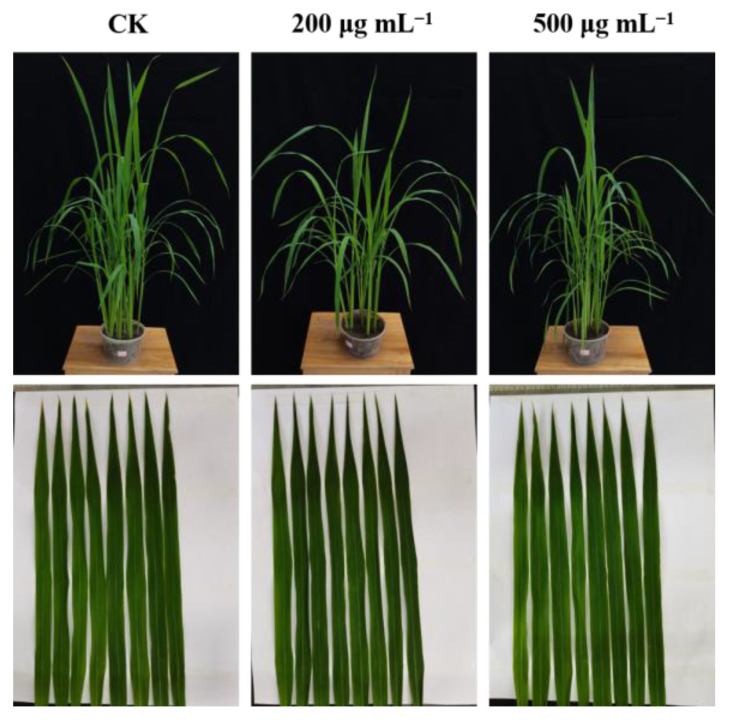
The phytotoxicity of **C_4_** at 200 and 500 µg mL^−1^ on rice plants in a controllable greenhouse.

**Figure 10:**
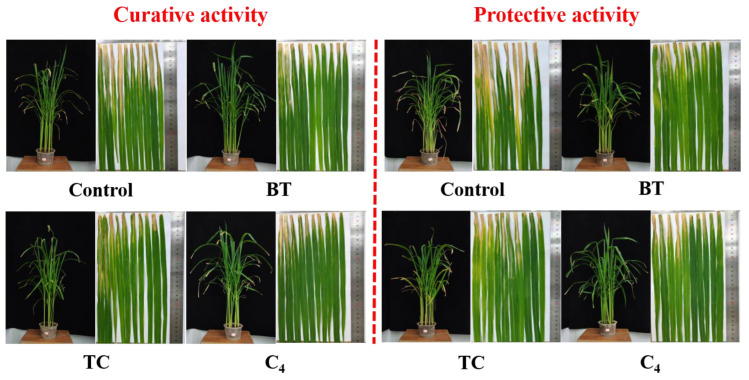
In vivo antibacterial activity of compound **C_4_** (200 µg mL^−1^) toward rice bacterial blight in a controllable greenhouse. BT and TC were the positive controls.

Next, to estimate the prospective application of the synthesized compounds as agricultural agents in the control of plant bacterial diseases, we assessed the phytotoxic activity and in vivo effect of compound **C_4_** on rice leaf blight. Commercial agents TC and BT were used as positive controls, while DMSO was used as the negative control, and the outcomes are shown in Figure 9 and Figure 10 and Table 4. First, the phytotoxicity assay revealed that **C_4_**-treated rice leaves appeared healthy and compound **C_4_** exhibited low phytotoxicity at 200 and 500 µg mL^−1^ (Figure 9). In addition, the in vivo assay revealed that **C_4_** showed acceptable activity toward rice bacterial leaf blight with the curative and protection control efficiencies of 34.78%, and 39.83%, respectively, at 200 µg mL^−1^, which were better than those of TC (29.57% and 30.51%) and comparable to those of BT (33.91% and 38.14%) (Figure 10 and Table 4). This finding indicates that compound **C_4_** is a promising lead for the control of intractable plant bacterial diseases, which should be further explored and developed.

## 3. Materials and Methods

### 3.1. Instruments and Chemicals

^1^H NMR, ^13^C NMR, and ^19^F NMR spectra were detected on a Bruker Biospin AG-400 (Bruker Optics, Ettlingen, Germany) or a JEOL-ECX500 (Akishima, Japan) apparatus with internal standard tetramethylsilane (TMS). High resolution mass spectrometry (HRMS) spectra were performed on a Q-Exactive Orbitrap MS apparatus (Thermo Fisher Scientific, Waltham, MA, USA). Scanning electron microscopy (SEM) images were obtained by using a Nova Nano SEM 450 instrument (FEI, USA, 200 kV). Fluorescent images were achieved under an Olympus-BX53 microscope (Olympus, Tokyo, Japan). ADDS-307 conductivity meter (INESA and Scientific Instrument Co., Ltd., Shanghai, China) was employed to record electrical conductivity. All of the chemicals (≥95%) used for reaction were purchased from Bide Pharmatech Ltd. (Shanghai, China).

### 3.2. Synthesis of the Target Molecules

For all compounds, we dissolved 4-*N*-Boc-aminopiperidine (0.50 g, 2.50 mmol) in 5 mL of *N*, *N*-dimethylformamide (DMF), then added K_2_CO_3_ (1.04 g, 7.49 mmol) and 1-bromodecane (774 µL, 3.74 mmol) and heated the mixture to 80 °C for 72 h to synthesize intermediate **1** [42,43]. After that, we obtained intermediate **2** by adding trifluoroacetic acid to intermediate **1**. Finally, we mixed intermediate **2** with various substituted sulfonyl chloride to obtain title molecules **A_1_**−**A_24_**. Similarly, we obtained the target compounds **B_1_** and **B_2_** via the reaction of intermediate **2** with the corresponding substituted benzoyl chloride [44,45]. We prepared compounds **B_3_**–**B_6_** via the condensation reaction between intermediate **2** (0.38 g, 1.58 mmol) and diverse aromatic acids (1.32 mmol) in the presence of 1-(3-dimethylaminopropyl)-3-ethylcarbodiimide hydrochloride (EDCI, 0.30 g, 1.58 mmol), 1-hydroxybenzotriazole (HOBt, 0.20 g, 1.45 mmol), and triethylamine (TEA, 274 µL, 1.98 mmol) in CH_2_Cl_2_ at room temperature [46,47]. Similarly, we mixed the raw material 4-amino-1-Boc-piperidine (5.00 g, 24.96 mmol) with 2-(trifluoromethyl) benzenesulfonyl chloride (5.95 mL, 37.45 mmol) and TEA (10.41 mL, 74.89 mmol) in CH_2_Cl_2_ at 0 °C to obtain intermediate **3**. Subsequently, we obtained intermediate **4** by adding trifluoroacetic acid to intermediate **3**. We obtained the target compounds **C_1_**–**C_6_** by performing a substitution reaction with intermediate **4** (0.40 g, 1.30 mmol) and different bromoalkanes (1.56 mmol), and K_2_CO_3_ (0.27 g, 1.95 mmol) in DMF at 80 °C. All the target molecular structures were characterized by ^1^H NMR, ^13^C NMR, ^19^F NMR, and HRMS; relevant detailed steps and the spectra information (Figures S4–S124) are provided in the Supporting Information.

### 3.3. Experimental Section

The antibacterial bioassays in vitro, growth curve assay, bacterial morphological study, and the relative electrical conductivity measurement were carried out according to our previous studies [48,49,50,51].

### 3.4. Molecular Docking Study

First, the primary gene sequence of *Xoo*DHPS was achieved in the National Center for Biotechnology Information (NCBI) (https://www.ncbi.nlm.nih.gov/, accessed on 21 October 2022). GenBank URQ81450.1 was imported into online program the SWISS MODEL (http://swissmodel.expasy.org/, accessed on 25 October 2020) for homology modeling, and 74.33% sequence identity with the protein 6DAY was observed. Then, the conformation of *Xoo*DHPS was optimized by energy minimization using the GROMOS 54A7 force field. Next, the SYBYL-X 2.0 was employed to conduct molecular automated docking simulations of compounds **C_4_** and sulfadiazine. Finally, the PyMol software was used to perform the docking results. 

### 3.5. Fluorescence Images of Propidium Iodide (PI)-Stained Xoo

Briefly, suspension *Xoo* cells were diluted to 0.2 (OD_595_). Subsequently, these *Xoo* cells were pre-incubated with molecule **C_4_** at concentrations of 4.04 µg mL^−1^ and 16.16 µg mL^−1^, and the same volume of dimethyl sulfoxide (solvent control) for 12 h at 28 °C. After the cultivation, the suspensions were centrifuged at 6500 rpm for 5 min and washed 2 times with phosphate buffer saline (PBS) (10 mM, pH 7.2). Then *Xoo* cells were resuspended in 100 µL of PBS buffer, and were stained with 10 µL of 10 µg mL^−1^ PI dye for in the dark 30 min at 28 °C. The samples were washed thrice with PBS to remove the superfluous PI dye and were imaged collected by using an Olympus-BX53 microscope.

### 3.6. Determination of Intracellular and Extracellular Protein Content

We cultured *Xoo* cells overnight, then adjusted the absorbance of this suspension to 0.2 (OD_595_). We then incubated these cells with compound **C_4_** (1.01, 2.02, 4.04, 8.08 µg mL^−1^) or the same volume of dimethyl sulfoxide (as the control) for 12 h at 28 °C. Next, we centrifuged the suspensions at 6500 rpm for 5 min and added 20 µL of sterile water, 125 µL of G250, and 5 µL of cell-free supernatant to wells of a 96-well plate to quantify extracellular proteins. Meanwhile, we lysed the *Xoo* cells by ultrasonic crushing (power 35%, ultrasound 5 s, interval 5 s, repeat 60 times at 0 °C), removed the *Xoo* debris by centrifugation at 10,000 rpm for 10 min, and prepared intracellular protein quantification as described above. Finally, we quantified intracellular and extracellular proteins (leaked proteins) using a standard Bradford assay [41].

### 3.7. Phytotoxicity Assay

The compound **C_4_** was provided to determine the phytotoxicity for rice leaves. The equal dose of DMSO was selected as the negative control. Rice variety “Xiangliangyou” was sowed for approximately 8 weeks to start testing phytotoxicity. First, the compound **C_4_**_,_ in concentrations of of 200 μg mL^−1^ and 500 μg mL^−1^, was evenly sprayed on the rice leaves until dripping down. Then, all of the inoculated plants were cultured in a controllable greenhouse (28 °C, relative humidity 90%), and the rice growth was observed to assess the phytotoxicity of the compound **C_4_** after 7 days.

### 3.8. Antibacterial Activity In Vivo against Rice Bacterial Leaf Blight

The compound **C_4_** was provided to determine the control efficiency against rice bacterial leaf blight in vivo. The commercialized agents BT and TC were selected as the positive controls, and an equal dose of DMSO was selected as the negative control. Rice variety “Xiangliangyou” was sown for approximately 8 weeks to start testing their curative and protective activity. For the curative effects, rice leaves of a distance of 1–2 cm from the leaf tips were cut off by scissors coated with *Xoo* (OD_595_ = 0.6–0.8). One day later, the compound **C_4_** of 200 μg mL^−1^ was evenly sprayed on the rice leaves until dripping down. Then, all of the inoculated plants were cultured in a controllable greenhouse (28 °C, relative humidity 90%), and the disease index was measured after 14 days. For the protective activity, the difference was that the same drug doses were first sprayed in leaves and the leaves were inoculated with *Xoo* cells a day later. Then, all of the inoculated plants were cultured in a controllable greenhouse (28 °C, relative humidity 90%), and the disease index was measured after 14 days. Then, the leaf was graded according to the percentage of the spot area in the entire leaf area: grade 1, proportion of less than 5%; grade 3, proportion of 6−10%; grade 5, proportion of 11−20%; grade 7, proportion of 21−50%; grade 9, proportion of more than 50%. Second, the disease index (C or T) was calculated according to the grade: Disease index (C or T) = ∑ (the number of leaves at each grade × the corresponding grade)/ (the total number of leaves × the superlative grade). Finally, the curative and protection activities were calculated using the following formula: Control efficiency I (%) = (C − T)/C × 100 (C was the disease index of the negative control and T was the disease index of the treatment group) [48].

## 4. Conclusions

In summary, we documented the bioactivity of a series of new sulfonamide compounds with an alkyl side chain tail and a piperidine link against two destructive plant pathogens. Bioassay results suggested that most of the title compounds displayed noteworthy bioactivity toward the two pathogens. Among them, compound **C_4_** exhibited extremely potent bioactivity toward *Xoo*, with an EC_50_ value of 2.02 µg mL^−1^ in vitro. In addition, compound **A_8_** displayed the best inhibitory activity against *Xac*, with an EC_50_ value of 4.74 µg mL^−1^. The SAR analysis suggested that the synergistic effects of the benzsulfamide skeleton and suitable alkyl chain played an essential role in antibacterial activity. The growth assay showed that compound **C_4_** possessed conspicuous bactericidal potency. The molecular docking results confirmed that the designed compounds were promising DHPS inhibitors. Additionally, SEM imaging, PI staining, electric conductivity, and protein leakage assays confirmed that compound **C_4_** significantly affected the diverse physiological processes of pathogens by changing cell morphology and irreversibly damaging the cell membrane, eventually causing bacterial death. Additionally, the in vivo experiment on rice bacterial blight demonstrated that compound **C_4_** efficiently suppressed the growth of *Xoo* in rice leaves with curative and protection activities of 34.78% and 39.83%, respectively, at 200 µg mL^−1^. Given these unique characteristics, these molecules are potential leads for the development of compounds concurrently targeting DHPS and destroying the cell membrane to effectively manage refractory plant bacterial diseases.

## Figures and Tables

**Table 1:** In vitro antibacterial activities of target compounds **A_1_**−**A_24_** against plant pathogens.

Compds	*Xoo*		*Xac*	
	Regression Equation	EC_50_^a^ (µg mL^−1^)	Regression Equation	EC_50_ ^a^ (µg mL^−1^)
**A_1_**	y = 1.4343x + 4.0238	4.79 ± 0.43	y = 1.8300x + 3.3715	7.76 ± 0.67
**A_2_**	y = 4.6625x + 2.0919	4.20 ± 0.10	y = 2.6383x + 3.0814	5.34 ± 0.30
**A_3_**	y = 5.6177x + 1.2286	4.69 ± 0.31	y = 4.9718x + 0.5371	7.90 ± 0.04
**A_4_**	y = 9.6320x − 2.5659	6.10 ± 0.02	y = 3.0552x + 2.3945	7.13 ± 0.23
**A_5_**	y = 4.9037x + 2.5394	3.18 ± 0.07	y = 2.0715x + 3.2566	6.94 ± 0.88
**A_6_**	y = 5.5240x + 1.7972	3.80 ± 0.02	y = 1.8178x + 3.4108	7.49 ± 1.02
**A_7_**	y = 5.6023x + 1.7953	3.73 ± 0.13	y = 0.6510x + 4.4427	7.18 ± 0.99
**A_8_**	y = 5.1991x + 2.4018	3.16 ± 0.18	y = 3.1487x + 2.8714	4.74 ± 0.14
**A_9_**	y = 8.4556x − 1.0261	5.16 ± 0.08	y = 3.9909x + 1.6175	7.04 ± 0.14
**A_10_**	y = 3.4044x + 3.5605	2.65 ± 0.07	y = 2.0645x + 2.6144	14.31 ± 0.59
**A_11_**	y = 7.4910x + 1.5325	2.90 ± 0.18	y = 7.8360x − 0.5968	5.18 ± 0.06
**A_12_**	y = 14.402x − 6.1575	5.95 ± 0.05	y = 4.2303x + 1.4896	6.76 ± 0.07
**A_13_**	y = 3.8692x + 1.7679	6.84 ± 0.10	y = 1.3363x + 3.7871	8.08 ± 0.29
**A_14_**	y = 3.4330x + 3.1455	3.47 ± 0.13	y = 1.8159x + 2.8787	14.73 ± 1.52
**A_15_**	y = 4.4933x + 1.9684	4.73 ± 0.16	y = 7.3320x − 1.3252	7.29 ± 0.50
**A_16_**	y = 2.2578x + 3.4263	4.98 ± 0.21	y = 7.1180x − 0.9297	6.81 ± 0.06
**A_17_**	y = 7.2855x + 1.8567	2.70 ± 0.05	y = 3.1753x + 2.5126	6.07 ± 0.24
**A_18_**	y = 5.0487x + 1.1615	5.76 ± 0.22	y = 2.0441x + 3.2704	7.02 ± 0.14
**A_19_**	y = 3.9981x + 2.7651	3.62 ± 0.16	y = 3.8577x + 0.7400	12.71 ± 0.57
**A_20_**	y = 8.6187x − 0.9974	4.96 ± 0.22	y = 2.7816x + 3.0557	5.00 ± 0.19
**A_21_**	y = 7.9264x + 1.2165	3.00 ± 0.20	y = 6.1030x − 0.9408	9.41 ± 0.13
**A_22_**	y = 6.6465x − 2.0600	11.54 ± 0.27	y = 1.5202x + 3.0320	19.71 ± 3.22
**A_23_**	y = 6.8367x − 2.3662	11.83 ± 0.17	y = 4.7402x − 0.6111	15.27 ± 0.81
**A_24_**	y = 6.0096x − 1.2526	10.98 ± 0.13	y = 1.5720x + 2.9129	21.26 ± 3.90
**SD ^b^**	/	>150	/	>150
**BT ^b^**	y = 4.3565x − 2.0887	42.38 ± 0.82	y = 4.3041x − 3.7955	110.54 ± 4.88
**TC ^b^**	y = 5.2027x − 4.4146	64.50 ± 0.45	y = 9.4597x − 14.7160	121.40 ± 2.60

^a^ EC_50_ values of antibacterial activities are indicated as the means ± SD (standard deviation) of three independent repetitions. ^b^ Commercialized antibacterial agents as positive controls. Abbreviations: SD, sulfadiazine; BT, bismerthiazol; TC, thiodiazole copper.

**Table 2:** In vitro antibacterial activities of target compounds **B_1_**−**B_6_** against plant pathogens.

Compds	*Xoo*		*Xac*	
	Regression Equation	EC_50_^a^ (µg mL^−1^)	Regression Equation	EC_50_ ^a^ (µg mL^−1^)
**B_1_**	y = 1.0841x + 3.4603	26.32 ± 1.90	y = 1.5204x + 2.7135	31.91 ± 2.38
**B_2_**	y = 1.2982x + 3.3740	17.89 ± 4.46	y = 1.7303x + 2.9865	14.58 ± 1.37
**B_3_**	y = 0.7878x + 3.7917	34.18 ± 2.49	/	>150
**B_4_**	y = 1.9602x + 3.1950	8.33 ± 0.88	y = 1.9231x + 2.7285	15.18 ± 1.05
**B_5_**	y = 10.1760x − 6.9093	14.80 ± 0.23	y = 1.5972x + 3.2558	12.36 ± 0.14
**B_6_**	/	>150	/	>150
**SD ^b^**	/	>150	/	>150
**BT ^b^**	y = 4.3565x − 2.0887	42.38 ± 0.82	y = 4.3041x − 3.7955	110.54 ± 4.88
**TC ^b^**	y = 5.2027x − 4.4146	64.50 ± 0.45	y = 9.4597x − 14.7160	121.40 ± 2.60

^a^ EC_50_ values of antibacterial activities are indicated as the means ± SD (standard deviation) of three independent repetitions. ^b^ Commercialized antibacterial agents as positive controls. Abbreviations: SD, sulfadiazine; BT, bismerthiazol; TC, thiodiazole copper.

**Table 3:** In vitro antibacterial activities of target molecules **C_1_**−**C_6_** against plant pathogens.

Compds	*Xoo*		*Xac*	
	Regression Equation	EC_50_^a^ (µg mL^−1^)	Regression Equation	EC_50_ ^a^ (µg mL^−1^)
**C_1_**	y = 1.8612x + 1.9174	45.32 ± 5.39	y = 2.3393x + 0.8311	60.55 ± 1.64
**C_2_**	y = 3.1093x + 0.7133	23.92 ± 0.77	y = 3.1899x + 0.8925	19.39 ± 0.42
**C_3_**	y = 5.7483x + 1.6495	3.83 ± 0.02	y = 2.7728x + 2.4090	8.60 ± 0.60
**C_4_**	y = 5.4304x + 3.3394	2.02 ± 0.22	y = 2.2438x + 2.7277	10.30 ± 0.36
**C_5_**	y = 2.1944x + 2.4490	14.54 ± 0.63	y = 4.7345x − 0.9608	18.16 ± 0.81
**C_6_**	/	>150	/	>150
**SD ^b^**	/	>150	/	>150
**BT ^b^**	y = 4.3565x − 2.0887	42.38 ± 0.82	y = 4.3041x − 3.7955	110.54 ± 4.88
**TC ^b^**	y = 5.2027x − 4.4146	64.50 ± 0.45	y = 9.4597x − 14.7160	121.40 ± 2.60

^a^ EC_50_ values of antibacterial activities are indicated as the means ± SD (standard deviation) of three independent repetitions. ^b^ Commercialized antibacterial agents as positive controls. Abbreviations: SD, sulfadiazine; BT, bismerthiazol; TC, thiodiazole copper.

**Table 4:** In vivo control efficiency of compound **C_4_** against rice bacterial blight under greenhouse conditions at 200 µg mL^−1^.

Treatment	Curative Activity (14 Days after Spraying)			Protective Activity (14 Days after Spraying)		
	Morbidity (%)	Disease Index (%)	Control Efficiency (%)	Morbidity (%)	Disease Index (%)	Control Efficiency (%)
**C_4_**	100	55.56	34.78	100	52.59	39.83
BT	100	56.30	33.91	100	54.07	38.14
TC	100	60.00	29.57	100	60.74	30.51
Control	100	85.19		100	87.41	

## Data Availability

Not applicable.

## Supplementary Materials

The following supporting information can be downloaded at: https://www.mdpi.com/article/10.3390/ijms24065861/s1.
