# Supplementary material for: Novel Sulfonamide Derivatives Containing a Piperidine Moiety as New Bactericide Leads for Managing Plant Bacterial Diseases
Source: Int J Mol Sci. 2023 Mar 20;24(6):5861. doi: 10.3390/ijms24065861 (PMC10054644; doi:10.3390/ijms24065861)
Supplement: Supplementary file 1 [file ijms-24-05861-s001.zip › ijms-2287486-supplementary.pdf]

Supporting Information

Novel Sulfonamide Derivatives Containing a Piperidine Moiety as New Bactericide Leads for Managing Plant Bacterial Diseases

Jiao Xie, Zhou-Qing Long, Ai-Qun Chen, Ying-Guo Ding, Shi-Tao Liu, Xiang Zhou*, Li-Wei Liu, Song Yang*

National Key Laboratory of Green Pesticide, Key Laboratory of Green Pesticide and Agricultural Bioengineering, Ministry of Education, Center for R&D of Fine Chemicals of Guizhou University, Guiyang, 550025, China.

* Corresponding author.

E-mail: jhzx.msm@gmail.com (S. Yang) or syang@gzu.edu.cn; xiangzhou@gzu.edu.cn or zhoux1534@163.com (X. Zhou)

Contents

1. Synthesis for the intermediates and target compounds 2

2. Homologous modelling..4

3. Fluorescence images of C_4-treated *Xoo* cells stained with PI.................................6

4. Effect of C_4 on protein concentration 6

5. Characterization data for tatget compounds 7

6. ^{1}H NMR, ^{13}C NMR, ^{19}F NMR and HRMS spectra analysis of A_1-A_{24}, B_1-B_6, and C_1-C_6..29

1. Synthesis for the intermediates and target compounds

1.1 General procedures for preparing intermediate **1**

To a 15 mL pressure-proof pipe, 4-*N*-Boc-aminopiperidine (0.50 g, 2.50 mmol) and K_2CO_3 (1.04 g, 7.49 mmol) were added. Then DMF (4 mL) and 1-bromodecane (774 μL, 3.74 mmol) were added and stirred at 80 °C for 3 days. Upon complete consumption of the starting materials, the reaction mixture was quenched with saturated aqueous solution of NH_4Cl (10 mL), extracted with CH_2Cl_2 (50 mL × 3). The organic layers were then dried over anhydrous sodium sulfate and concentrated in vacuo to obtain crude product. The residue was purified by column chromatography on silica gel with a mixture of dichloromethane and methanol (50:1, v/v) to afford 0.7486 g (yield 88.1%) of a white solid intermediate **1**.

1.2 General procedures for preparing intermediate **2**

intermediate **1** (0.50 g, 1.47 mmol) was added into a 50 mL round-bottomed, then dichloromethane (CH_2Cl_2) (15 mL) and trifluoroacetic acid (CF_3COOH) (2 mL) was added. The mixture was stirred at 0 °C for 5 h and the reaction was finished through TLC. The mixture was concentrated in vacuo to obtain crude compound **2**.

1.3 General procedures for preparing compounds **A_1-A_{24}**

The crude compound **2** was dissolved in CH_2Cl_2 (15 mL) and triethylamine (550 μL, 3.96 mmol) was added. Then various acyl chloride substituted compounds (1.58 mmol) was added dropwise at 0 °C within about 5 min. After that, the reaction continues stirring for another 48 h at room temperature. The mixture was dissolved in 75 mL

CH_2Cl_2, washed three times with 25 mL water. The organic layers were then dried over anhydrous sodium sulfate and concentrated in vacuo to obtain crude product. The residue was purified by column chromatography on silica gel with a mixture of dichloromethane / methanol (100:1-50:1, v/v) to give compounds **A_1-A_{24}**.

1.4 General procedures for preparing target compounds **B_1-B_6**.

Similar to step 1.3, we obtained the target compounds **B_1** and **B_2** *via* the reaction of intermediate **2** with the corresponding substituted benzoyl chloride.

The crude intermediate **2** was dissolved in CH_2Cl_2 (15 mL) and EDCI (0.30 g, 1.58 mmol), HOBt (0.20 g, 1.45 mmol), and triethylamine (550 μL, 3.96 mmol) was added successively. Then the mixture was stirred at room temperature for 48 h. After the reaction is completed, the filtrate was evaporated using a rotary evaporator and extracted with CH_2Cl_2 (75 mL), the organic layer was washed by water, dried with anhydrous sodium sulfate, and followed by the removal of the solvent under vacuum. Finally, the residue was purified by column chromatography on silica gel with a mixture of dichloromethane / methanol (100:1-30:1, v/v) to give compounds **B_3-B_6**.

1.5 General procedures for preparing intermediate **3**

To a 250 mL round-bottomed flask, 4-amino-1-Boc-piperidine (5.00 g, 24.96 mmol) were dissolved in 20 mL of CH_2Cl_2 and triethylamine (TEA, 10.41 mL, 74.89 mmol) was added, and then 2-(trifluoromethyl) benzenesulfonyl chloride (5.95 mL, 37.45 mmol) was added dropwise in the ice bath. The mixture was reacted for 48 h. After that, 200 mL of CH_2Cl_2 was added into the reaction system and the organic layer was water washed by (50 mL × 3) and dried with anhydrous sodium sulfate, and then

the solvent was removed under vacuum. Finally, the residue was purified by column chromatography on silica gel with a mixture of petroleum ether / ethyl acetate (10:1, v/v) to give intermediate **3**.

1.6 General procedures for preparing intermediate **4**

intermediates **3** (8.60 g, 21.06 mmol) was added into a 250 mL round-bottomed, then dichloromethane (CH_2Cl_2) (100 mL) and trifluoroacetic acid (CF_3COOH) (20 mL) was added. The mixture was stirred at 0 °C for 5 h and the reaction was found completed through TLC. The mixture was concentrated in vacuo to obtain intermediate **4**.

1.7 General procedures for preparing target compounds **C_1-C_6**

Intermediate **4** (0.40 g, 1.30 mmol), bromoalkanes (R_4-Br) (1.56 mmol), K_2CO_3 (0.27 g, 1.95 mmol), and *N*, *N*-dimethylformamide (4.0 mL) were added to a 15 mL pressure bottle and stirred at 80 °C until intermediate **4** had reacted completely. After that, the reaction mixture was quenched with water (20 mL) and extracted with ethyl acetate (50 mL × 3). The organic layer was dried with anhydrous sodium sulfate and evaporated under vacuum. Finally, the residue was purified by column chromatography on silica gel with a mixture of dichloromethane / methanol (80:1-60:1, v/v) to give compounds **C_1-C_6**.

2. Homologous modelling

The quality resulting of the structure model was assessed using the SAVES v5.0 server (http://www.ebi.ac.uk/thornton-srv/databases/pdbsum/Generate.html). While the Ramachandran plot was used to validate the 3D structures.

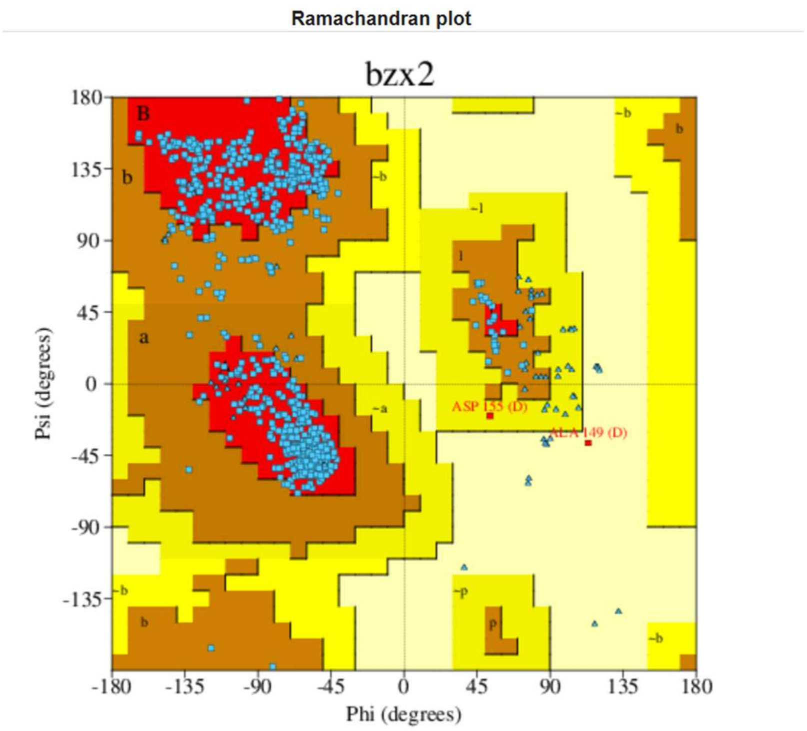

Figure S1. Pull-pattern diagram of homologous modeled proteins (6DAY).

Plot statistics		
Residues in most favored regions [A, B, L]	880	91.0%
Residues in additional allowed regions [A, B, 1, p]	85	8.8%
Residues in generously allowed regions [~a, ~b, ~l, ~p]	1	0.1%
Residues in disallowed regions	1	0.1%
Number of non-glycine and non-proline residues	967	100%
Number of end-residues (excl. Gly and Pro)	12	
Number of glycine residues	88	
Number of proline residues	56	
Total number of residues	1123	

3. Fluorescence images of C4-treated *Xoo* cells stained with PI

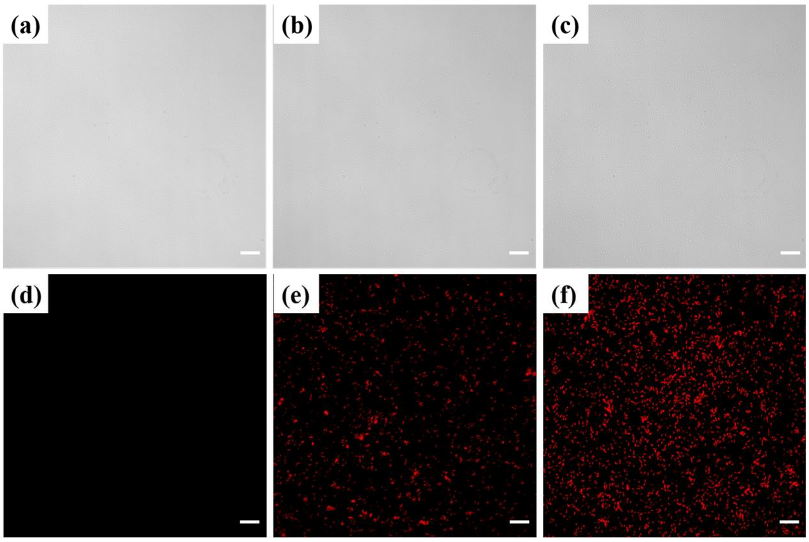

Figure S2. Fluorescence images of **C4**-treated *Xoo* cells stained with PI: (a, d) 0 μg mL^{-1}: Control, (b, e) 4.04 μg mL^{-1}, and (c, f) 16.16 μg mL^{-1} of **C4**. Scale bars: 10 μm.

4. Effect of C4 on protein concentration

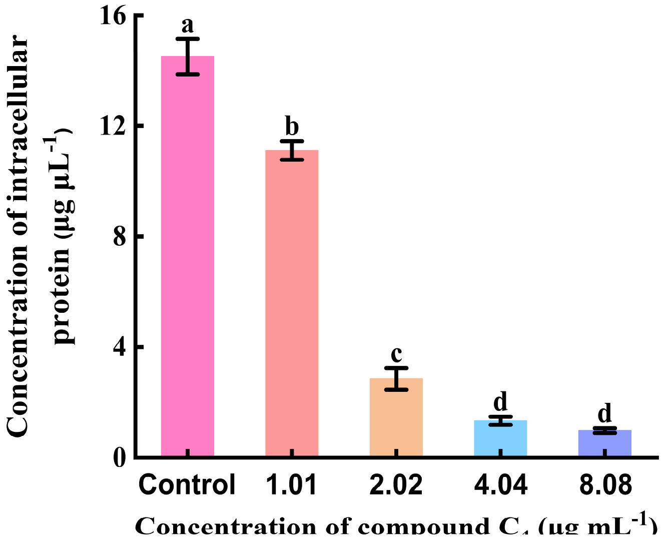

Figure S3. Intracellular protein content in *Xoo* cells treated with **C4** (0, 1.01, 2.02, 4.04,

and 8.08 μg mL^{-1}). Bars indicate the mean ± SD. Lowercase letters indicate significant differences between the different treatment groups, as calculated using one-way ANOVA ($P < 0.05$).

5. Characterization data for tatget compounds

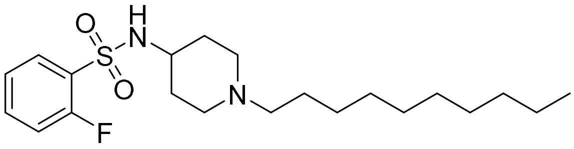

***N*-(1-decylpiperidin-4-yl)-2-fluorobenzenesulfonamide (A_1).** A yellow solid, yield 28.1%, m. p. 67.3-68.0 °C; ^{1}H NMR (500 MHz, $CDCl_3$) δ 7.90 (td, J = 7.6, 1.7 Hz, 1H, ph-6H), 7.60 – 7.54 (m, 1H, ph-4H), 7.29 – 7.25 (m, 1H, ph-3H), 7.22 – 7.17 (m, 1H, ph-5H), 4.86 (d, J = 2.5 Hz, 1H, $-NHSO_2-$), 3.24 (s, 1H, piperidin-CH), 2.75 (d, J = 8.9 Hz, 2H, piperidin-CH_2), 2.25 (dd, J = 8.9, 6.8 Hz, 2H, $-NCH_2-$), 1.97 (d, J = 9.3 Hz, 2H, piperidin-CH_2), 1.80 – 1.74 (m, 2H, piperidin-CH_2), 1.50 (ddd, J = 14.1, 10.9, 3.7 Hz, 2H, piperidin-CH_2), 1.41 (dd, J = 14.7, 7.4 Hz, 2H, $-NCH_2\underline{CH_2}-$), 1.27 – 1.21 (m, 14H, $-NCH_2CH_2\underline{(CH_2)_7}CH_3$), 0.86 (t, J = 7.0 Hz, 3H, $-CH_3$); ^{13}C NMR (126 MHz, $CDCl_3$) δ 158.8 (d, ^{1}J = 253.6 Hz, C-F), 135.0 (d, ^{3}J = 8.5 Hz, C-F), 130.1, 129.4 (d, ^{2}J = 13.4 Hz, C-F), 124.6 (d, ^{4}J = 3.6 Hz, C-F), 117.0 (d, ^{2}J = 21.1 Hz, C-F), 58.7, 52.0, 51.1, 32.9, 32.0, 29.7, 29.6, 29.4, 27.6, 27.0, 22.8, 14.2; ^{19}F NMR (471 MHz, $CDCl_3$) δ -110.7; HRMS (ESI) $[M+H]^+$ calcd for $C_{21}H_{36}O_2N_2FS$: 399.2476, found: 399.2470.

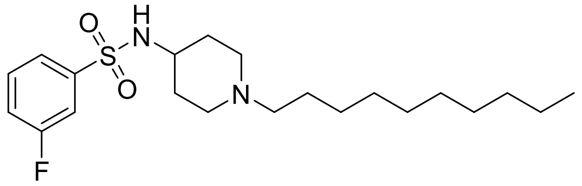

***N*-(1-decylpiperidin-4-yl)-3-fluorobenzenesulfonamide (A2)**. A gray solid, yield 38.3%, m. p. 74.0-74.7 °C; ^{1}H NMR (400 MHz, $CDCl_3$) δ 7.68 (ddd, J = 7.8, 1.5, 1.0 Hz, 1H, ph-2H), 7.62 – 7.56 (m, 1H, ph-6H), 7.49 (td, J = 8.1, 5.3 Hz, 1H, ph-5H), 7.30 – 7.22 (m, 1H, ph-4H), 3.26 – 3.13 (m, 1H, piperidin-CH), 2.75 (d, J = 11.7 Hz, 2H, piperidin-CH_2), 2.30 – 2.21 (m, 2H, -NCH_2-), 1.99 (t, J = 10.7 Hz, 2H, piperidin-CH_2), 1.77 (dd, J = 13.4, 2.9 Hz, 2H, piperidin-CH_2), 1.50 (ddd, J = 14.0, 10.8, 3.6 Hz, 2H, piperidin-CH_2), 1.41 (dd, J = 7.7, 6.3 Hz, 2H, -$NCH_2\underline{CH_2}$-), 1.23 (s, 14H, -$NCH_2CH_2\underline{(CH_2)_7}CH_3$), 0.86 (t, J = 6.9 Hz, 3H, -CH_3); ^{13}C NMR (101 MHz, $CDCl_3$) δ 162.4 (d, ^{1}J = 251.5 Hz, C-F), 143.5 (d, ^{3}J = 6.6 Hz, C-F), 131.0 (d, ^{3}J= 7.9 Hz, C-F), 122.6 (d, ^{4}J= 3.2 Hz, C-F), 119.8 (d, ^{2}J = 21.3 Hz, C-F), 114.3 (d, ^{2}J = 24.5 Hz, C-F), 58.6, 51.9, 51.0, 32.9, 31.9, 29.6, 29.5, 29.3, 27.6, 27.0, 22.7, 14.1; ^{19}F NMR (376 MHz, $CDCl_3$) δ -109.6; HRMS (ESI) $[M+H]^+$ calcd for $C_{21}H_{36}O_2N_2FS$: 399.2476, found: 399.2472.

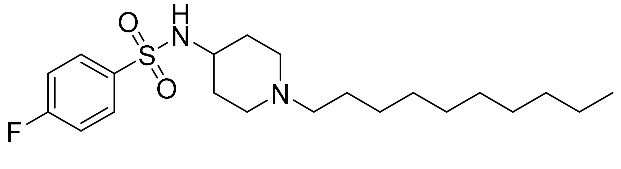

***N*-(1-decylpiperidin-4-yl)-4-fluorobenzenesulfonamide (A3)**. A yellow solid, yield 44.9%, m. p. 55.1-56.5 °C; ^{1}H NMR (500 MHz, $CDCl_3$) δ 7.92 – 7.87 (m, 2H, ph-2,6H), 7.20 – 7.14 (m, 2H, ph-3,5H), 5.00 (s, 1H, -$NHSO_2$-), 3.15 (s, 1H, piperidin-CH), 2.75 (d, J = 8.2 Hz, 2H, piperidin-CH_2), 2.25 (dd, J = 8.9, 6.8 Hz, 2H, -NCH_2-), 1.98 (s, 2H, piperidin-CH_2), 1.78 – 1.71 (m, 2H, piperidin-CH_2), 1.48 (ddd, J = 14.1,

11.0, 3.7 Hz, 2H, piperidin-CH2), 1.41 (dd, J = 14.7, 7.4 Hz, 2H, -NCH2<u>CH2</u>-), 1.26 – 1.21 (m, 14H, -NCH2CH2<u>(CH2)7</u>CH3), 0.85 (t, J = 7.0 Hz, 3H, -CH3); ^{13}C NMR (126 MHz, CDCl3) δ 165.0 (d, ^{1}J = 254.6 Hz, C-F), 137.4 (d, ^{4}J = 2.8 Hz, C-F), 129.7 (d, ^{3}J = 9.2 Hz, C-F), 116.4 (d, ^{2}J = 22.5 Hz, C-F), 58.7, 52.0, 50.9, 32.9, 32.0, 29.7, 29.6, 29.4, 27.6, 27.0, 22.8, 14.2; ^{19}F NMR (471 MHz, CDCl3) δ -105.4; HRMS (ESI) $[M+H]^+$ calcd for $C_{21}H_{36}O_2N_2FS$: 399.2476, found: 399.2471.

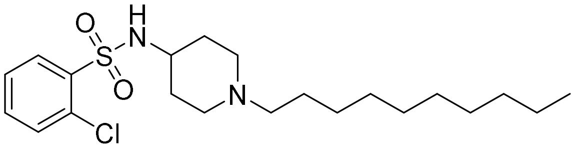

2-chloro-*N*-(1-decylpiperidin-4-yl)benzenesulfonamide (A4). A yellow solid, yield 34.4%, m. p. 56.1-57.0 °C; ^{1}H NMR (400 MHz, CDCl3) δ 8.10 (dd, J = 8.3, 1.1 Hz, 1H, ph-6H), 7.54 – 7.47 (m, 2H, ph-3,4H), 7.41 (ddd, J = 7.9, 6.0, 2.7 Hz, 1H, ph-5H), 5.03 (d, J = 5.1 Hz, 1H, -NHSO2-), 3.17 (d, J = 3.9 Hz, 1H, piperidin-CH), 2.70 (d, J = 11.7 Hz, 2H, piperidin-CH2), 2.23 (dd, J = 8.8, 6.7 Hz, 2H, -NCH2-), 1.96 (t, J = 10.6 Hz, 2H, piperidin-CH2), 1.77 – 1.68 (m, 2H, piperidin-CH2), 1.49 (ddd, J = 23.7, 10.6, 3.7 Hz, 2H, piperidin-CH2), 1.41 (dd, J = 14.8, 7.0 Hz, 2H, -NCH2<u>CH2</u>-), 1.25 (d, J = 18.8 Hz, 14H, -NCH2CH2<u>(CH2)7</u>CH3), 0.86 (t, J = 6.9 Hz, 3H, -CH3); ^{13}C NMR (101 MHz, CDCl3) δ 138.6, 133.6, 131.6, 131.3, 130.9, 127.3, 58.6, 51.9, 51.2, 32.7, 31.9, 29.6, 29.5, 29.3, 27.6, 27.0, 22.7, 14.1; HRMS (ESI) $[M+H]^+$ calcd for $C_{21}H_{36}O_2N_2ClS$: 415.2181, found: 415.2177.

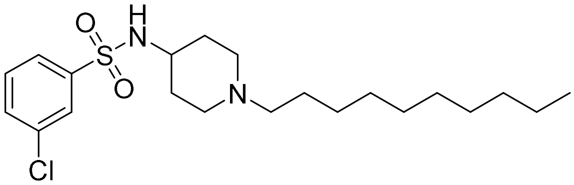

3-chloro-*N*-(1-decylpiperidin-4-yl)benzenesulfonamide (A5). A yellow solid,

yield 30.6%, m. p. 48.5-49.2 °C; ^{1}H NMR (400 MHz, $CDCl_3$) δ 7.87 (t, *J* = 1.8 Hz, 1H, ph-2H), 7.76 (ddd, *J* = 7.8, 1.6, 1.2 Hz, 1H, ph-6H), 7.53 (ddd, *J* = 8.0, 2.0, 1.1 Hz, 1H, ph-4H), 7.44 (t, *J* = 7.9 Hz, 1H, ph-5H), 4.86 (s, 1H, $-NHSO_2-$), 3.24 – 3.13 (m, 1H, piperidin-CH), 2.74 (d, *J* = 11.7 Hz, 2H, piperidin-CH_2), 2.25 (dd, *J* = 8.9, 6.7 Hz, 2H, $-NCH_2-$), 1.99 (t, *J* = 10.8 Hz, 2H, piperidin-CH_2), 1.82 – 1.72 (m, 2H, piperidin-CH_2), 1.49 (ddd, *J* = 23.9, 10.8, 3.6 Hz, 2H, piperidin-CH_2), 1.41 (dd, *J* = 8.8, 5.4 Hz, 2H, -$NCH_2\underline{CH_2}-$), 1.26 (d, *J* = 18.0 Hz, 14H, $-NCH_2CH_2\underline{(CH_2)_7}CH_3$), 0.86 (t, *J* = 6.9 Hz, 3H, $-CH_3$); ^{13}C NMR (101 MHz, $CDCl_3$) δ 143.2, 135.3, 132.7, 130.4, 127.0, 125.0, 58.6, 52.0, 51.1, 33.0, 31.9, 29.6, 29.5, 29.3, 27.6, 27.0, 22.7, 14.1; HRMS (ESI) $[M+H]^+$ calcd for $C_{21}H_{36}O_2N_2ClS$: 415.2181, found: 415.2175.

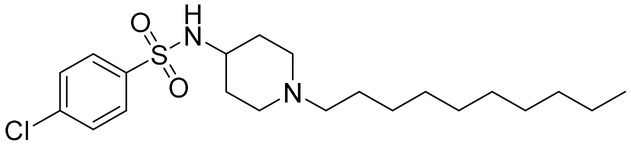

4-chloro-*N*-(1-decylpiperidin-4-yl)benzenesulfonamide (A_6). A yellow solid, yield 31.5%, m. p. 71.0-72.2 °C; ^{1}H NMR (400 MHz, $CDCl_3$) δ 7.85 – 7.78 (m, 2H, ph-2,6H), 7.50 – 7.44 (m, 2H, ph-3,5H), 4.84 (s, 1H, $-NHSO_2-$), 3.22 – 3.10 (m, 1H, piperidin-CH), 2.74 (d, *J* = 11.7 Hz, 2H, piperidin-CH_2), 2.24 (dd, *J* = 8.9, 6.7 Hz, 2H, $-NCH_2-$), 1.97 (t, *J* = 10.8 Hz, 2H, piperidin-CH_2), 1.80 – 1.70 (m, 2H, piperidin-CH_2), 1.53 – 1.36 (m, 4H, piperidin-CH_2 and $-NCH_2\underline{CH_2}-$), 1.29 – 1.20 (m, 14H, -$NCH_2CH_2\underline{(CH_2)_7}CH_3$), 0.86 (t, *J* = 6.9 Hz, 3H, $-CH_3$); ^{13}C NMR (101 MHz, $CDCl_3$) δ 139.9, 139.0, 129.4, 128.4, 58.6, 52.0, 51.0, 33.0, 32.0, 29.6, 29.5, 29.3, 27.6, 27.0, 22.7, 14.1; HRMS (ESI) $[M+H]^+$ calcd for $C_{21}H_{36}O_2N_2ClS$: 415.2181, found: 415.2174.

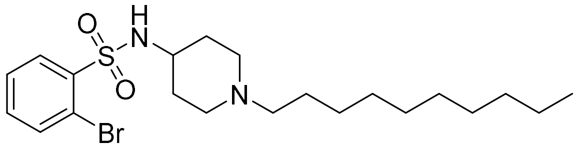

2-bromo-*N*-(1-decylpiperidin-4-yl)benzenesulfonamide (A_7). A yellow solid, yield 24.5%, m. p. 57.8-58.5 °C; ^{1}H NMR (500 MHz, $CDCl_3$) δ 8.14 (dd, *J* = 7.7, 1.0 Hz, 1H, ph-3H), 7.72 (d, *J* = 7.7 Hz, 1H, ph-6H), 7.46 (t, *J* = 7.4 Hz, 1H, ph-4H), 7.40 (td, *J* = 7.5, 1.2 Hz, 1H, ph-5H), 5.24 (s, 1H, $-NHSO_2-$), 3.17 (s, 1H, piperidin-CH), 2.74 (d, *J* = 9.5 Hz, 2H, piperidin-CH_2), 2.32 – 2.21 (m, 2H, $-NCH_2-$), 2.00 (s, 2H, piperidin-CH_2), 1.75 (d, *J* = 10.8 Hz, 2H, piperidin-CH_2), 1.54 (td, *J* = 12.9, 2.9 Hz, 2H, piperidin-CH_2), 1.41 (s, 2H, $-NCH_2\underline{CH_2}-$), 1.25 (d, *J* = 22.2 Hz, 14H, $-NCH_2CH_2\underline{(CH_2)_7}CH_3$), 0.85 (t, *J* = 6.9 Hz, 3H, $-CH_3$); ^{13}C NMR (126 MHz, $CDCl_3$) δ 140.1, 135.2, 133.7, 131.2, 128.0, 119.8, 58.6, 51.8, 51.0, 32.5, 32.0, 29.6, 29.6, 29.4, 27.6, 26.9, 22.6, 14.2; HRMS (ESI) $[M+H]^+$ calcd for $C_{21}H_{36}O_2N_2{}^{81}BrS$: 461.1655, found: 461.1649.

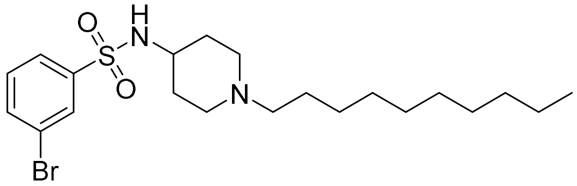

3-bromo-*N*-(1-decylpiperidin-4-yl)benzenesulfonamide (A_8). A yellow solid, yield 46.3%, m. p. 65.1-66.2 °C; ^{1}H NMR (400 MHz, $CDCl_3$) δ 8.03 (t, *J* = 1.8 Hz, 1H, ph-2H), 7.81 (ddd, *J* = 7.9, 1.7, 1.0 Hz, 1H, ph-4H), 7.69 (ddd, *J* = 8.0, 1.9, 1.0 Hz, 1H, ph-6H), 7.38 (t, *J* = 7.9 Hz, 1H, ph-5H), 4.81 (s, 1H, $-NHSO_2-$), 3.25 – 3.12 (m, 1H, piperidin-CH), 2.74 (d, *J* = 11.7 Hz, 2H, piperidin-CH_2), 2.25 (dd, *J* = 8.9, 6.7 Hz, 2H, $-NCH_2-$), 1.98 (t, *J* = 10.8 Hz, 2H, piperidin-CH_2), 1.82 – 1.72 (m, 2H, piperidin-CH_2), 1.48 (ddd, *J* = 24.2, 10.9, 3.8 Hz, 2H, piperidin-CH_2), 1.43 – 1.35 (m, 2H, $-NCH_2\underline{CH_2}-$),

1.29 – 1.20 (m, 14H, $-NCH_2CH_2\underline{(CH_2)_7}CH_3$), 0.86 (t, *J* = 6.9 Hz, 3H, $-CH_3$); ^{13}C NMR (101 MHz, $CDCl_3$) δ 143.3, 135.6, 130.7, 129.8, 125.4, 123.1, 58.6, 52.0, 51.1, 33.0, 31.9, 29.6, 29.6, 29.3, 27.6, 27.0, 22.7, 14.1; HRMS (ESI) $[M+H]^+$ calcd for $C_{21}H_{36}O_2N_2{}^{81}BrS$: 461.1655, found: 461.1648.

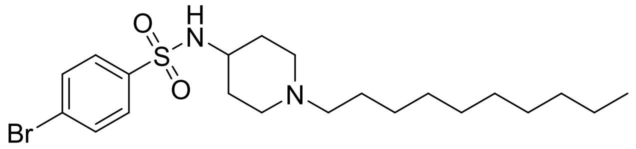

4-bromo-*N*-(1-decylpiperidin-4-yl) benzenesulfonamide (A_9). A white solid, yield 41.0%, m. p. 82.0-83.1 °C; ^1H NMR (400 MHz, $CDCl_3$) δ 7.77 – 7.72 (m, 2H, ph-2,6H), 7.66 – 7.61 (m, 2H, ph-3,5H), 4.90 (s, 1H, $-NHSO_2-$), 3.22 – 3.11 (m, 1H, piperidin-CH), 2.75 (d, *J* = 11.7 Hz, 2H, piperidin-CH_2), 2.25 (dd, *J* = 8.8, 6.8 Hz, 2H, $-NCH_2-$), 1.98 (t, *J* = 10.9 Hz, 2H, piperidin-CH_2), 1.76 (dd, *J* = 13.0, 3.3 Hz, 2H, piperidin-CH_2), 1.49 (ddd, *J* = 14.4, 11.1, 3.8 Hz, 2H, piperidin-CH_2), 1.41 (dd, *J* = 7.9, 6.4 Hz, 2H, $-NCH_2\underline{CH_2}-$), 1.23 (s, 14H, $-NCH_2CH_2\underline{(CH_2)_7}CH_3$), 0.86 (t, *J* = 6.9 Hz, 3H, $-CH_3$); ^{13}C NMR (101 MHz, $CDCl_3$) δ 140.5, 132.4, 128.5, 127.5, 58.6, 52.0, 51.0, 32.9, 31.9, 29.6, 29.5, 29.3, 27.6, 27.0, 22.7, 14.1; HRMS (ESI) $[M+H]^+$ calcd for $C_{21}H_{36}O_2N_2{}^{81}BrS$: 461.1655, found: 461.1649.

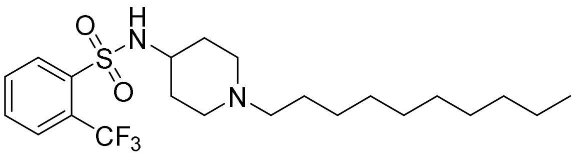

***N*-(1-decylpiperidin-4-yl)-2-(trifluoromethyl)benzenesulfonamide (A_{10})**. A yellow solid, yield 27.1%, m. p. 91.9-92.7 °C; ^1H NMR (400 MHz, $CDCl_3$) δ 8.28 – 8.22 (m, 1H, ph-3H), 7.86 (ddd, *J* = 9.2, 5.0, 2.4 Hz, 1H, ph-6H), 7.74 – 7.66 (m, 2H, ph-4,5H), 4.77 (d, *J* = 3.3 Hz, 1H, $-NHSO_2-$), 3.28 – 3.18 (m, 1H, piperidin-CH), 2.73

(d, J = 10.9 Hz, 2H, piperidin-CH_2), 2.30 – 2.19 (m, 2H, -NCH_2-), 1.97 (t, J = 10.1 Hz, 2H, piperidin-CH_2), 1.72 (dd, J = 13.2, 2.9 Hz, 2H, piperidin-CH_2), 1.53 – 1.35 (m, 4H, piperidin-CH_2 and -$NCH_2\underline{CH_2}$-), 1.29 – 1.19 (m, 14H, -$NCH_2CH_2\underline{(CH_2)_7}CH_3$), 0.85 (t, J = 6.8 Hz, 3H, -CH_3); ^{13}C NMR (101 MHz, $CDCl_3$) δ 140.0, 132.7, 132.5, 131.3, 128.6 (q, ^3J = 6.1 Hz, -CF_3), 127.3 (q, ^2J = 32.8 Hz, -CF_3), 123.0 (q, ^1J = 273.8 Hz, -CF_3), 58.6, 51.9, 51.2, 32.8, 31.9, 29.6, 29.5, 29.3, 27.5, 26.9, 22.7, 14.2; ^{19}F NMR (376 MHz, $CDCl_3$) δ -57.8; HRMS (ESI) $[M+H]^+$ calcd for $C_{22}H_{36}O_2N_2F_3S$: 449.2444, found: 449.2437.

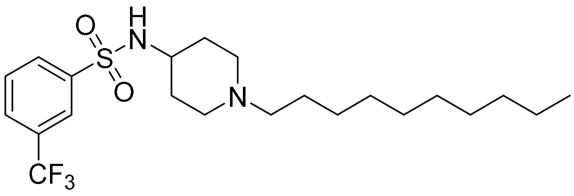

***N*-(1-decylpiperidin-4-yl)-3-(trifluoromethyl)benzenesulfonamide (A_{11})**. A yellow solid, yield 39.3%, m. p. 67.2-68.4 °C; ^1H NMR (400 MHz, $CDCl_3$) δ 8.15 (s, 1H, ph-2H), 8.08 (d, J = 7.9 Hz, 1H, ph-4H), 7.82 (d, J = 7.8 Hz, 1H, ph-6H), 7.66 (t, J = 7.9 Hz, 1H, ph-5H), 3.27 – 3.15 (m, 1H, piperidin-CH), 2.75 (d, J = 11.7 Hz, 2H, piperidin-CH_2), 2.25 (dd, J = 8.9, 6.7 Hz, 2H, -NCH_2-), 1.98 (t, J = 10.8 Hz, 2H, piperidin-CH_2), 1.81 – 1.70 (m, 2H, piperidin-CH_2), 1.50 (ddd, J = 14.3, 11.0, 3.7 Hz, 2H, piperidin-CH_2), 1.41 (dd, J = 8.5, 5.4 Hz, 2H, -$NCH_2\underline{CH_2}$-), 1.29 – 1.19 (m, 14H, -$NCH_2CH_2\underline{(CH_2)_7}CH_3$), 0.86 (t, J = 6.9 Hz, 3H, -CH_3); ^{13}C NMR (101 MHz, $CDCl_3$) δ 142.8, 131.8 (q, ^2J = 33.4 Hz, -CF_3), 130.1, 129.9, 129.2 (q, ^3J = 3.5 Hz, -CF_3), 124.0 (q, ^3J = 3.8 Hz, -CF_3), 123.2 (q, ^1J = 272.9 Hz, -CF_3), 58.6, 52.0, 51.2, 33.0, 31.9, 29.6, 29.5, 29.3, 27.6, 27.0, 22.7, 14.1; ^{19}F NMR (376 MHz, $CDCl_3$) δ -62.8; HRMS (ESI) $[M+H]^+$ calcd for $C_{22}H_{36}O_2N_2F_3S$: 449.2444, found: 449.2438.

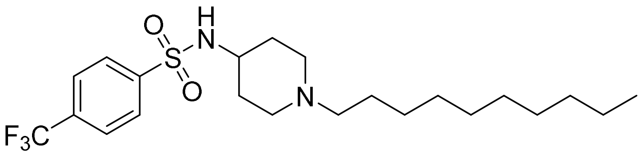

***N*-(1-decylpiperidin-4-yl)-4-(trifluoromethyl)benzenesulfonamide (A$_{12}$)**. A white solid, yield 29.2%, m. p. 87.6-88.4 °C; ^{1}H NMR (500 MHz, $CDCl_3$) δ 8.07 – 7.96 (m, 2H, ph-3,5H), 7.83 – 7.72 (m, 2H, ph-2,6H), 3.21 (s, 1H, piperidin-CH), 2.80 (d, *J* = 7.3 Hz, 2H, piperidin-CH_2), 2.32 – 2.24 (m, 2H, -NCH_2-), 2.01 (s, 2H, piperidin-CH_2), 1.77 (d, *J* = 11.5 Hz, 2H, piperidin-CH_2), 1.53 (dd, *J* = 21.1, 10.2 Hz, 2H, piperidin-CH_2), 1.42 (s, 2H, -$NCH_2\underline{CH_2}$-), 1.23 (d, *J* = 5.2 Hz, 14H, -$NCH_2CH_2\underline{(CH_2)_7}CH_3$), 0.86 (t, *J* = 6.4 Hz, 3H, -CH_3); ^{13}C NMR (126 MHz, $CDCl_3$) δ 145.0, 134.3 (q, ^2J = 33.0 Hz, -CF_3), 127.5, 126.4 (d, ^3J = 3.5 Hz, -CF_3), δ 123.3 (q, ^1J = 272.6 Hz, -CF_3), 58.6, 52.0, 51.1, 32.9, 32.0, 29.6, 29.6, 29.4, 27.6, 26.9, 22.8, 14.2; ^{19}F NMR (471 MHz, $CDCl_3$) δ -62.9; HRMS (ESI) $[M+H]^+$ calcd for $C_{22}H_{36}O_2N_2F_3S$: 449.2444, found: 449.2439.

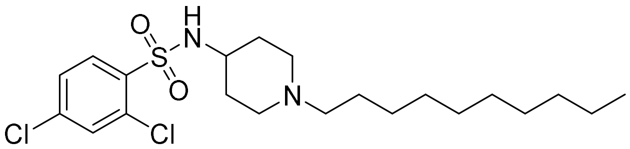

2,4-dichloro-*N*-(1-decylpiperidin-4-yl)benzenesulfonamide (A$_{13}$). A yellow solid, yield 38.6%, m. p. 87.2-88.3 °C; ^{1}H NMR (400 MHz, $CDCl_3$) δ 8.04 (d, *J* = 8.5 Hz, 1H, ph-3H), 7.53 (d, *J* = 2.0 Hz, 1H, ph-6H), 7.39 (dd, *J* = 8.5, 2.0 Hz, 1H, ph-5H), 5.01 (d, *J* = 2.9 Hz, 1H, -$NHSO_2$-), 3.24 – 3.11 (m, 1H, piperidin-CH), 2.73 (d, *J* = 11.5 Hz, 2H, piperidin-CH_2), 2.29 – 2.21 (m, 2H, -NCH_2-), 1.98 (t, *J* = 10.3 Hz, 2H, piperidin-CH_2), 1.75 (dd, *J* = 12.9, 3.7 Hz, 2H, piperidin-CH_2), 1.51 (ddd, *J* = 13.9, 10.6, 3.7 Hz, 2H, piperidin-CH_2), 1.45 – 1.36 (m, 2H, -$NCH_2\underline{CH_2}$-), 1.29 – 1.20 (m, 14H, -$NCH_2CH_2\underline{(CH_2)_7}CH_3$), 0.86 (t, *J* = 6.9 Hz, 3H, -CH_3); ^{13}C NMR (101 MHz,

CDCl3) δ 139.4, 137.2, 132.3, 131.8, 131.5, 127.6, 58.6, 51.8, 51.2, 32.7, 31.9, 29.6, 29.5, 29.3, 27.6, 27.0, 22.7, 14.1; HRMS (ESI) $[M+H]^+$ calcd for $C_{21}H_{35}O_2N_2Cl_2S$: 449.1791, found: 449.1786.

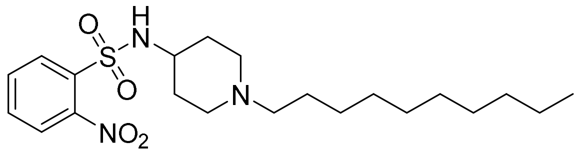

***N*-(1-decylpiperidin-4-yl)-2-nitrobenzenesulfonamide (A14)**. A yellow solid, yield 30.5%, m. p. 97.6-98.2 °C; ^1H NMR (500 MHz, $CDCl_3$) δ 8.19 – 8.13 (m, 1H, ph-3H), 7.89 – 7.83 (m, 1H, ph-6H), 7.77 – 7.71 (m, 2H, ph-4,5H), 3.41 – 3.36 (m, 1H, piperidin-CH), 2.74 (d, *J* = 10.0 Hz, 2H, piperidin-CH_2), 2.30 – 2.23 (m, 2H, -NCH_2-), 2.04 (s, 2H, piperidin-CH_2), 1.81 (dd, *J* = 13.0, 3.6 Hz, 2H, piperidin-CH_2), 1.56 (qd, *J* = 10.4, 3.7 Hz, 2H, piperidin-CH_2), 1.43 (dd, *J* = 14.8, 7.0 Hz, 2H, -$NCH_2\underline{CH_2}$-), 1.28 – 1.21 (m, 14H, -$NCH_2CH_2\underline{(CH_2)_7}CH_3$), 0.86 (t, *J* = 7.0 Hz, 3H, -CH_3); ^{13}C NMR (126 MHz, $CDCl_3$) δ 148.0, 135.2, 133.6, 133.0, 130.7, 125.6, 58.7, 51.8, 51.8, 32.8, 32.0, 29.7, 29.6, 29.4, 27.6, 27.1, 22.8, 14.2; HRMS (ESI) $[M+H]^+$ calcd for $C_{21}H_{36}O_4N_3S$: 426.2421, found: 426.2415.

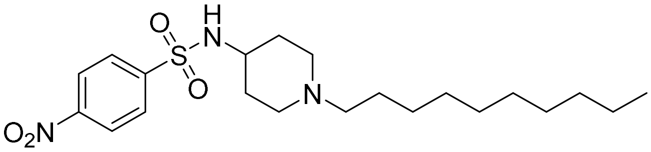

***N*-(1-decylpiperidin-4-yl)-4-nitrobenzenesulfonamide (A15).** A yellow solid, yield 45.2%, m. p. 88.9-89.6 °C; ^1H NMR (500 MHz, $CDCl_3$) δ 8.37 – 8.34 (m, 2H, ph-3,5H), 8.10 – 8.06 (m, 2H, ph-2,6H), 3.27 – 3.19 (m, 1H, piperidin-CH), 2.78 (d, *J* = 10.7 Hz, 2H, piperidin-CH_2), 2.27 (dd, *J* = 8.9, 6.7 Hz, 2H, -NCH_2-), 1.99 (t, *J* = 10.6 Hz, 2H, piperidin-CH_2), 1.81 – 1.73 (m, 2H, piperidin-CH_2), 1.57 – 1.48 (m, 2H,

piperidin-CH_2), 1.42 (dd, *J* = 14.6, 7.4 Hz, 2H, -$NCH_2\underline{CH_2}$-), 1.28 – 1.21 (m, 14H, -$NCH_2CH_2\underline{(CH_2)_7}CH_3$), 0.86 (t, *J* = 7.0 Hz, 3H, -CH_3); ^{13}C NMR (126 MHz, $CDCl_3$) δ 150.0, 147.4, 128.2, 124.5, 58.6, 52.0, 51.4, 33.0, 32.0, 29.6, 29.6, 29.4, 27.6, 27.0, 22.8, 14.2; HRMS (ESI) $[M+H]^+$ calcd for $C_{21}H_{36}O_4N_3S$: 426.2421, found: 426.2415.

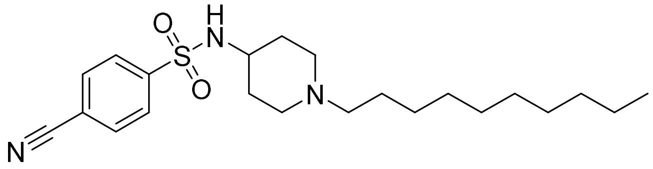

4-cyano-*N*-(1-decylpiperidin-4-yl)benzenesulfonamide (A$_{16}$). A yellow solid, yield 64.8%, m. p. 80.1-81.5 °C; ^1H NMR (400 MHz, $CDCl_3$) δ 8.05 – 8.00 (m, 2H, ph-2,6H), 7.85 – 7.79 (m, 2H, ph-3,5H), 3.29 – 3.16 (m, 1H, piperidin-CH), 2.86 (d, *J* = 11.6 Hz, 2H, piperidin-CH_2), 2.34 (dd, *J* = 8.9, 6.8 Hz, 2H, -NCH_2-), 2.07 (t, *J* = 10.9 Hz, 2H, piperidin-CH_2), 1.79 (d, *J* = 10.3 Hz, 2H, piperidin-CH_2), 1.67 – 1.54 (m, 2H, piperidin-CH_2), 1.51 – 1.39 (m, 2H, -$NCH_2\underline{CH_2}$-), 1.31 – 1.21 (m, 14H, -$NCH_2CH_2\underline{(CH_2)_7}CH_3$), 0.87 (t, *J* = 6.9 Hz, 3H, -CH_3); ^{13}C NMR (101 MHz, $CDCl_3$) δ 145.7, 133.0, 127.5, 117.4, 116.3, 58.4, 51.9, 50.9, 32.6, 31.9, 29.6, 29.5, 29.3, 27.5, 26.6, 22.7, 14.1; HRMS (ESI) $[M+H]^+$ calcd for $C_{22}H_{36}O_2N_3S$: 406.2523, found: 406.2516.

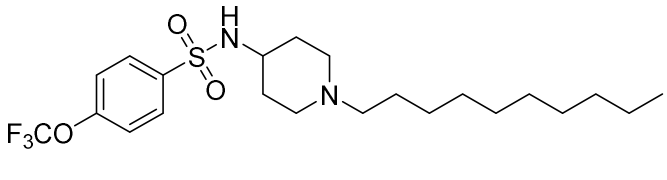

***N*-(1-decylpiperidin-4-yl)-4-(trifluoromethoxy)benzenesulfonamide (A$_{17}$)**. A yellow solid, yield 51.7%, m. p. 83.4-84.6 °C; ^1H NMR (400 MHz, $CDCl_3$) δ 7.96 – 7.91 (m, 2H, ph-2,6H), 7.32 (d, *J* = 8.2 Hz, 2H, ph-3,5H), 3.20 (dt, *J* = 10.2, 4.8 Hz, 1H, piperidin-CH), 2.80 (d, *J* = 11.0 Hz, 2H, piperidin-CH_2), 2.34 – 2.24 (m, 2H, -

NCH_2-), 2.03 (t, *J* = 10.0 Hz, 2H, piperidin-CH_2), 1.78 (d, *J* = 10.4 Hz, 2H, piperidin-CH_2), 1.55 (td, *J* = 13.8, 3.5 Hz, 2H, piperidin-CH_2), 1.44 (t, *J* = 10.1 Hz, 2H, -$NCH_2\underline{CH_2}$-), 1.30 – 1.20 (m, 14H, -$NCH_2CH_2\underline{(CH_2)_7}CH_3$), 0.86 (t, *J* = 6.8 Hz, 3H, -CH_3); ^{13}C NMR (101 MHz, $CDCl_3$) δ 152.0, 139.7, 129.0, 121.0, 120.2 (q, ^1J = 259.2 Hz, -OCF_3), 58.5, 51.9, 50.8, 32.7, 31.9, 29.6, 29.5, 29.3, 27.5, 26.8, 22.7, 14.1; ^{19}F NMR (376 MHz, $CDCl_3$) δ -57.7; HRMS (ESI) $[M+H]^+$ calcd for $C_{22}H_{36}O_3N_2F_3S$: 465.2393, found: 465.2387.

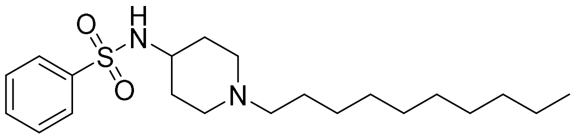

***N*-(1-decylpiperidin-4-yl)benzenesulfonamide (A_{18})**. A yellow oil, yield 68.4%; ^1H NMR (500 MHz, $CDCl_3$) δ 7.93 – 7.83 (m, 2H, ph-2,6H), 7.56 (t, *J* = 7.4 Hz, 1H, ph-4H), 7.53 – 7.46 (m, 2H, ph-3,5H), 5.11 (d, *J* = 1.2 Hz, 1H, -$NHSO_2$-), 3.18 (s, 1H, piperidin-CH), 2.77 (s, 2H, piperidin-CH_2), 2.36 – 2.22 (m, 2H, -NCH_2-), 2.05 (d, *J* = 0.5 Hz, 2H, piperidin-CH_2), 1.76 (d, *J* = 11.1 Hz, 2H, piperidin-CH_2), 1.52 (td, *J* = 12.9, 3.0 Hz, 2H, piperidin-CH_2), 1.43 (t, *J* = 8.9 Hz, 2H, -$NCH_2\underline{CH_2}$-), 1.26 – 1.19 (m, 14H, -$NCH_2CH_2\underline{(CH_2)_7}CH_3$), 0.85 (t, *J* = 7.0 Hz, 3H, -CH_3); ^{13}C NMR (126 MHz, $CDCl_3$) δ 141.2, 132.7, 129.2, 126.9, 58.5, 51.9, 50.5, 32.5, 32.0, 29.6, 29.6, 29.4, 27.6, 26.7, 22.8, 14.2; HRMS (ESI) $[M+H]^+$ calcd for $C_{21}H_{37}O_2N_2S$: 381.2570, found: 381.2564.

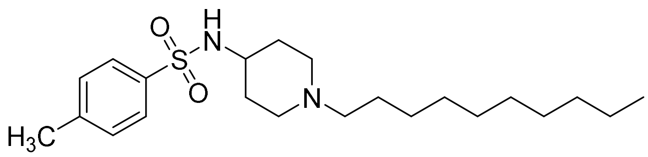

***N*-(1-decylpiperidin-4-yl)-4-methylbenzenesulfonamide (A_{19})**. A white solid, yield 63.4%, m. p. 88.0-88.9 °C; ^1H NMR (500 MHz, $CDCl_3$) δ 7.78 – 7.74 (m, 2H, ph-

2,6H), 7.28 (d, *J* = 8.0 Hz, 2H, ph-3,5H), 4.75 (d, *J* = 1.8 Hz, 1H, -NHSO$_2$-), 3.15 (d, *J* = 1.4 Hz, 1H, piperidin-CH), 2.72 (d, *J* = 11.2 Hz, 2H, piperidin-CH$_2$), 2.42 (s, 3H, ph-CH$_3$), 2.24 (dd, *J* = 8.9, 6.7 Hz, 2H, -NCH$_2$-), 1.98 (t, *J* = 9.9 Hz, 2H, piperidin-CH$_2$), 1.79 – 1.71 (m, 2H, piperidin-CH$_2$), 1.47 (ddd, *J* = 14.2, 10.8, 3.9 Hz, 2H, piperidin-CH$_2$), 1.41 (dd, *J* = 10.2, 4.4 Hz, 2H, -NCH$_2$$\underline{CH_2}$-), 1.27 – 1.19 (m, 14H, -NCH$_2$CH$_2$$\underline{(CH_2)_7}CH_3$), 0.86 (t, *J* = 7.0 Hz, 3H, -CH$_2$$\underline{CH_3}$); ^{13}C NMR (126 MHz, CDCl$_3$) δ 143.4, 138.4, 129.8, 127.0, 58.7, 52.0, 50.8, 33.0, 32.0, 29.6, 29.6, 29.4, 27.6, 27.0, 22.7, 21.6, 14.2; HRMS (ESI) [M+H]$^+$ calcd for $C_{22}H_{39}O_2N_2S$: 395.2727, found: 395.2722.

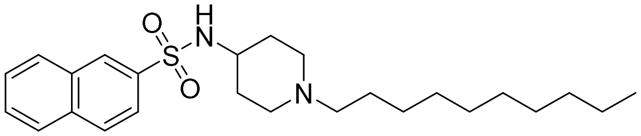

***N*-(1-decylpiperidin-4-yl)naphthalene-2-sulfonamide (A$_{20}$)**. A yellow solid, yield 72.8%, m. p. 59.9-60.8 °C; ^{1}H NMR (400 MHz, CDCl$_3$) δ 8.46 (d, *J* = 1.4 Hz, 1H, naphthyl-1H), 7.99 – 7.93 (m, 2H, naphthyl-5,8H), 7.91 (d, *J* = 7.8 Hz, 1H, naphthyl-4H), 7.85 (dd, *J* = 8.7, 1.9 Hz, 1H, naphthyl-3H), 7.63 (pd, *J* = 6.9, 1.4 Hz, 2H, naphthyl-6,7H), 4.85 (s, 1H, -NHSO$_2$-), 3.23 (dd, *J* = 11.8, 8.0 Hz, 1H, piperidin-CH), 2.73 (d, *J* = 11.7 Hz, 2H, piperidin-CH$_2$), 2.28 – 2.21 (m, 2H, -NCH$_2$-), 1.98 (t, *J* = 10.2 Hz, 2H, piperidin-CH$_2$), 1.77 (dd, *J* = 13.3, 3.0 Hz, 2H, piperidin-CH$_2$), 1.51 (qd, *J* = 10.7, 3.7 Hz, 2H, piperidin-CH$_2$), 1.40 (dd, *J* = 12.1, 4.7 Hz, 2H, -NCH$_2$$\underline{CH_2}$-), 1.28 – 1.19 (m, 14H, -NCH$_2$CH$_2$$\underline{(CH_2)_7}CH_3$), 0.86 (t, *J* = 6.8 Hz, 3H, -CH$_3$); ^{13}C NMR (101 MHz, CDCl$_3$) δ 138.0, 134.8, 132.2, 129.6, 129.3, 128.8, 128.2, 127.9, 127.6, 122.3, 58.6, 51.9, 50.8, 32.9, 31.9, 29.6, 29.5, 29.3, 27.5, 26.9, 22.7, 14.1; HRMS (ESI)

[M+H]$^+$ calcd for $C_{25}H_{39}O_2N_2S$: 431.2727, found: 431.2722.

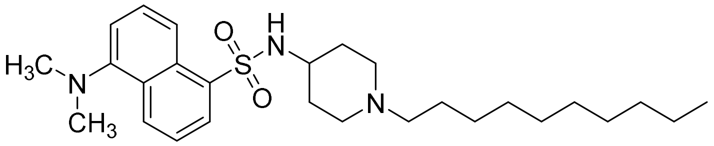

***N*-(1-decylpiperidin-4-yl)-5-(dimethylamino)naphthalene-1-sulfonamide (A$_{21}$)**. A green oil, yield 66.7%; ^{1}H NMR (500 MHz, $CDCl_3$) δ 8.56 – 8.50 (m, 1H, naphthyl-4H), 8.29 – 8.21 (m, 2H, naphthyl-2,8H), 7.53 (ddd, *J* = 18.1, 8.5, 7.5 Hz, 2H, naphthyl-3,7H), 7.17 (d, *J* = 7.3 Hz, 1H, naphthyl-6H), 4.74 (d, *J* = 4.5 Hz, 1H, -$NHSO_2$-), 3.15 (d, *J* = 4.5 Hz, 1H, piperidin-CH), 2.88 (s, 6H, -$N(CH_3)_2$), 2.64 (d, *J* = 10.3 Hz, 2H, piperidin-CH_2), 2.19 (dd, *J* = 8.9, 6.7 Hz, 2H, -NCH_2-), 1.88 (d, *J* = 9.0 Hz, 2H, piperidin-CH_2), 1.64 (dd, *J* = 13.4, 2.9 Hz, 2H, piperidin-CH_2), 1.41 – 1.32 (m, 4H, piperidin-CH_2 and -$NCH_2\underline{CH_2}$-), 1.28 – 1.18 (m, 14H, -$NCH_2CH_2\underline{(CH_2)_7}CH_3$), 0.85 (t, *J* = 7.0 Hz, 3H, -$CH_2\underline{CH_3}$); ^{13}C NMR (126 MHz, $CDCl_3$) δ 152.1, 135.9, 130.5, 129.9, 129.6, 129.4, 128.4, 123.3, 118.8, 115.3, 58.7, 52.0, 51.0, 45.5, 32.9, 32.0, 29.6, 29.6, 29.4, 27.6, 27.1, 22.8, 14.2; HRMS (ESI) [M+H]$^+$ calcd for $C_{27}H_{44}O_2N_3S$: 474.3149, found: 474.3142.

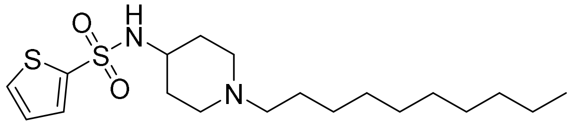

***N*-(1-decylpiperidin-4-yl)thiophene-2-sulfonamide (A$_{22}$)**. A yellow solid, yield 60.5%, m. p. 56.1-57.6 °C; ^{1}H NMR (500 MHz, $CDCl_3$) δ 7.63 (dd, *J* = 3.7, 1.3 Hz, 1H, thiophene-5H), 7.55 (dd, *J* = 5.0, 1.3 Hz, 1H, thiophene-3H), 7.05 (dd, *J* = 5.0, 3.8 Hz, 1H, thiophene-4H), 3.34 (s, 1H, piperidin-CH), 3.08 – 2.95 (m, 2H, piperidin-CH_2), 2.61 – 2.23 (m, 4H, -NCH_2- and piperidin-CH_2), 1.91 (s, 2H, piperidin-CH_2), 1.79 –

1.70 (m, 2H, piperidin-CH_2), 1.54 (s, 2H, -$NCH_2\underline{CH_2}$-), 1.25 – 1.19 (m, 14H, -$NCH_2CH_2\underline{(CH_2)_7}CH_3$), 0.85 (t, *J* = 7.0 Hz, 3H, -CH_3); ^{13}C NMR (126 MHz, $CDCl_3$) δ 142.2, 132.1, 131.8, 127.6, 58.1, 51.2, 49.8, 32.0, 31.2, 29.6, 29.6, 29.4, 29.4, 27.3, 25.8, 22.8, 14.2; HRMS (ESI) $[M+H]^+$ calcd for $C_{19}H_{35}O_2N_2S_2$: 387.2134, found: 387.2127.

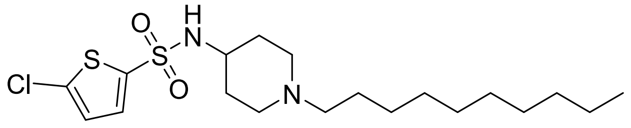

5-chloro-*N*-(1-decylpiperidin-4-yl)thiophene-2-sulfonamide (A23). A yellow solid, yield 34.2%, m. p. 78.6-80.1 °C; ^1H NMR (500 MHz, $CDCl_3$) δ 7.42 (d, *J* = 2.9 Hz, 1H, thiophene-3H), 6.90 (d, *J* = 3.5 Hz, 1H, thiophene-4H), 3.30 (d, *J* = 1.1 Hz, 1H, piperidin-CH), 2.98 (d, *J* = 3.4 Hz, 2H, piperidin-CH_2), 2.45 (s, 2H, -NCH_2-), 2.25 (dd, *J* = 24.4, 10.1 Hz, 2H, piperidin-CH_2), 1.92 (d, *J* = 1.3 Hz, 2H, piperidin-CH_2), 1.70 (d, *J* = 21.2 Hz, 2H, piperidin-CH_2), 1.52 (s, 2H, -$NCH_2\underline{CH_2}$-), 1.24 (d, *J* = 4.1 Hz, 14H, -$NCH_2CH_2\underline{(CH_2)_7}CH_3$), 0.86 (t, *J* = 7.0 Hz, 3H, -CH_3); ^{13}C NMR (126 MHz, $CDCl_3$) 140.3, 137.1, 131.5, 126.9, 58.4, 51.7, 50.5, 32.0, 32.0, 29.8, 29.6, 29.5, 29.4, 27.4, 26.2, 22.8, 14.2; HRMS (ESI) $[M+H]^+$ calcd for $C_{19}H_{34}O_2N_2ClS_2$: 421.1745, found: 421.1740.

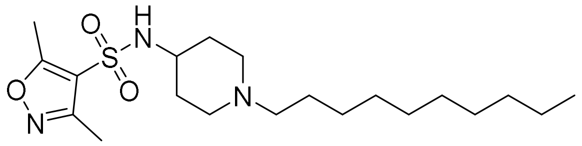

***N*-(1-decylpiperidin-4-yl)-3,5-dimethylisoxazole-4-sulfonamide (A24)**. A yellow solid, yield 36.6%, m. p. 72.7-73.8 °C; ^1H NMR (500 MHz, $CDCl_3$) δ 3.18 – 3.05 (m, 1H, piperidin-CH), 2.80 (d, *J* = 9.2 Hz, 2H, piperidin-CH_2), 2.63 (s, 3H, isoxazole-3-CH_3), 2.40 (s, 3H, isoxazole-5-CH_3), 2.27 (dd, *J* = 8.9, 6.7 Hz, 2H, -NCH_2-),

2.00 (t, *J* = 9.9 Hz, 2H, piperidin-CH_2), 1.86 – 1.79 (m, 2H, piperidin-CH_2), 1.53 (qd, *J* = 11.2, 3.7 Hz, 2H, piperidin-CH_2), 1.43 (dd, *J* = 14.3, 7.0 Hz, 2H, $-NCH_2\underline{CH_2}-$), 1.28 – 1.22 (m, 14H, $-NCH_2CH_2\underline{(CH_2)_7}CH_3$), 0.86 (t, *J* = 7.0 Hz, 3H, $-CH_3$); ^{13}C NMR (126 MHz, $CDCl_3$) δ 172.9, 157.6, 117.1, 58.7, 52.1, 51.0, 33.1, 32.0, 29.7, 29.6, 29.4, 27.6, 27.1, 22.8, 14.2, 12.8, 10.9; HRMS (ESI) $[M+H]^+$ calcd for $C_{20}H_{38}O_3N_3S$: 400.2628, found: 400.2622.

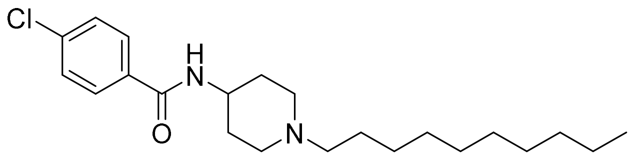

4-chloro-*N*-(1-decylpiperidin-4-yl)benzamide (B_1). A white solid, yield 71.6%, m. p. 149.1-149.9 °C; ^1H NMR (500 MHz, $CDCl_3$) δ 7.71 – 7.66 (m, 2H, ph-2,6H), 7.40 – 7.35 (m, 2H, ph-3,5H), 6.07 (s, 1H, -NHCO-), 4.03 – 3.91 (m, 1H, piperidin-CH), 2.90 (d, *J* = 11.6 Hz, 2H, piperidin-CH_2), 2.33 (dd, *J* = 8.9, 6.7 Hz, 2H, $-NCH_2-$), 2.11 (t, *J* = 11.1 Hz, 2H, piperidin-CH_2), 2.01 (dd, *J* = 8.3, 3.6 Hz, 2H, piperidin-CH_2), 1.58 (qd, *J* = 11.9, 3.7 Hz, 2H, piperidin-CH_2), 1.49 (dd, *J* = 14.5, 7.3 Hz, 2H, $-NCH_2\underline{CH_2}-$), 1.27 (dd, *J* = 14.9, 10.2 Hz, 14H, $-NCH_2CH_2\underline{(CH_2)_7}CH_3$), 0.87 (t, *J* = 7.0 Hz, 3H, $-CH_3$); ^{13}C NMR (126 MHz, $CDCl_3$) δ 165.9, 137.7, 133.2, 128.8, 128.4, 58.9, 52.5, 47.3, 32.3, 32.0, 29.7, 29.4, 27.7, 27.2, 22.8, 14.2; HRMS (ESI) $[M+H]^+$ calcd for $C_{22}H_{36}ON_2Cl$: 379.2511, found: 379.2506.

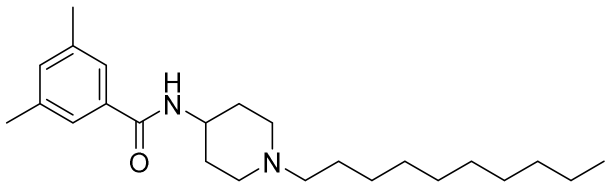

***N*-(1-decylpiperidin-4-yl)-3,5-dimethylbenzamide (B_2)**. A white solid, yield 68.0%, m. p. 125.9-127.1 °C; ^1H NMR (500 MHz, $CDCl_3$) δ 7.33 (s, 2H, ph-2,6H), 7.11

(s, 1H, ph-4H), 5.96 (d, *J* = 7.9 Hz, 1H, -NHCO-), 4.05 – 3.93 (m, 1H, piperidin-CH), 2.91 (d, *J* = 10.9 Hz, 2H, piperidin-CH_2), 2.38 – 2.30 (m, 8H, -NCH_2- and ph-3,5-CH_3), 2.14 (t, *J* = 11.1 Hz, 2H, piperidin-CH_2), 2.06 – 1.99 (m, 2H, piperidin-CH_2), 1.58 (qd, *J* = 12.3, 3.6 Hz, 2H, piperidin-CH_2), 1.48 (d, *J* = 7.0 Hz, 2H, -$NCH_2\underline{CH_2}$-), 1.31 – 1.23 (m, 14H, -$NCH_2CH_2\underline{(CH_2)_7}CH_3$), 0.87 (t, *J* = 6.9 Hz, 3H, -$CH_2\underline{CH_3}$); ^{13}C NMR (126 MHz, $CDCl_3$) δ 167.4, 138.3, 134.9, 133.0, 124.7, 58.9, 52.6, 47.0, 32.3, 32.0, 29.7, 29.4, 27.7, 27.2, 22.8, 21.3, 14.2; HRMS (ESI) $[M+H]^+$ calcd for $C_{24}H_{41}ON_2$: 373.3213, found: 373.3210.

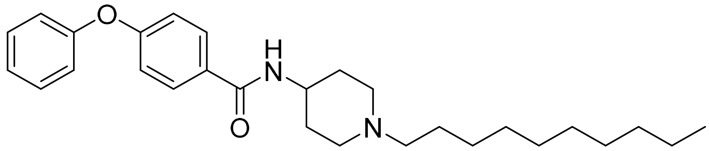

***N*-(1-decylpiperidin-4-yl)-4-phenoxybenzamide (B$_3$)**. A white solid, yield 67.7%, m. p. 127.5-128.3 °C; ^{1}H NMR (400 MHz, $CDCl_3$) δ 7.78 – 7.67 (m, 2H, -CO-ph-2,6H), 7.42 – 7.32 (m, 2H, -O-ph-3,5H), 7.16 (t, *J* = 7.4 Hz, 1H, -O-ph-4H), 7.03 (dd, *J* = 8.5, 0.8 Hz, 2H, -O-ph-2,6H), 7.01 – 6.96 (m, 2H, -CO-ph-3,5H), 6.04 (d, *J* = 8.0 Hz, 1H, -NHCO-), 4.19 – 3.86 (m, 1H, piperidin-CH), 2.92 (d, *J* = 11.3 Hz, 2H, piperidin-CH_2), 2.43 – 2.27 (m, 2H, -NCH_2-), 2.14 (t, *J* = 11.2 Hz, 2H, piperidin-CH_2), 2.03 (d, *J* = 10.3 Hz, 2H, piperidin-CH_2), 1.60 (qd, *J* = 12.3, 3.6 Hz, 2H, piperidin-CH_2), 1.48 (d, *J* = 6.7 Hz, 2H, -$NCH_2\underline{CH_2}$-), 1.26 (d, *J* = 8.2 Hz, 14H, -$NCH_2CH_2\underline{(CH_2)_7}CH_3$), 0.87 (t, *J* = 6.8 Hz, 3H, -CH_3); ^{13}C NMR (101 MHz, $CDCl_3$) δ 166.2, 160.4, 156.0, 130.0, 129.1, 128.8, 124.2, 119.7, 117.9, 58.8, 52.5, 47.0, 32.2, 31.9, 29.6, 29.4, 27.7, 27.1, 22.7, 14.2; HRMS (ESI) $[M+H]^+$ calcd for $C_{28}H_{41}O_2N_2$: 437.3163, found: 437.3158.

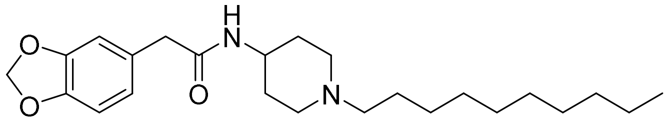

2-(benzo[*d*][1,3]dioxol-5-yl)-*N*-(1-decylpiperidin-4-yl)acetamide (B_4). A white solid, yield 42.3%, m. p. 148.0-148.9 °C; ^{1}H NMR (500 MHz, $CDCl_3$) δ 6.75 (d, J = 7.9 Hz, 1H, benzodioxole-7H), 6.70 (d, J = 1.6 Hz, 1H, benzodioxole-4H), 6.66 (dd, J = 7.9, 1.7 Hz, 1H, benzodioxole-6H), 5.94 (s, 2H, benzodioxole-CH_2), 5.40 (d, J = 8.0 Hz, 1H, -NHCO-), 3.81 – 3.70 (m, 1H, piperidin-CH), 3.42 (s, 2H, -$COCH_2$-), 2.78 (d, J = 7.2 Hz, 2H, piperidin-CH_2), 2.27 (dd, J = 9.0, 6.7 Hz, 2H, -NCH_2-), 2.03 (t, J = 11.1 Hz, 2H, piperidin-CH_2), 1.89 – 1.81 (m, 2H, piperidin-CH_2), 1.48 – 1.40 (m, 2H, -$NCH_2\underline{CH_2}$-), 1.36 (ddd, J = 14.8, 12.6, 3.8 Hz, 2H, piperidin-CH_2), 1.27 – 1.19 (m, 14H, -$NCH_2CH_2\underline{(CH_2)_7}CH_3$), 0.85 (t, J = 7.0 Hz, 3H, -CH_3); ^{13}C NMR (126 MHz, $CDCl_3$) δ 170.6, 148.1, 146.9, 128.6, 122.6, 109.7, 108.7, 101.2, 58.8, 52.4, 46.5, 43.6, 32.0, 29.7, 29.6, 29.4, 27.7, 27.1, 22.8, 14.2; HRMS (ESI) $[M+H]^+$ calcd for $C_{24}H_{39}O_3N_2$: 403.2955, found: 403.2951.

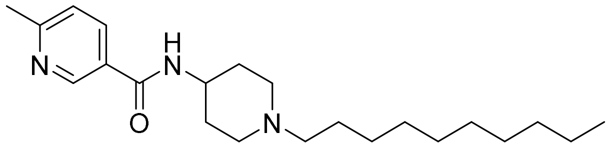

***N*-(1-decylpiperidin-4-yl)-6-methylnicotinamide (B_5)**. A yellow solid, yield 31.3%, m. p. 106.4-107.8 °C; ^{1}H NMR (400 MHz, $CDCl_3$) δ 8.82 (d, J = 2.0 Hz, 1H, pyridine-2H), 7.96 (dd, J = 8.1, 2.3 Hz, 1H, pyridine-4H), 7.18 (d, J = 8.1 Hz, 1H, pyridine-5H), 6.36 (d, J = 7.8 Hz, 1H, -NHCO-), 4.05 – 3.92 (m, 1H, piperidin-CH), 2.92 (d, J = 11.0 Hz, 2H, piperidin-CH_2), 2.56 (s, 3H, pyridine-CH_3), 2.33 (dd, J = 9.0, 6.8 Hz, 2H, -NCH_2-), 2.12 (t, J = 11.4 Hz, 2H, piperidin-CH_2), 2.01 (d, J = 10.7 Hz, 2H, piperidin-CH_2), 1.62 (qd, J = 12.2, 3.6 Hz, 2H, piperidin-CH_2), 1.46 (d, J = 6.6 Hz, 2H,

-NCH$_2\underline{CH_2}$-), 1.24 (d, *J* = 8.1 Hz, 14H, -NCH$_2$CH$_2\underline{(CH_2)_7}$CH$_3$), 0.85 (t, *J* = 6.8 Hz, 3H, -CH$_2\underline{CH_3}$); HRMS (ESI) $[M+H]^+$ calcd for $C_{22}H_{38}ON_3$: 360.3009, found: 360.3008.

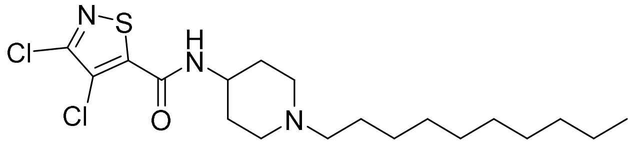

3,4-dichloro-*N*-(1-decylpiperidin-4-yl)isothiazole-5-carboxamide (B$_6$). A white solid, yield 67.3%, m. p. 111.6-112.9 °C; ^{1}H NMR (400 MHz, CDCl$_3$) δ 6.74 (d, *J* = 7.6 Hz, 1H, -NHCO-), 4.07 – 3.94 (m, 1H, piperidin-CH), 2.85 (d, *J* = 10.8 Hz, 2H, piperidin-CH$_2$), 2.33 (dd, *J* = 8.9, 6.7 Hz, 2H, -NCH$_2$-), 2.17 (t, *J* = 10.7 Hz, 2H, piperidin-CH$_2$), 2.09 – 1.99 (m, 2H, piperidin-CH$_2$), 1.63 (qd, *J* = 10.8, 3.7 Hz, 2H, piperidin-CH$_2$), 1.53 – 1.42 (m, 2H, -NCH$_2\underline{CH_2}$-), 1.26 (d, *J* = 9.5 Hz, 14H, -NCH$_2$CH$_2\underline{(CH_2)_7}$CH$_3$), 0.86 (t, *J* = 6.8 Hz, 3H, -CH$_3$); ^{13}C NMR (101 MHz, CDCl$_3$) δ 157.2, 156.7, 149.0, 118.3, 58.8, 52.0, 47.2, 31.9, 31.8, 29.6, 29.4, 27.6, 27.1, 22.7, 14.2; HRMS (ESI) $[M+H]^+$ calcd for $C_{19}H_{32}ON_3Cl_2S$: 420.1638, found: 420.1633.

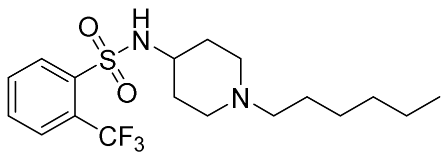

***N*-(1-hexylpiperidin-4-yl)-2-(trifluoromethyl)benzenesulfonamide (C$_1$).** A yellow solid, yield 74.4%, m. p. 83.9-84.7 °C; ^{1}H NMR (400 MHz, CDCl$_3$) δ 8.29 – 8.20 (m, 1H, ph-3H), 7.90 – 7.82 (m, 1H, ph-6H), 7.75 – 7.64 (m, 2H, ph-4,5H), 4.75 (d, *J* = 4.7 Hz, 1H, -NHSO$_2$-), 3.30 – 3.16 (m, 1H, piperidin-CH), 2.71 (d, *J* = 11.2 Hz, 2H, piperidin-CH$_2$), 2.23 (dd, *J* = 8.9, 6.7 Hz, 2H, -NCH$_2$-), 1.96 (t, *J* = 10.6 Hz, 2H, piperidin-CH$_2$), 1.76 – 1.66 (m, 2H, piperidin-CH$_2$), 1.52 – 1.34 (m, 4H, piperidin-CH$_2$ and -NCH$_2\underline{CH_2}$-), 1.29 – 1.18 (m, 6H, -NCH$_2$CH$_2\underline{(CH_2)_3}$CH$_3$), 0.84 (t, *J* = 6.9 Hz, 3H,

-CH_3); ^{13}C NMR (101 MHz, $CDCl_3$) δ 140.0, 132.7, 132.5, 131.3, 128.6 (q, ^3J = 6.1 Hz, -CF_3), 127.3 (q, ^2J = 32.8 Hz, -CF_3), 123.0 (q, ^1J = 273.9 Hz, -CF_3) 58.6, 51.9, 51.3, 32.8, 31.8, 27.2, 27.0, 22.6, 14.1; ^{19}F NMR (376 MHz, $CDCl_3$) δ -57.8; HRMS (ESI) $[M+H]^+$ calcd for $C_{18}H_{28}O_2N_2F_3S$: 393.1818, found: 393.1813.

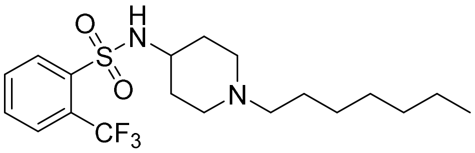

***N*-(1-heptylpiperidin-4-yl)-2-(trifluoromethyl)benzenesulfonamide (C2)**. A yellow solid, yield 45.6%, m. p. 85.2-86.5 °C; ^{1}H NMR (400 MHz, $CDCl_3$) δ 8.25 (ddd, J = 5.5, 4.8, 2.2 Hz, 1H, ph-3H), 7.90 – 7.83 (m, 1H, ph-6H), 7.74 – 7.65 (m, 2H, ph-4,5H), 4.75 (d, J = 6.1 Hz, 1H, -$NHSO_2$-), 3.30 – 3.15 (m, 1H, piperidin-CH), 2.72 (d, J = 11.0 Hz, 2H, piperidin-CH_2), 2.23 (dd, J = 8.9, 6.7 Hz, 2H, -NCH_2-), 1.96 (t, J = 10.3 Hz, 2H, piperidin-CH_2), 1.76 – 1.67 (m, 2H, piperidin-CH_2), 1.52 – 1.35 (m, 4H, piperidin-CH_2 and -$NCH_2\underline{CH_2}$-), 1.28 – 1.19 (m, 8H, -$NCH_2CH_2\underline{(CH_2)_4}CH_3$), 0.84 (t, J = 6.9 Hz, 3H, -CH_3); ^{13}C NMR (101 MHz, $CDCl_3$) δ 140.0, 132.7, 132.5, 131.3, 128.6 (q, ^3J = 6.2 Hz, -CF_3), 127.3 (q, ^2J = 32.8 Hz, -CF_3), 123.0 (q, ^1J = 274.0 Hz, -CF_3), 58.6, 51.9, 51.3, 32.9, 31.8, 29.2, 27.5, 27.0, 22.6, 14.1; ^{19}F NMR (376 MHz, $CDCl_3$) δ -57.8; HRMS (ESI) $[M+H]^+$ calcd for $C_{19}H_{30}O_2N_2F_3S$: 407.1975, found: 407.1968.

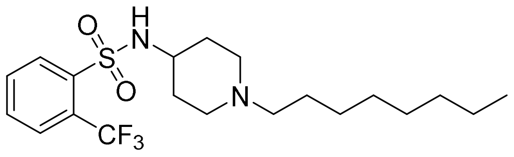

***N*-(1-octylpiperidin-4-yl)-2-(trifluoromethyl)benzenesulfonamide (C3)**. A yellow solid, yield 69.5%, m. p. 89.7-90.4 °C; ^{1}H NMR (500 MHz, $CDCl_3$) δ 8.27 – 8.21 (m, 1H, ph-3H), 7.89 – 7.83 (m, 1H, ph-6H), 7.74 – 7.65 (m, 2H, ph-4,5H), 3.33

– 3.22 (m, 1H, piperidin-CH), 2.83 (d, *J* = 10.9 Hz, 2H, piperidin-CH_2), 2.40 – 2.28 (m, 2H, -NCH_2-), 2.12 (d, *J* = 1.1 Hz, 2H, piperidin-CH_2), 1.79 (d, *J* = 11.1 Hz, 2H, piperidin-CH_2), 1.64 – 1.53 (m, 2H, piperidin-CH_2), 1.45 (d, *J* = 6.6 Hz, 2H, -$NCH_2\underline{CH_2}$-), 1.24 (t, *J* = 8.1 Hz, 10H, -$NCH_2CH_2\underline{(CH_2)_5}CH_3$), 0.84 (t, *J* = 7.0 Hz, 3H, -CH_3); ^{13}C NMR (126 MHz, $CDCl_3$) δ 139.9, 132.8, 132.6, 131.3, 128.7 (q, ^3J = 6.1 Hz, -CF_3), 127.4 (q, ^2J = 32.9 Hz, -CF_3), 123.1 (q, ^1J = 273.9 Hz, -CF_3), 58.4, 51.7, 50.8, 31.9, 29.8, 29.5, 29.3, 27.5, 26.5, 22.7, 14.2; ^{19}F NMR (471 MHz, $CDCl_3$) δ -57.7; HRMS (ESI) $[M+H]^+$ calcd for $C_{20}H_{32}O_2N_2F_3S$: 421.2131, found: 421.2126.

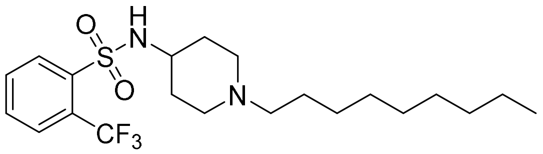

***N*-(1-nonylpiperidin-4-yl)-2-(trifluoromethyl)benzenesulfonamide (C4)**. A yellow solid, yield 55.4%, m. p. 89.4-90.3 °C; ^{1}H NMR (400 MHz, $CDCl_3$) δ 8.29 – 8.22 (m, 1H, ph-3H), 7.87 (ddd, *J* = 8.9, 4.4, 2.1 Hz, 1H, ph-6H), 7.76 – 7.66 (m, 2H, ph-4,5H), 4.74 (s, 1H, -$NHSO_2$-), 3.33 – 3.16 (m, 1H, piperidin-CH), 2.75 (d, *J* = 10.9 Hz, 2H, piperidin-CH_2), 2.30 – 2.23 (m, 2H, -NCH_2-), 2.00 (t, *J* = 9.5 Hz, 2H, piperidin-CH_2), 1.80 – 1.67 (m, 2H, piperidin-CH_2), 1.56 – 1.36 (m, 4H, piperidin-CH_2 and -$NCH_2\underline{CH_2}$-), 1.28 – 1.20 (m, 12H, -$NCH_2CH_2\underline{(CH_2)_6}CH_3$), 0.86 (t, *J* = 6.9 Hz, 3H, -CH_3); ^{13}C NMR (101 MHz, $CDCl_3$) δ 139.9, 132.7, 132.5, 131.3, 128.6 (q, ^3J = 6.1 Hz, -CF_3), 127.4 (q, ^2J = 32.9 Hz, -CF_3), 123.0 (q, ^1J = 273.9 Hz, -CF_3), 58.6, 51.8, 51.2, 32.7, 31.9, 29.5, 29.3, 27.5, 26.9, 22.7, 14.2; ^{19}F NMR (376 MHz, $CDCl_3$) δ -57.8; HRMS (ESI) $[M+H]^+$ calcd for $C_{21}H_{34}O_2N_2F_3S$: 435.2288, found: 435.2281.

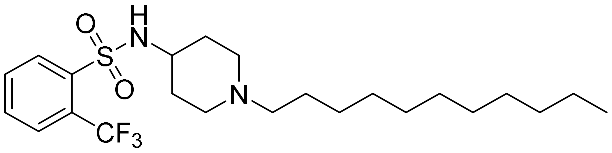

2-(trifluoromethyl)-*N*-(1-undecylpiperidin-4-yl)benzenesulfonamide (C_5). A yellow solid, yield 65.7%, m. p. 93.8-94.8 °C; ^{1}H NMR (400 MHz, $CDCl_3$) δ 8.28 – 8.21 (m, 1H, ph-3H), 7.90 – 7.84 (m, 1H, ph-6H), 7.75 – 7.66 (m, 2H, ph-4,5H), 4.74 (d, J = 6.0 Hz, 1H, -$NHSO_2$-), 3.31 – 3.16 (m, 1H, piperidin-CH), 2.73 (d, J = 11.2 Hz, 2H, piperidin-CH_2), 2.29 – 2.20 (m, 2H, -NCH_2-), 1.97 (t, J = 10.3 Hz, 2H, piperidin-CH_2), 1.72 (dd, J = 13.3, 3.0 Hz, 2H, piperidin-CH_2), 1.54 – 1.34 (m, 4H, piperidin-CH_2 and -$NCH_2\underline{CH_2}$-), 1.29 – 1.18 (m, 16H, -$NCH_2CH_2\underline{(CH_2)_8}CH_3$), 0.86 (t, J = 6.9 Hz, 3H, -CH_3); ^{13}C NMR (101 MHz, $CDCl_3$) δ 134.0, 132.7, 132.5, 131.3, 128.6 (q, ^3J = 6.3 Hz, -CF_3), 127.4 (q, ^2J = 32.8 Hz, -CF_3), 123.0 (q, ^1J = 273.8 Hz, -CF_3), 58.6, 51.9, 51.2, 32.8, 31.9, 29.6, 29.6, 29.5, 29.4, 27.5, 27.0, 22.7, 14.2; ^{19}F NMR (376 MHz, $CDCl_3$) δ -57.8; HRMS (ESI) $[M+H]^+$ calcd for $C_{23}H_{38}O_2N_2F_3S$: 463.2601, found: 463.2593.

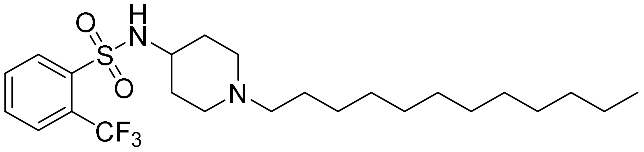

***N*-(1-dodecylpiperidin-4-yl)-2-(trifluoromethyl)benzenesulfonamide**. **(C_6)** A yellow solid, yield 69.4%, m. p. 92.7-93.5 °C; ^{1}H NMR (400 MHz, $CDCl_3$) δ 8.26 (ddd, J = 5.5, 4.9, 2.3 Hz, 1H, ph-3H), 7.90 – 7.83 (m, 1H, ph-6H), 7.74 – 7.66 (m, 2H, ph-4,5H), 4.73 (d, J = 11.0 Hz, 1H, -$NHSO_2$-), 3.31 – 3.15 (m, 1H, piperidin-CH), 2.72 (d, J = 11.1 Hz, 2H, piperidin-CH_2), 2.23 (dd, J = 8.8, 6.8 Hz, 2H, -NCH_2-), 1.96 (t, J = 10.5 Hz, 2H, piperidin-CH_2), 1.72 (dd, J = 13.4, 3.0 Hz, 2H, piperidin-CH_2), 1.53 –

1.34 (m, 4H, piperidin-CH_2 and -$NCH_2\underline{CH_2}$-), 1.30 – 1.18 (m, 18H, -$NCH_2CH_2\underline{(CH_2)_9}CH_3$), 0.86 (t, *J* = 6.9 Hz, 3H, -CH_3); ^{13}C NMR (101 MHz, $CDCl_3$) δ 134.0, 132.7, 132.5, 131.3, 128.6 (q, ^3J = 6.4 Hz, -CF_3), 127.4 (q, ^2J = 32.9 Hz, -CF_3), 123.0 (q, ^1J = 274.0 Hz, -CF_3), 58.6, 51.9, 51.3, 32.8, 31.9, 29.7, 29.7, 29.6, 29.6, 29.6, 29.4, 27.6, 27.0, 22.7, 14.2; ^{19}F NMR (376 MHz, $CDCl_3$) δ -57.8; HRMS (ESI) $[M+H]^+$ calcd for $C_{24}H_{40}O_2N_2F_3S$: 477.2757, found: 477.2751.

6. ^{1}H NMR, ^{13}C NMR, ^{19}F NMR and HRMS spectra analysis of A_1-A_{24}, B_1-B_6, and C_1-C_6

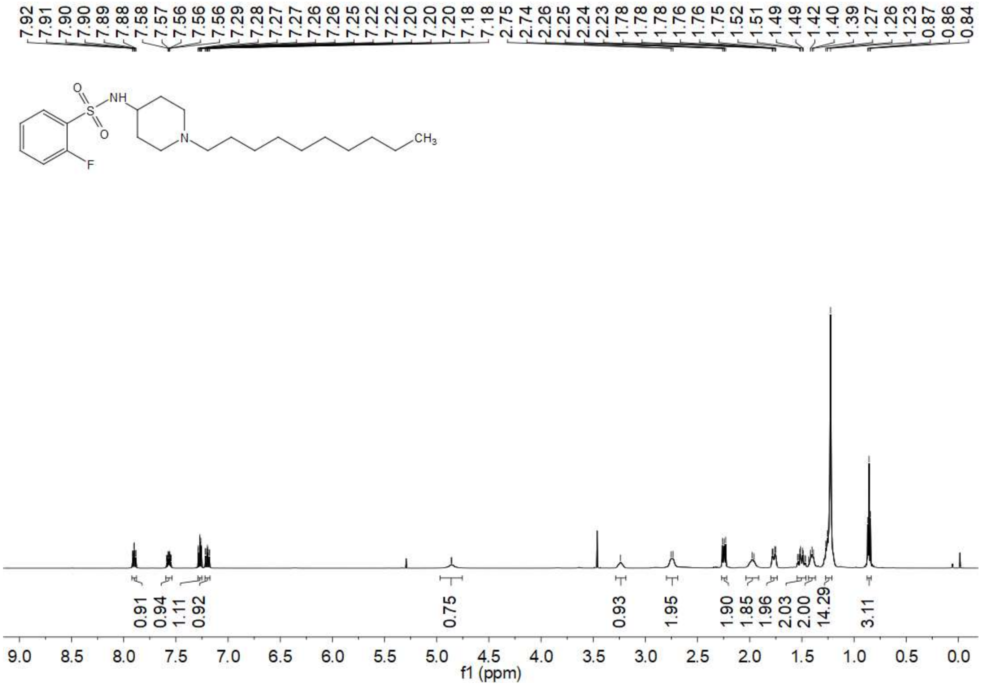

Figure S4. ^{1}H NMR spectrum ($CDCl_3$, 500 MHz) of $\mathbf{A_1}$.

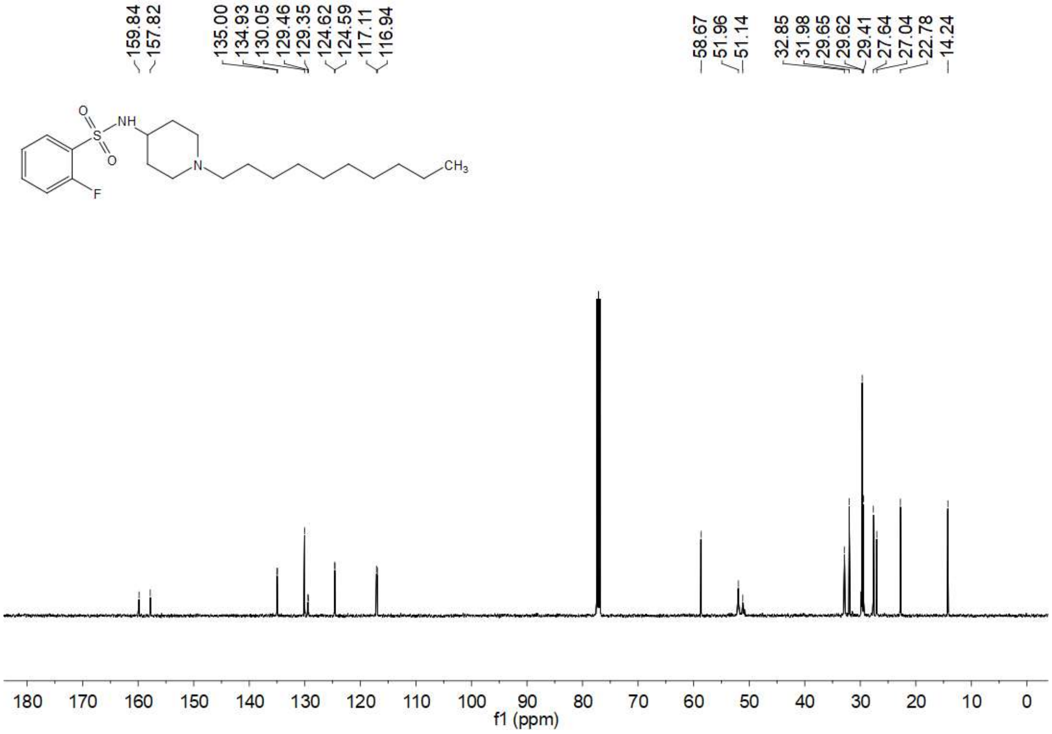

Figure S5. ^{13}C NMR spectrum ($CDCl_3$, 126 MHz) of $\mathbf{A_1}$.

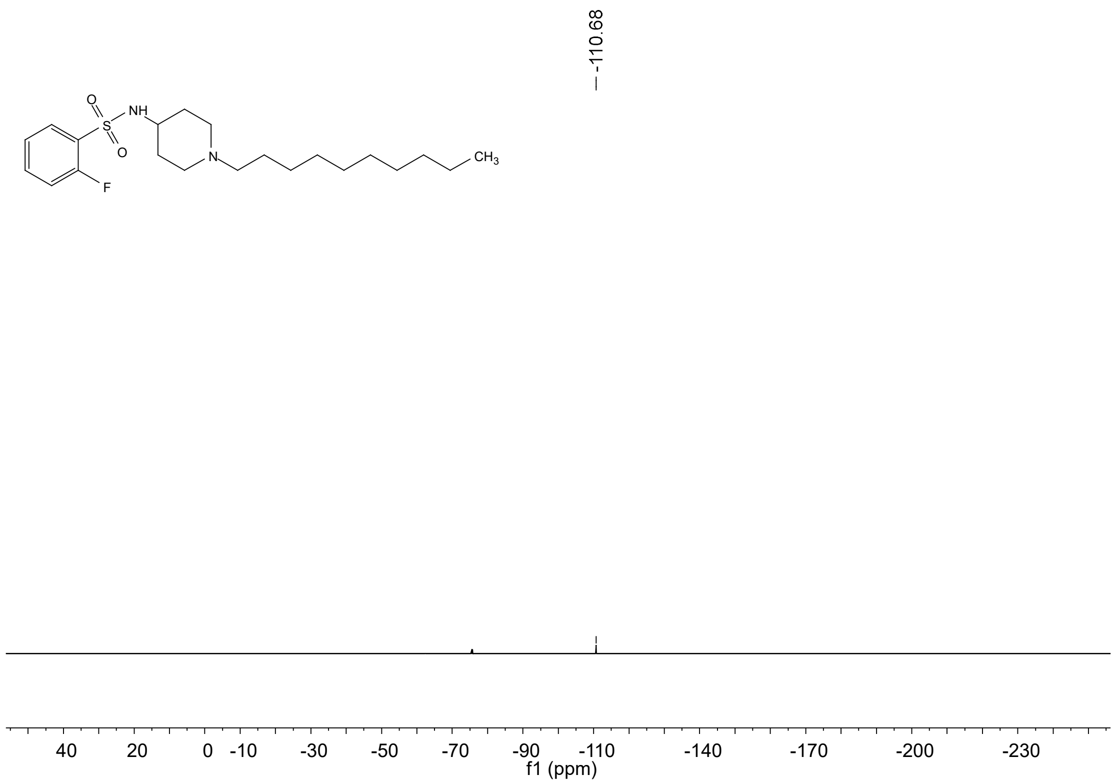

Figure S6. ^{19}F NMR spectrum ($CDCl_3$, 471 MHz) of **A1**.

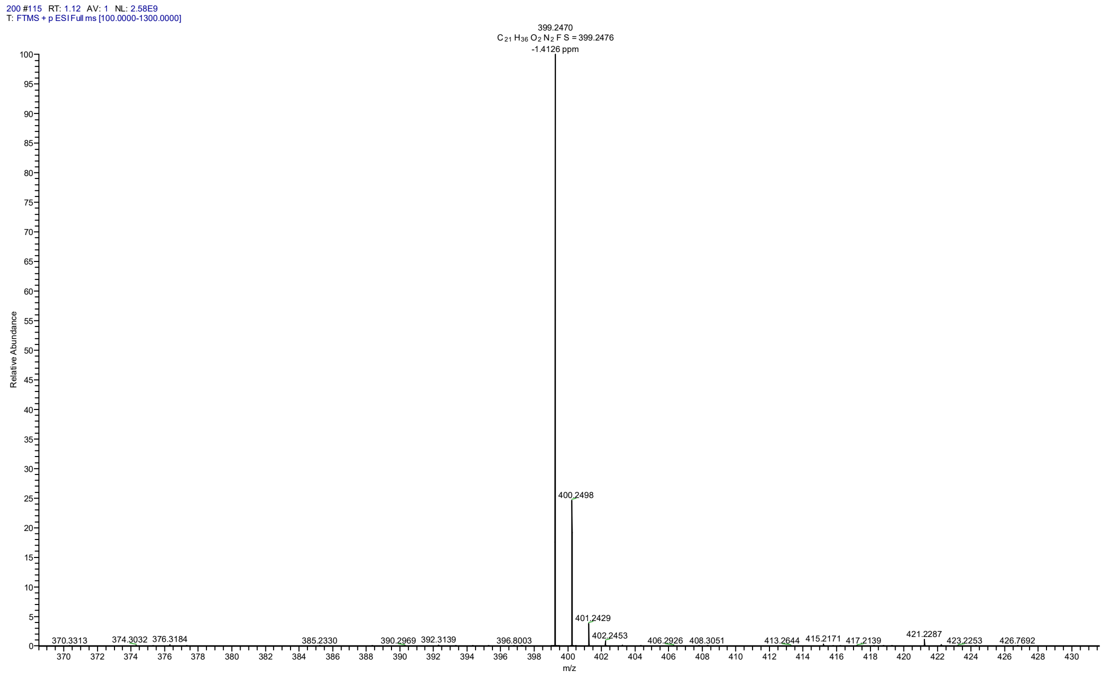

Figure S7. HRMS spectrum of **A1**.

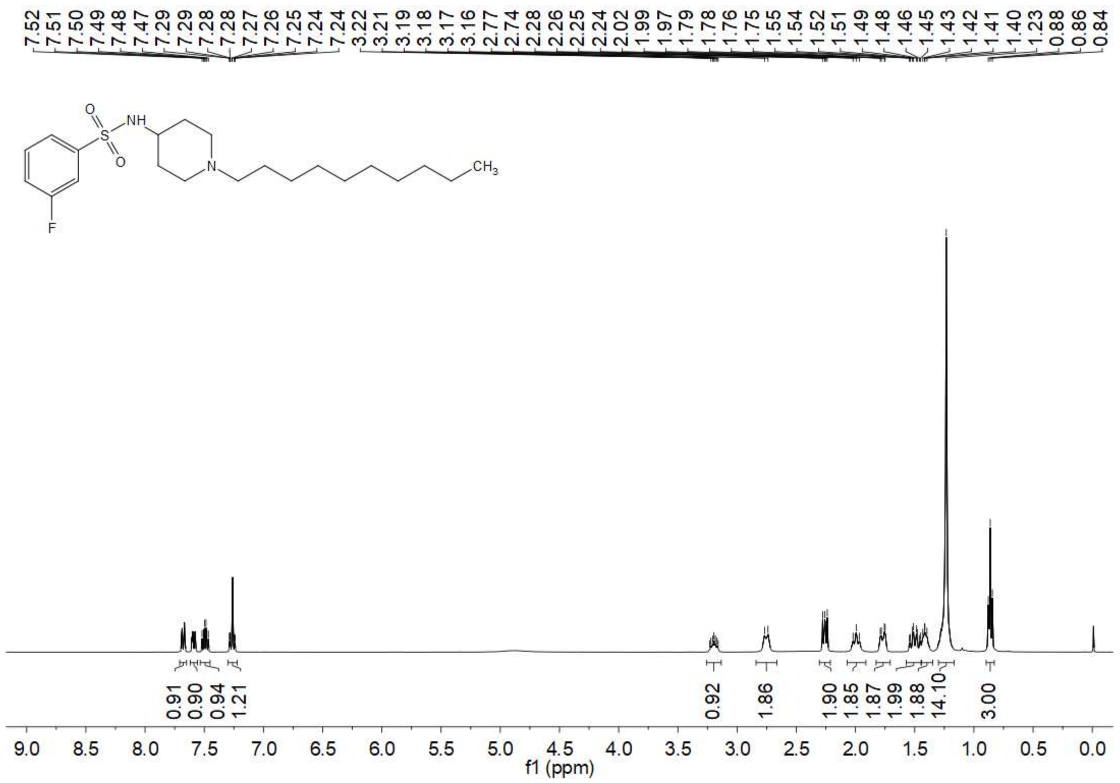

Figure S8. ^{1}H NMR spectrum ($CDCl_3$, 400 MHz) of $\mathbf{A_2}$.

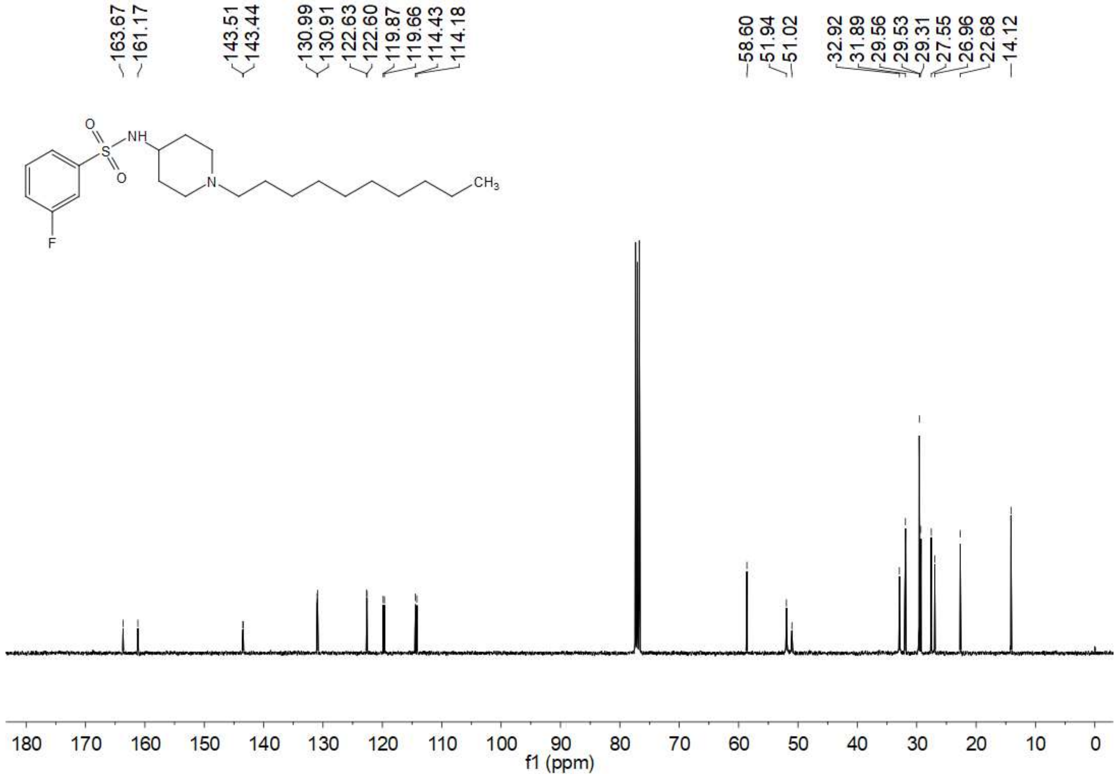

Figure S9. ^{13}C NMR spectrum ($CDCl_3$, 101 MHz) of $\mathbf{A_2}$.

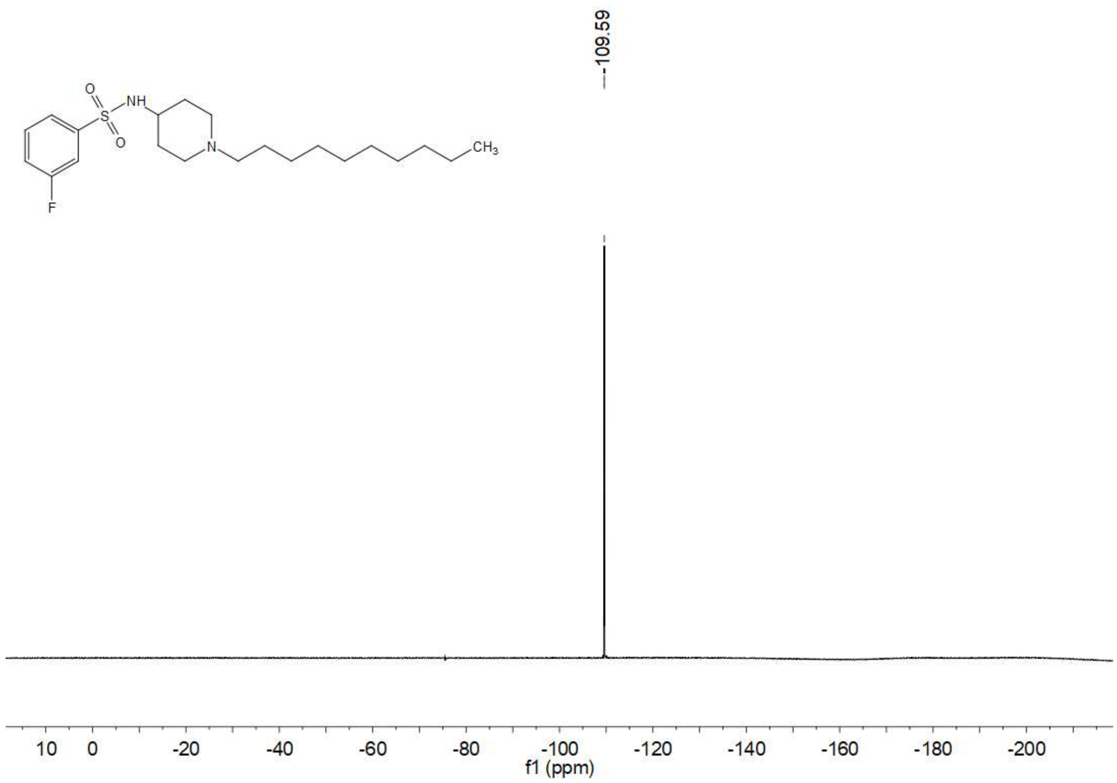

Figure S10. ^{19}F NMR spectrum ($CDCl_3$, 376 MHz) of **A2**.

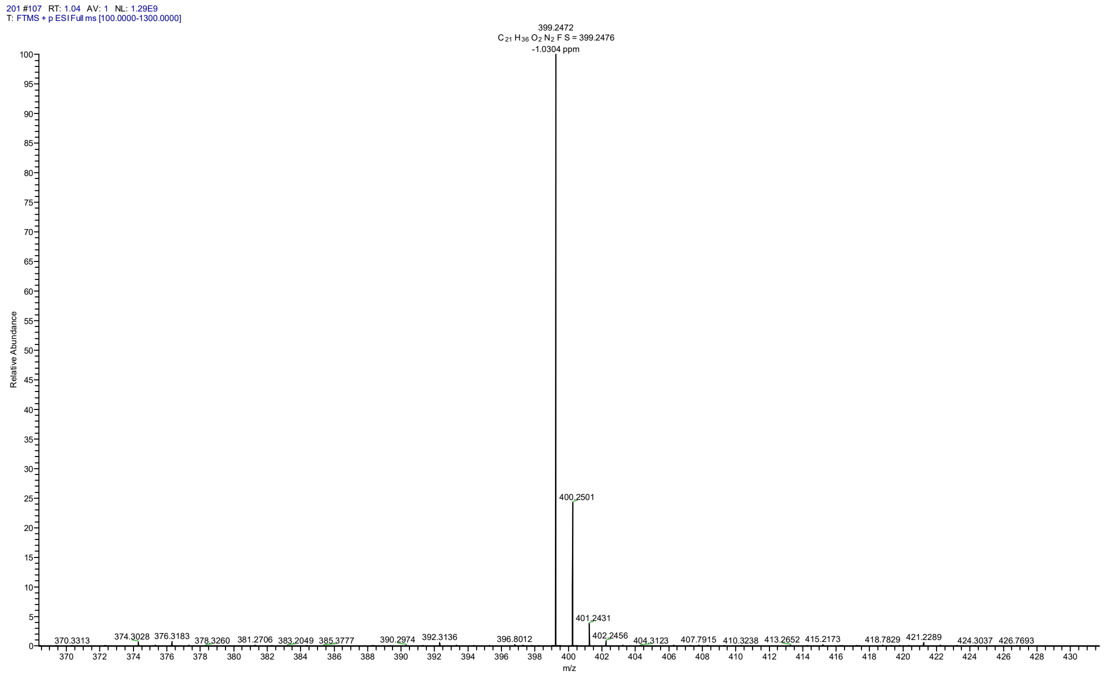

Figure S11. HRMS spectrum of **A2**.

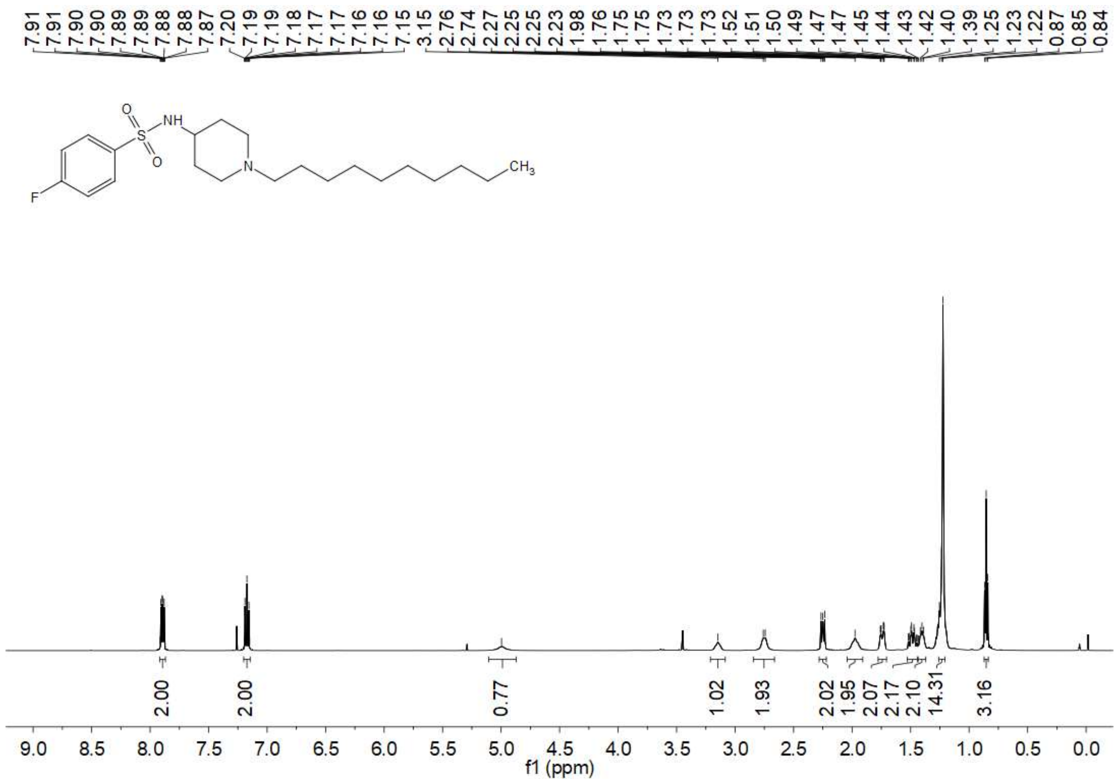

Figure S12. ^{1}H NMR spectrum ($CDCl_3$, 500 MHz) of **A3**.

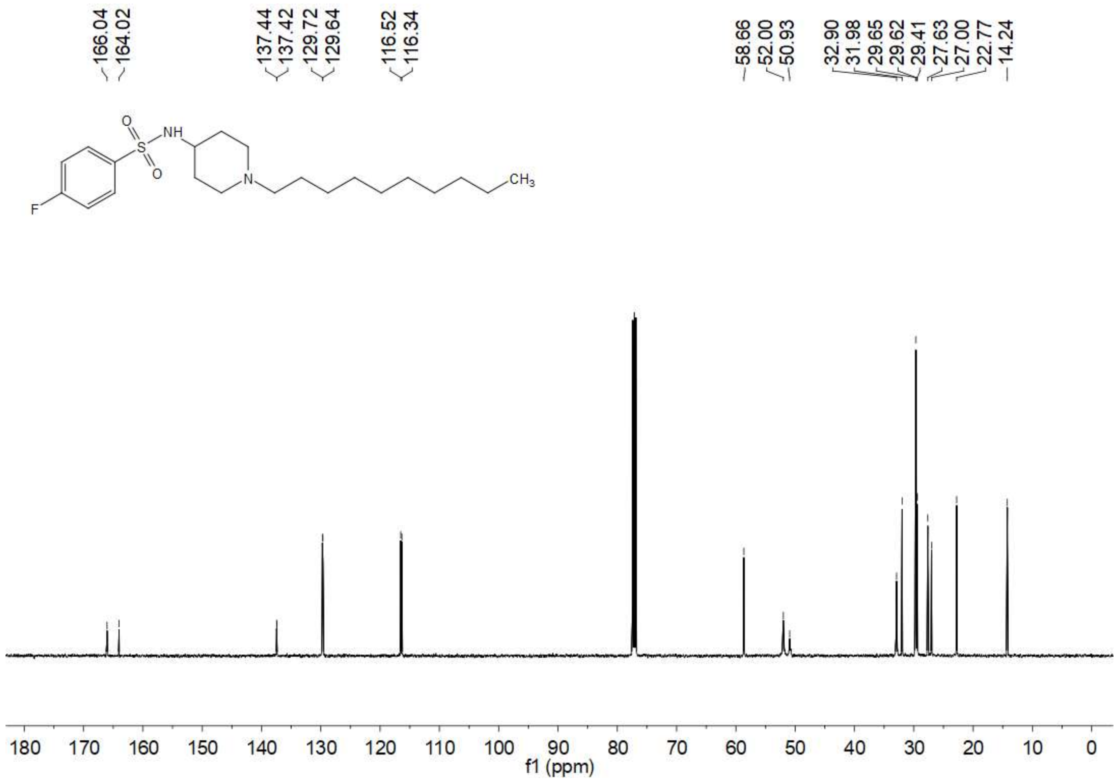

Figure S13. ^{13}C NMR spectrum ($CDCl_3$, 126 MHz) of **A3**.

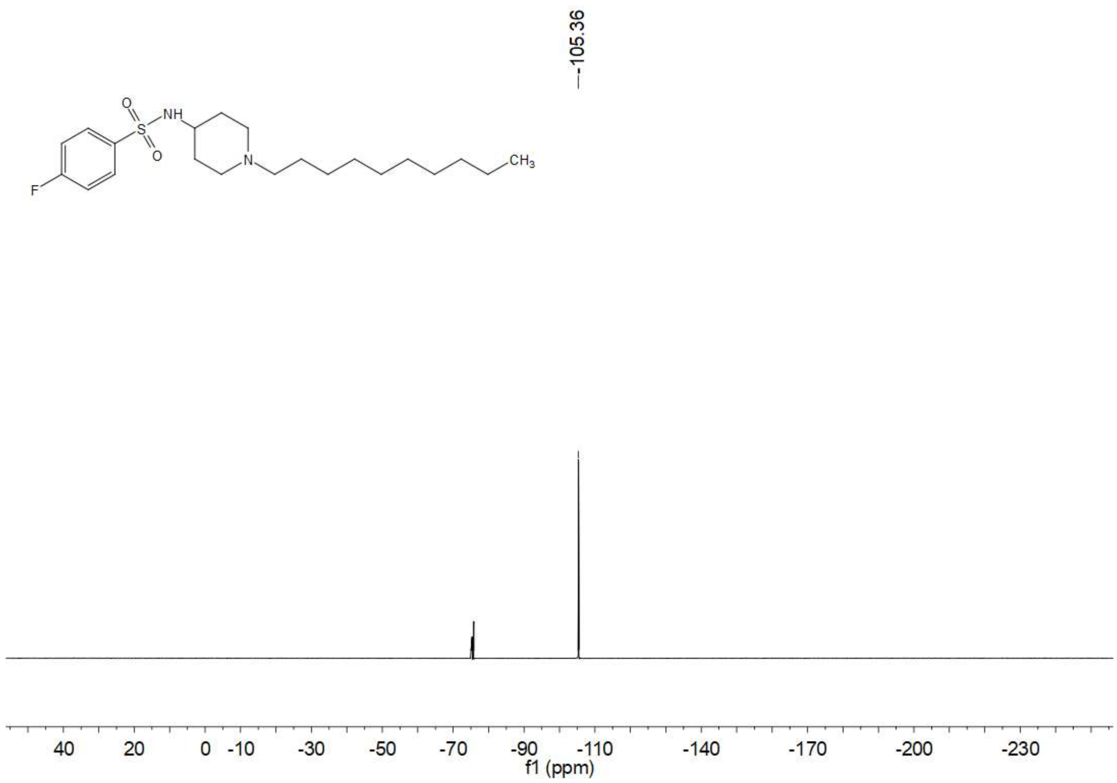

Figure S14. ^{19}F NMR spectrum ($CDCl_3$, 471 MHz) of **A3**.

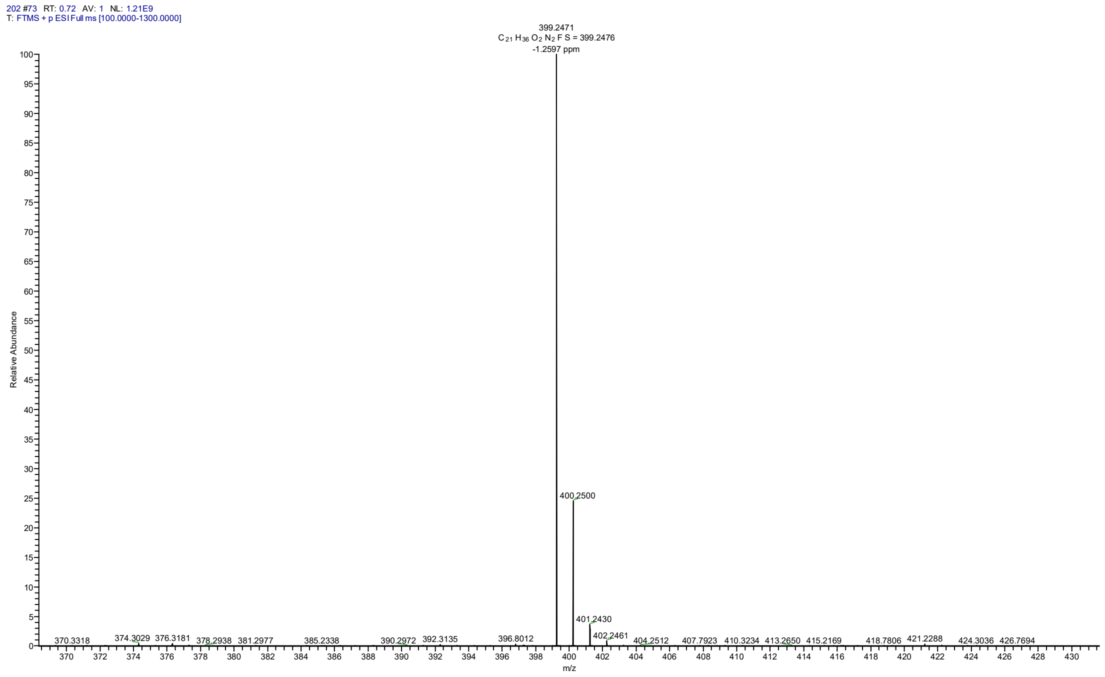

Figure S15. HRMS spectrum of **A3**.

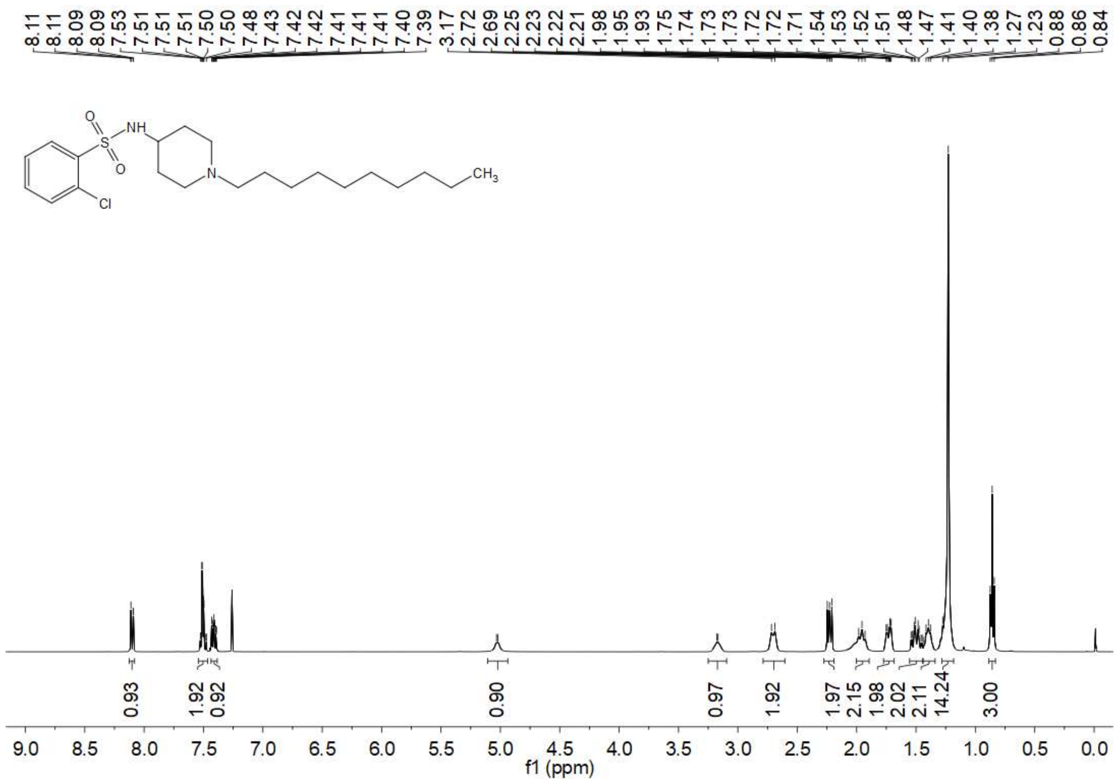

Figure S16. ^{1}H NMR spectrum ($CDCl_3$, 400 MHz) of **A4**.

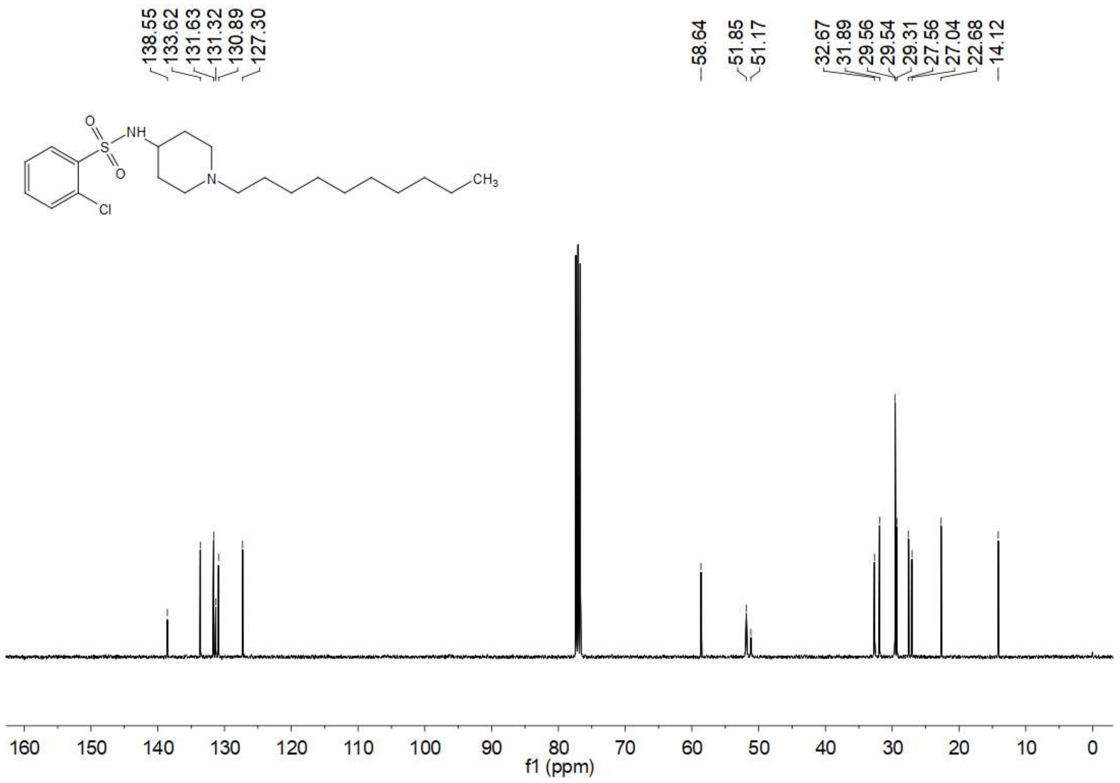

Figure S17. ^{13}C NMR spectrum ($CDCl_3$, 101 MHz) of **A4**.

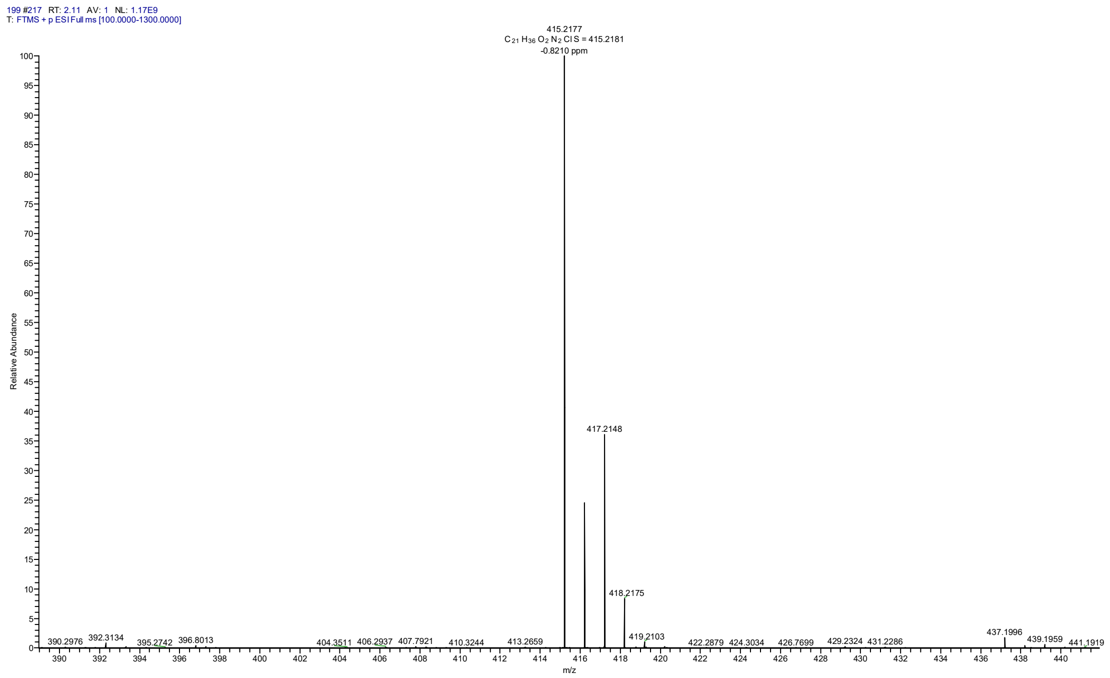

Figure S18. HRMS spectrum of **A4**.

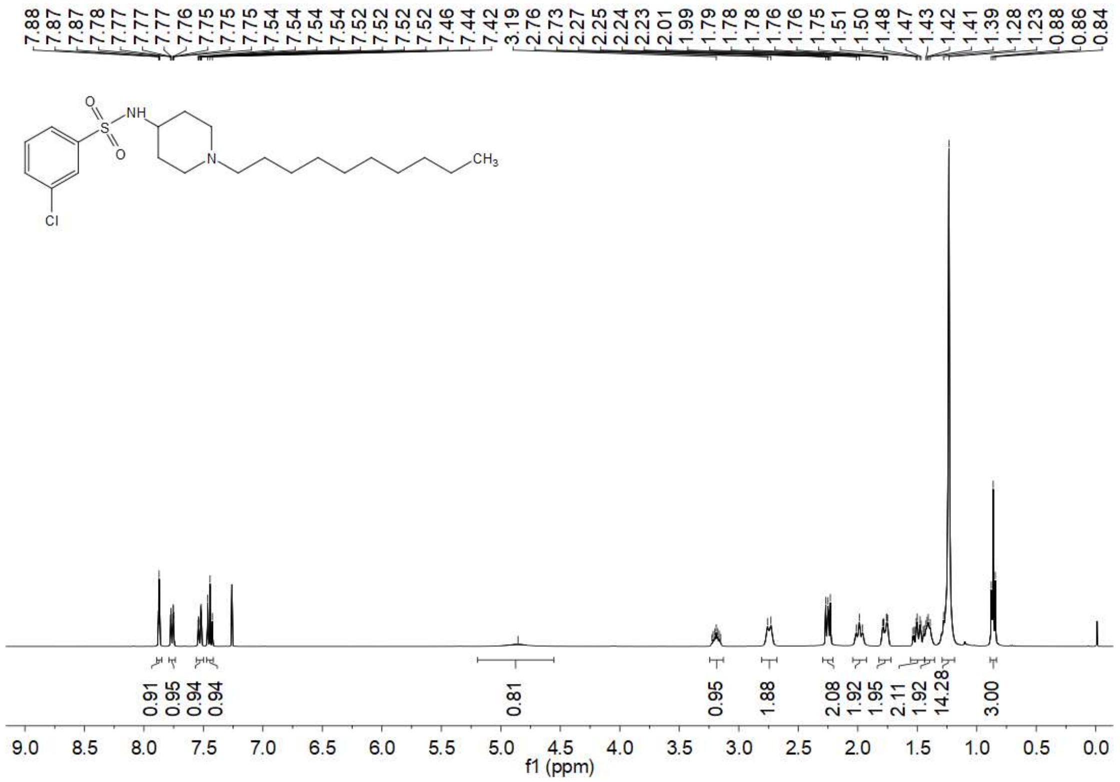

Figure S19. ^{1}H NMR spectrum ($CDCl_3$, 400 MHz) of **A5**.

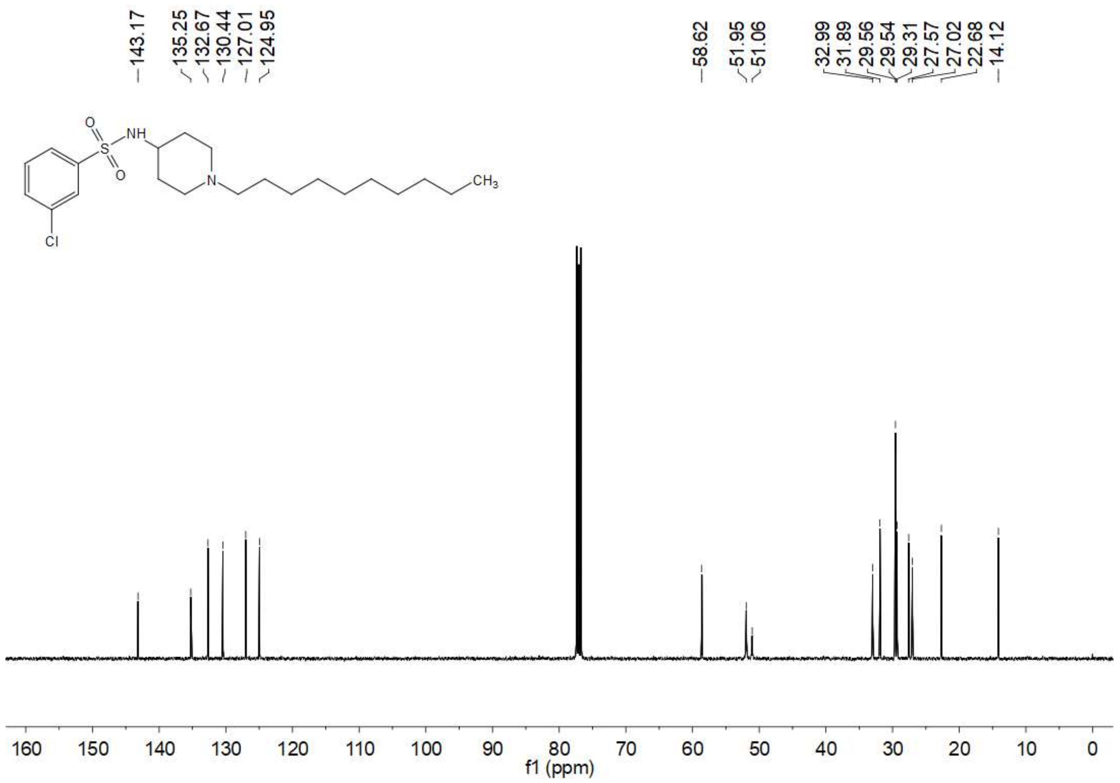

Figure S20. ^{13}C NMR spectrum ($CDCl_3$, 101 MHz) of **A5**.

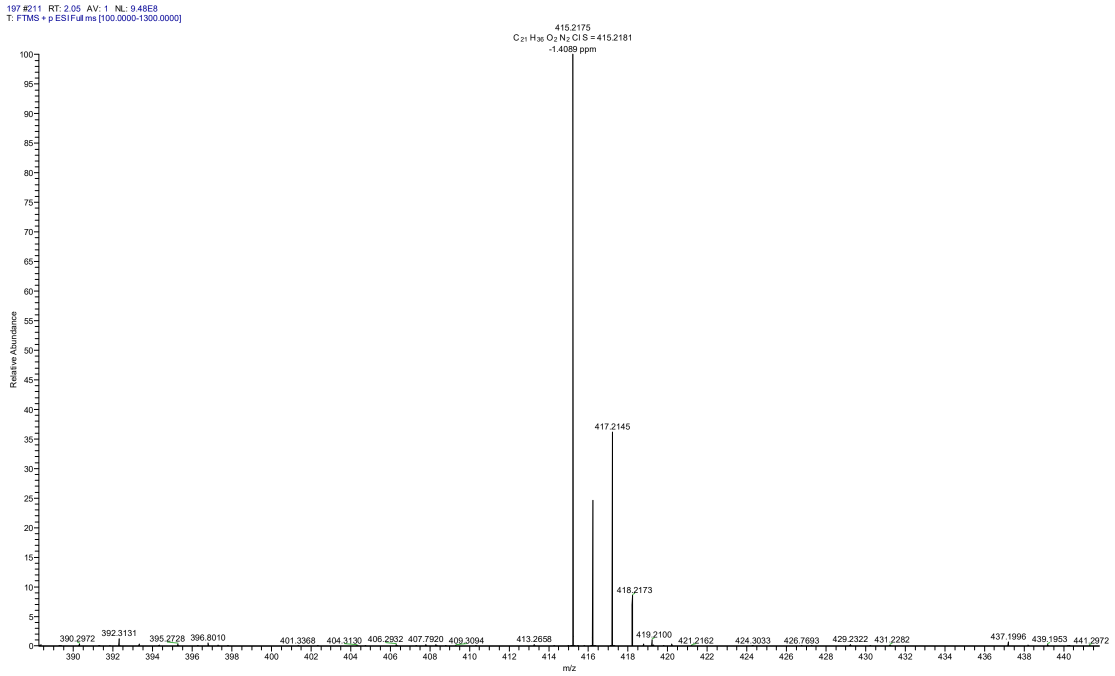

Figure S21. HRMS spectrum of **A5**.

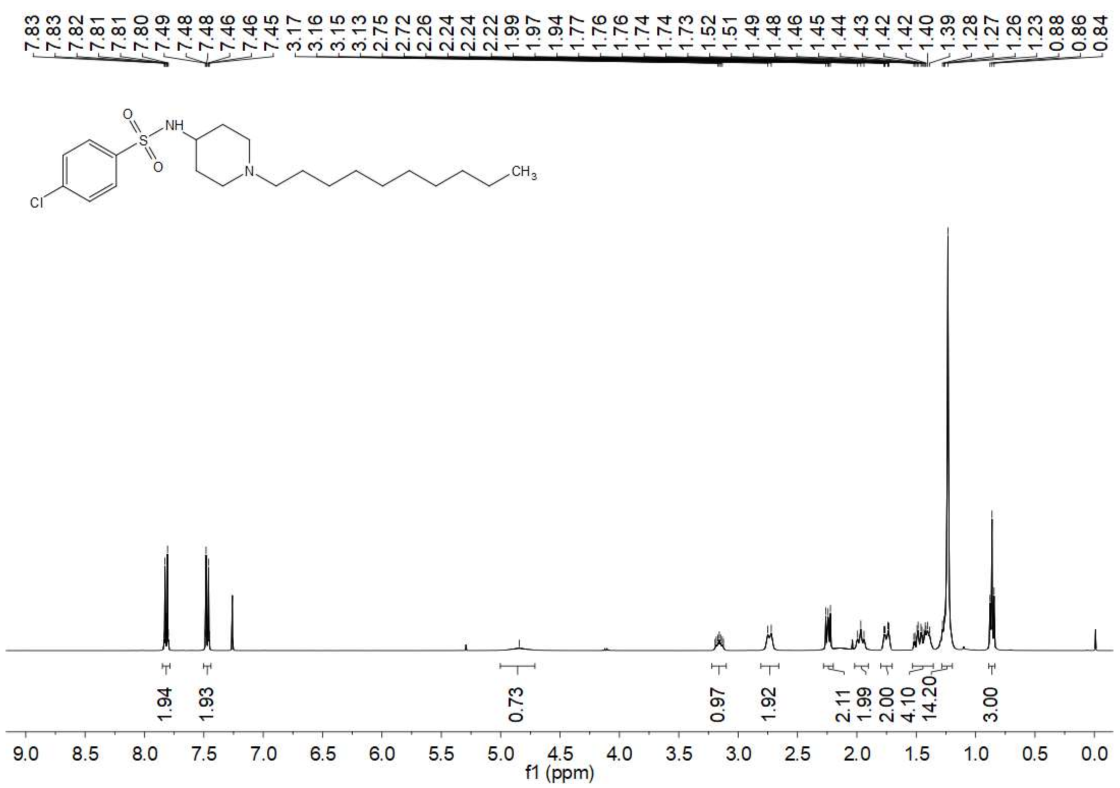

Figure S22. ^{1}H NMR spectrum ($CDCl_3$, 400 MHz) of $\mathbf{A_6}$.

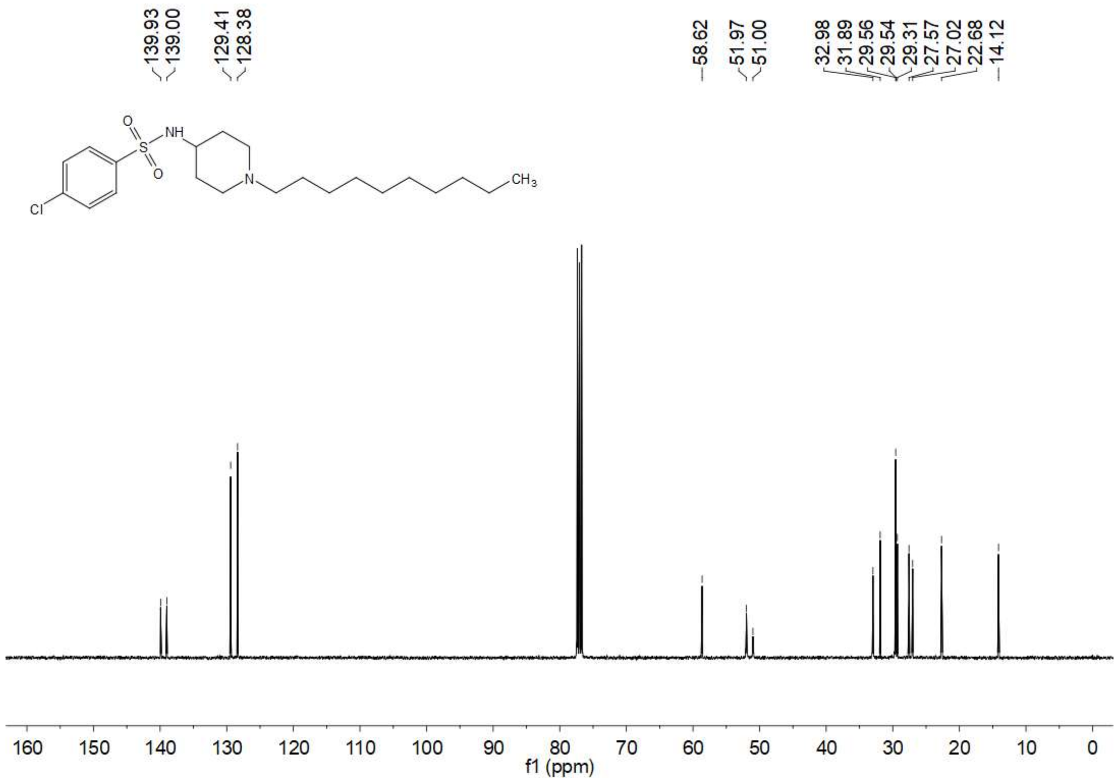

Figure S23. ^{13}C NMR spectrum ($CDCl_3$, 101 MHz) of $\mathbf{A_6}$.

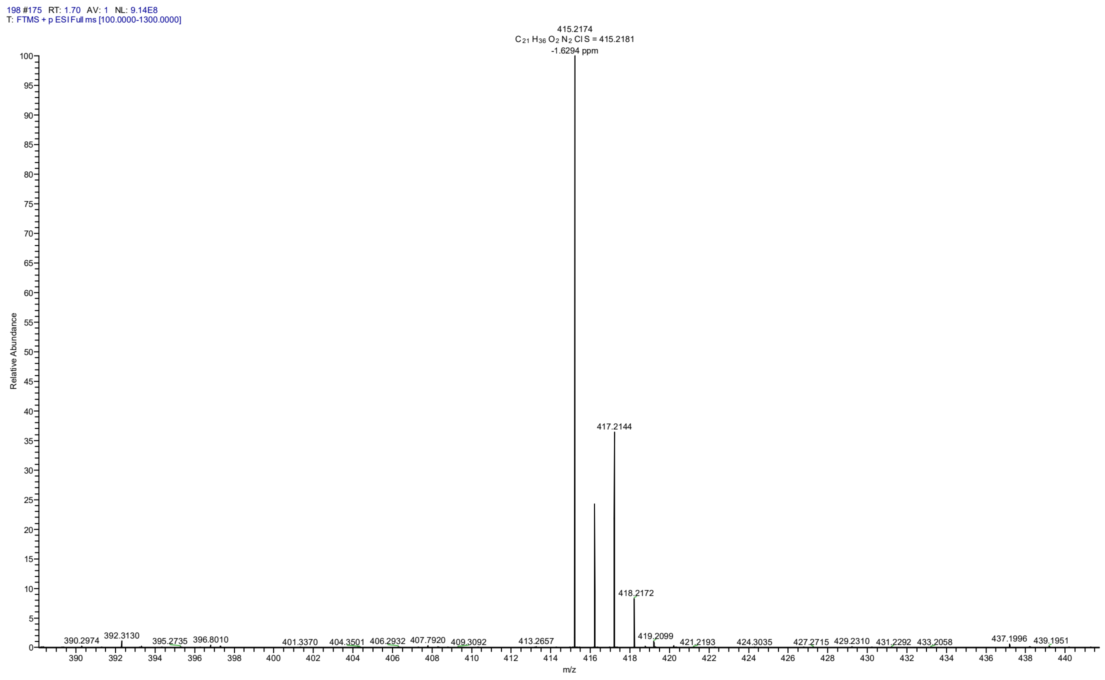

Figure S24. HRMS spectrum of **A**$_6$.

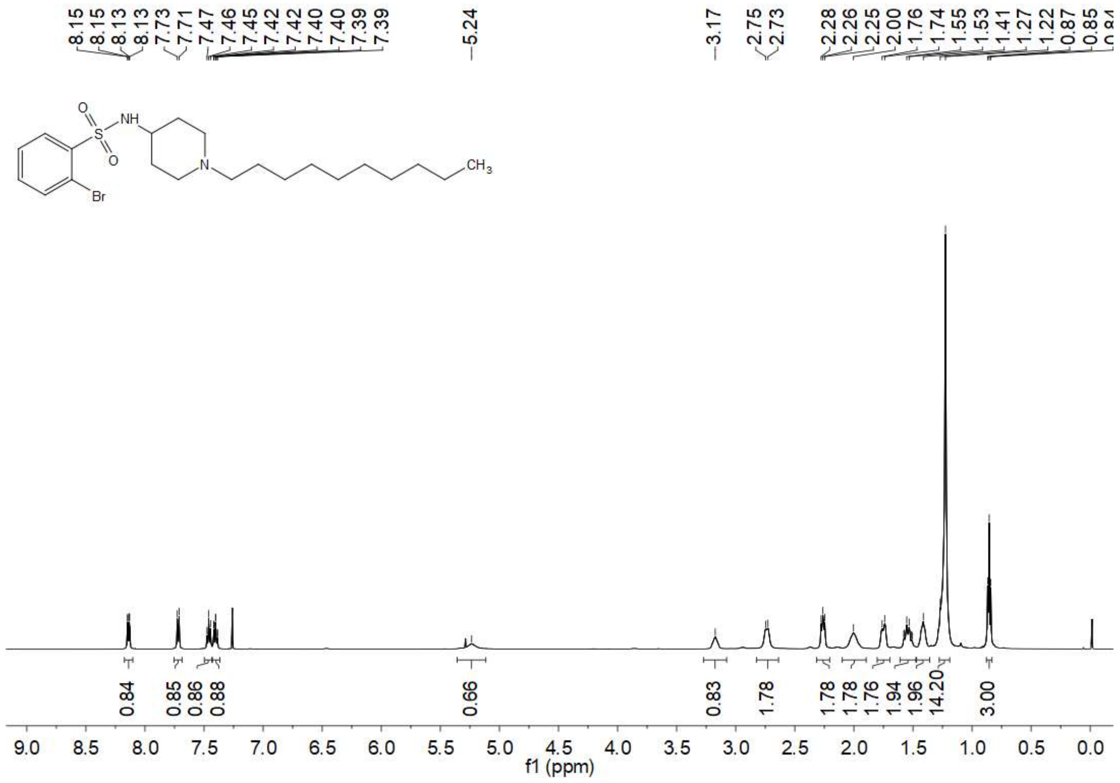

Figure S25. ^{1}H NMR spectrum ($CDCl_3$, 500 MHz) of **A**$_7$.

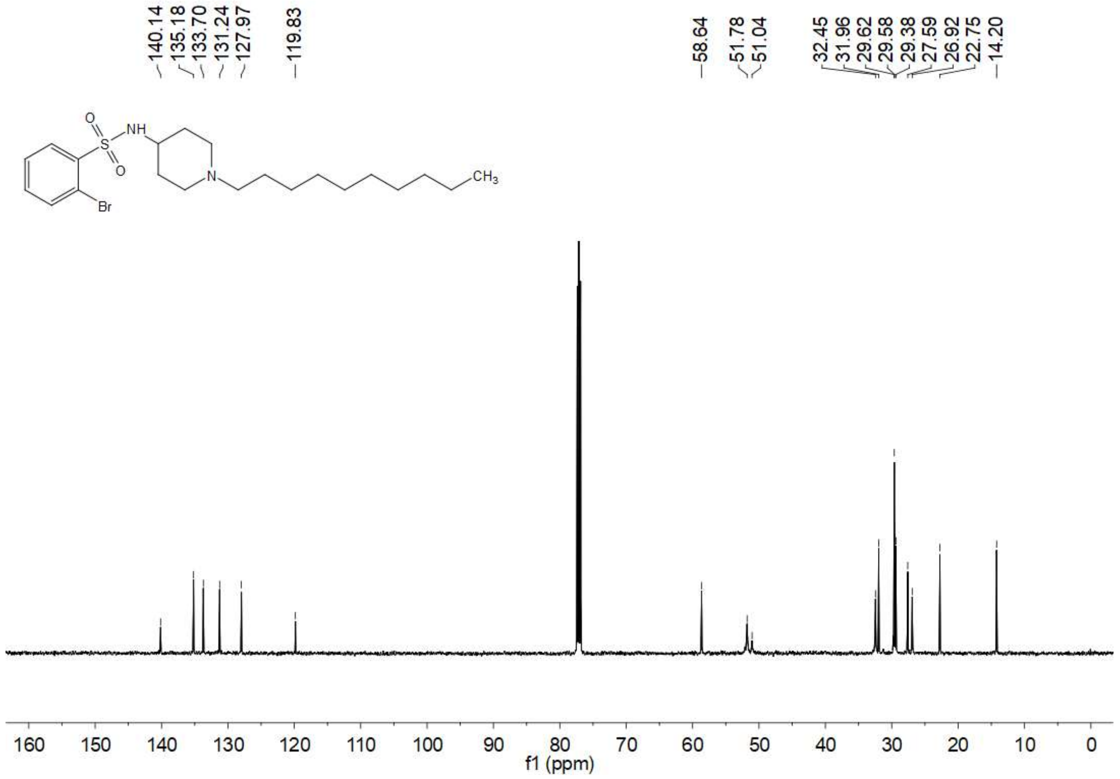

Figure S26. ^{13}C NMR spectrum ($CDCl_3$, 126 MHz) of **A7**.

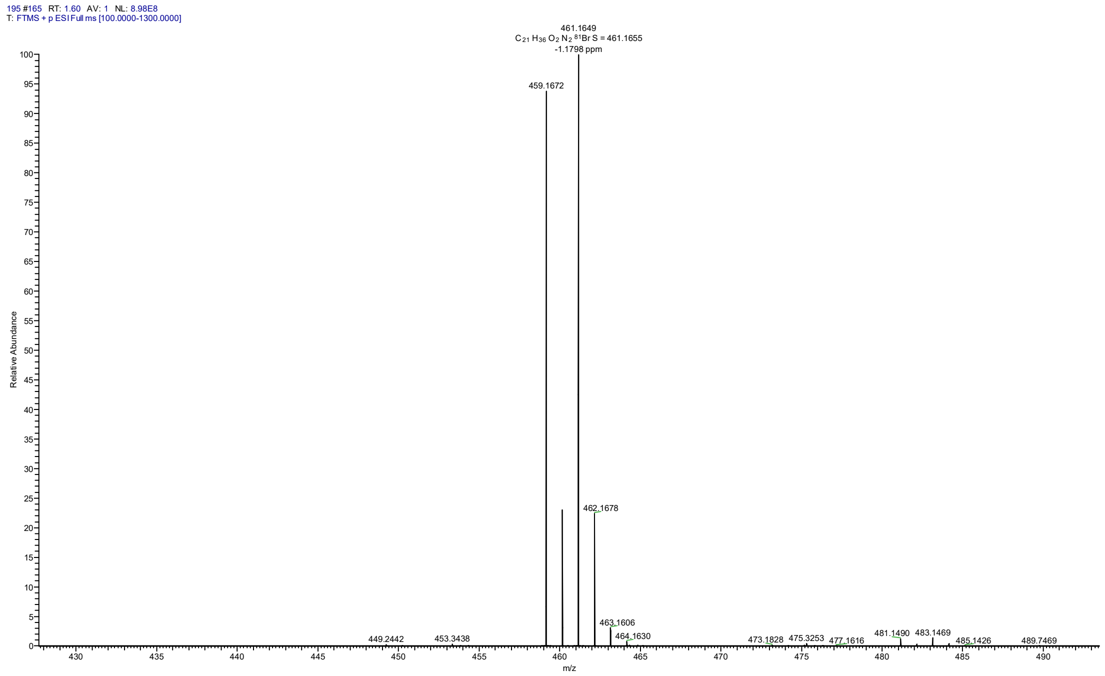

Figure S27. HRMS spectrum of **A7**.

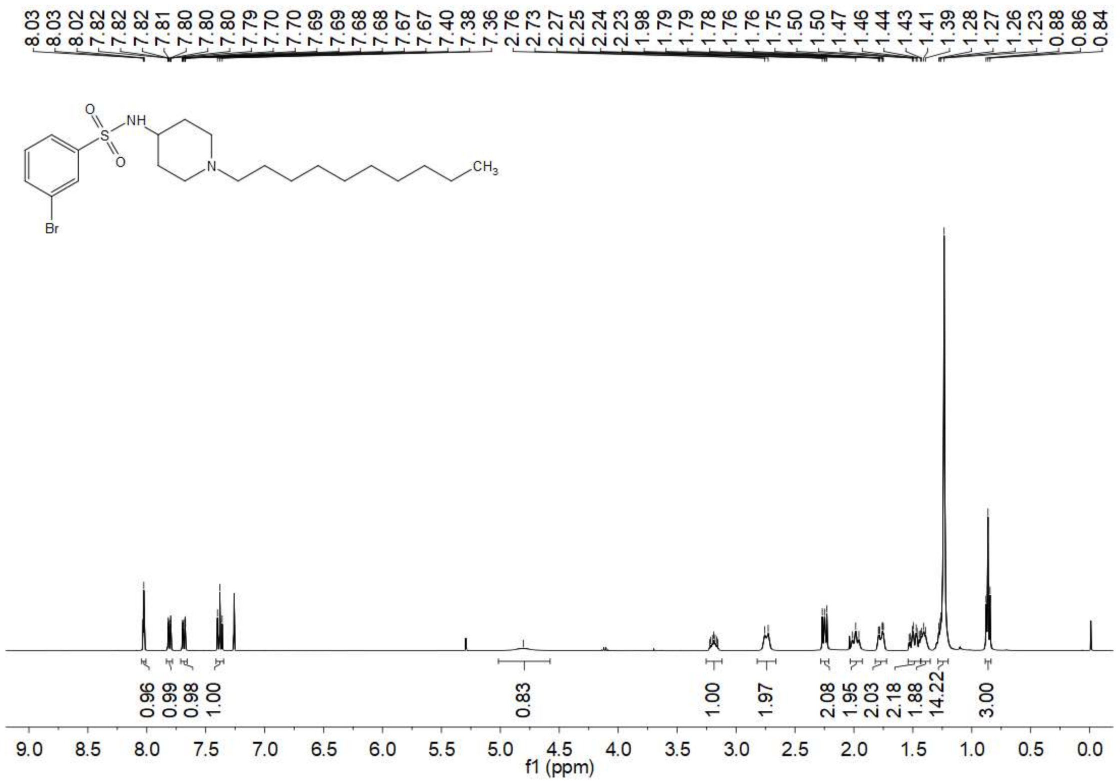

Figure S28. ^{1}H NMR spectrum ($CDCl_3$, 400 MHz) of **A**$_8$.

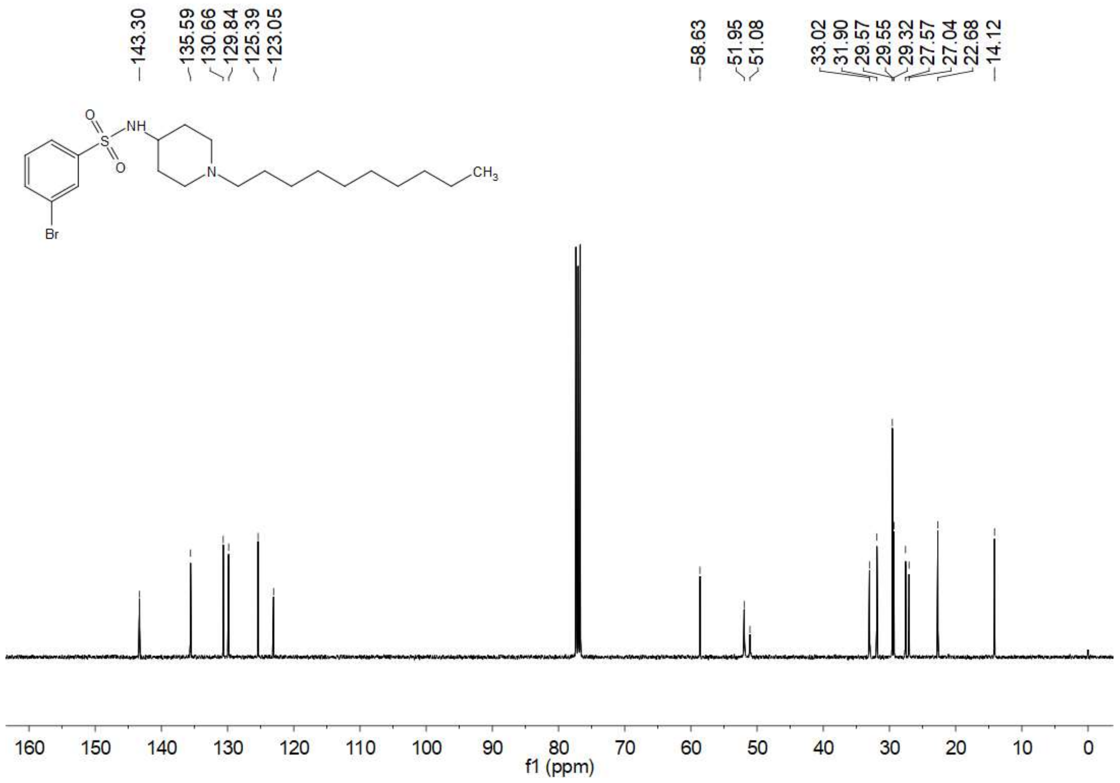

Figure S29. ^{13}C NMR spectrum ($CDCl_3$, 101 MHz) of **A**$_8$.

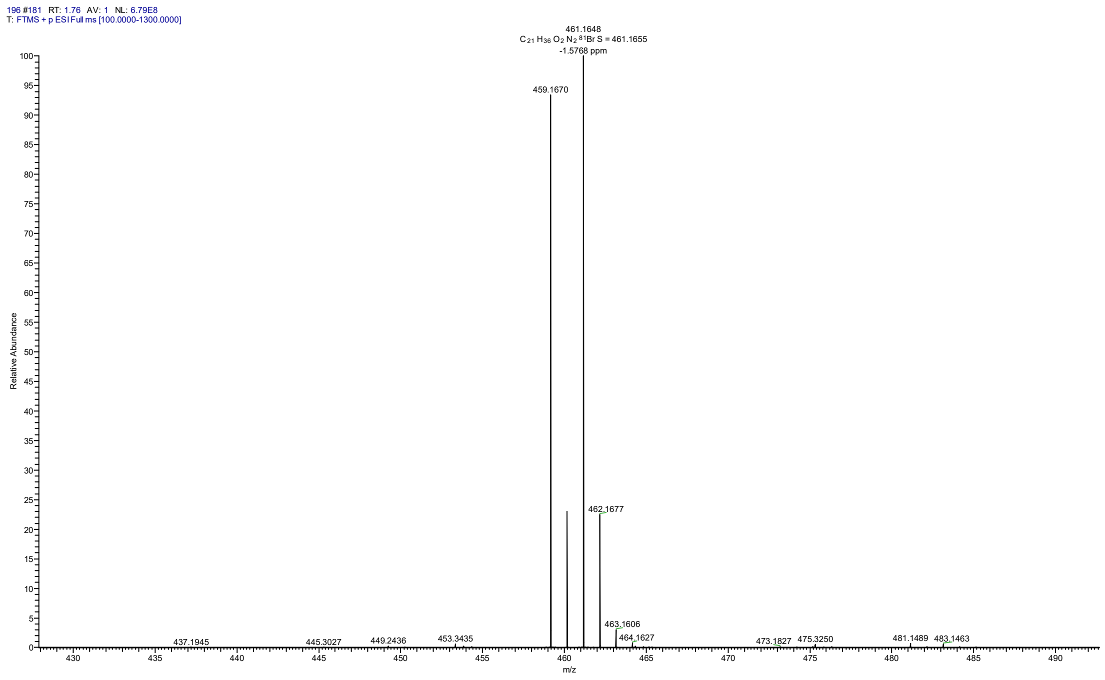

Figure S30. HRMS spectrum of **A8**.

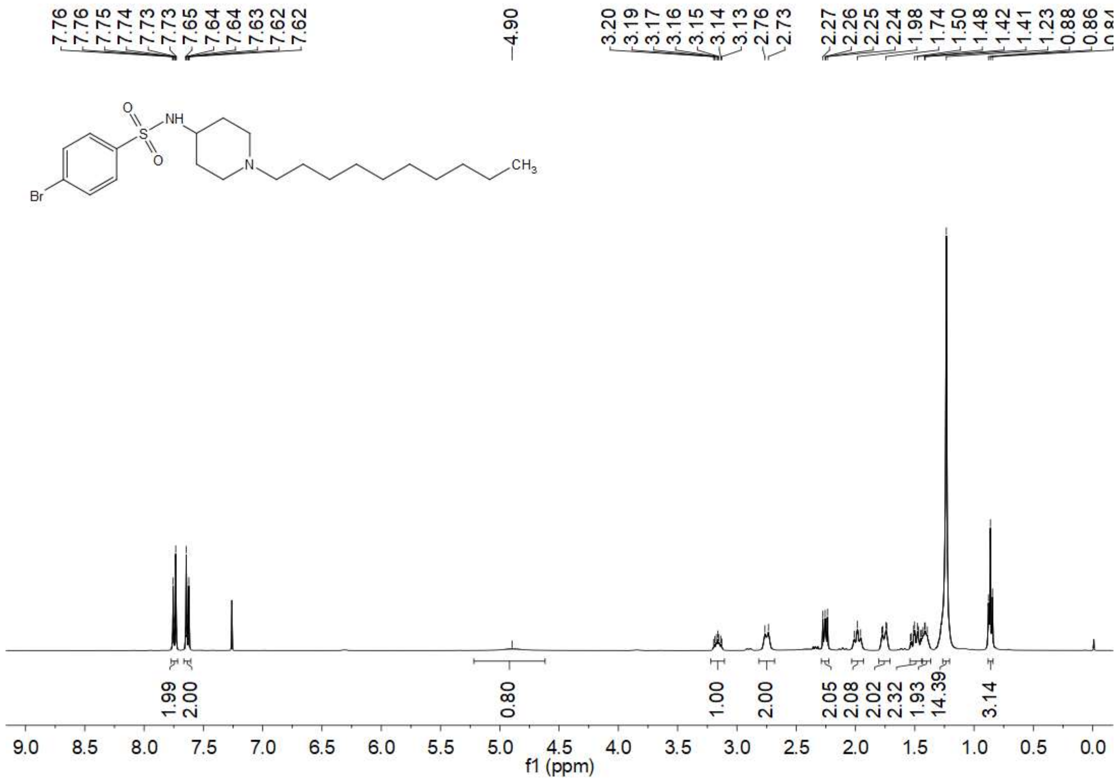

Figure S31. ^{1}H NMR spectrum ($CDCl_3$, 400 MHz) of **A9**.

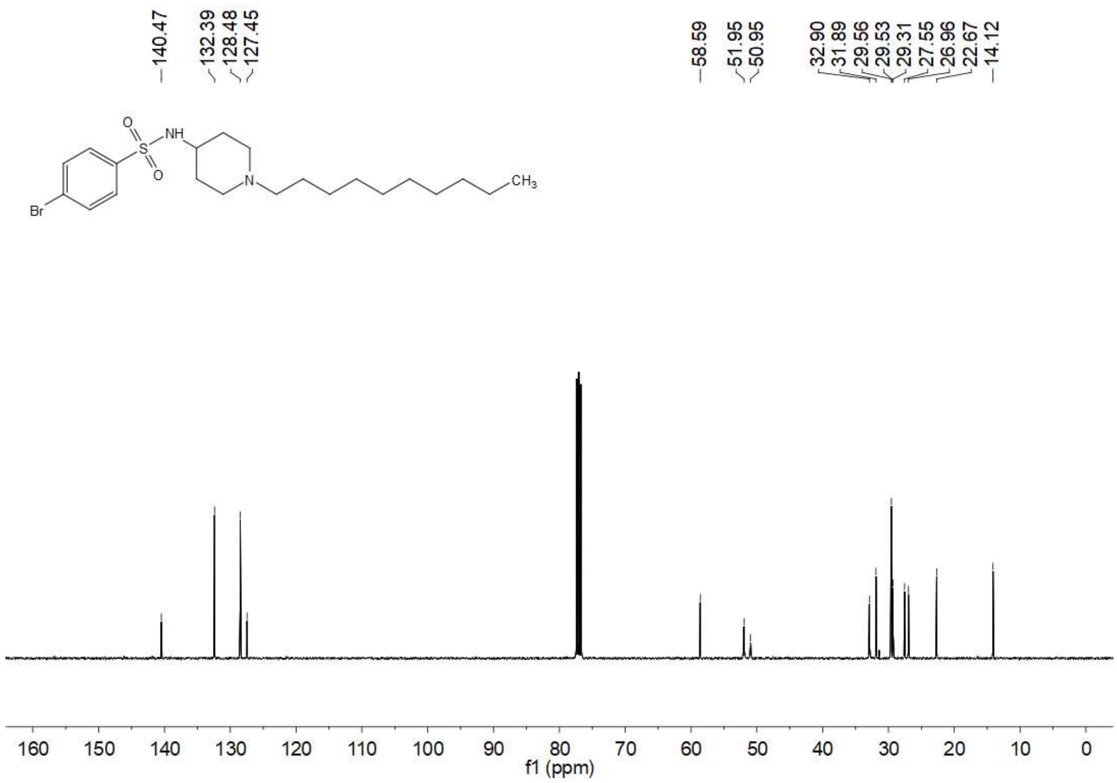

Figure S32. ^{13}C NMR spectrum ($CDCl_3$, 101 MHz) of **A9**.

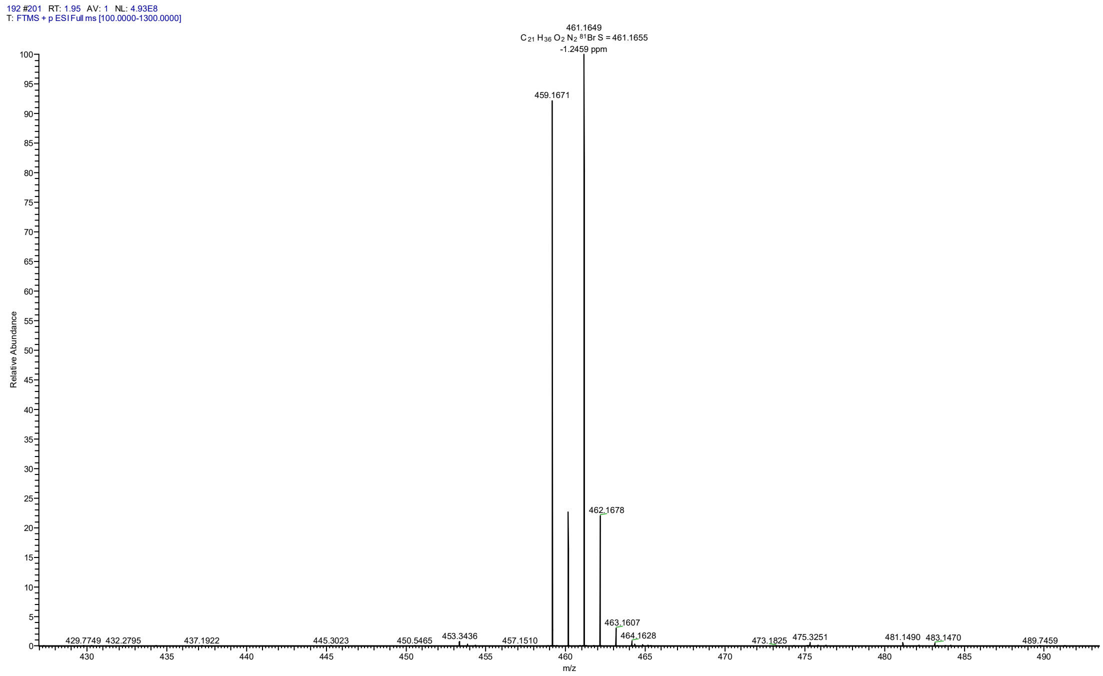

Figure S33. HRMS spectrum of **A9**.

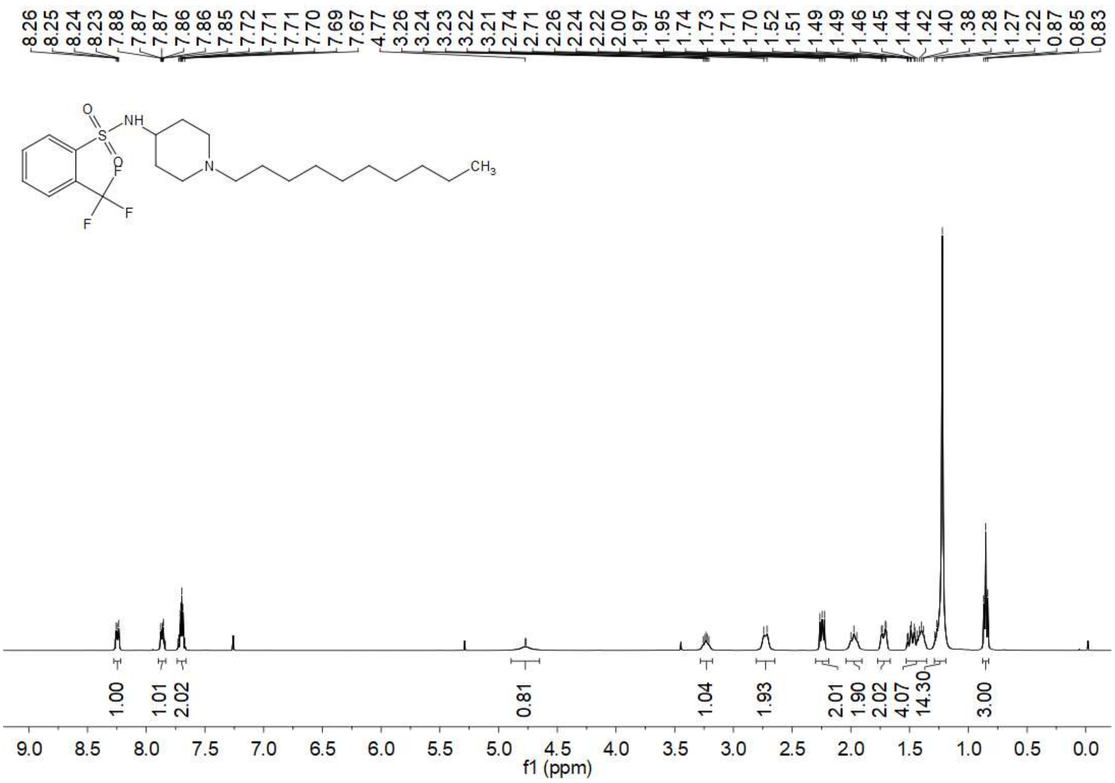

Figure S34. ^{1}H NMR spectrum ($CDCl_3$, 400 MHz) of **A_{10}**.

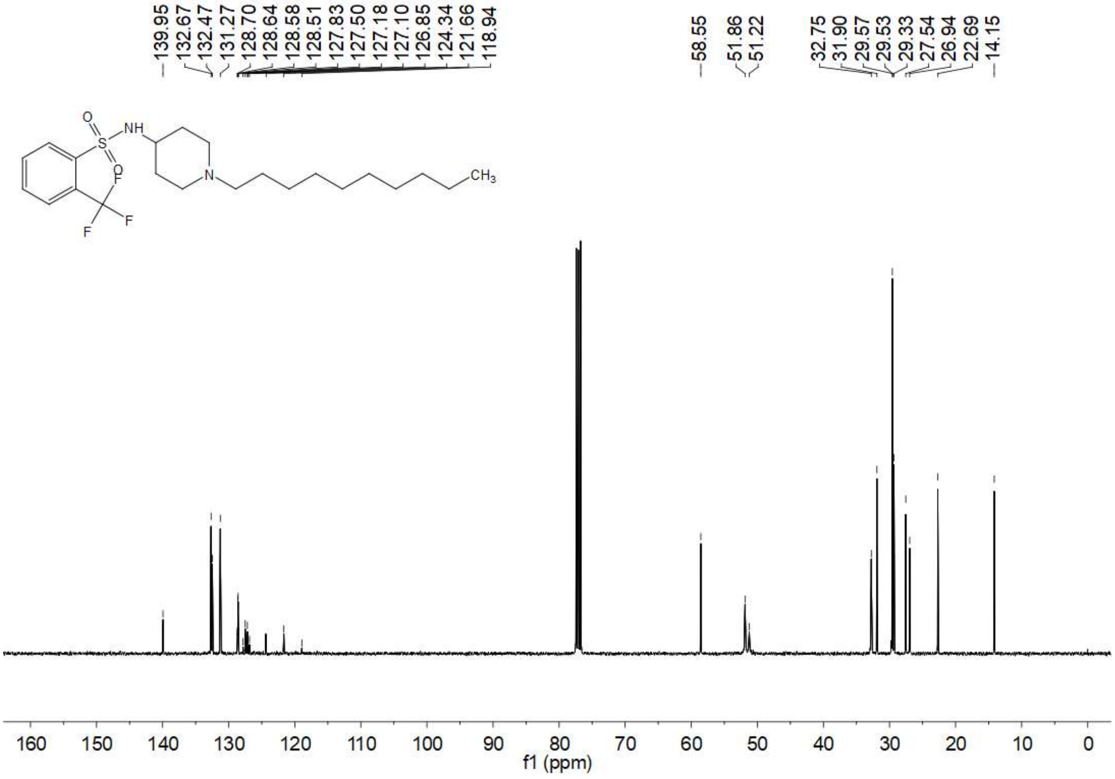

Figure S35. ^{13}C NMR spectrum ($CDCl_3$, 101 MHz) of **A_{10}**.

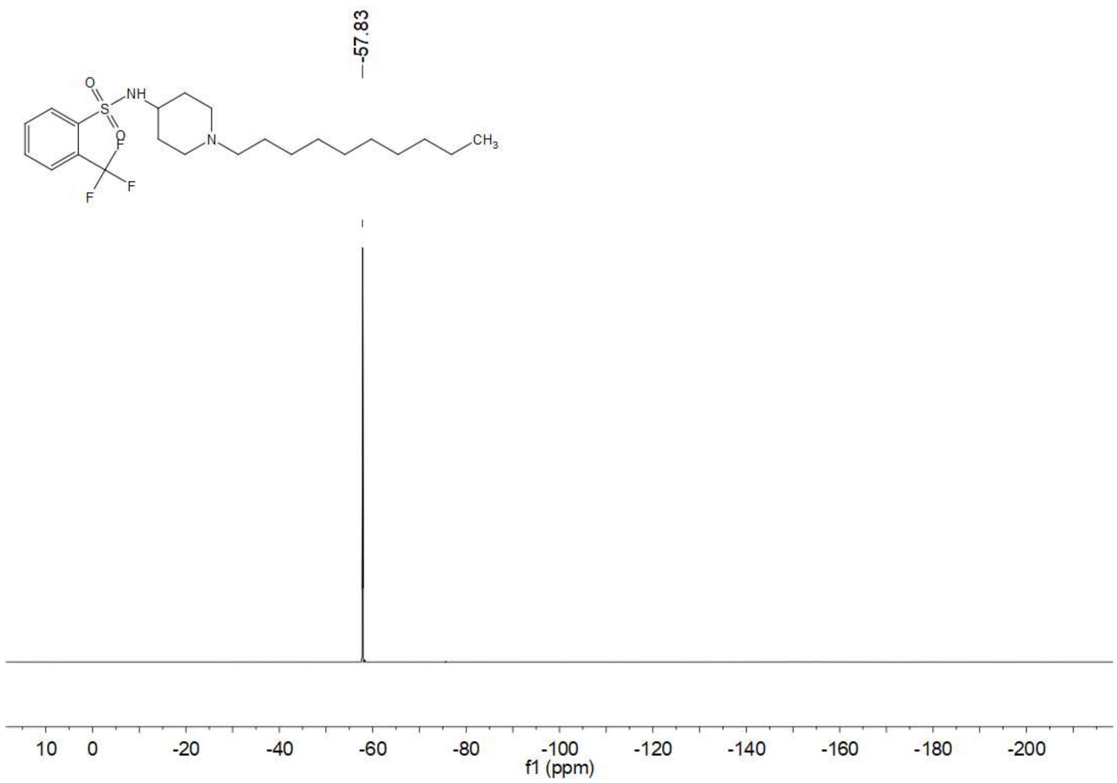

Figure S36. ^{19}F NMR spectrum ($CDCl_3$, 376 MHz) of **A10**.

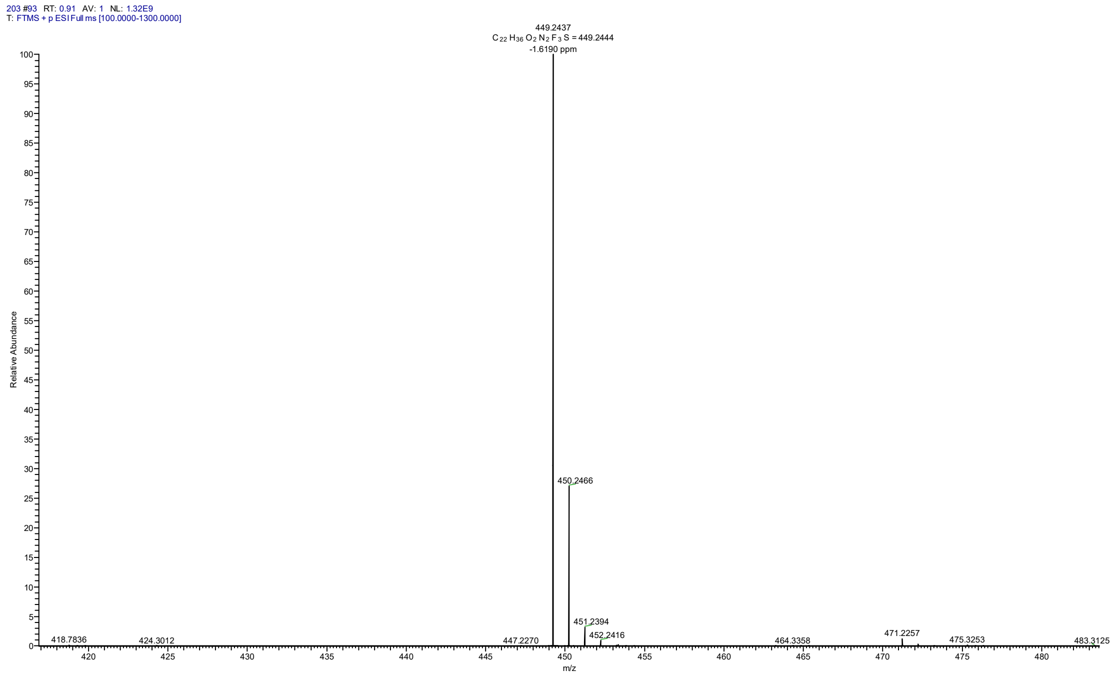

Figure S37. HRMS spectrum of **A10**.

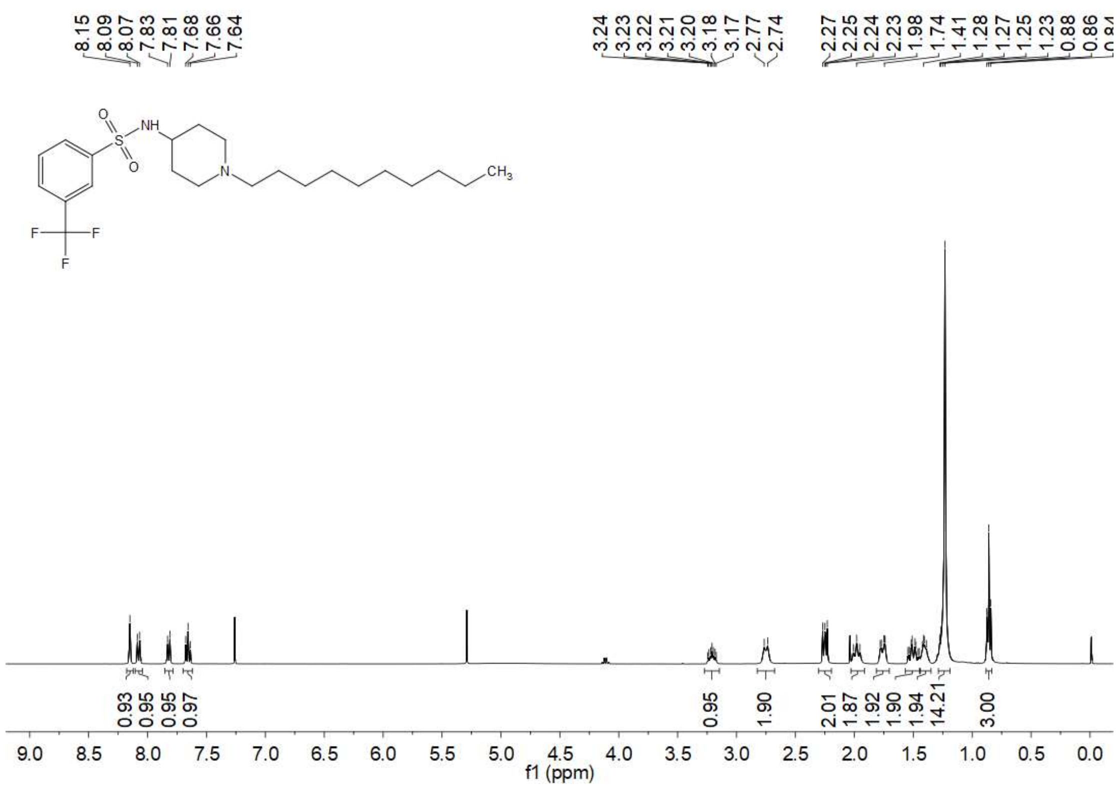

Figure S38. ^{1}H NMR spectrum ($CDCl_3$, 400 MHz) of $\mathbf{A_{11}}$.

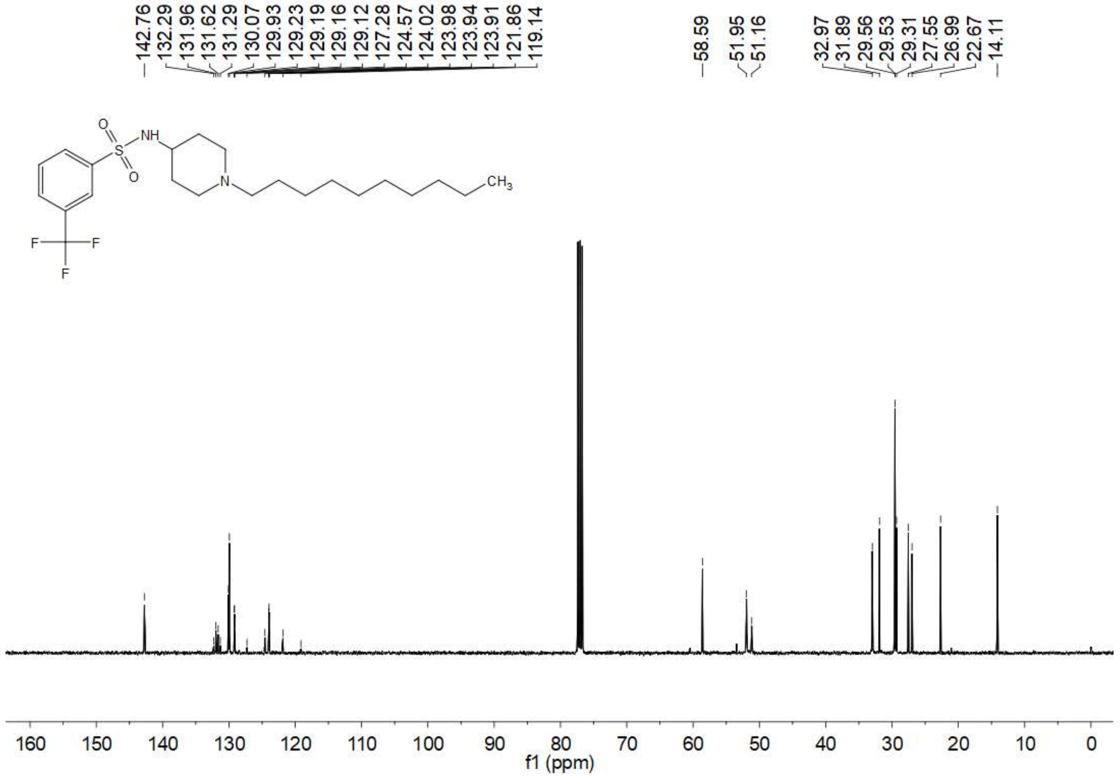

Figure S39. ^{13}C NMR spectrum ($CDCl_3$, 101 MHz) of $\mathbf{A_{11}}$.

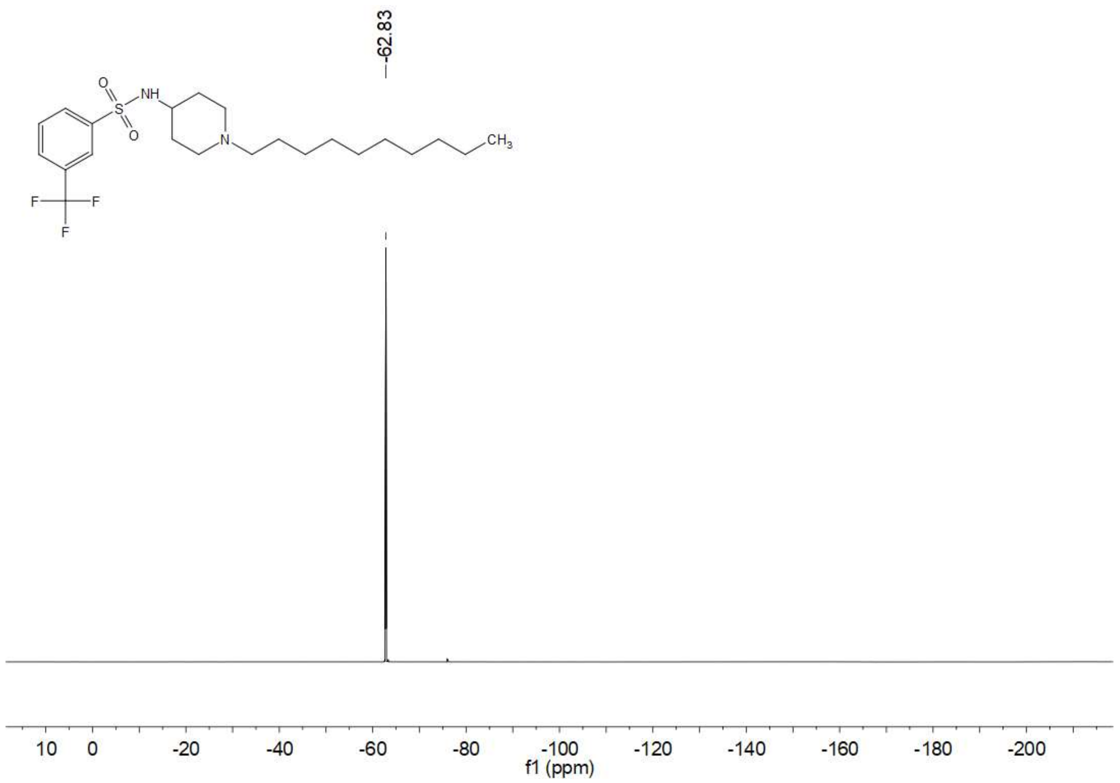

Figure S40. ^{19}F NMR spectrum ($CDCl_3$, 376 MHz) of **A11**.

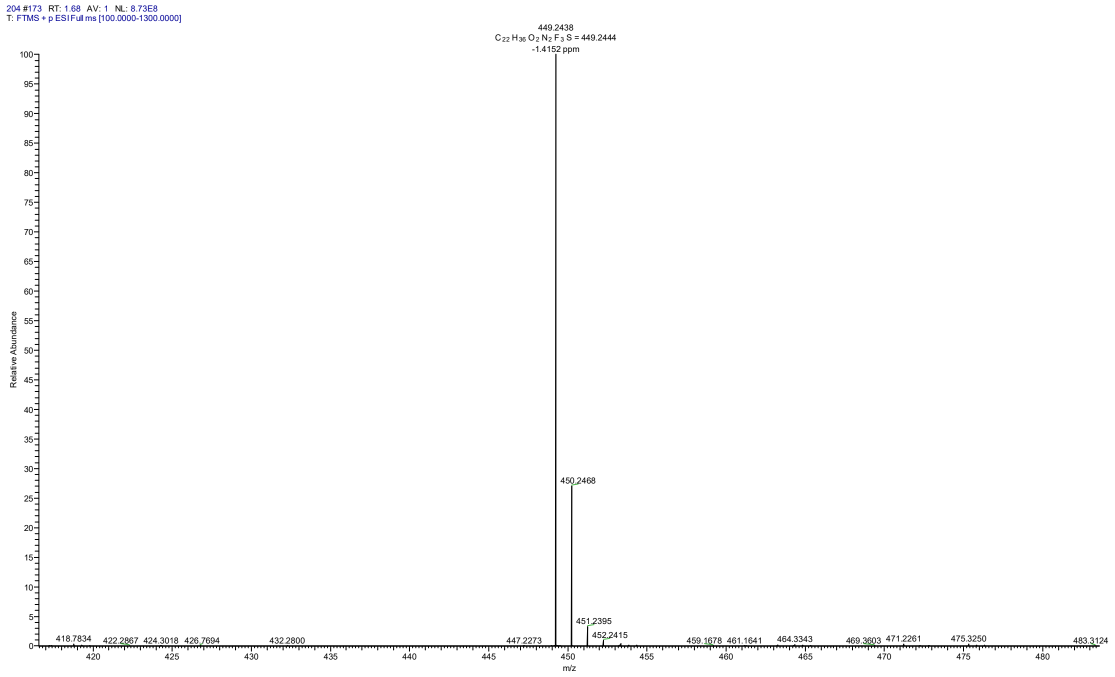

Figure S41. HRMS spectrum of **A11**.

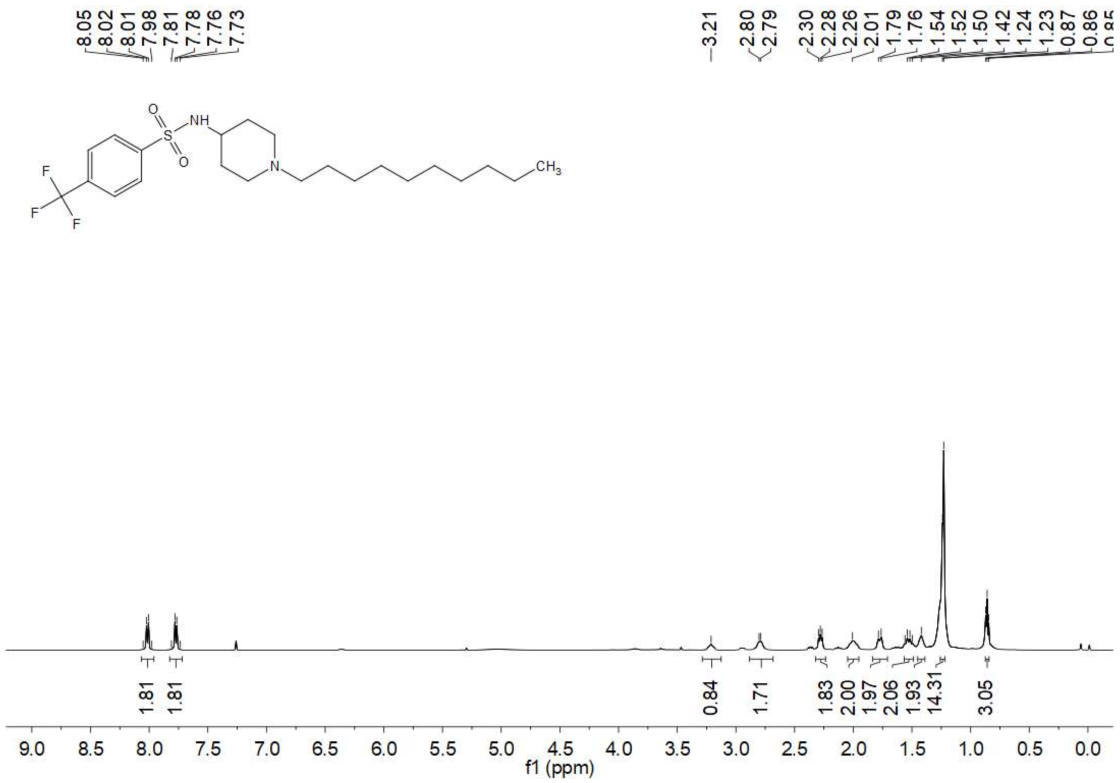

Figure S42. ^{1}H NMR spectrum ($CDCl_3$, 500 MHz) of **A_{12}**.

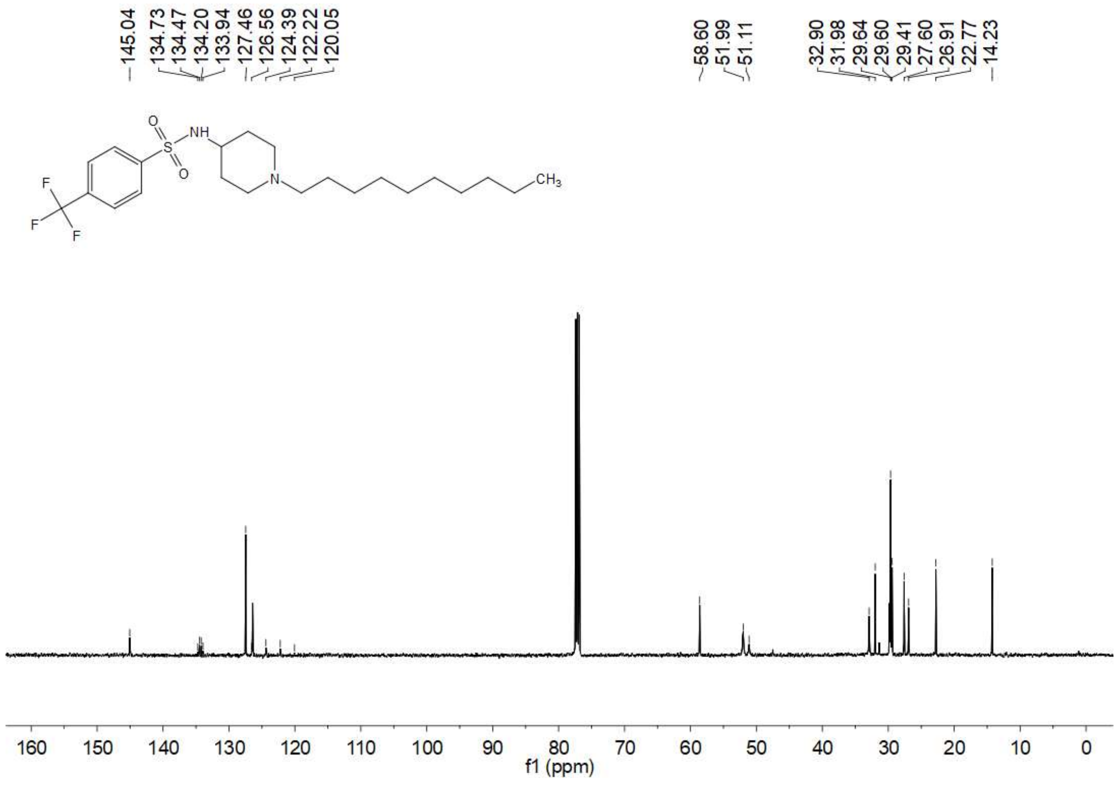

Figure S43. ^{13}C NMR spectrum ($CDCl_3$, 126 MHz) of **A_{12}**.

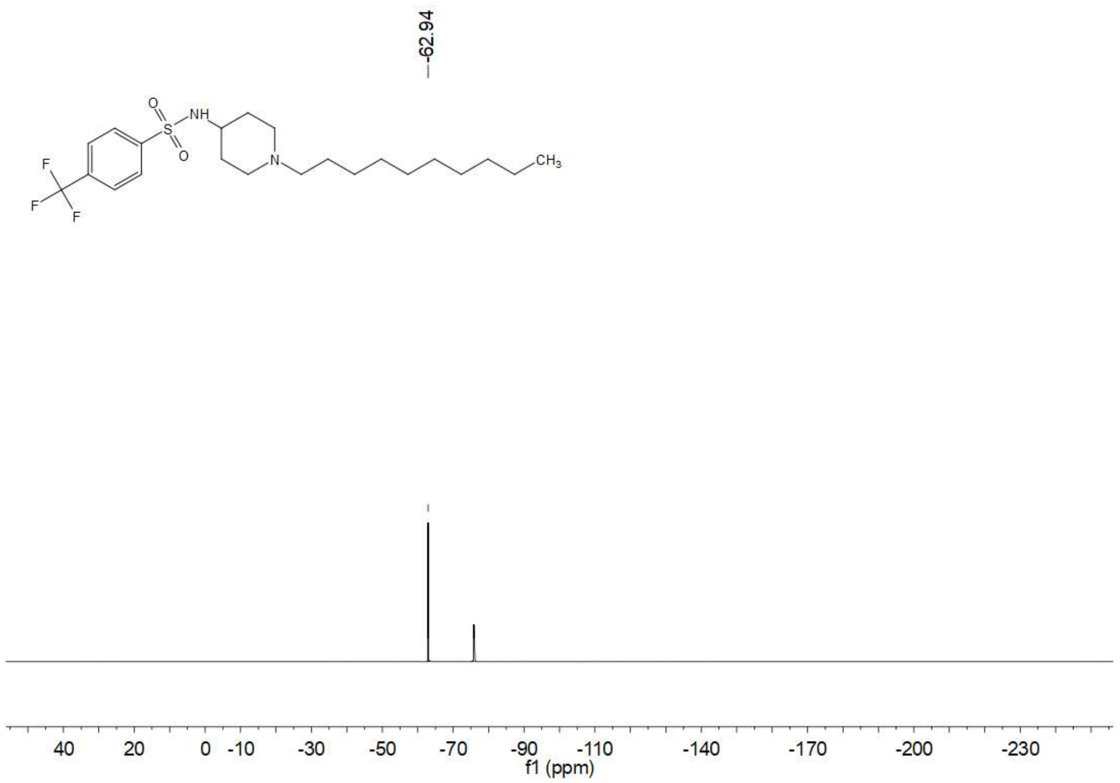

Figure S44. ^{19}F NMR spectrum ($CDCl_3$, 471 MHz) of **A12**.

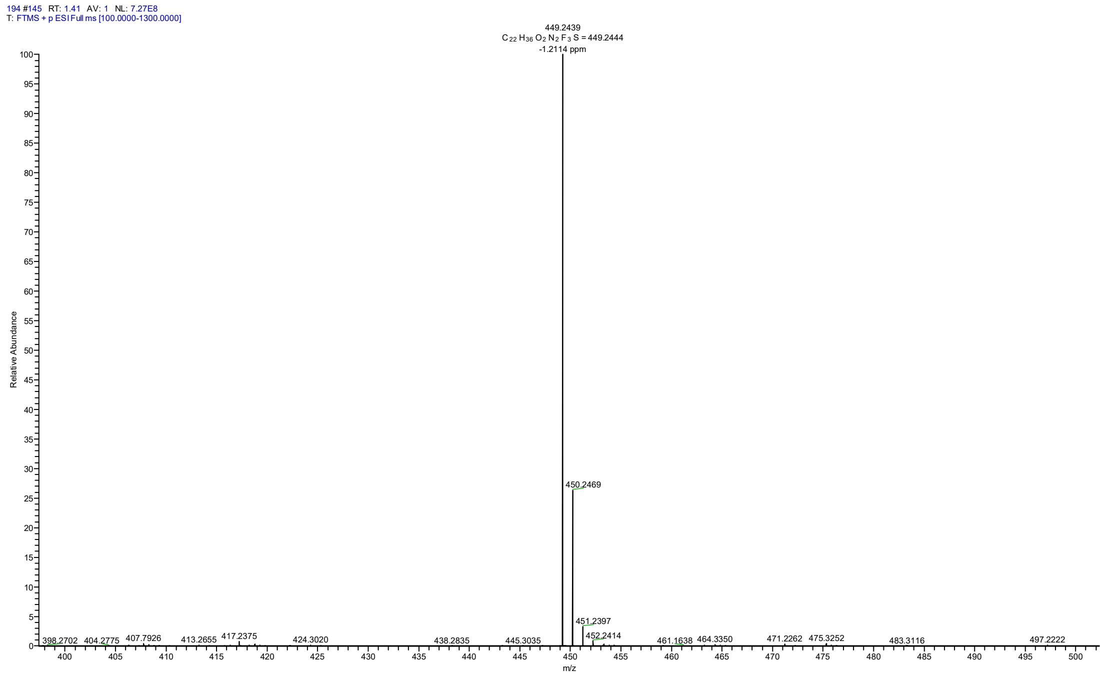

Figure S45. HRMS spectrum of **A12**.

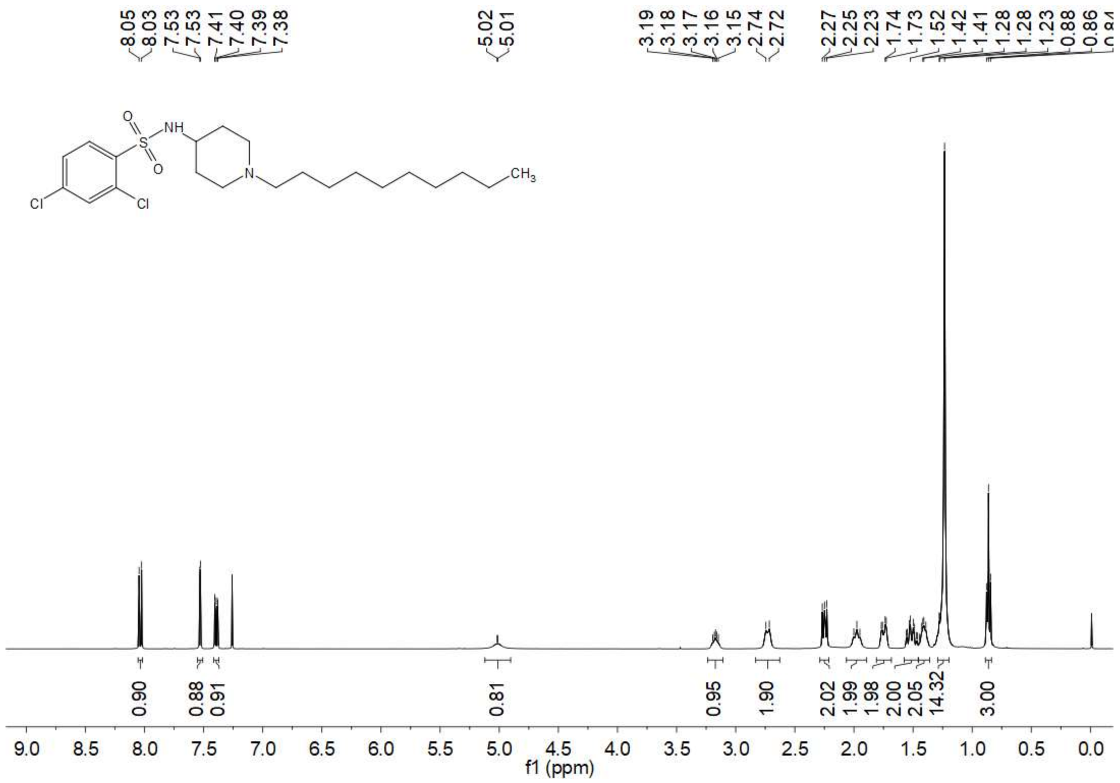

Figure S46. ^{1}H NMR spectrum ($CDCl_3$, 400 MHz) of $\mathbf{A_{13}}$.

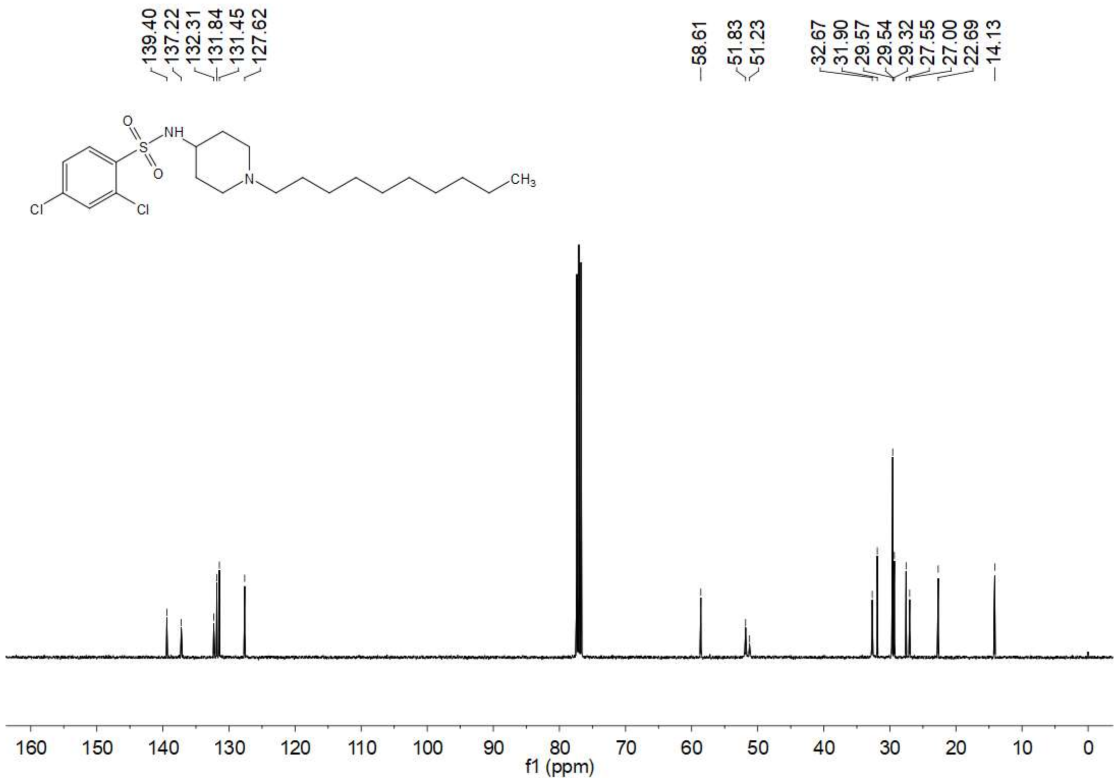

Figure S47. ^{13}C NMR spectrum ($CDCl_3$, 101 MHz) of $\mathbf{A_{13}}$.

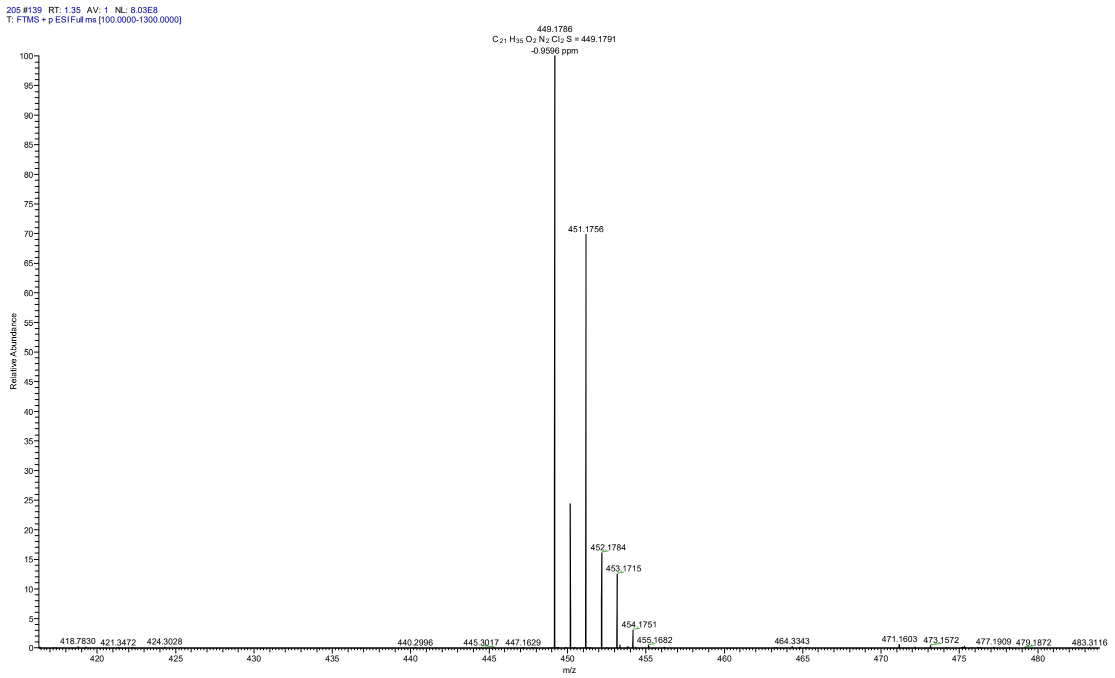

Figure S48. HRMS spectrum of **A13**.

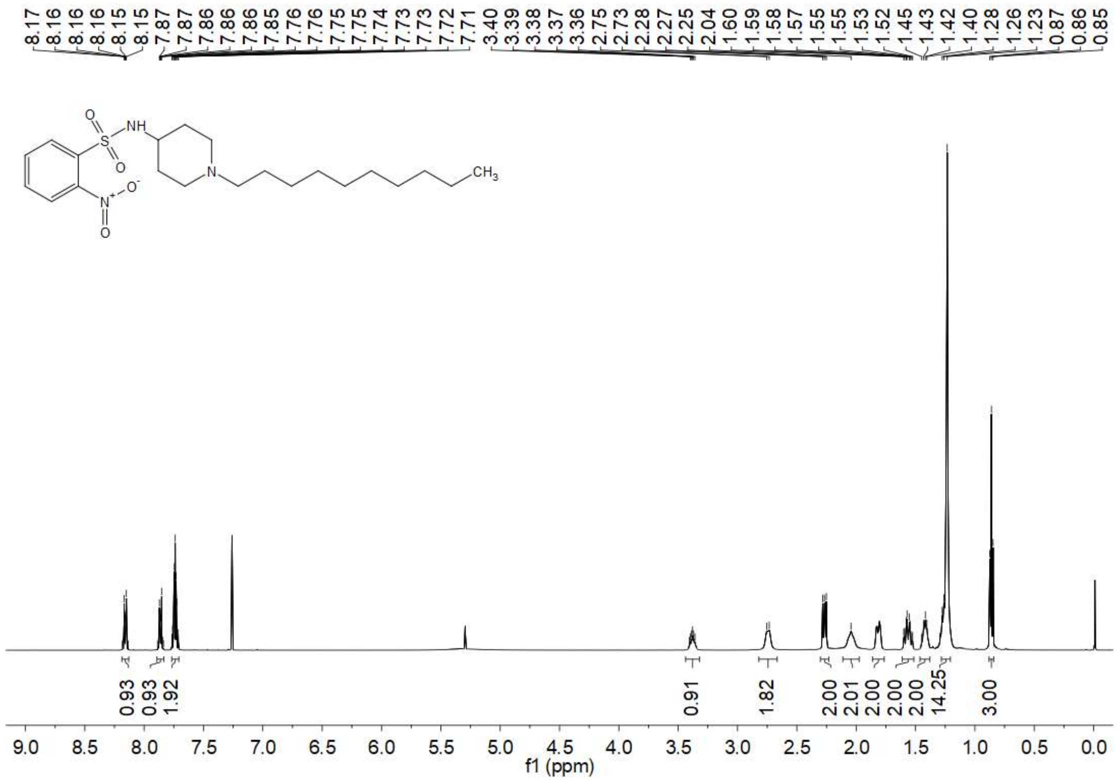

Figure S49. ^{1}H NMR spectrum ($CDCl_3$, 500 MHz) of **A14**.

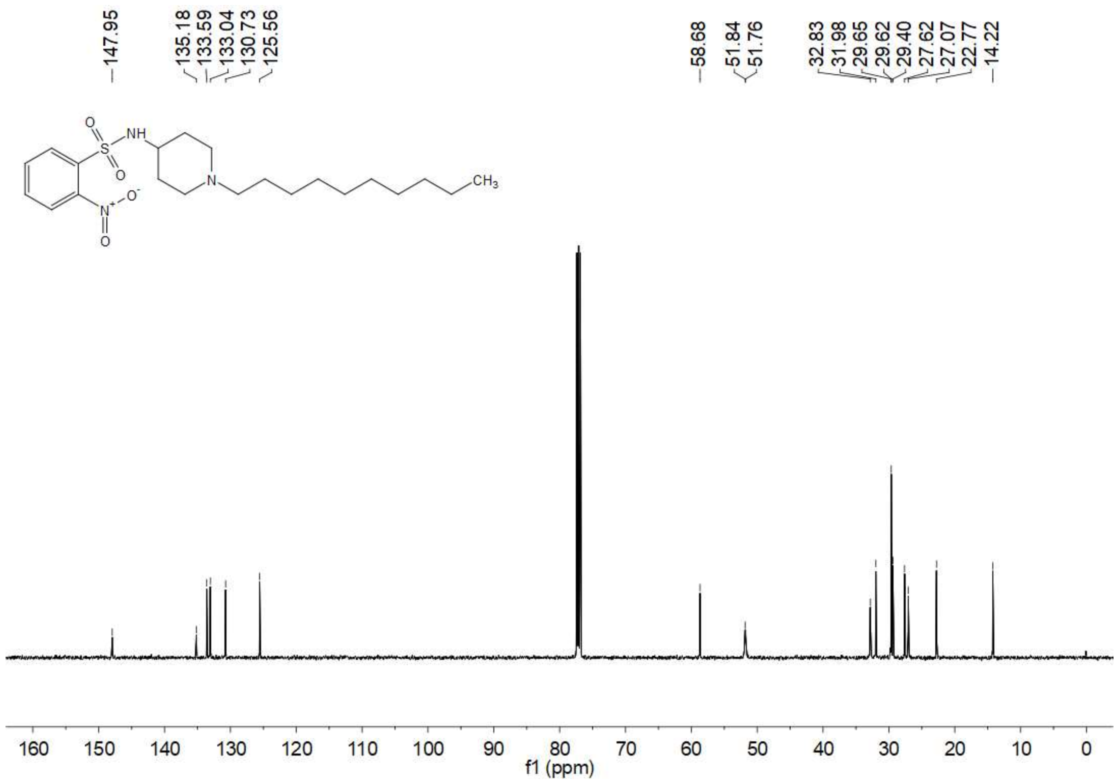

Figure S50. ^{13}C NMR spectrum ($CDCl_3$, 126 MHz) of **A14**.

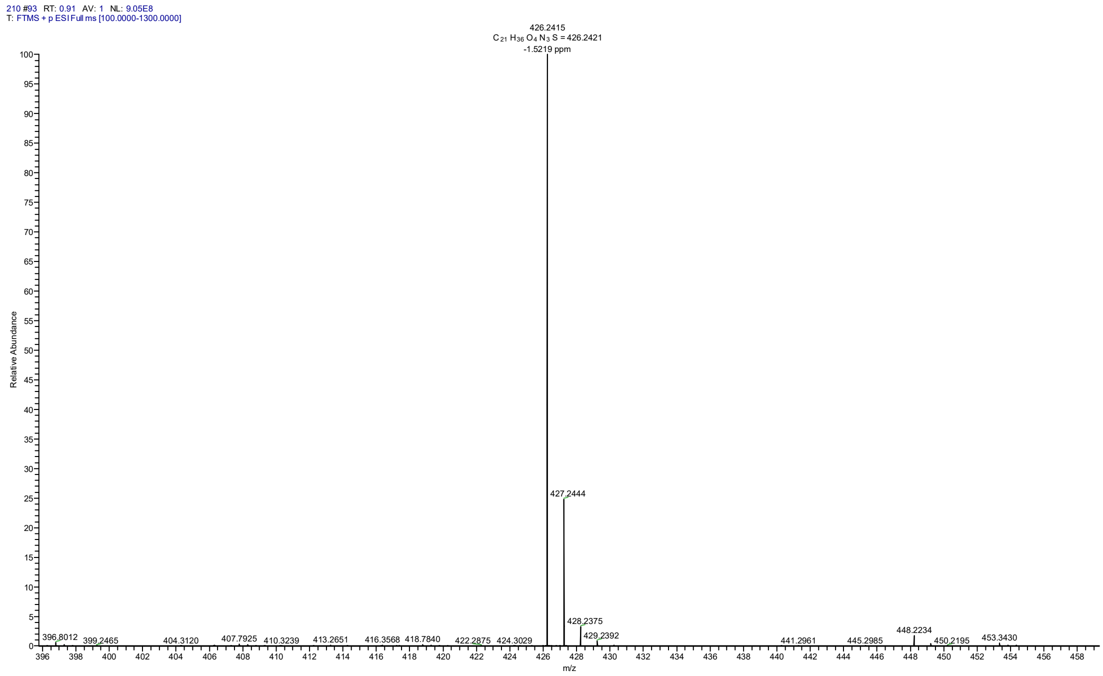

Figure S51. HRMS spectrum of **A14**.

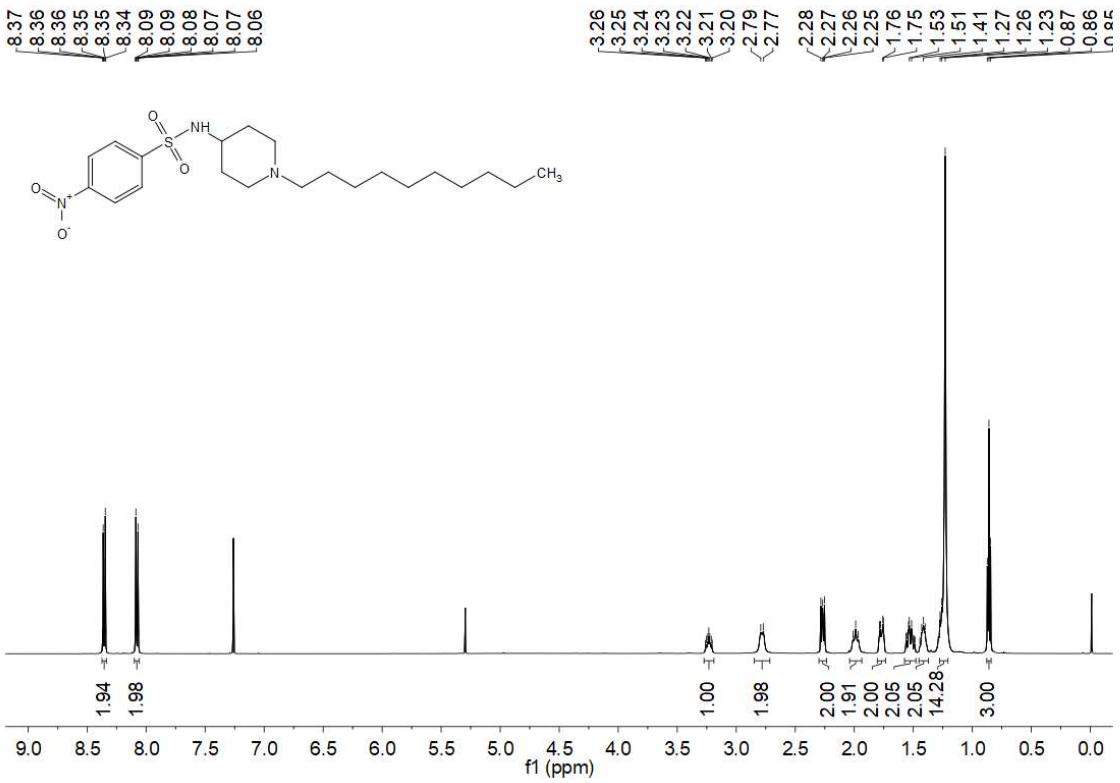

Figure S52. ^{1}H NMR spectrum ($CDCl_3$, 500 MHz) of **A_{15}**.

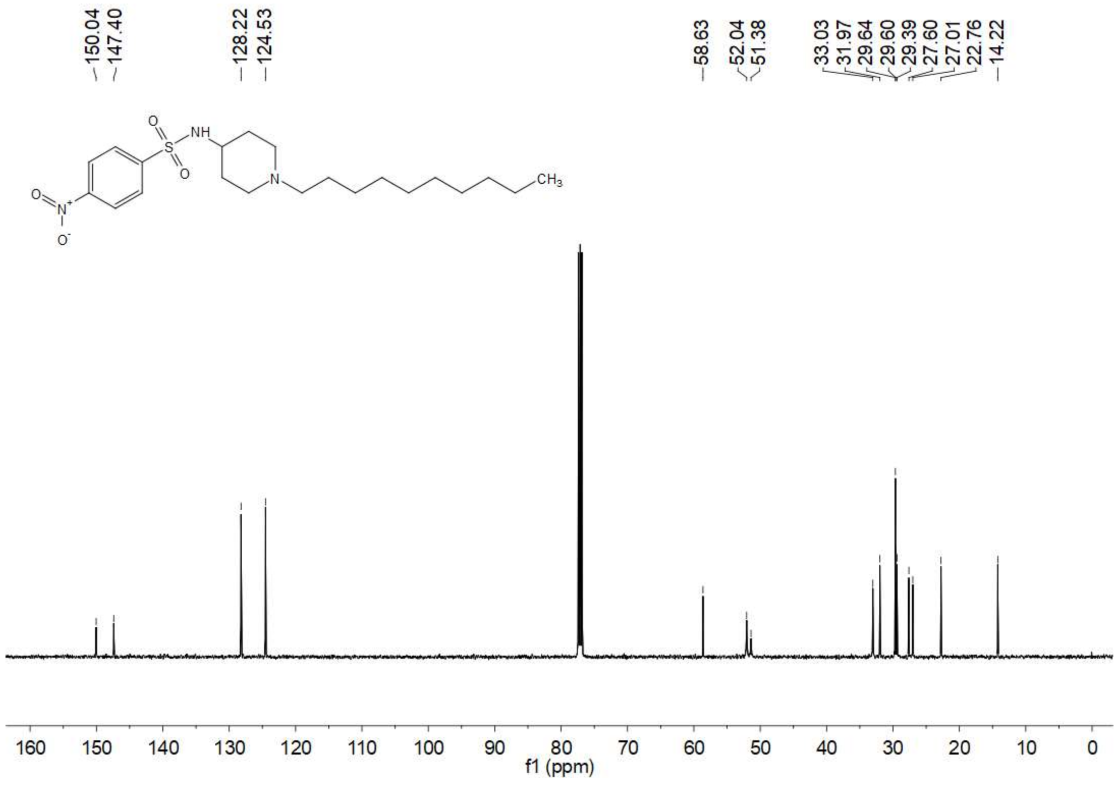

Figure S53. ^{13}C NMR spectrum ($CDCl_3$, 126 MHz) of **A_{15}**.

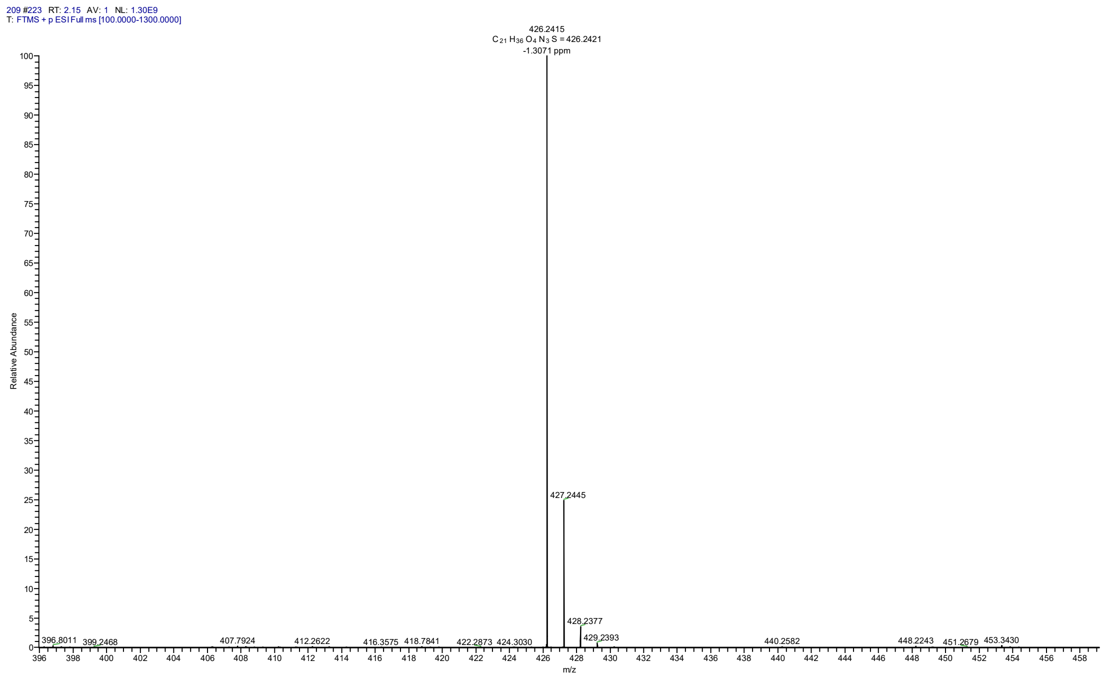

Figure S54. HRMS spectrum of **A15**.

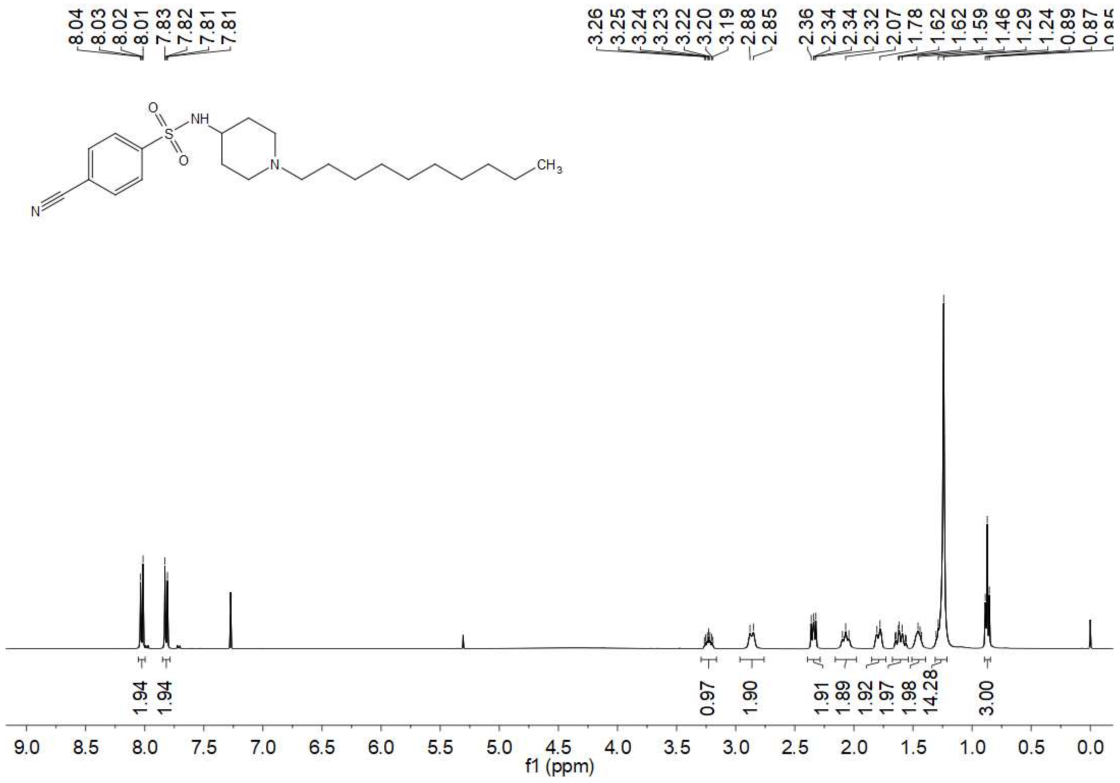

Figure S55. ^{1}H NMR spectrum ($CDCl_3$, 400 MHz) of **A16**.

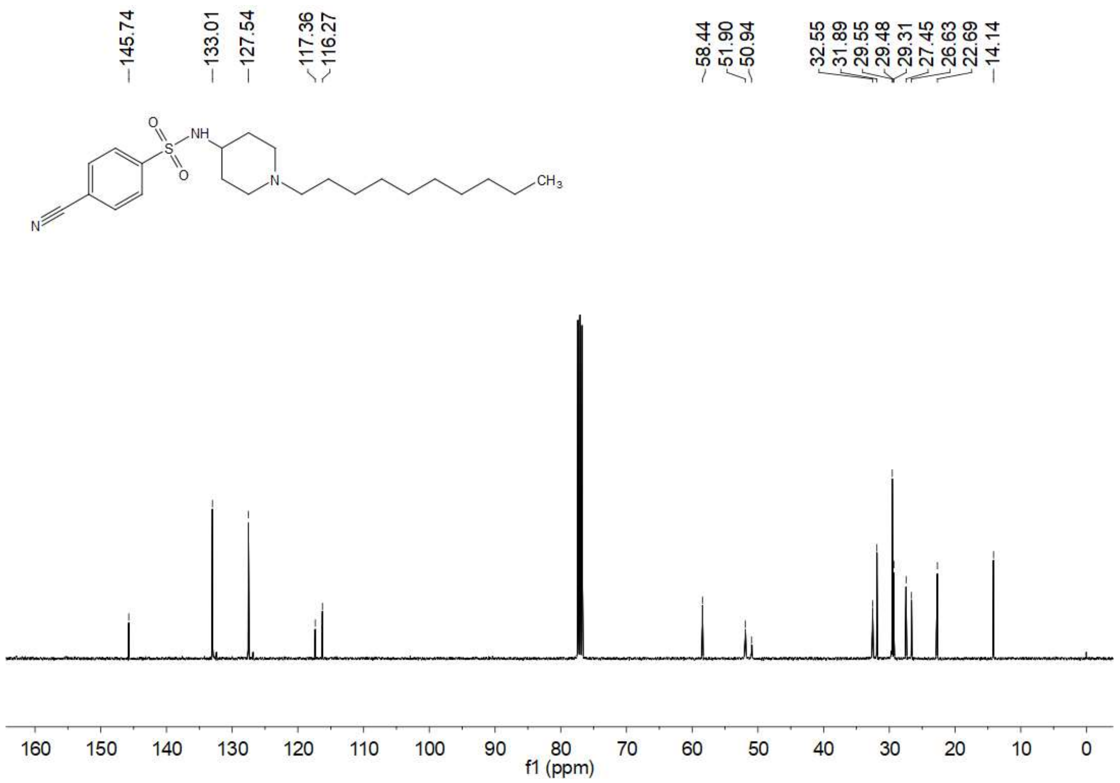

Figure S56. ^{13}C NMR spectrum ($CDCl_3$, 101 MHz) of $\mathbf{A_{16}}$.

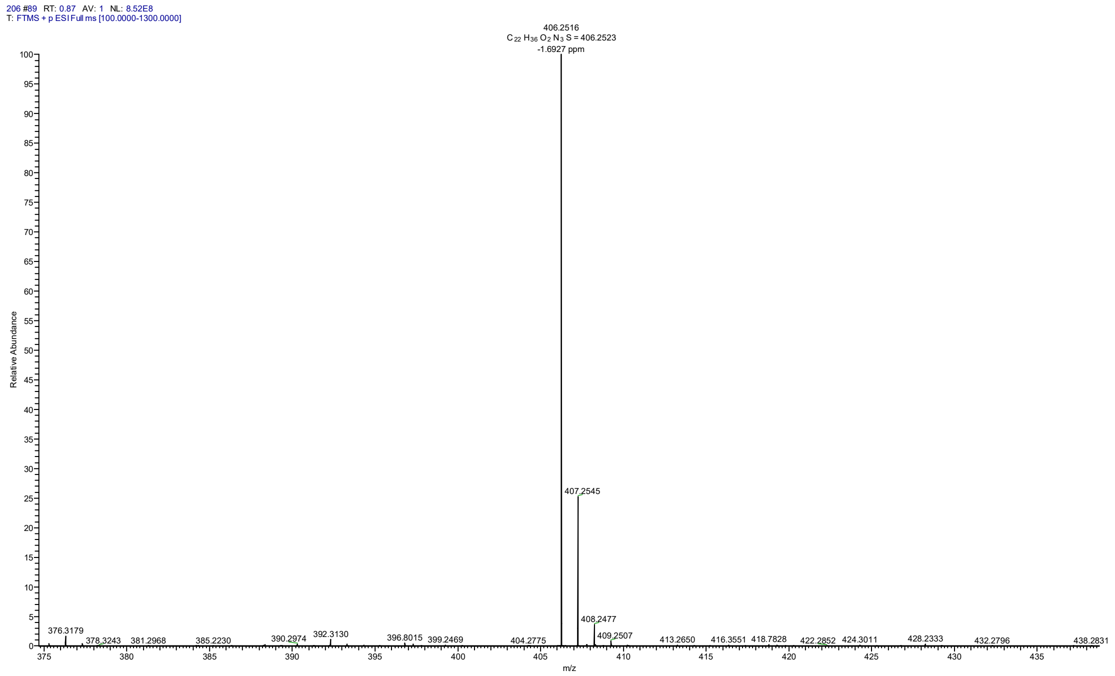

Figure S57. HRMS spectrum of $\mathbf{A_{16}}$.

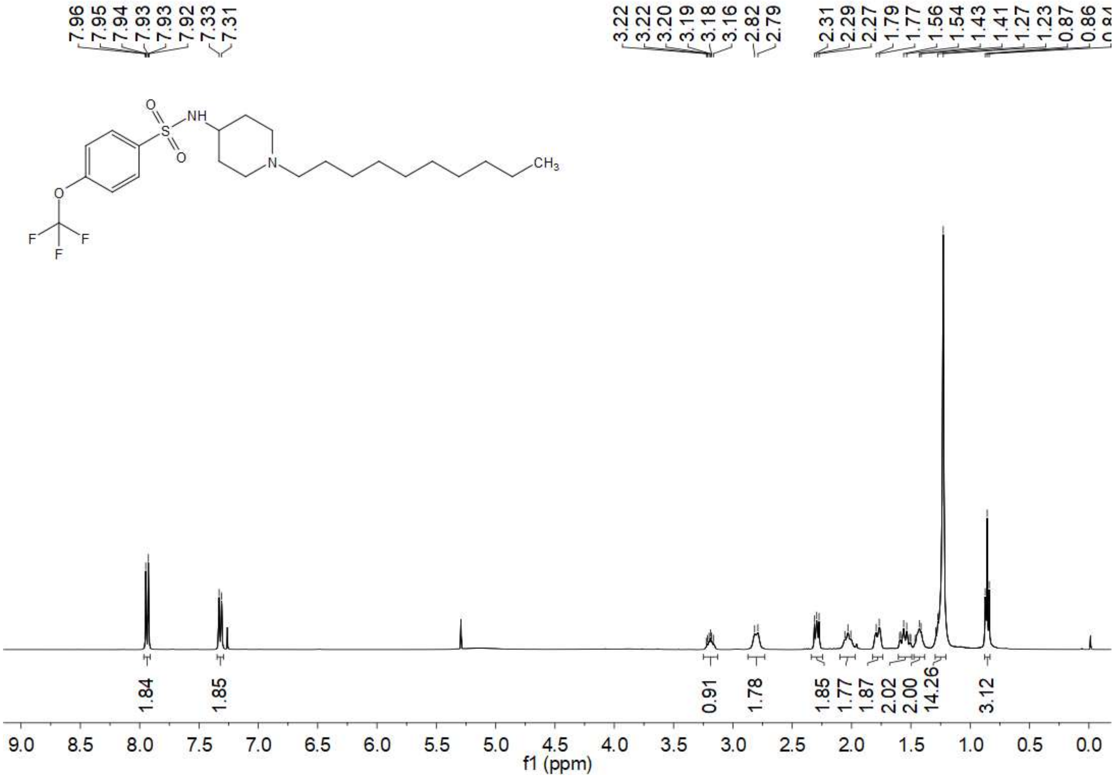

Figure S58. ^{1}H NMR spectrum ($CDCl_3$, 400 MHz) of $\mathbf{A_{17}}$.

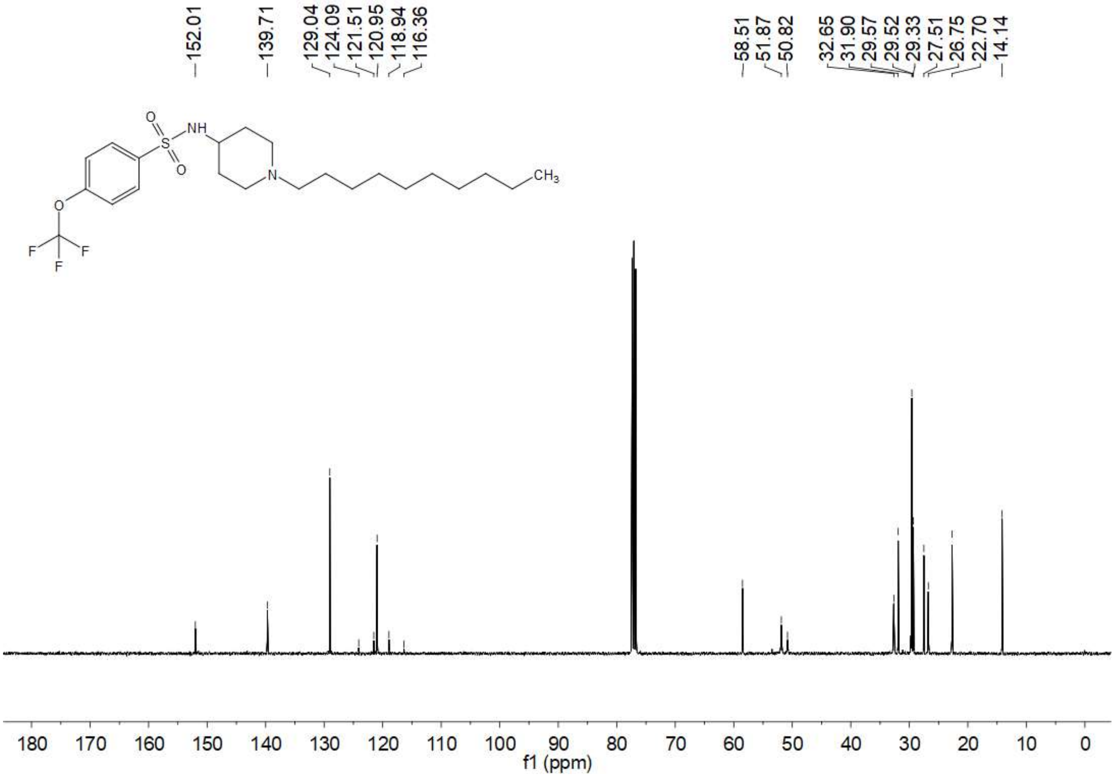

Figure S59. ^{13}C NMR spectrum ($CDCl_3$, 101 MHz) of $\mathbf{A_{17}}$.

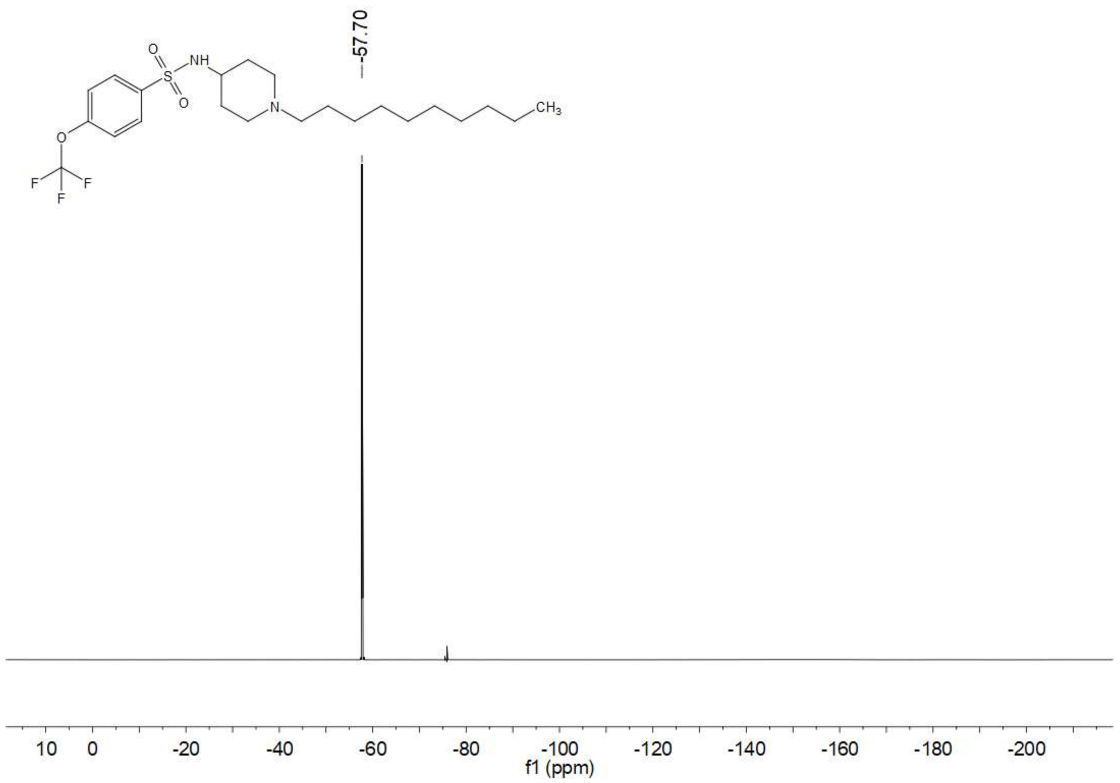

Figure S60. ^{19}F NMR spectrum ($CDCl_3$, 376 MHz) of **A17**.

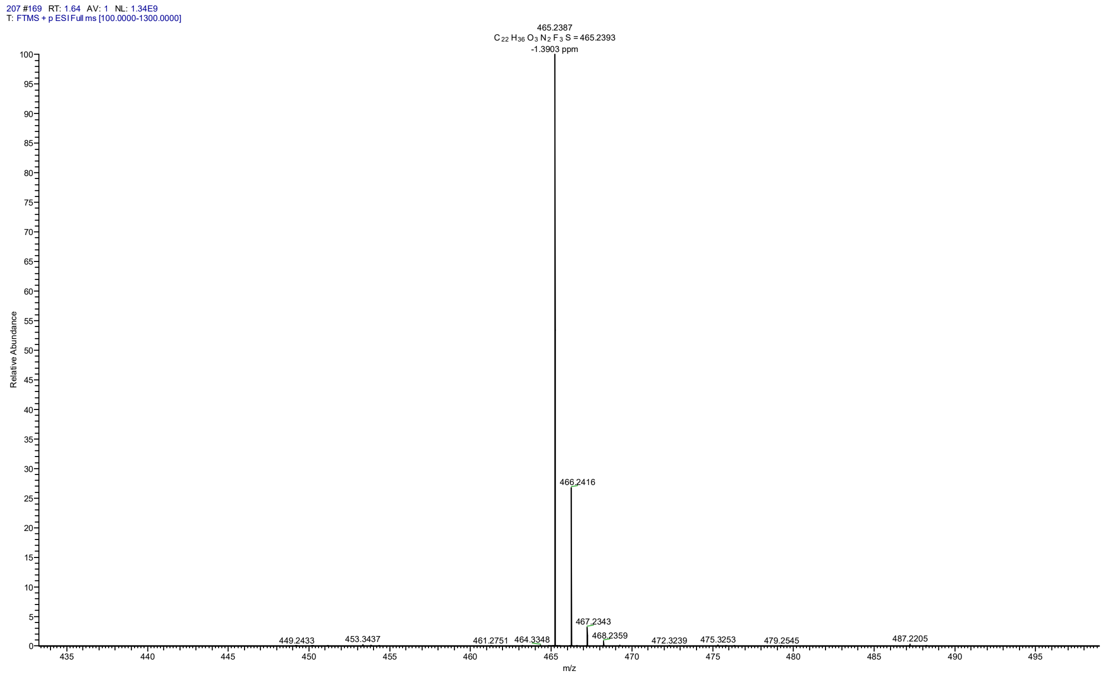

Figure S61. HRMS spectrum of **A17**.

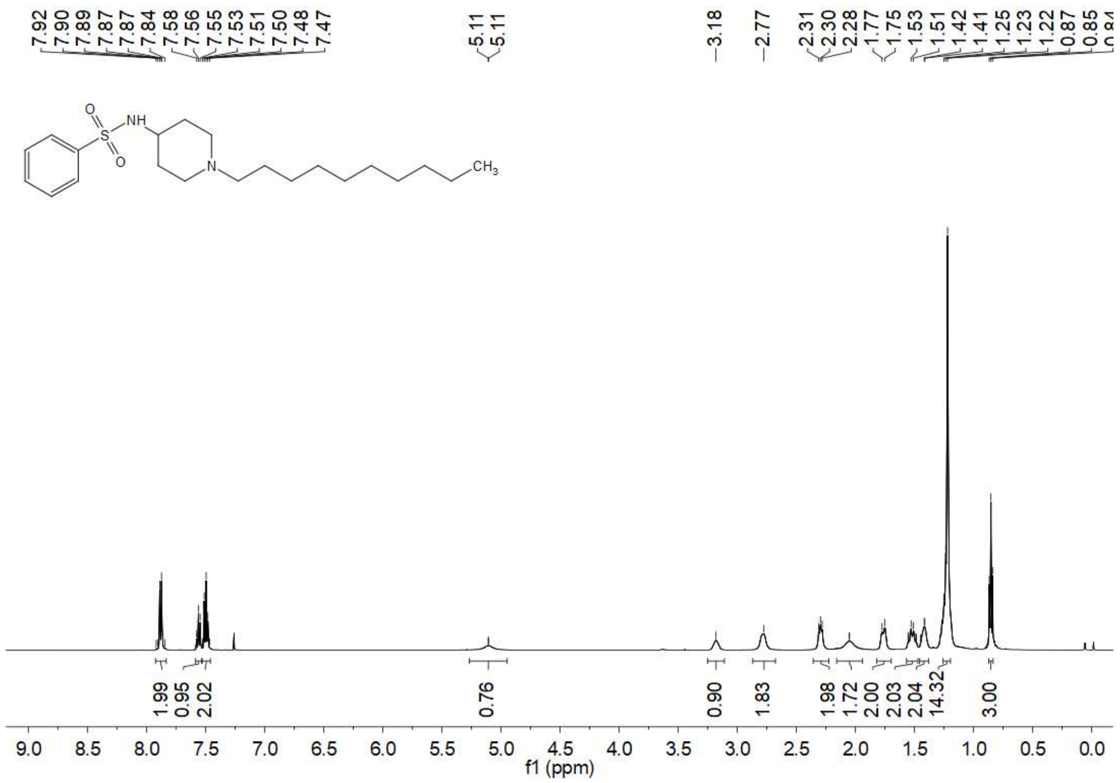

Figure S62. ^{1}H NMR spectrum ($CDCl_3$, 500 MHz) of $\mathbf{A_{18}}$.

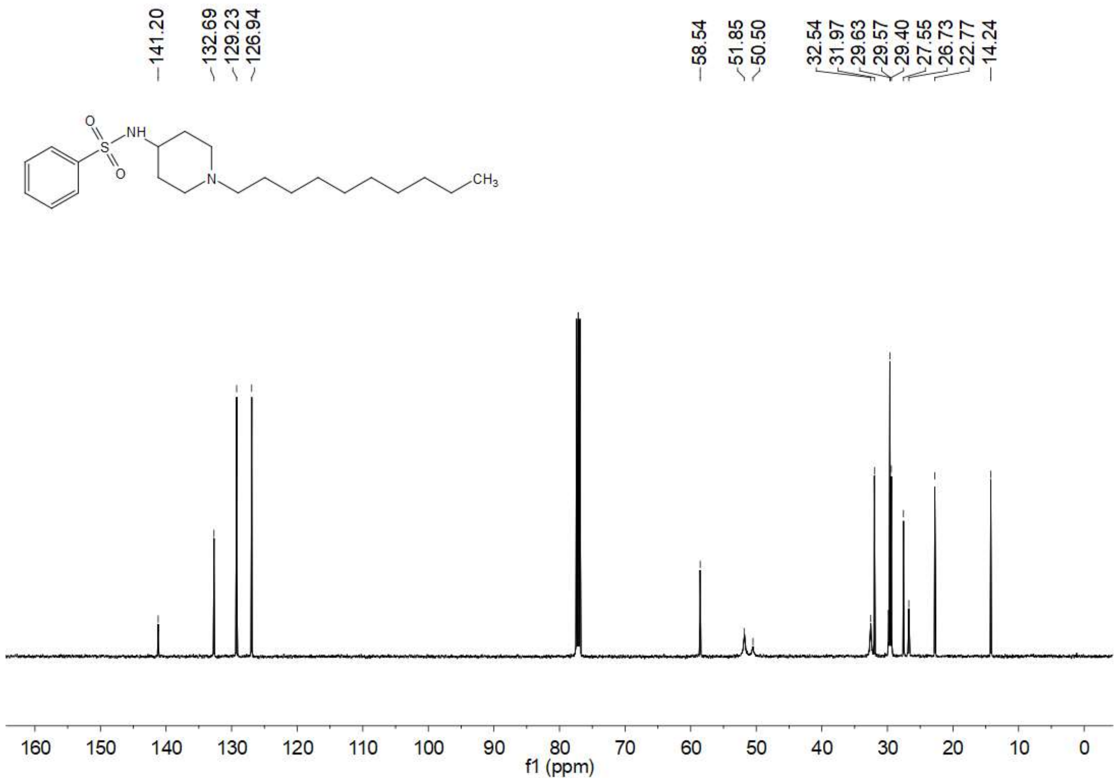

Figure S63. ^{13}C NMR spectrum ($CDCl_3$, 126 MHz) of $\mathbf{A_{18}}$.

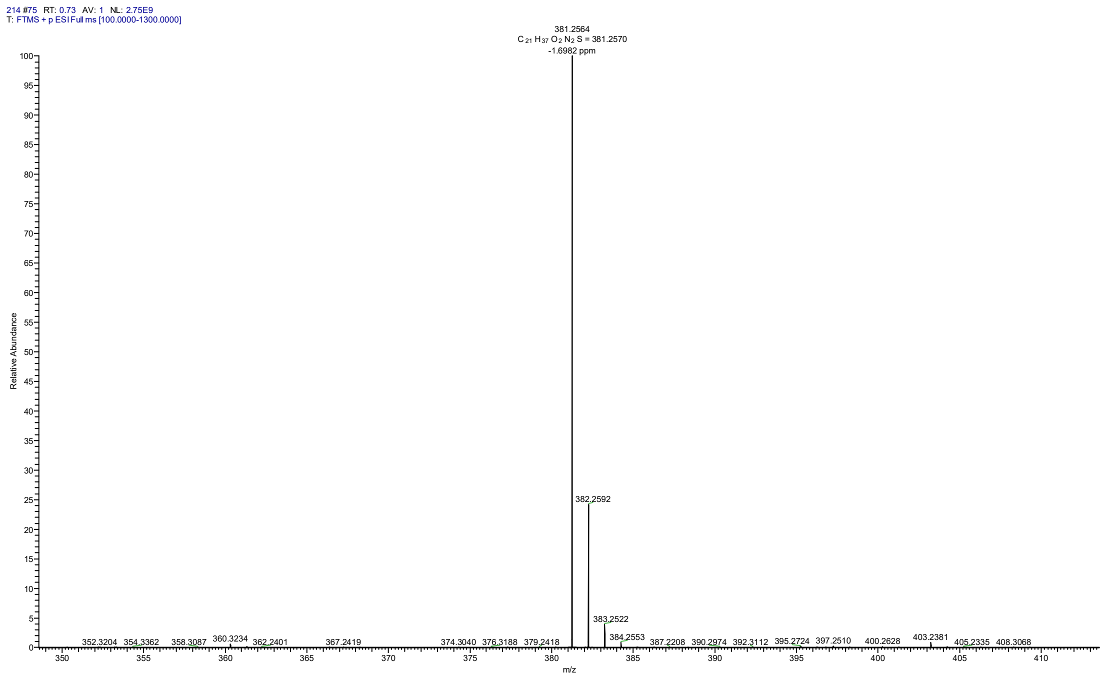

Figure S64. HRMS spectrum of $\mathbf{A_{18}}$.

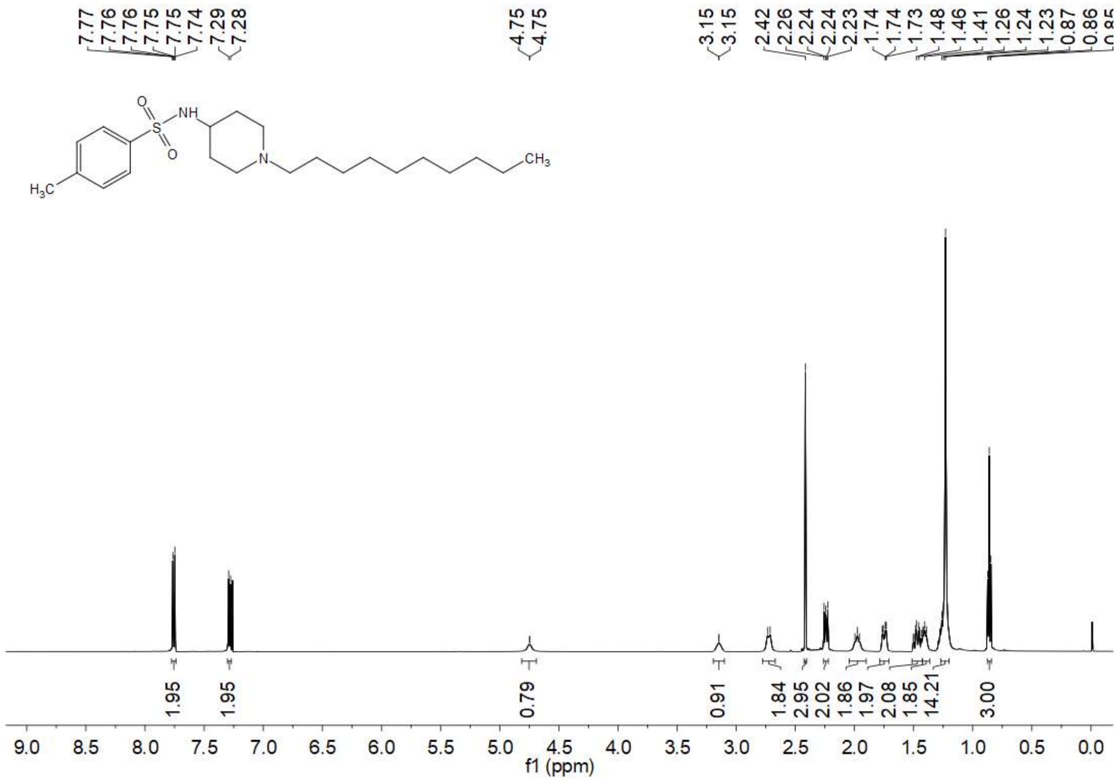

Figure S65. ^{1}H NMR spectrum ($CDCl_3$, 500 MHz) of $\mathbf{A_{19}}$.

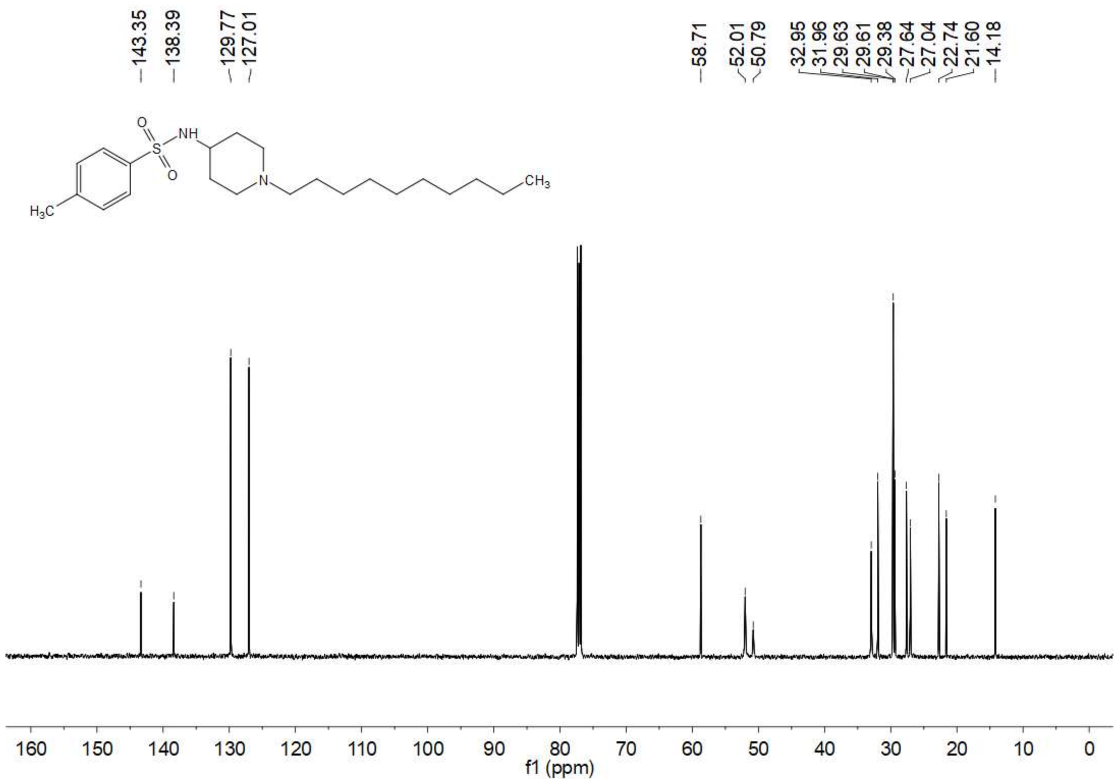

Figure S66. ^{13}C NMR spectrum ($CDCl_3$, 126 MHz) of **A19**.

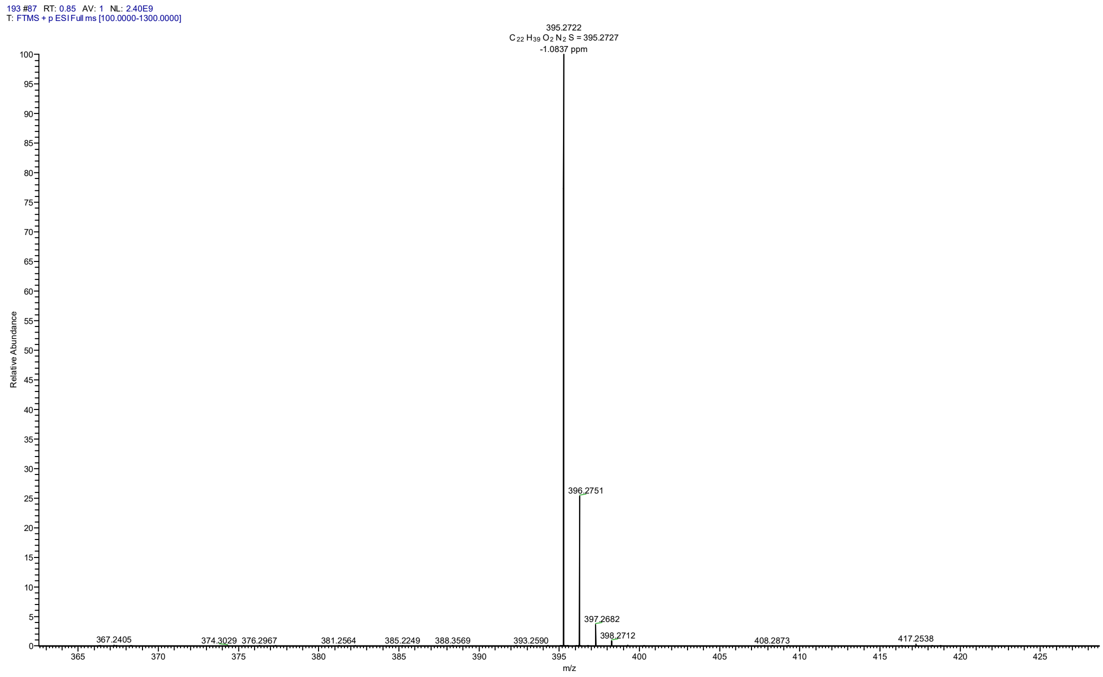

Figure S67. HRMS spectrum of **A19**.

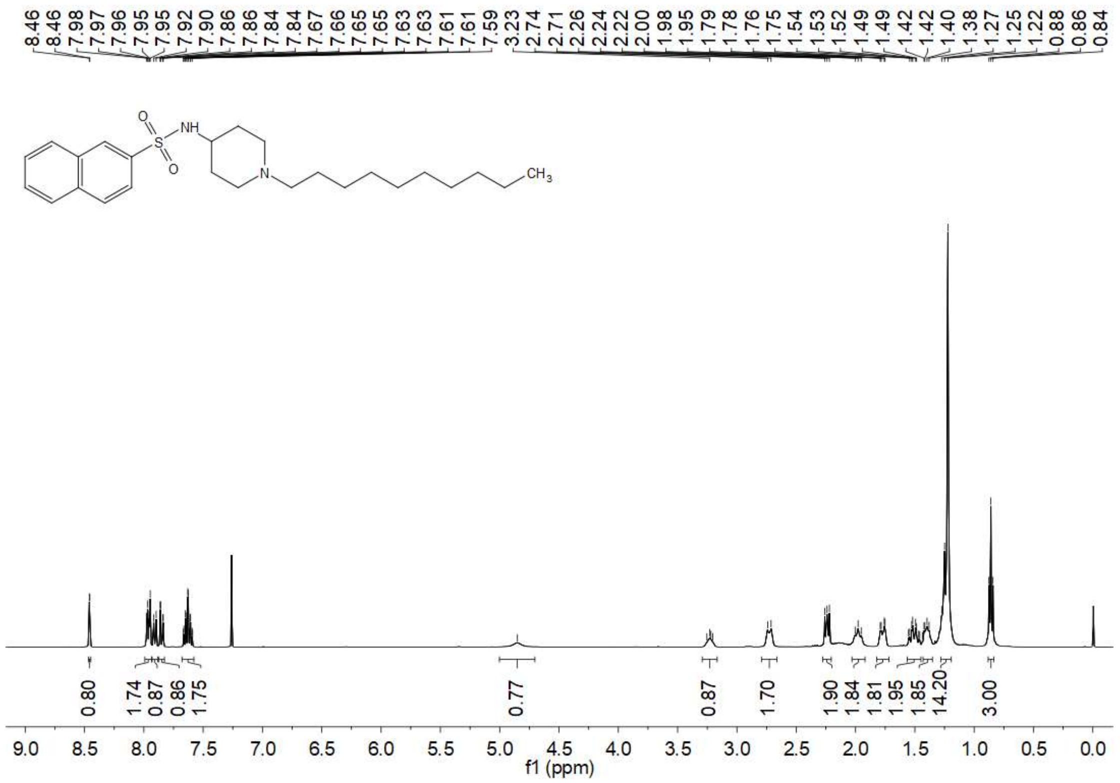

Figure S68. ^{1}H NMR spectrum ($CDCl_3$, 400 MHz) of **A_{20}**.

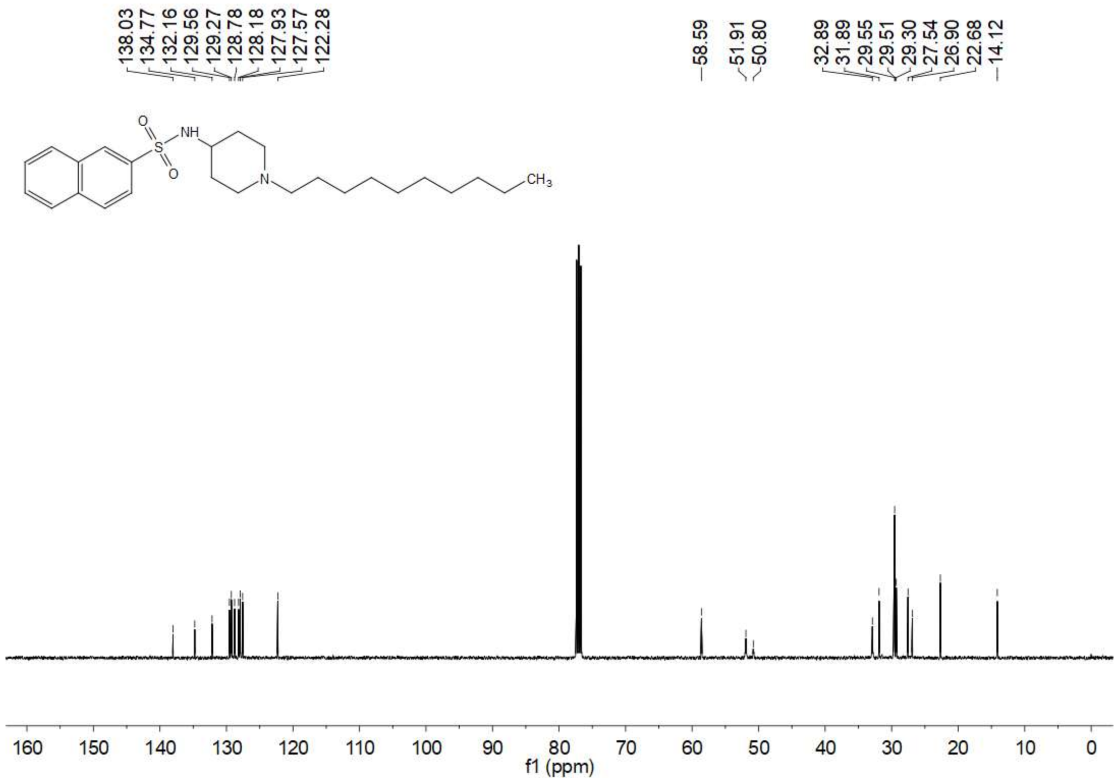

Figure S69. ^{13}C NMR spectrum ($CDCl_3$, 101 MHz) of **A_{20}**.

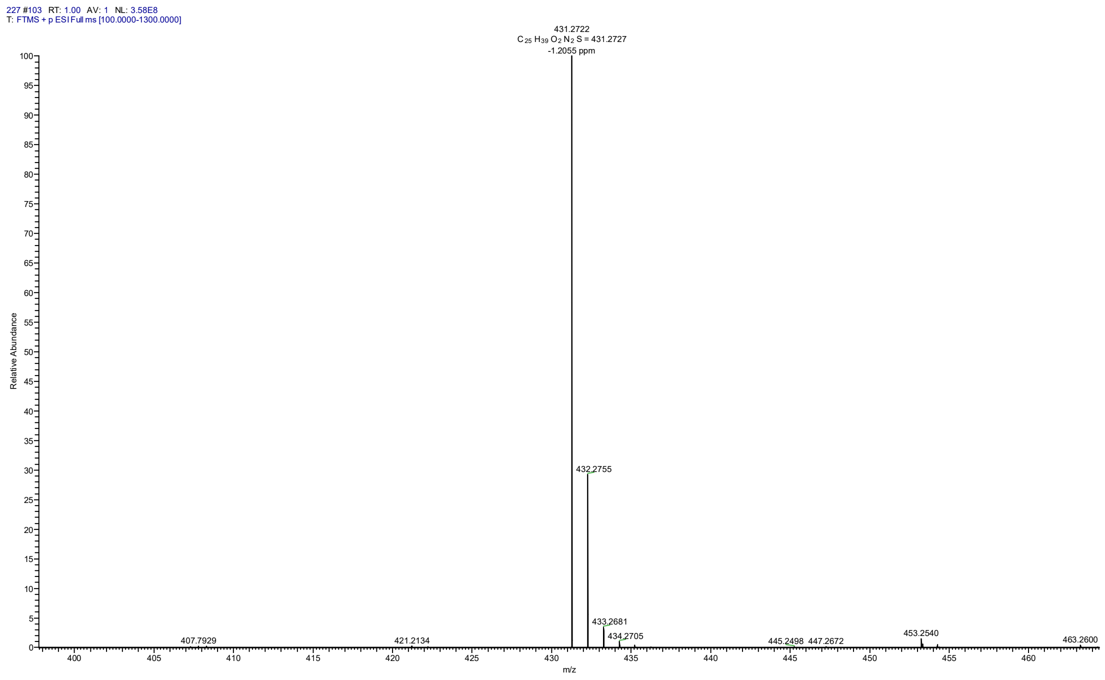

Figure S70. HRMS spectrum of **A20**.

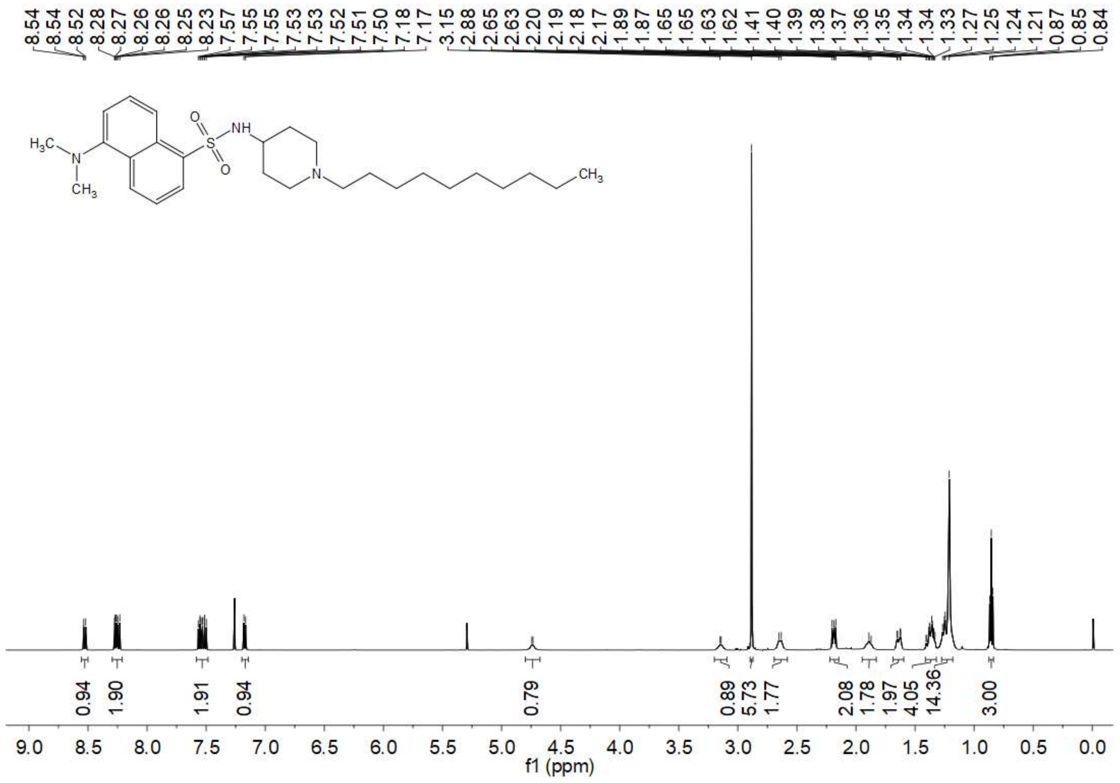

Figure S71. ^{1}H NMR spectrum ($CDCl_3$, 500 MHz) of **A21**.

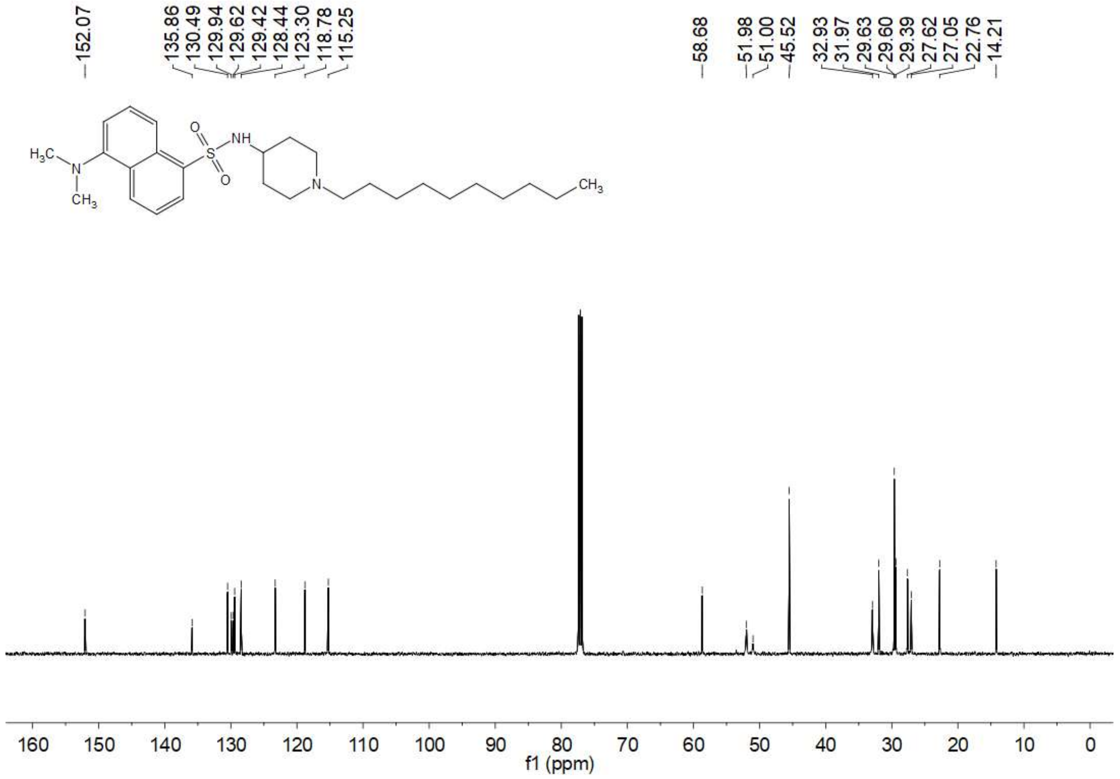

Figure S72. ^{13}C NMR spectrum ($CDCl_3$, 126 MHz) of **A21**.

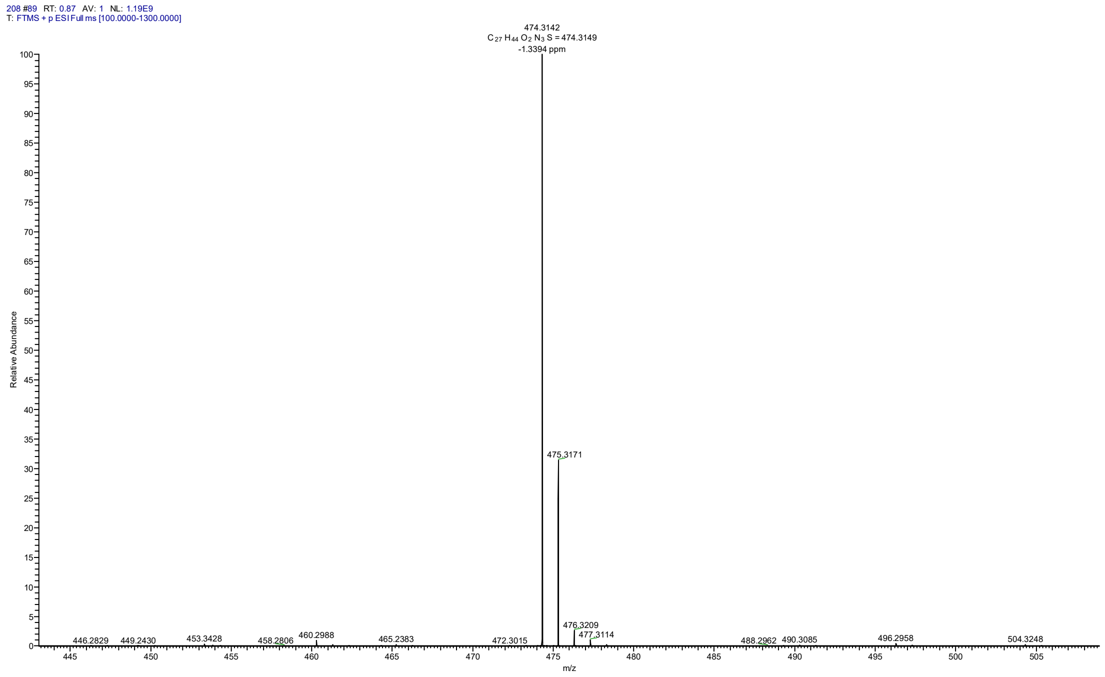

Figure S73. HRMS spectrum of **A21**.

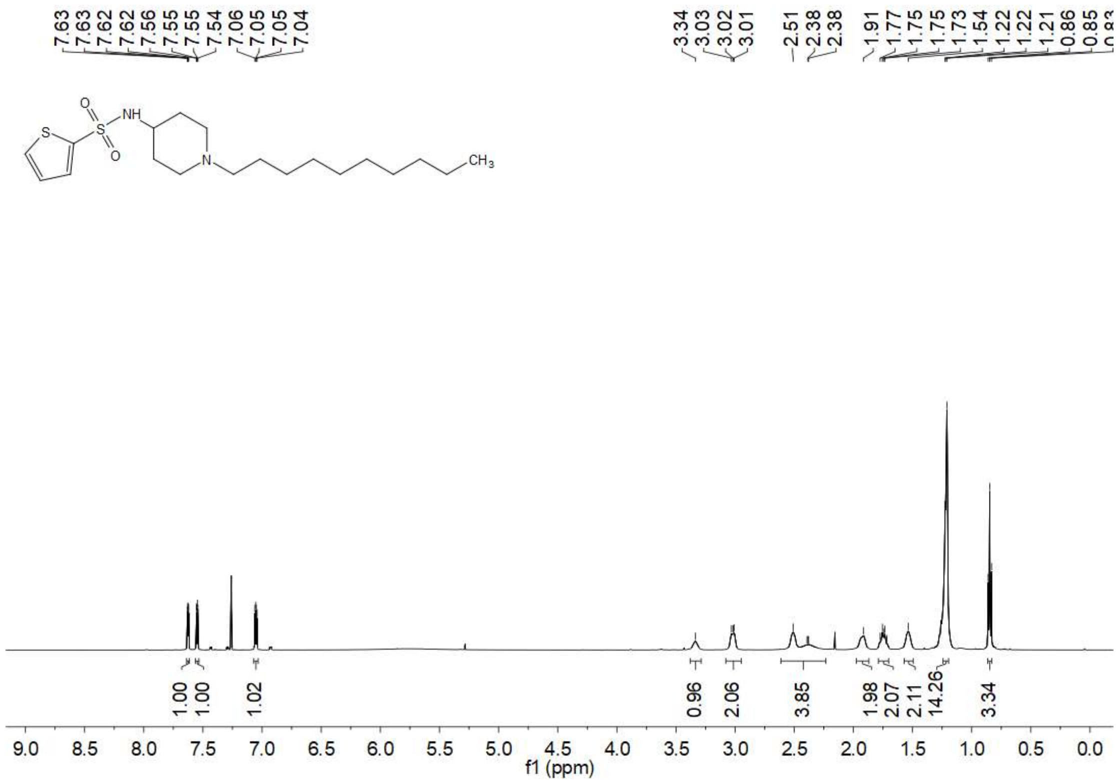

Figure S74. ^{1}H NMR spectrum ($CDCl_3$, 500 MHz) of **A$_{22}$**.

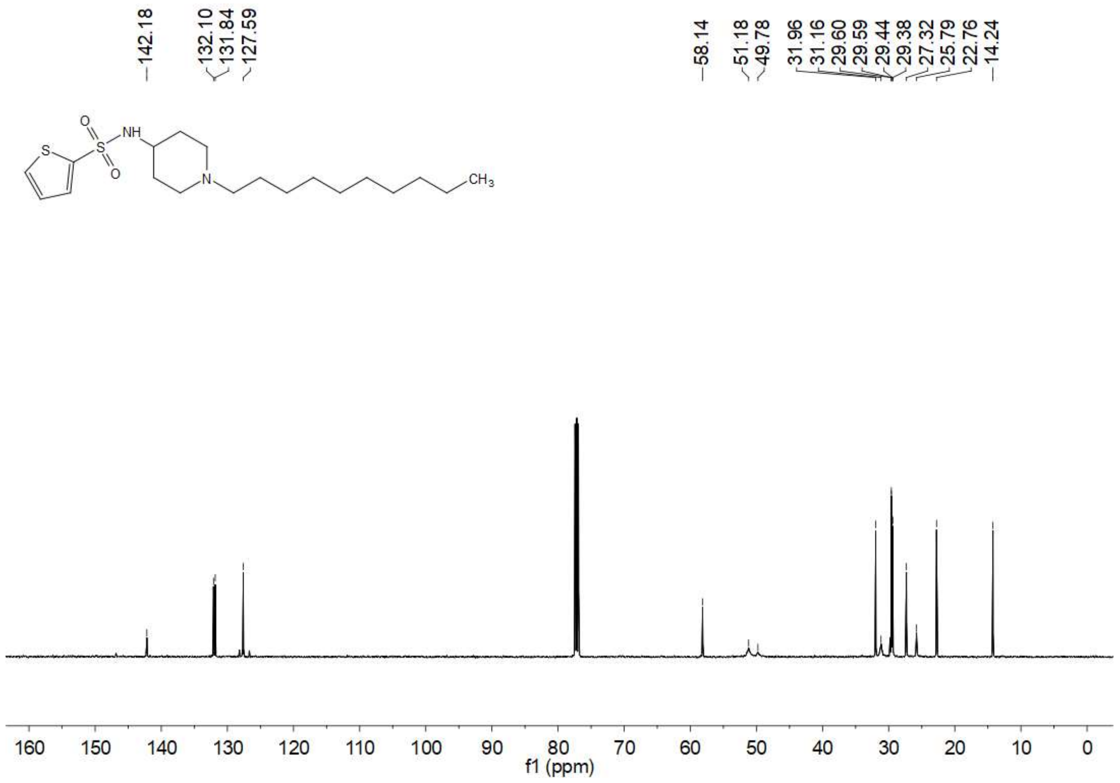

Figure S75. ^{13}C NMR spectrum ($CDCl_3$, 126 MHz) of **A$_{22}$**.

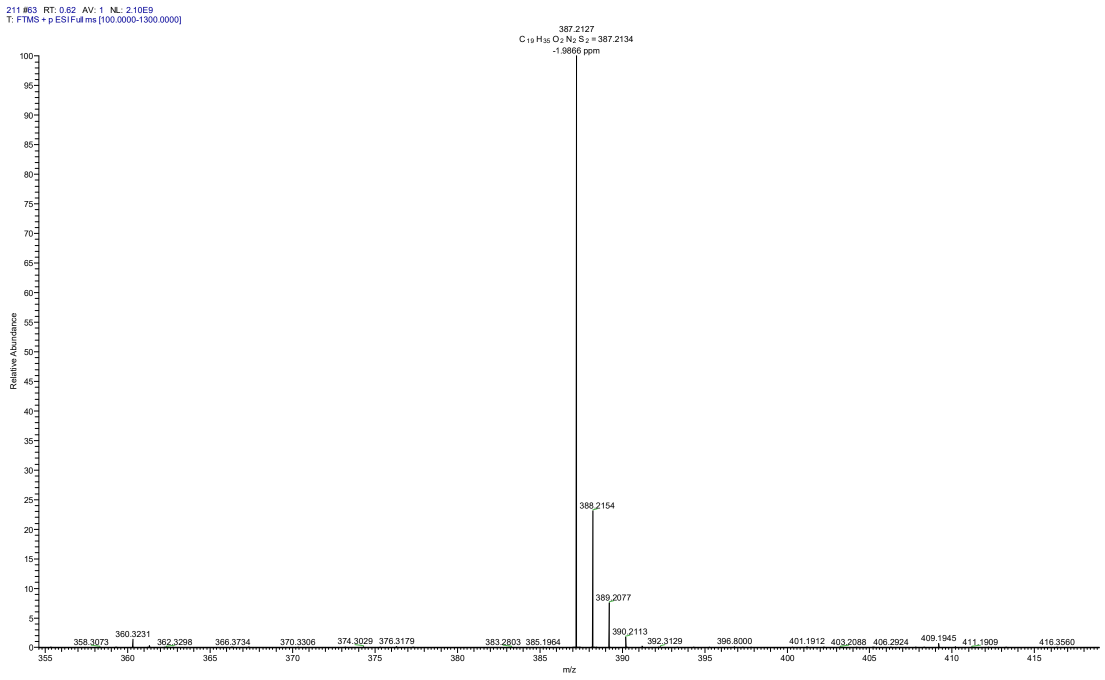

Figure S76. HRMS spectrum of **A22**.

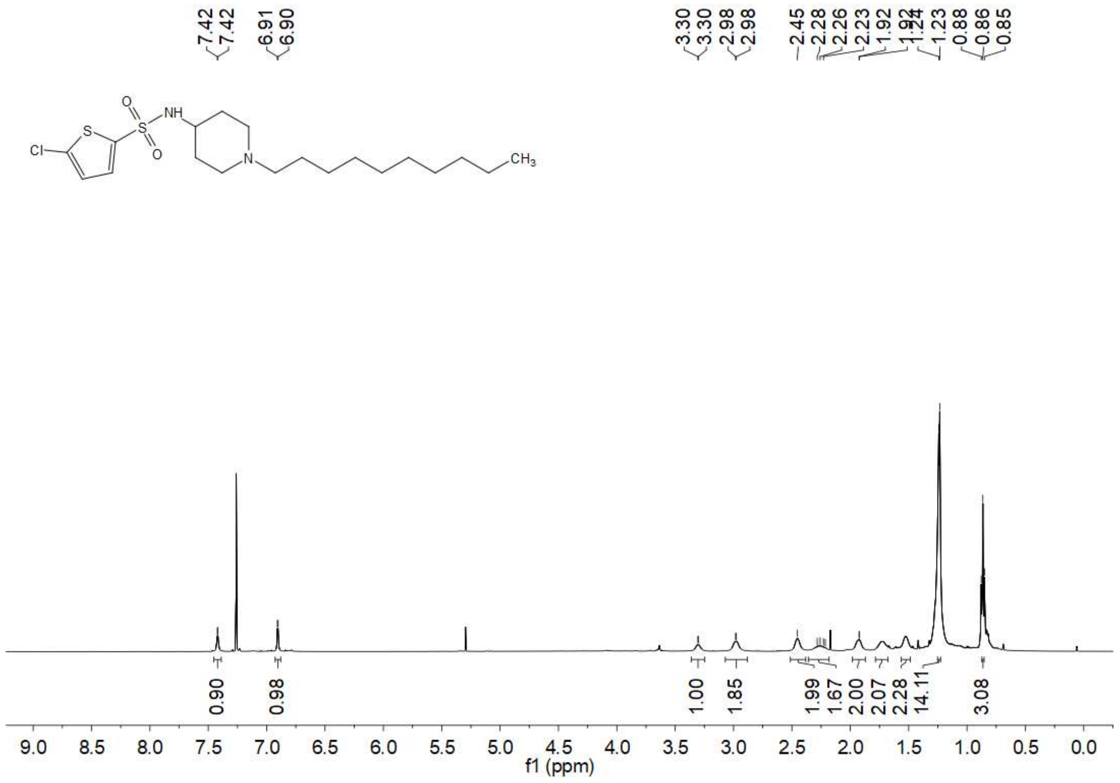

Figure S77. ^{1}H NMR spectrum ($CDCl_3$, 500 MHz) of **A23**.

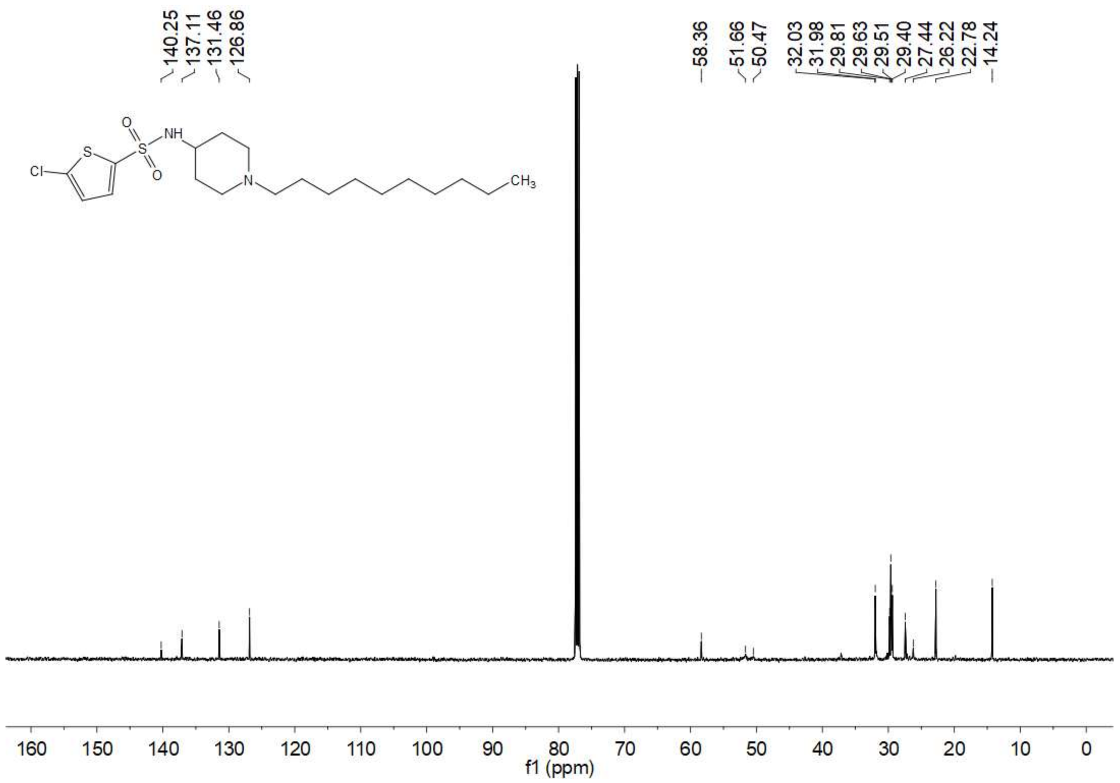

Figure S78. ^{13}C NMR spectrum ($CDCl_3$, 126 MHz) of **A23**.

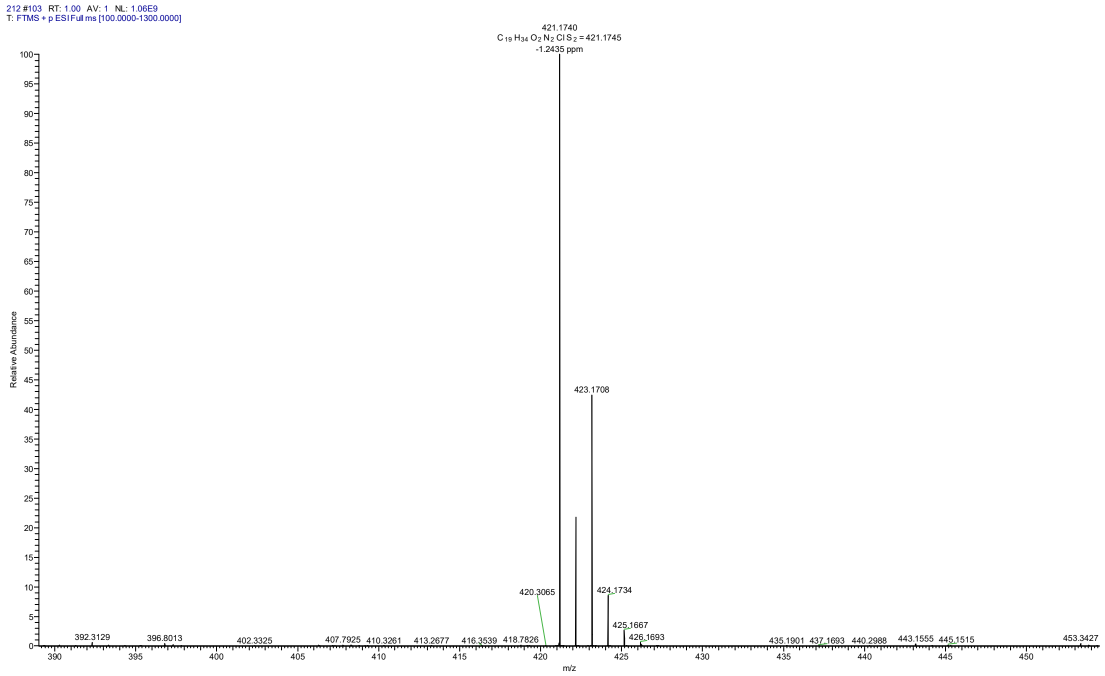

Figure S79. HRMS spectrum of **A23**.

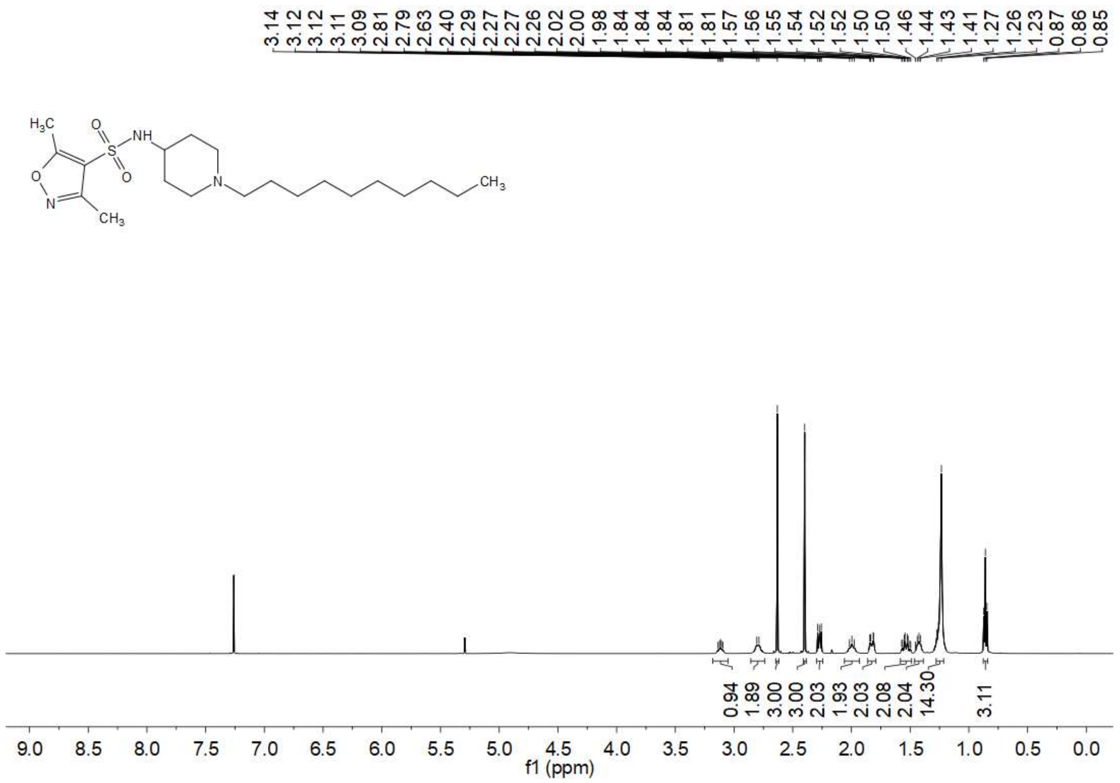

Figure S80. ^{1}H NMR spectrum ($CDCl_3$, 500 MHz) of **A_{24}**.

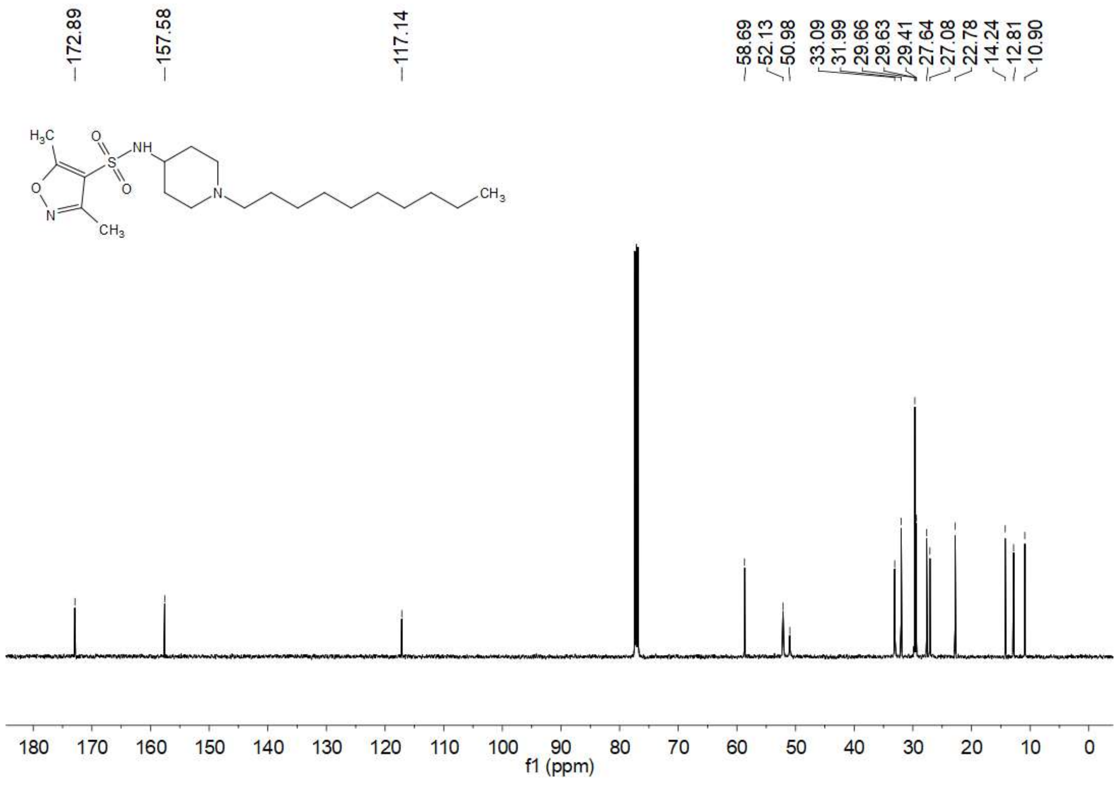

Figure S81. ^{13}C NMR spectrum ($CDCl_3$, 126 MHz) of **A_{24}**.

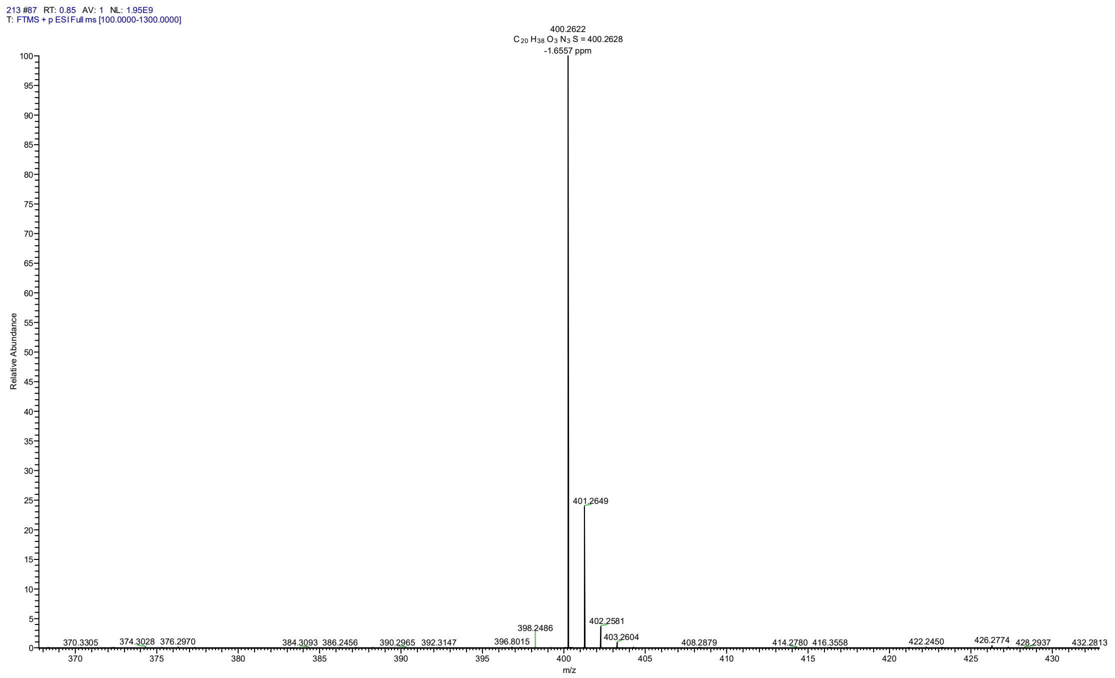

Figure S82. HRMS spectrum of $\mathbf{A_{24}}$.

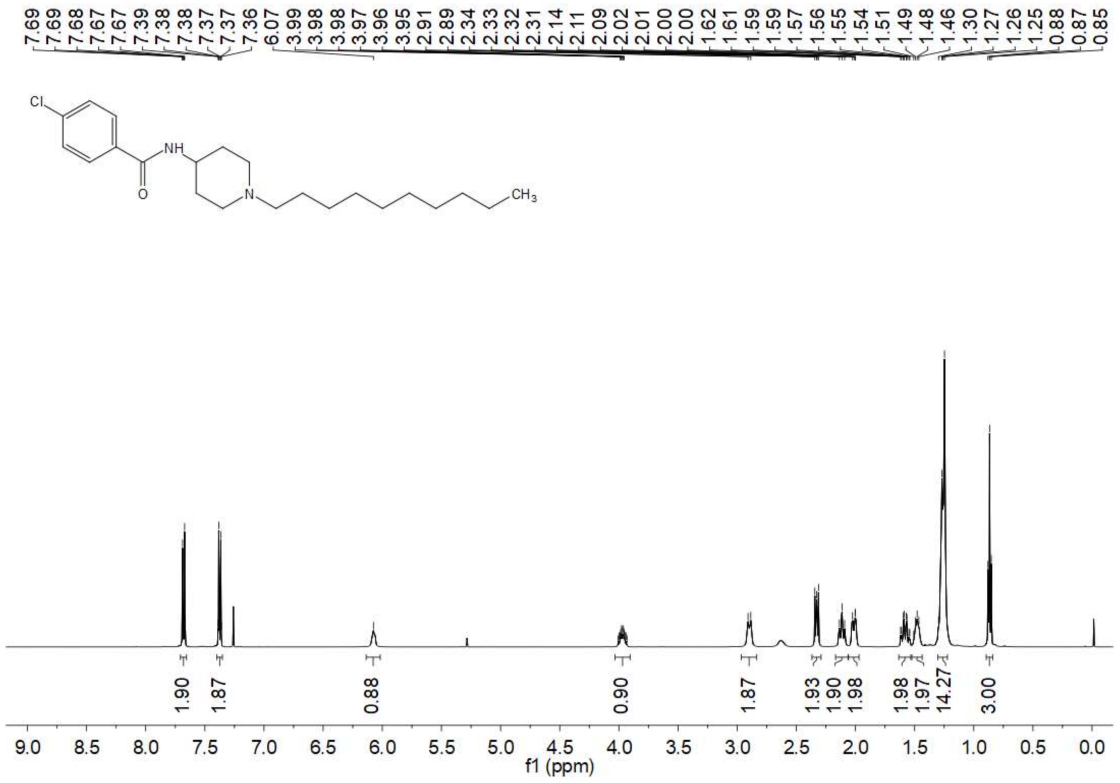

Figure S83. ^{1}H NMR spectrum ($CDCl_3$, 500 MHz) of $\mathbf{B_1}$.

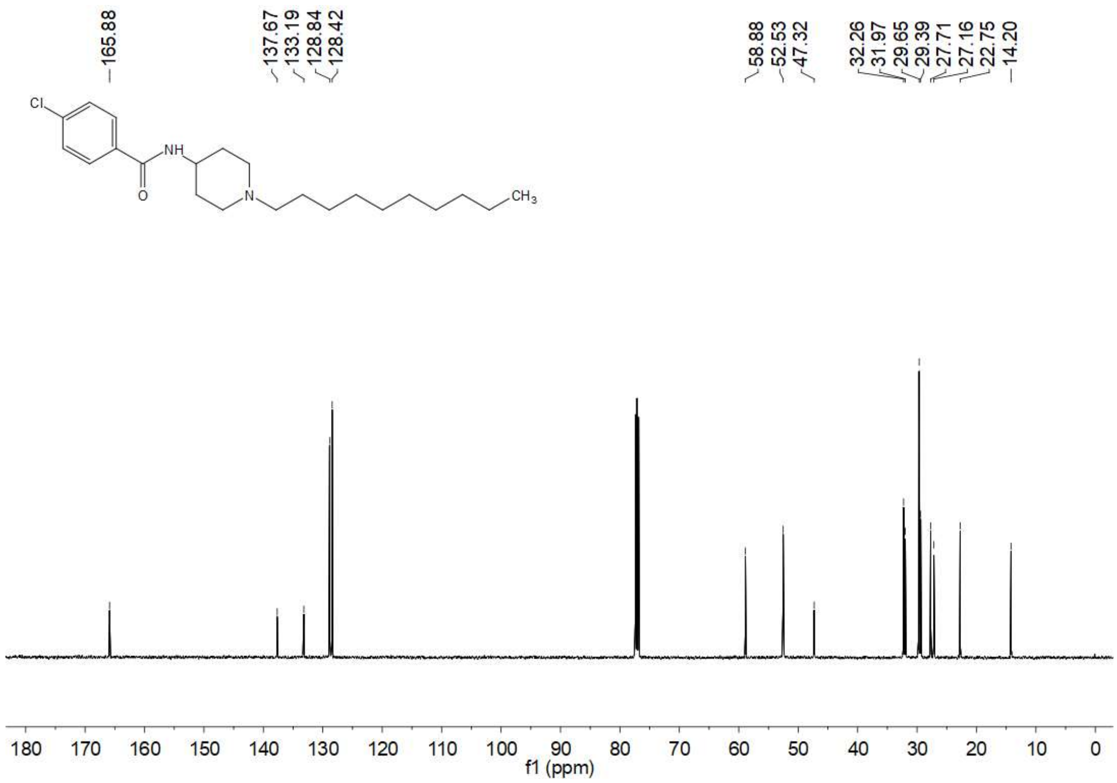

Figure S84. ^{13}C NMR spectrum ($CDCl_3$, 126 MHz) of **B1**.

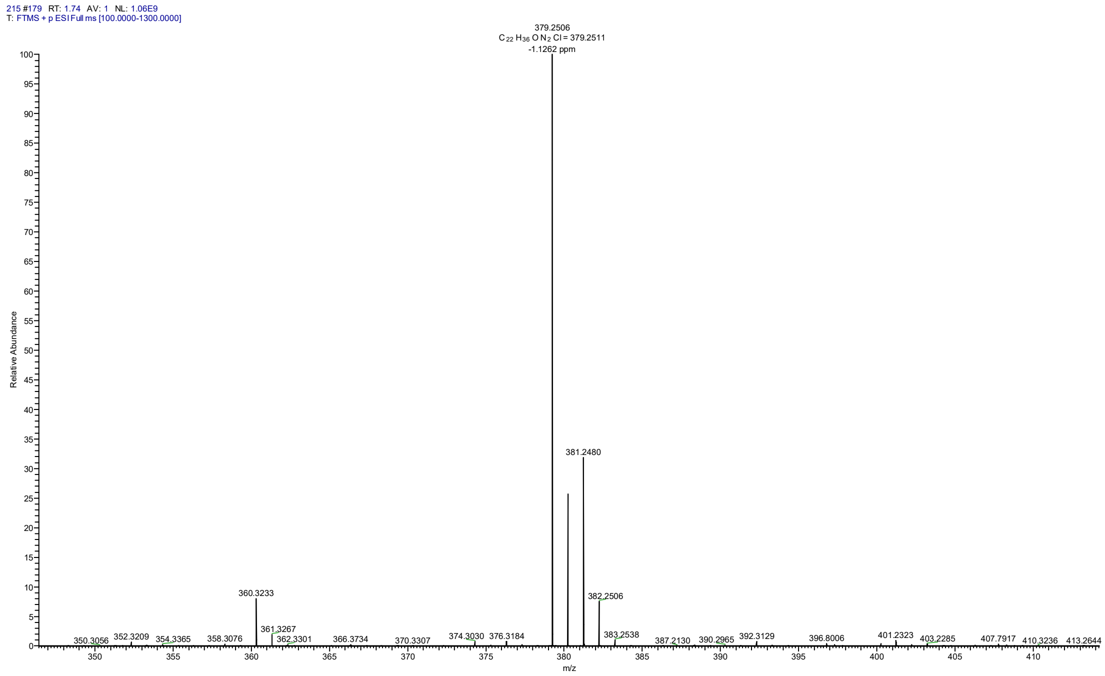

Figure S85. HRMS spectrum of **B1**.

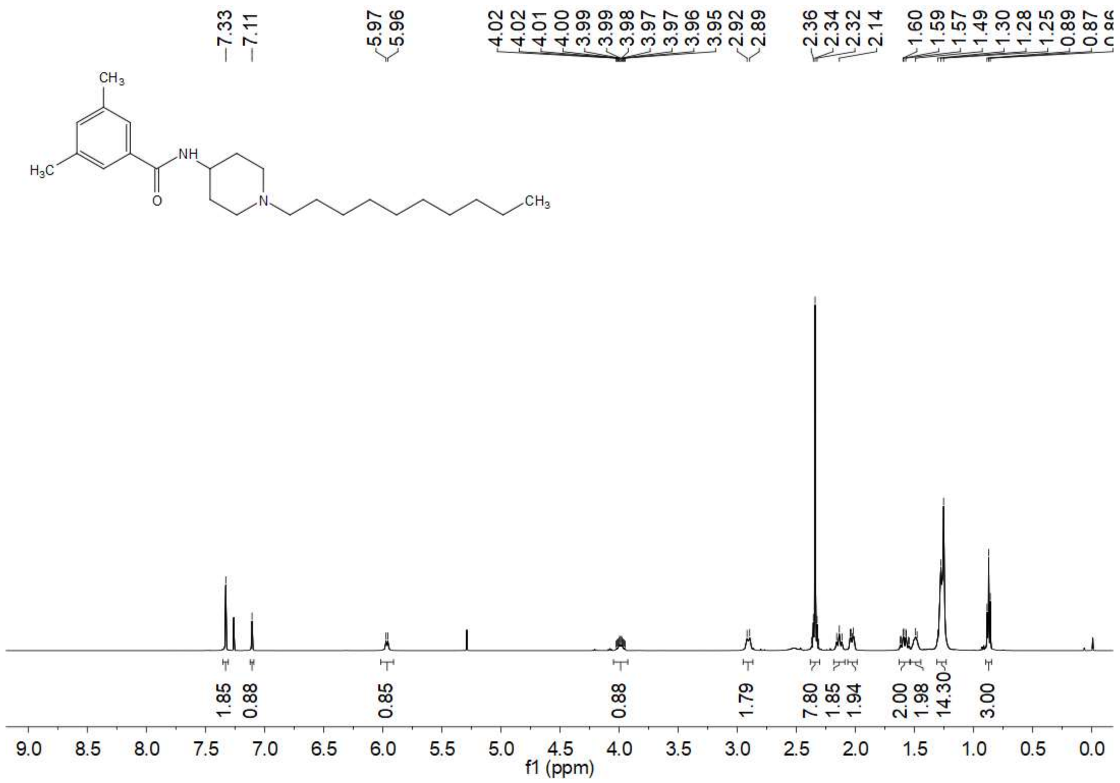

Figure S86. ^{1}H NMR spectrum ($CDCl_3$, 500 MHz) of **B_2**.

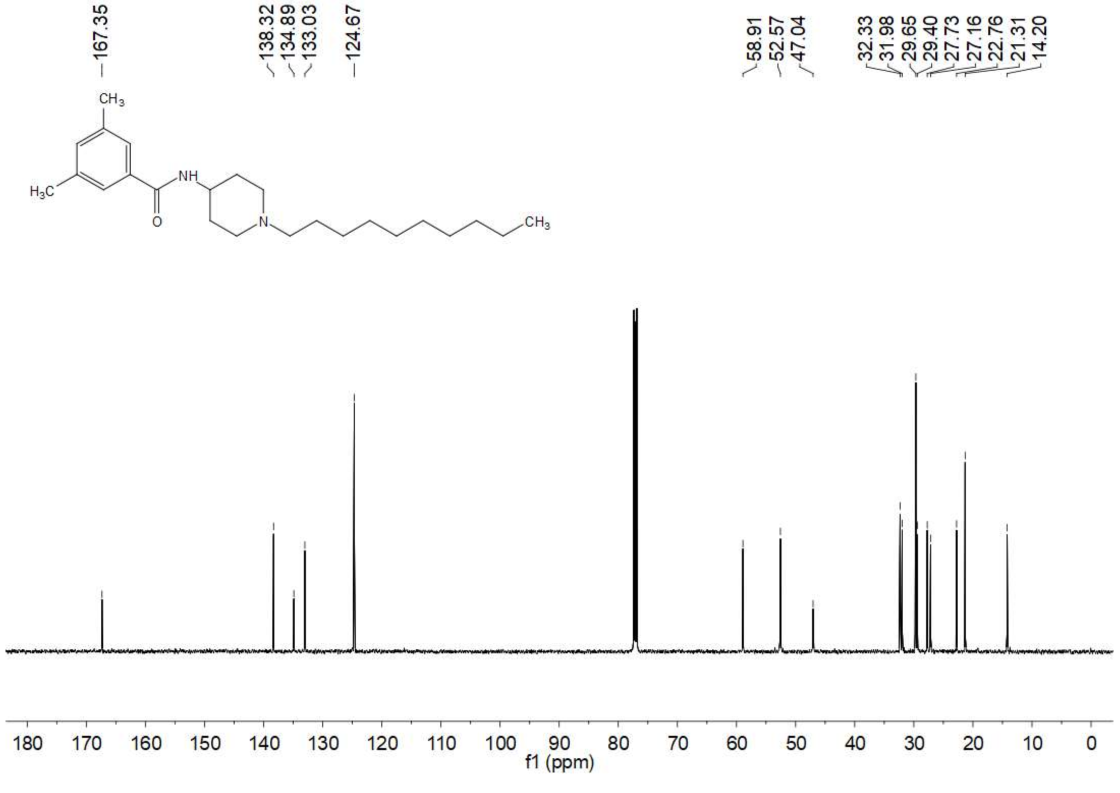

Figure S87. ^{13}C NMR spectrum ($CDCl_3$, 126 MHz) of **B_2**.

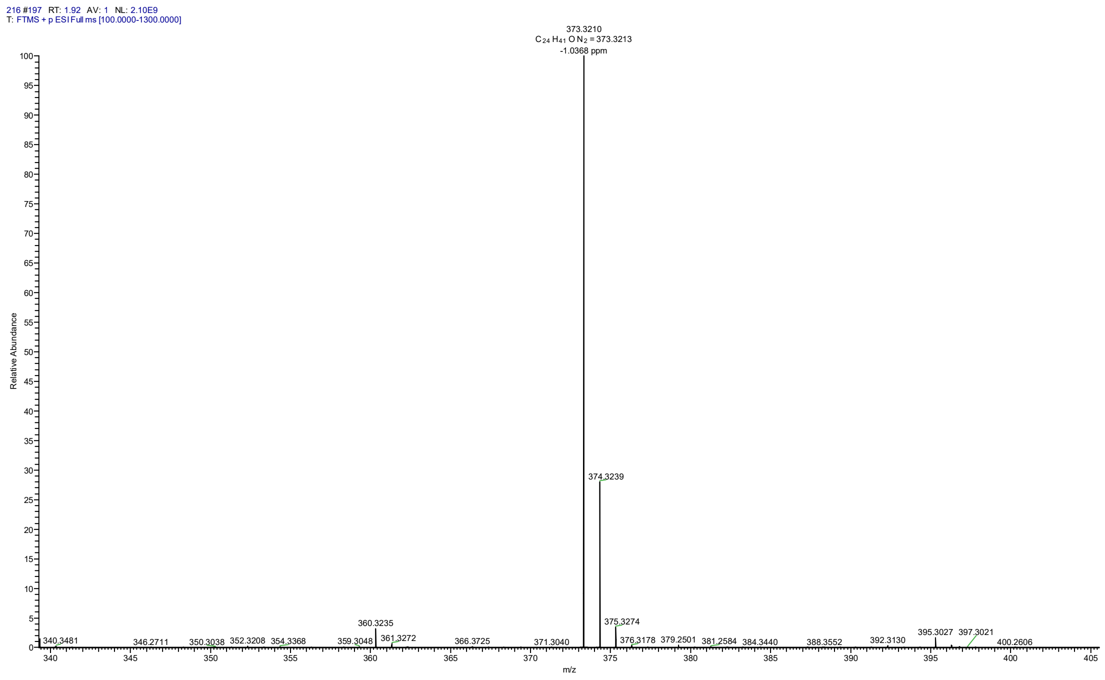

Figure S88. HRMS spectrum of **B2**.

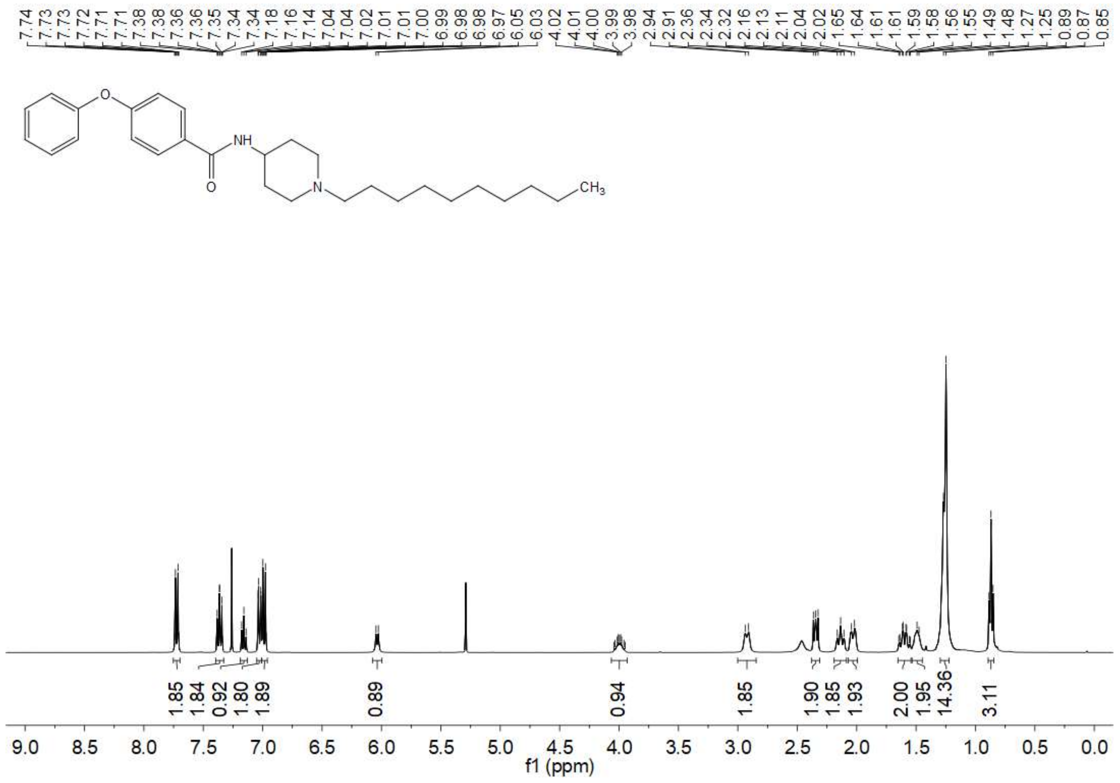

Figure S89. ^{1}H NMR spectrum ($CDCl_3$, 400 MHz) of **B3**.

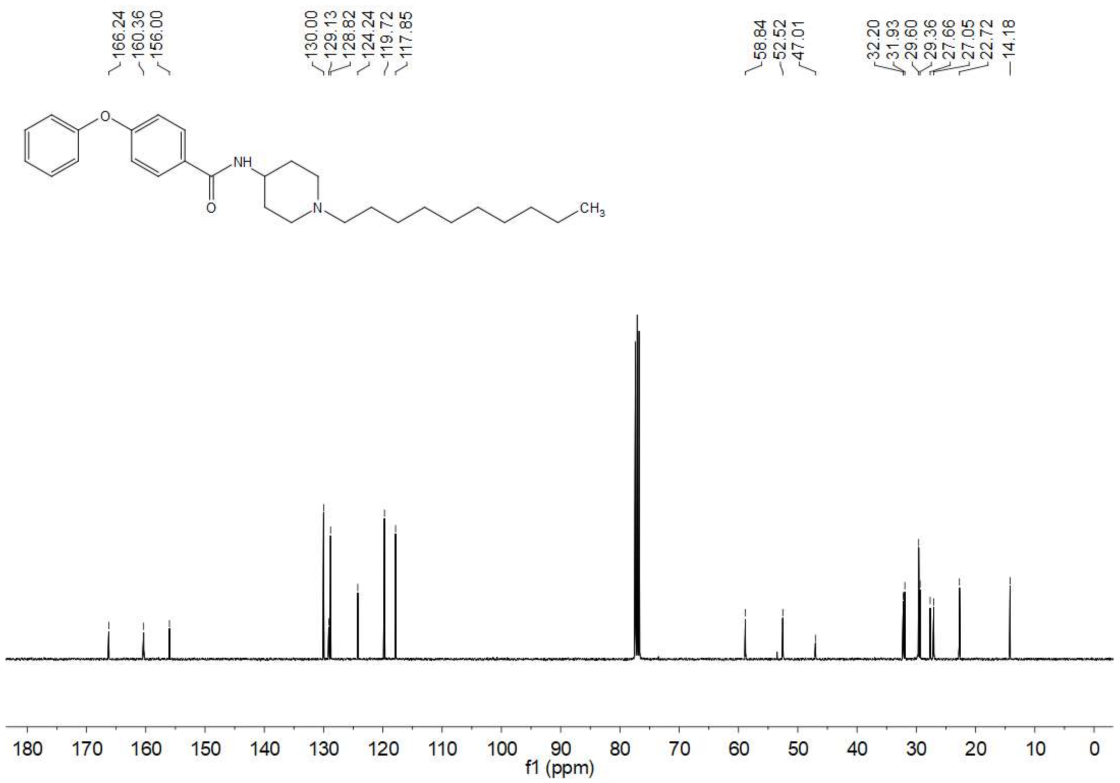

Figure S90. ^{13}C NMR spectrum ($CDCl_3$, 101 MHz) of **B3**.

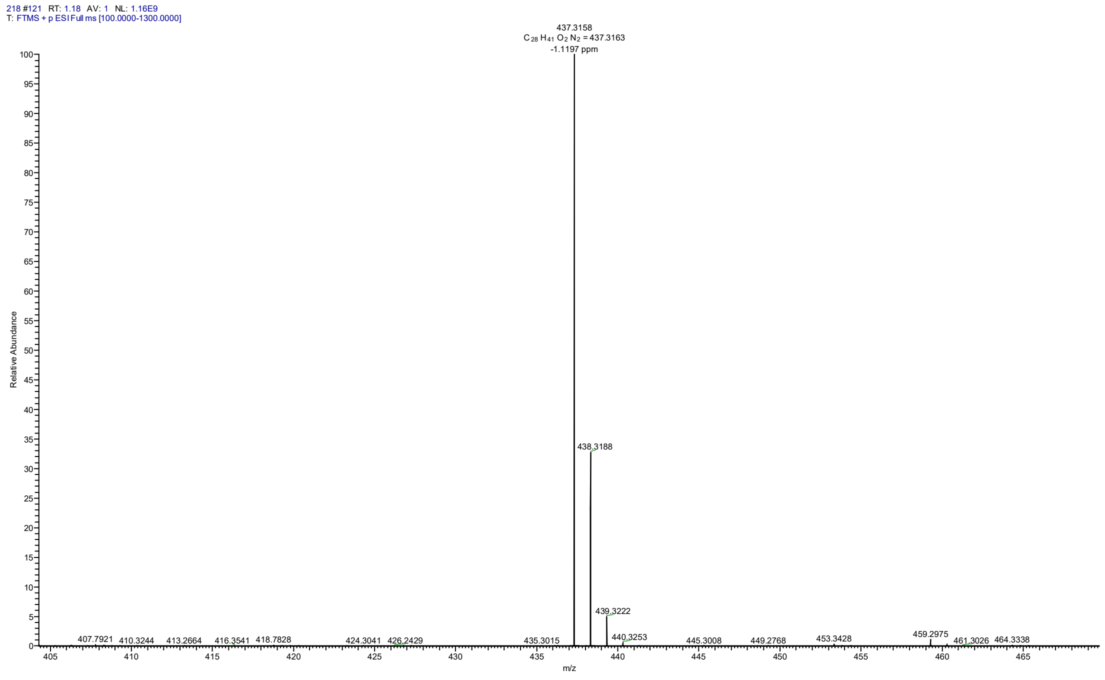

Figure S91. HRMS spectrum of **B3**.

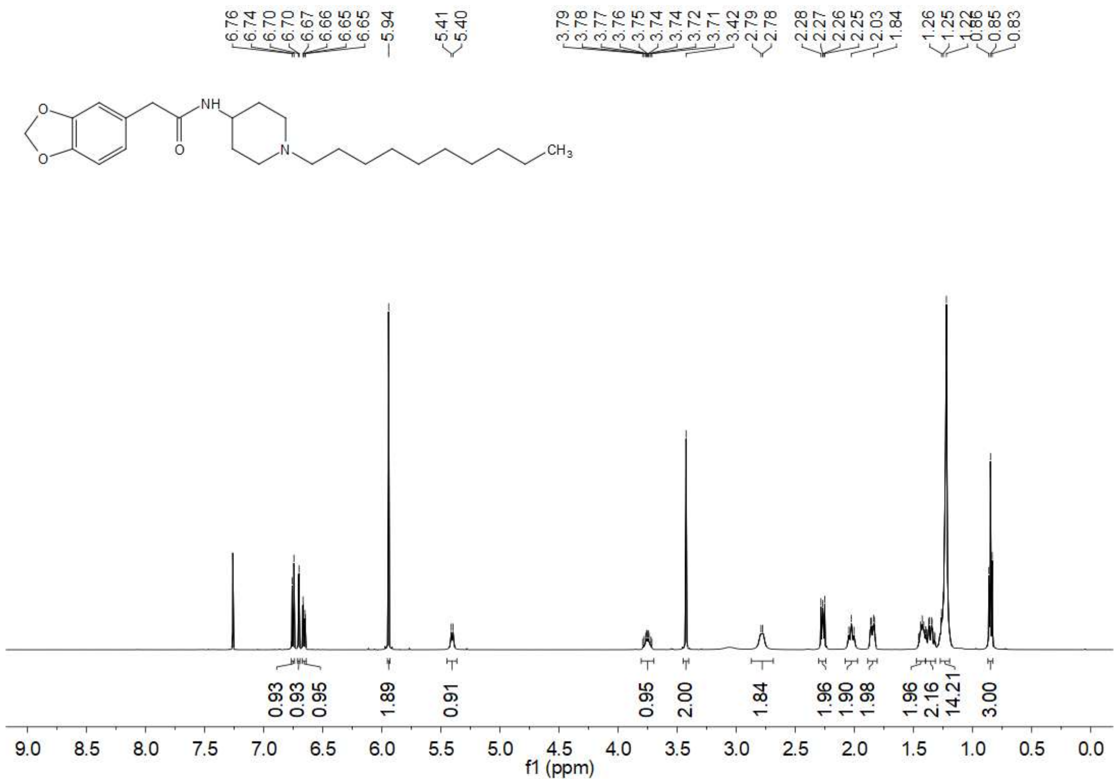

Figure S92. ^{1}H NMR spectrum ($CDCl_3$, 500 MHz) of **B$_4$**.

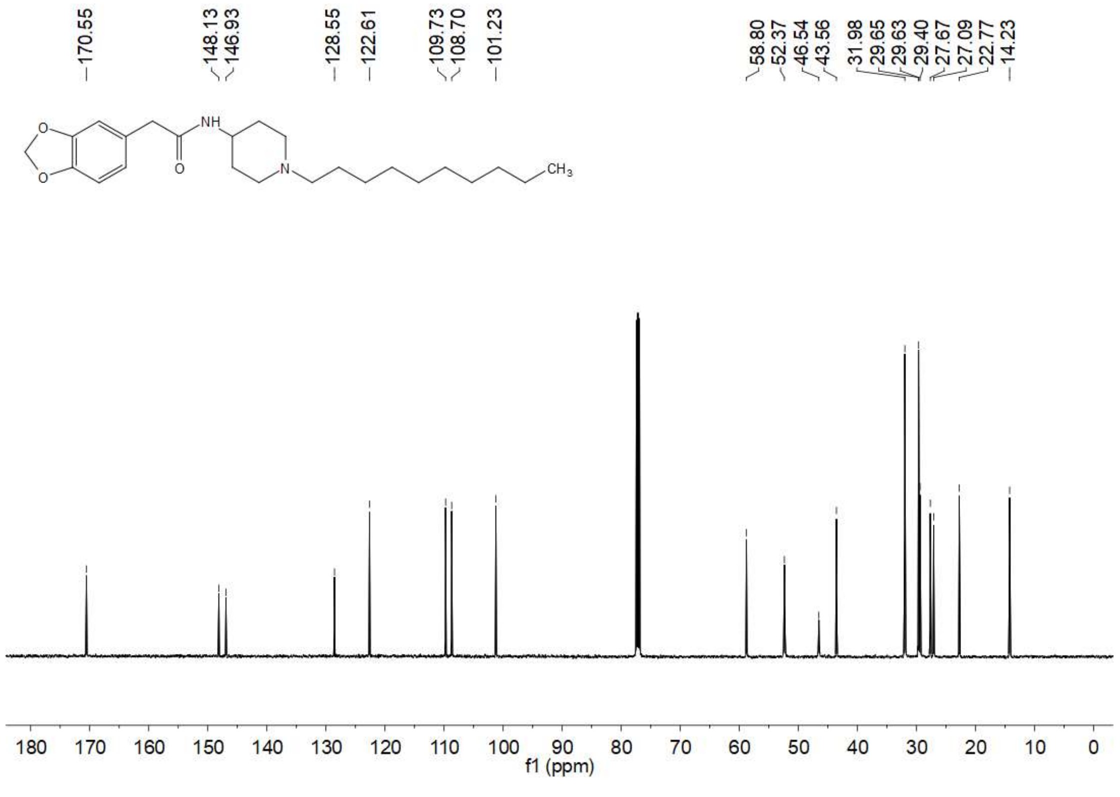

Figure S93. ^{13}C NMR spectrum ($CDCl_3$, 126 MHz) of **B$_4$**.

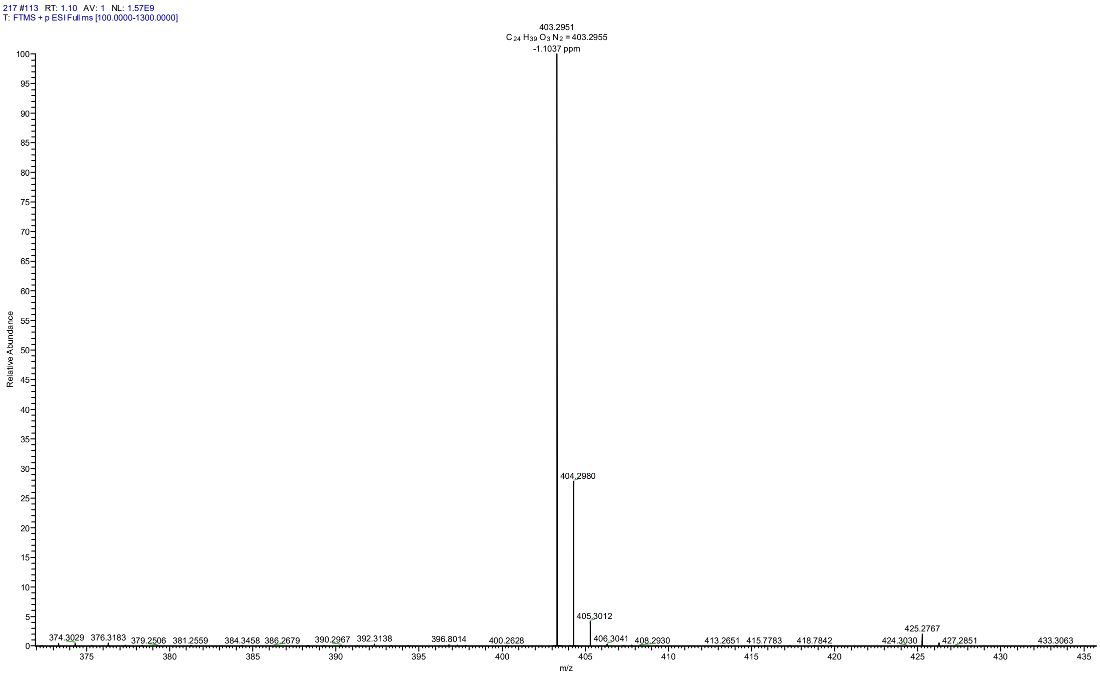

Figure S94. HRMS spectrum of **B**$_4$.

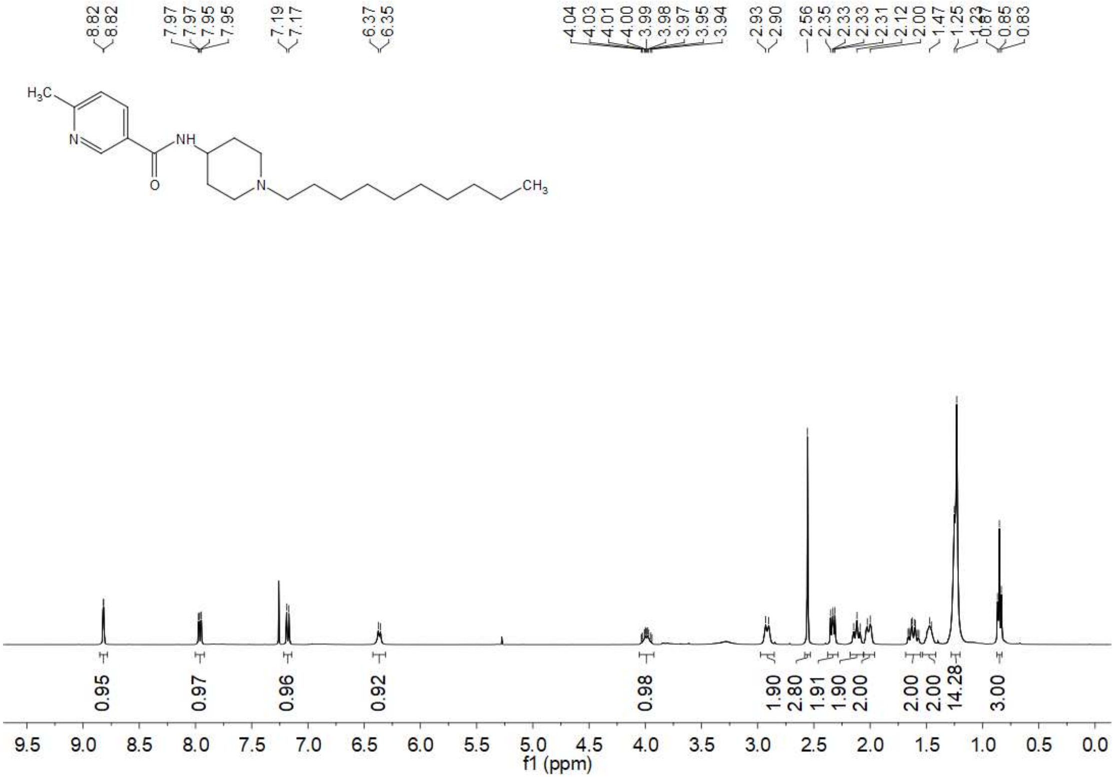

Figure S95. ^{1}H NMR spectrum ($CDCl_3$, 400 MHz) of **B**$_5$.

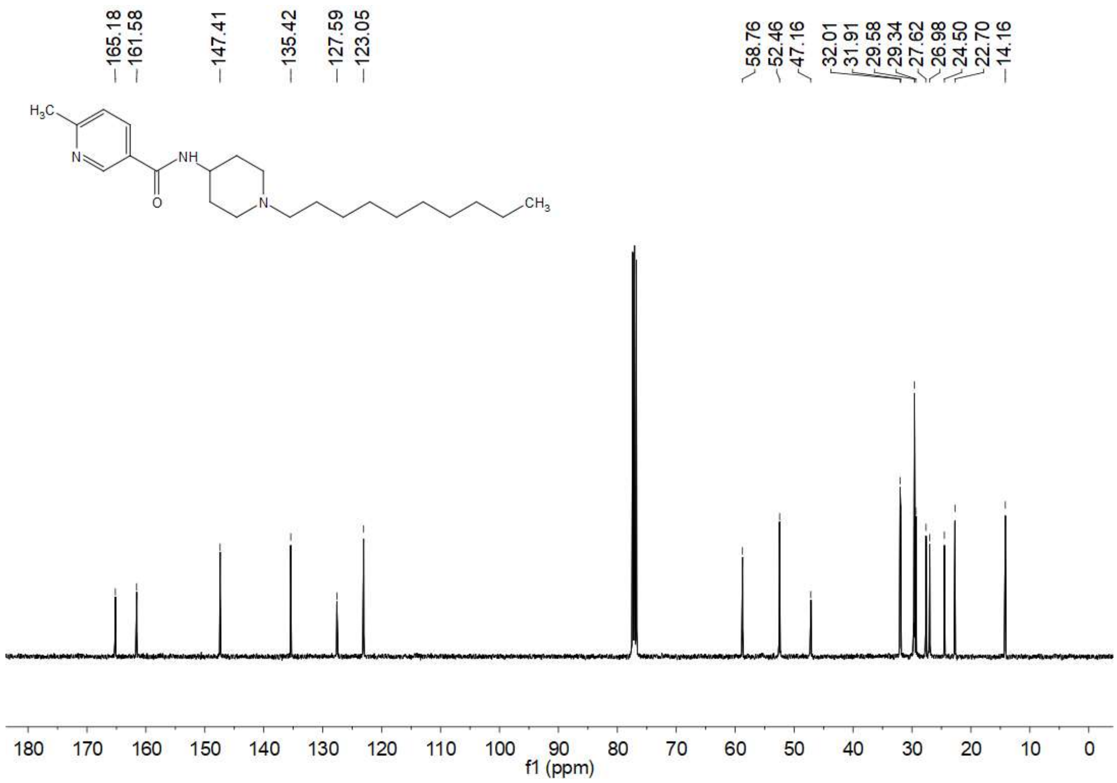

Figure S96. ^{13}C NMR spectrum ($CDCl_3$, 101 MHz) of $\mathbf{B_5}$.

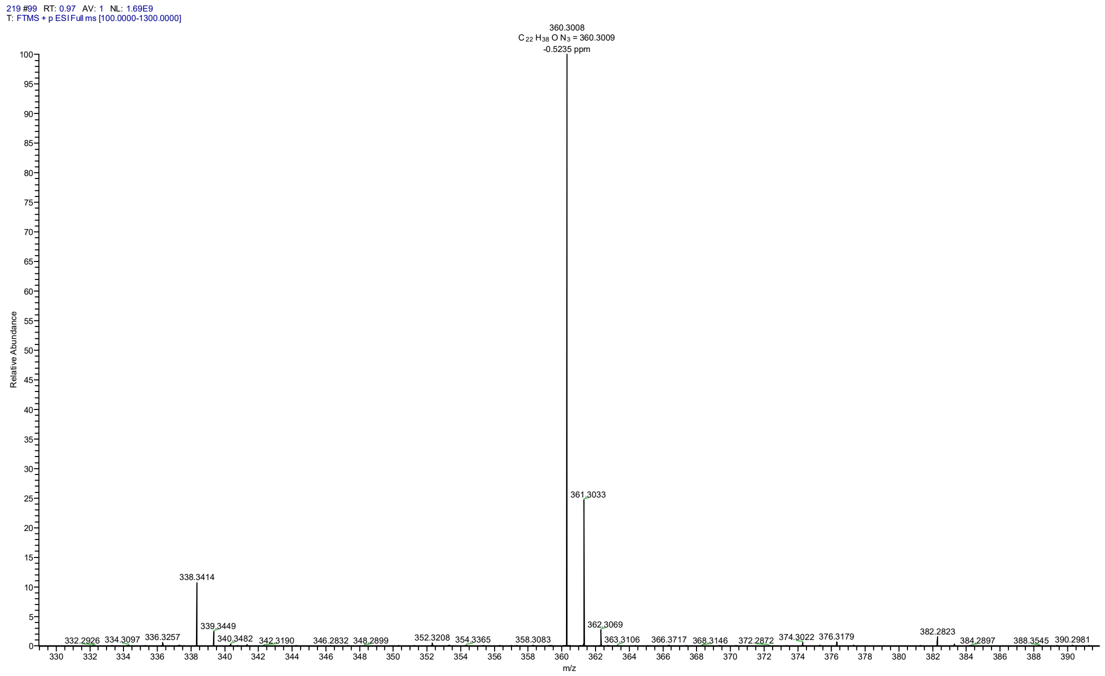

Figure S97. HRMS spectrum of $\mathbf{B_5}$.

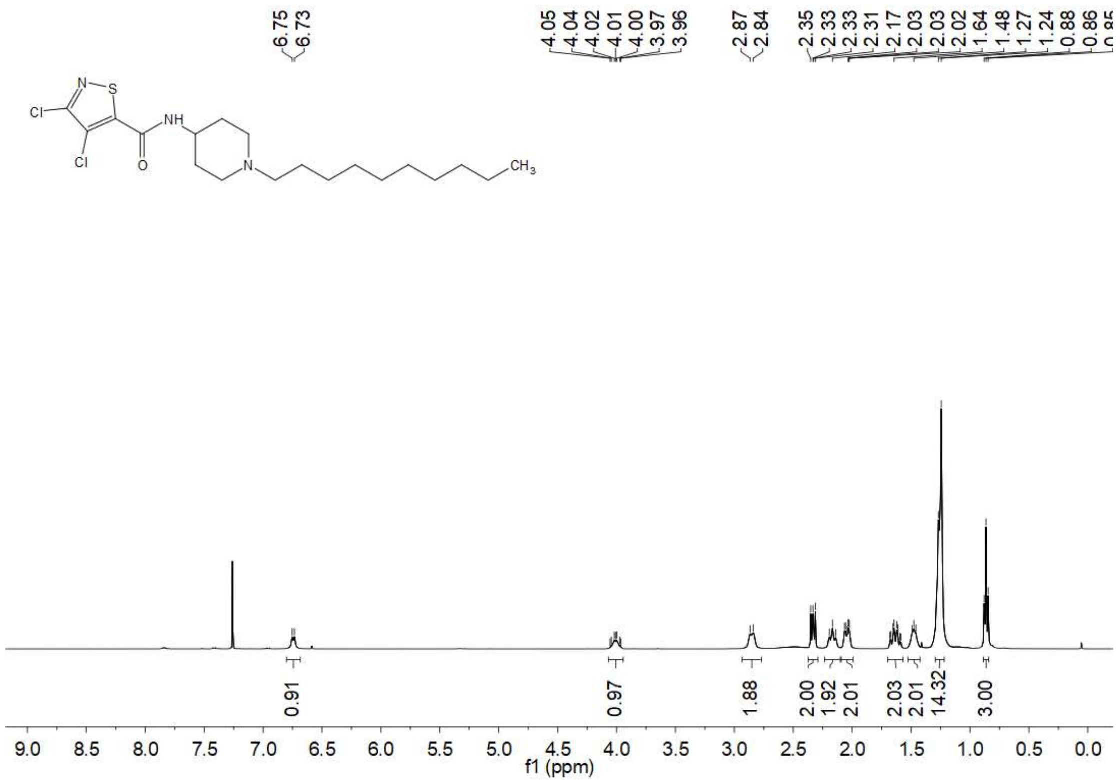

Figure S98. ^{1}H NMR spectrum ($CDCl_3$, 400 MHz) of **B$_6$**.

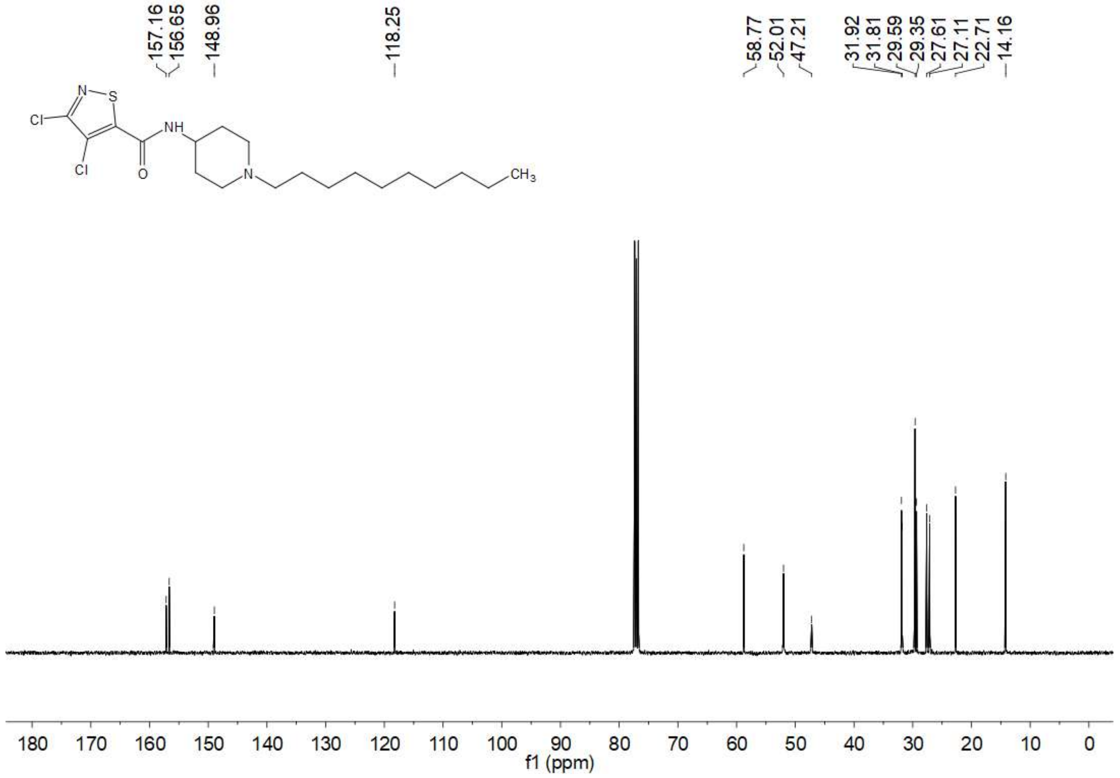

Figure S99. ^{13}C NMR spectrum ($CDCl_3$, 101 MHz) of **B$_6$**.

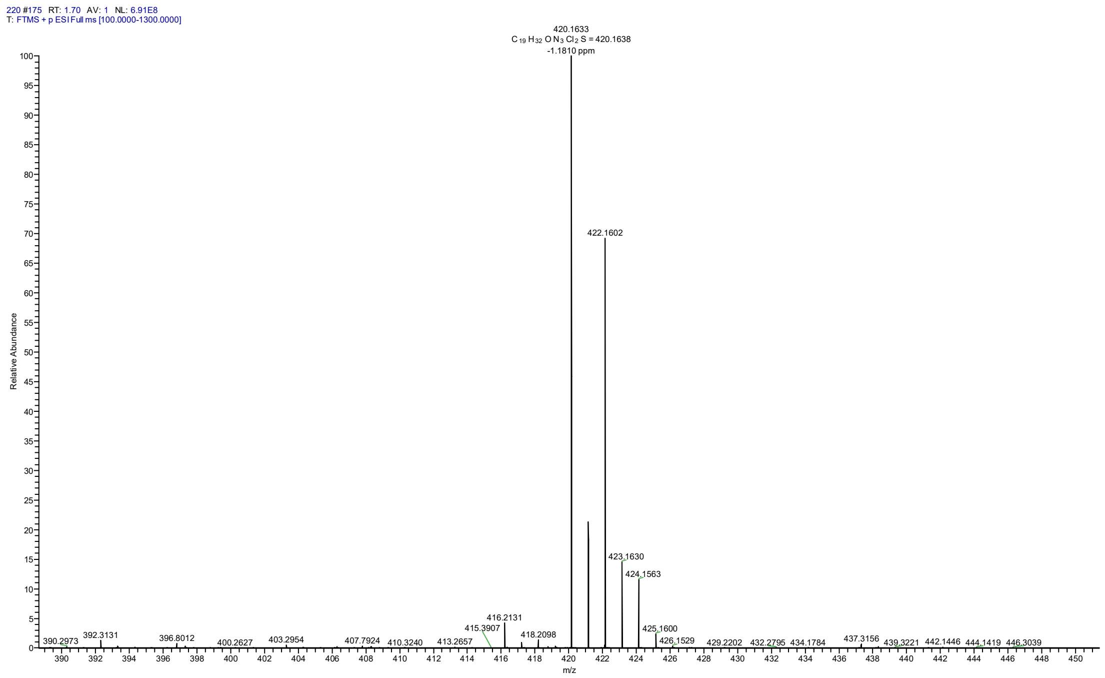

Figure S100. HRMS spectrum of $\mathbf{B_6}$.

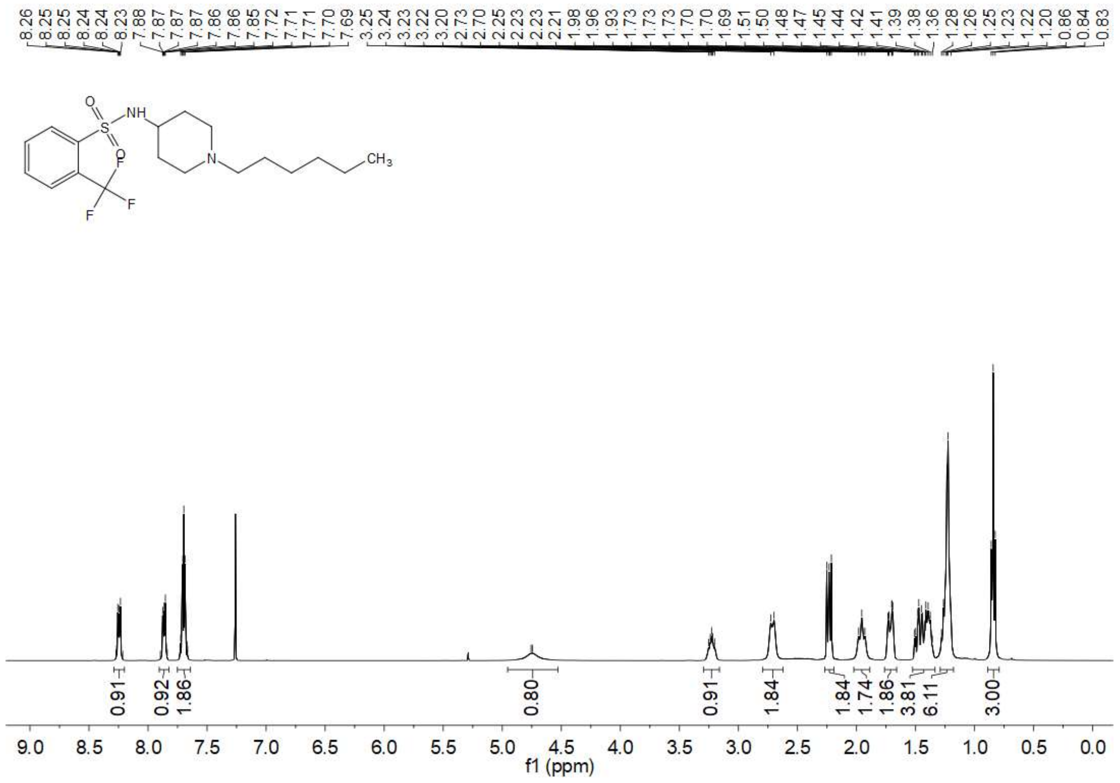

Figure S101. ^{1}H NMR spectrum ($CDCl_3$, 400 MHz) of $\mathbf{C_1}$.

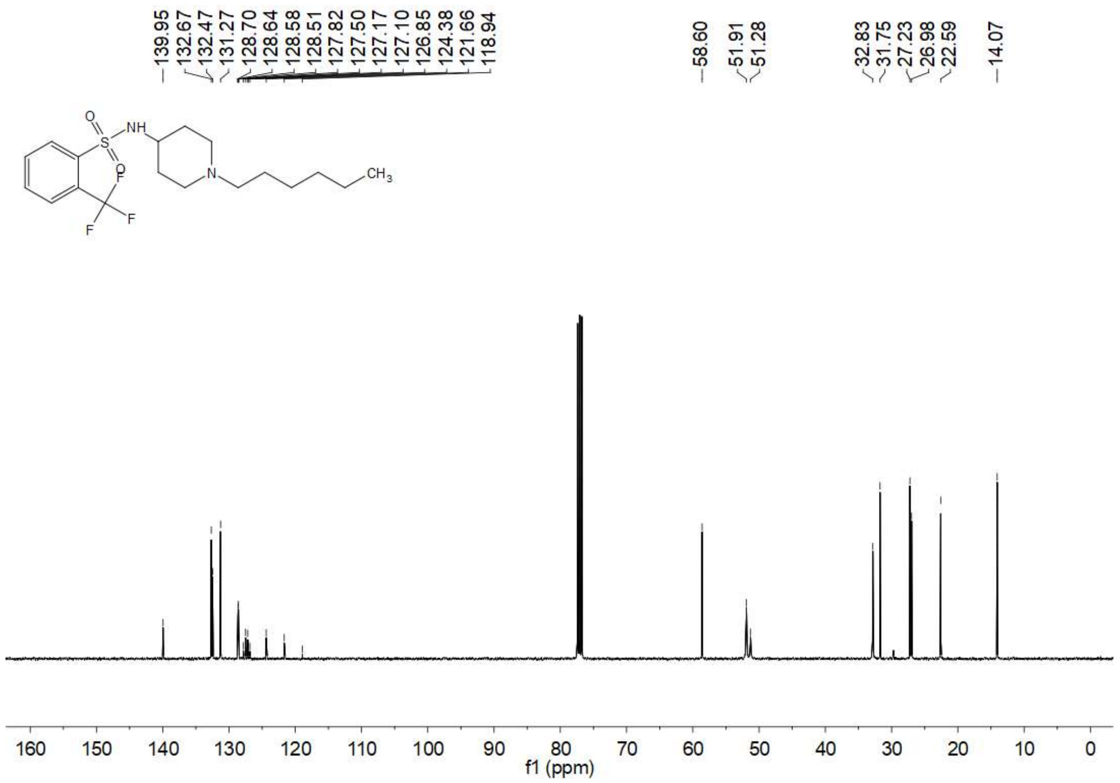

Figure S102. ^{13}C NMR spectrum ($CDCl_3$, 101 MHz) of **C_1**.

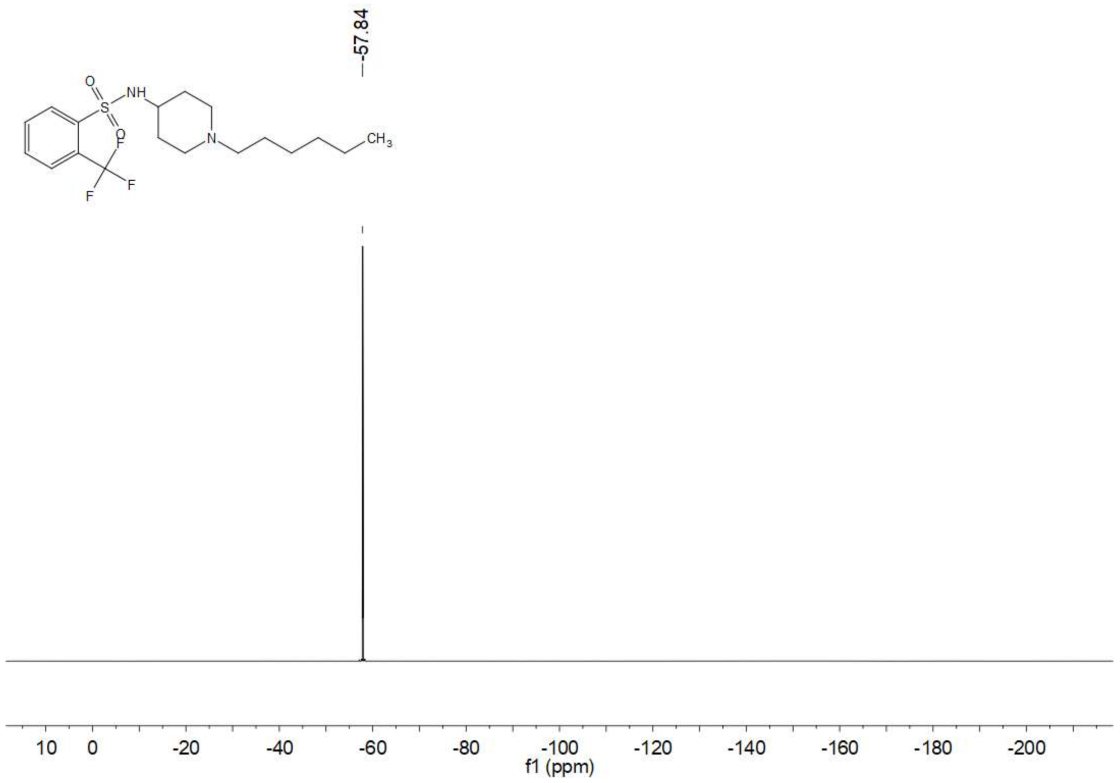

Figure S103. ^{19}F NMR spectrum ($CDCl_3$, 376 MHz) of **C_1**.

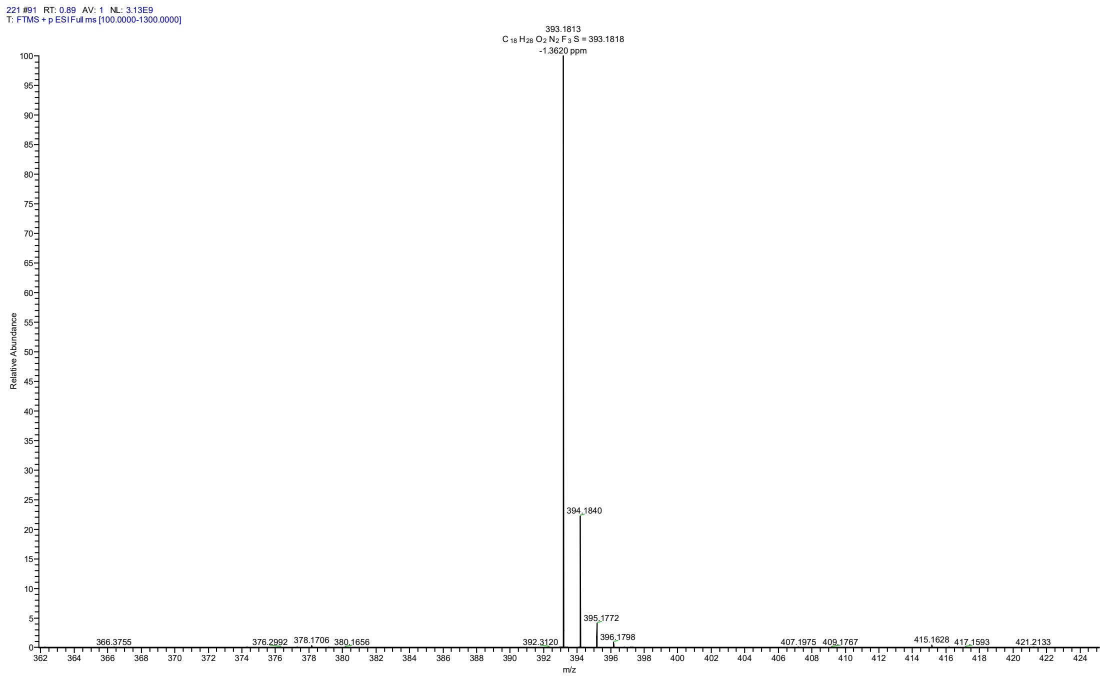

Figure S104. HRMS spectrum of $\mathbf{C_1}$.

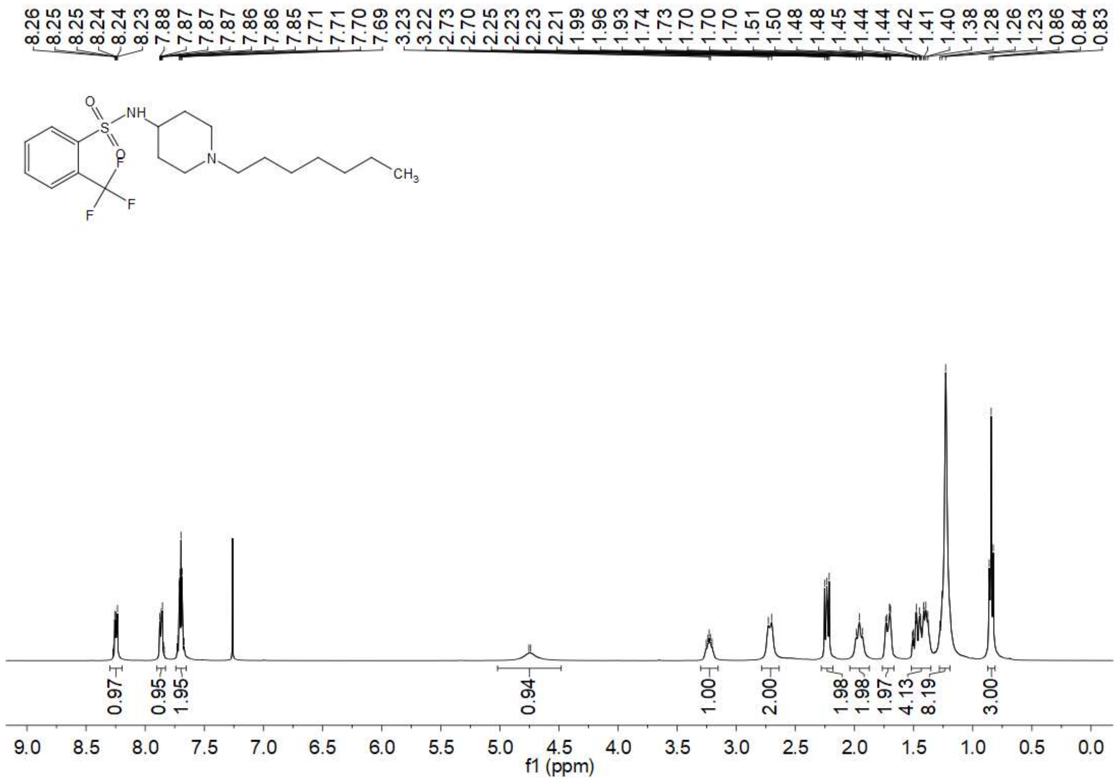

Figure S105. ^{1}H NMR spectrum ($CDCl_3$, 400 MHz) of $\mathbf{C_2}$.

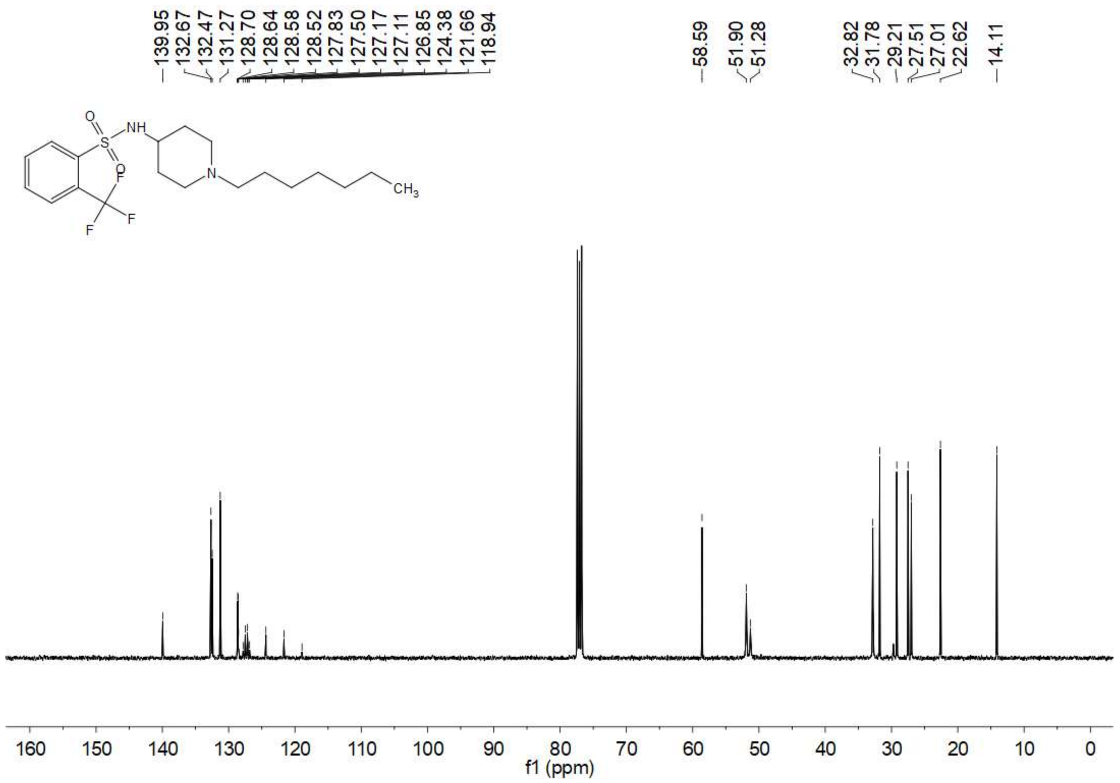

Figure S106. ^{13}C NMR spectrum ($CDCl_3$, 101 MHz) of **C_2**.

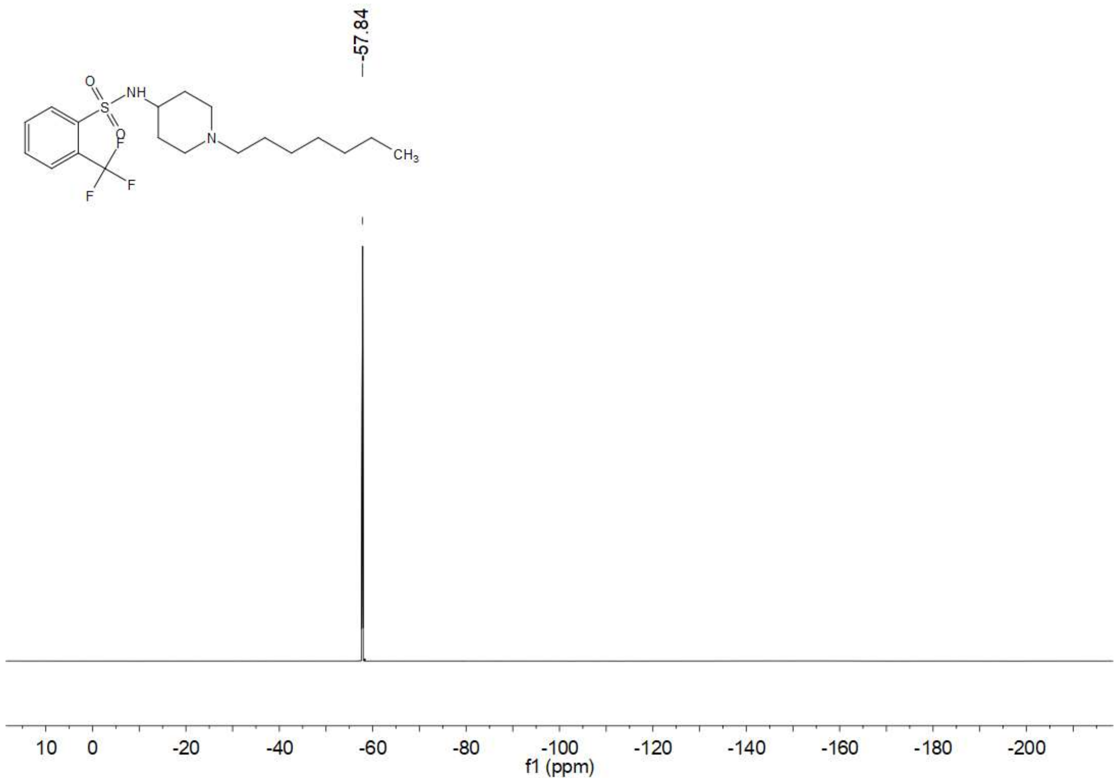

Figure S107. ^{19}F NMR spectrum ($CDCl_3$, 376 MHz) of **C_2**.

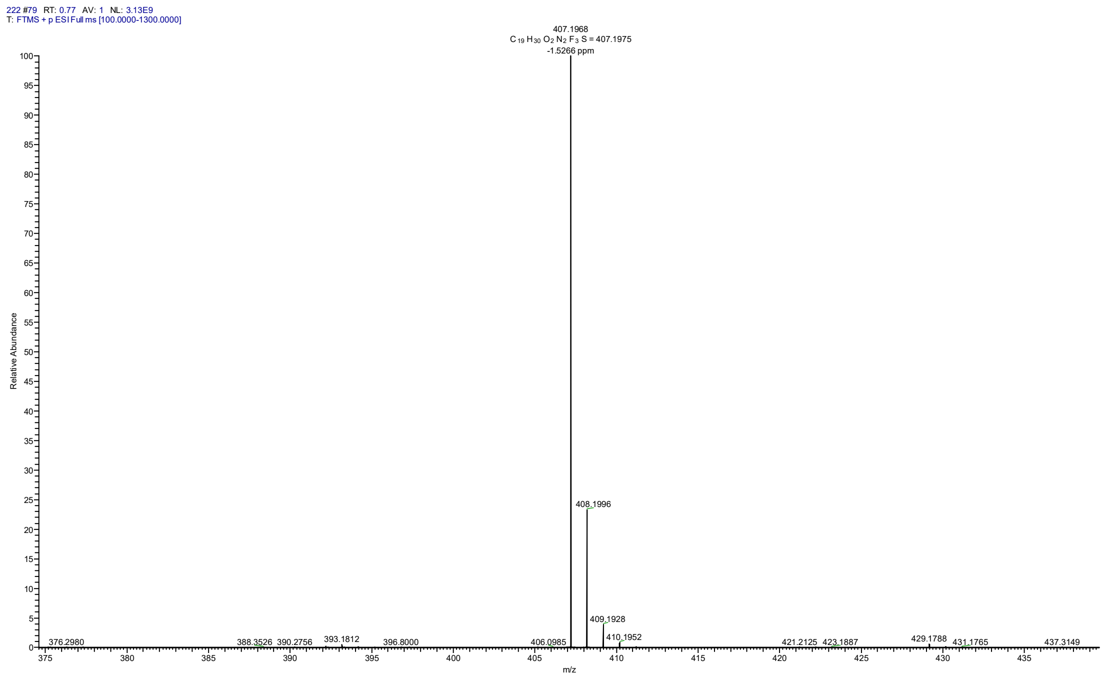

Figure S108. HRMS spectrum of $\mathbf{C_2}$.

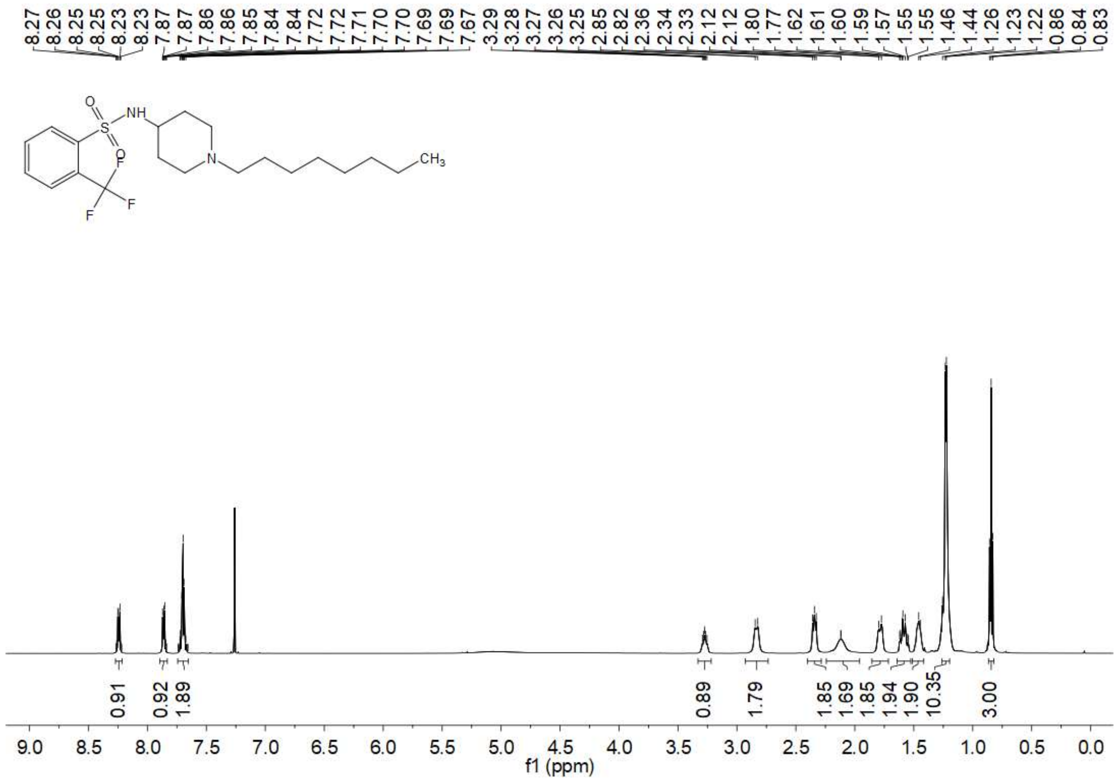

Figure S109. ^{1}H NMR spectrum ($CDCl_3$, 500 MHz) of $\mathbf{C_3}$.

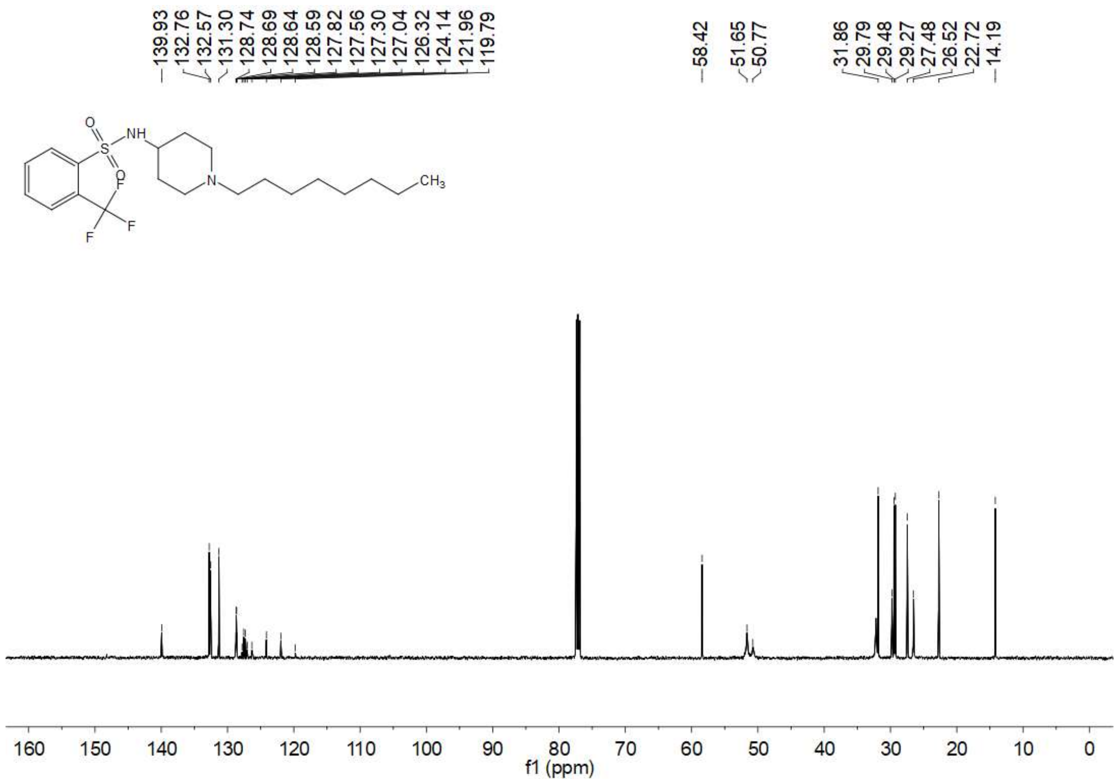

Figure S110. ^{13}C NMR spectrum ($CDCl_3$, 126 MHz) of **C3**.

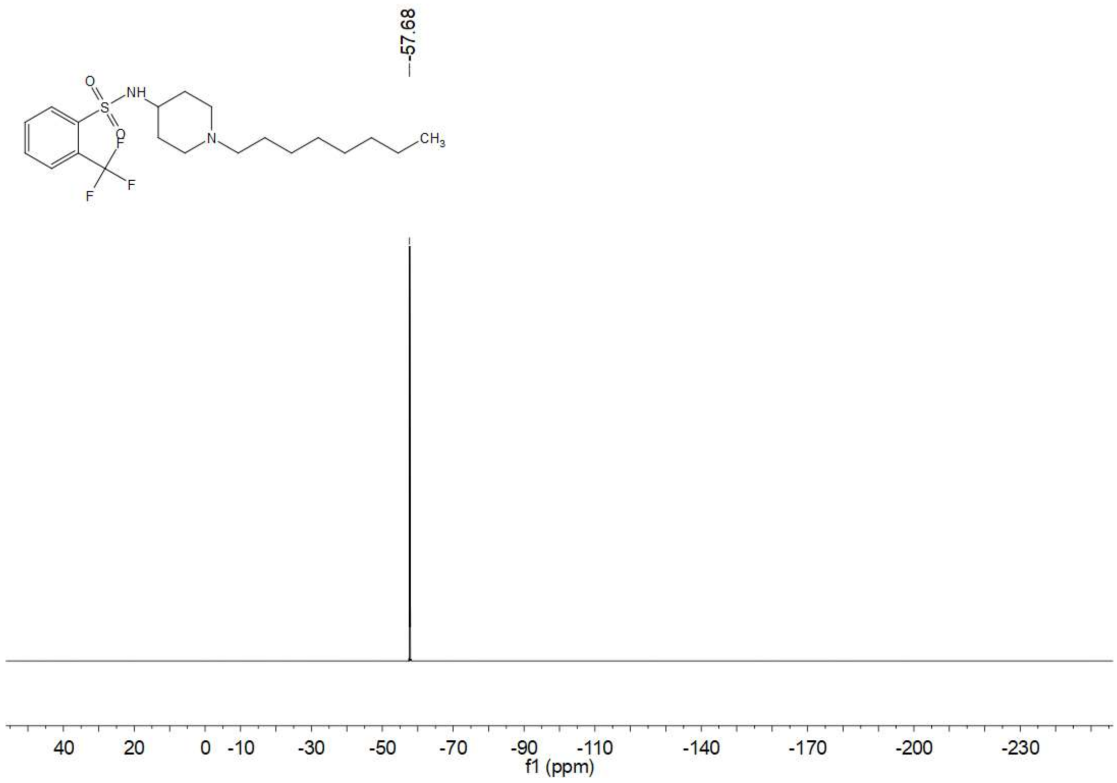

Figure S111. ^{19}F NMR spectrum ($CDCl_3$, 471 MHz) of **C3**.

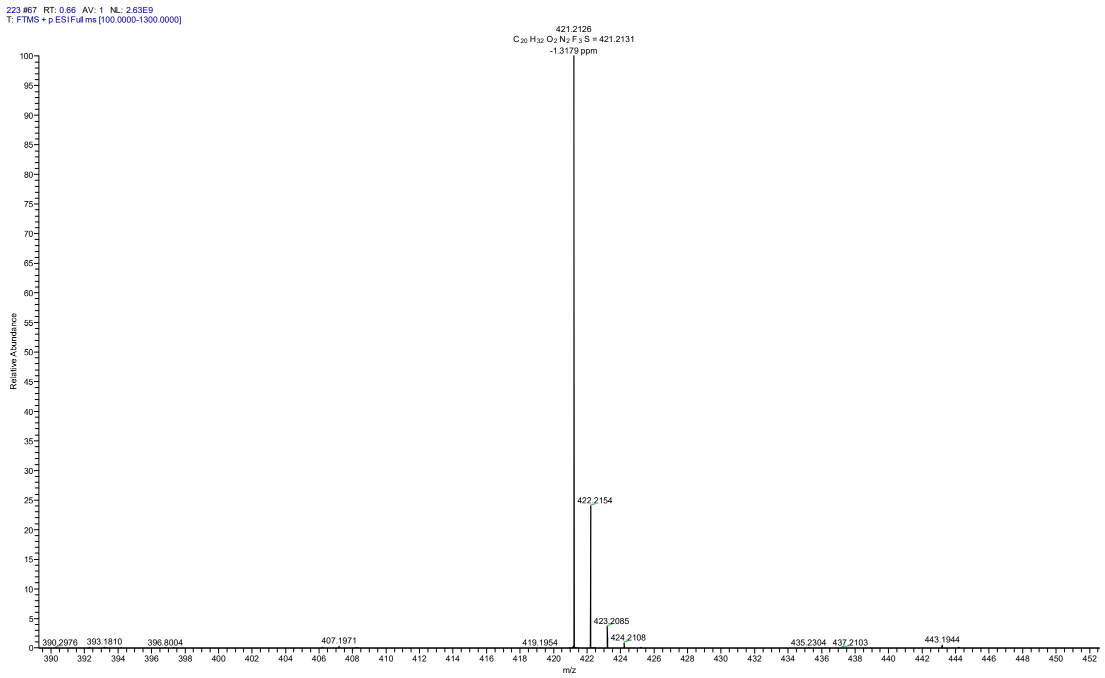

Figure S112. HRMS spectrum of **C3**.

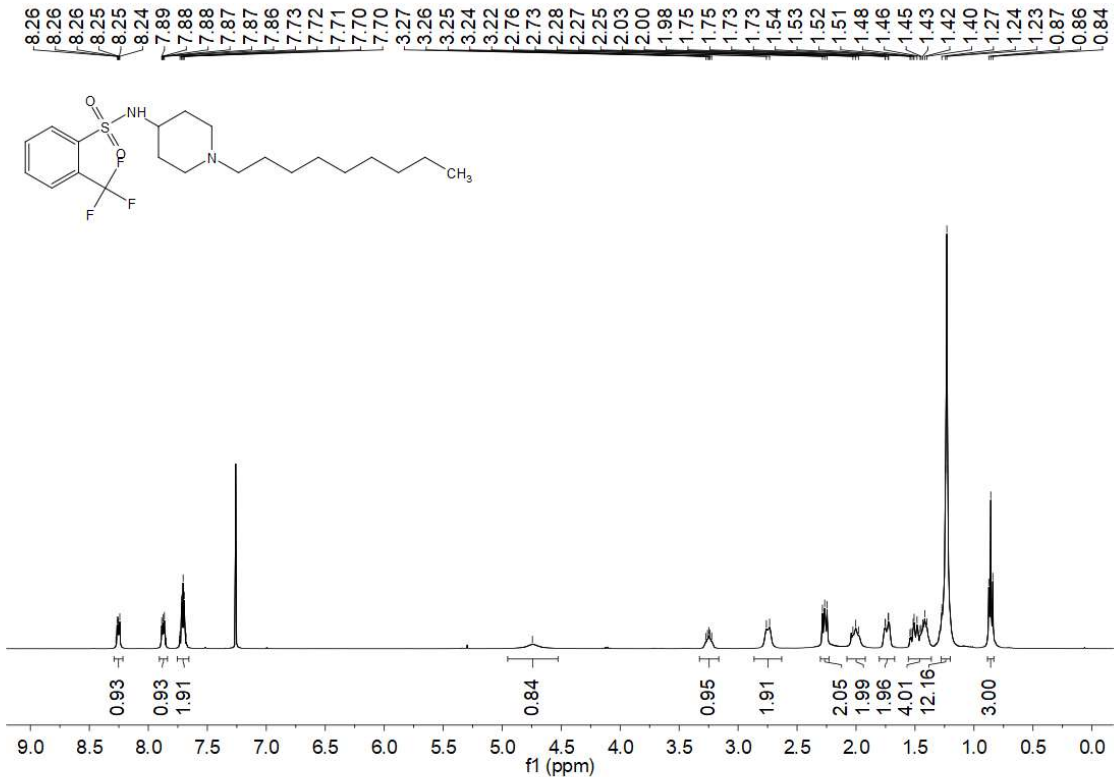

Figure S113. ^{1}H NMR spectrum ($CDCl_3$, 400 MHz) of **C4**.

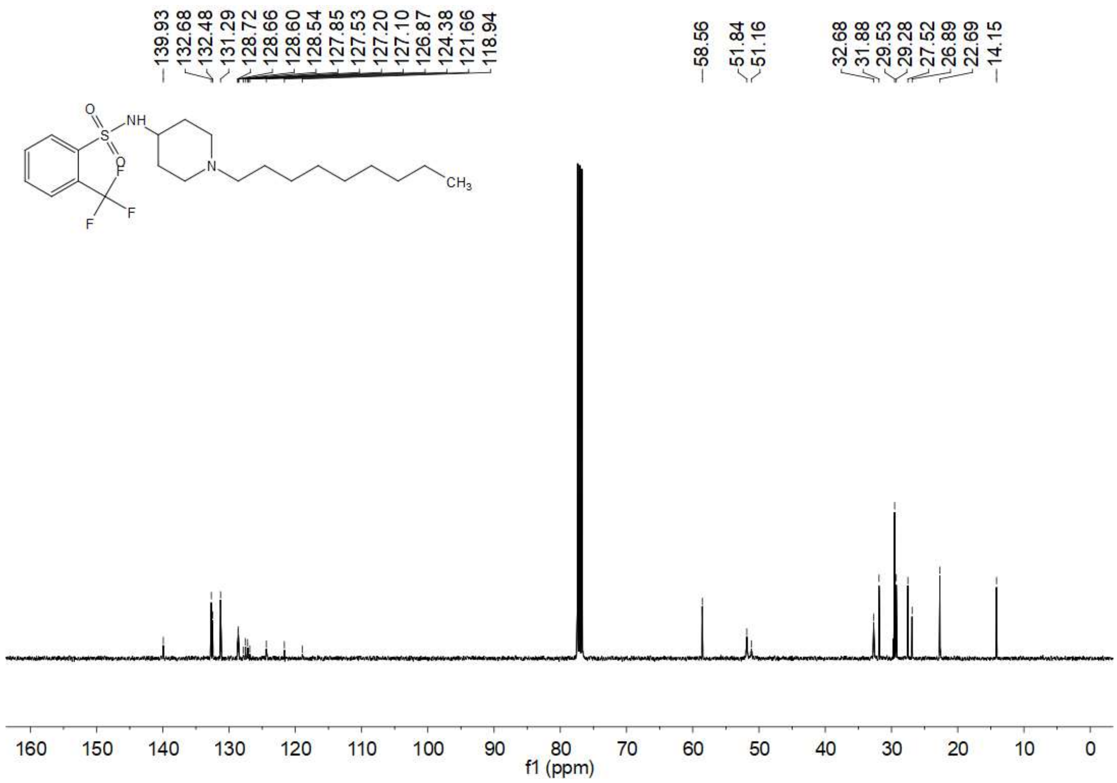

Figure S114. ^{13}C NMR spectrum ($CDCl_3$, 101 MHz) of **C4**.

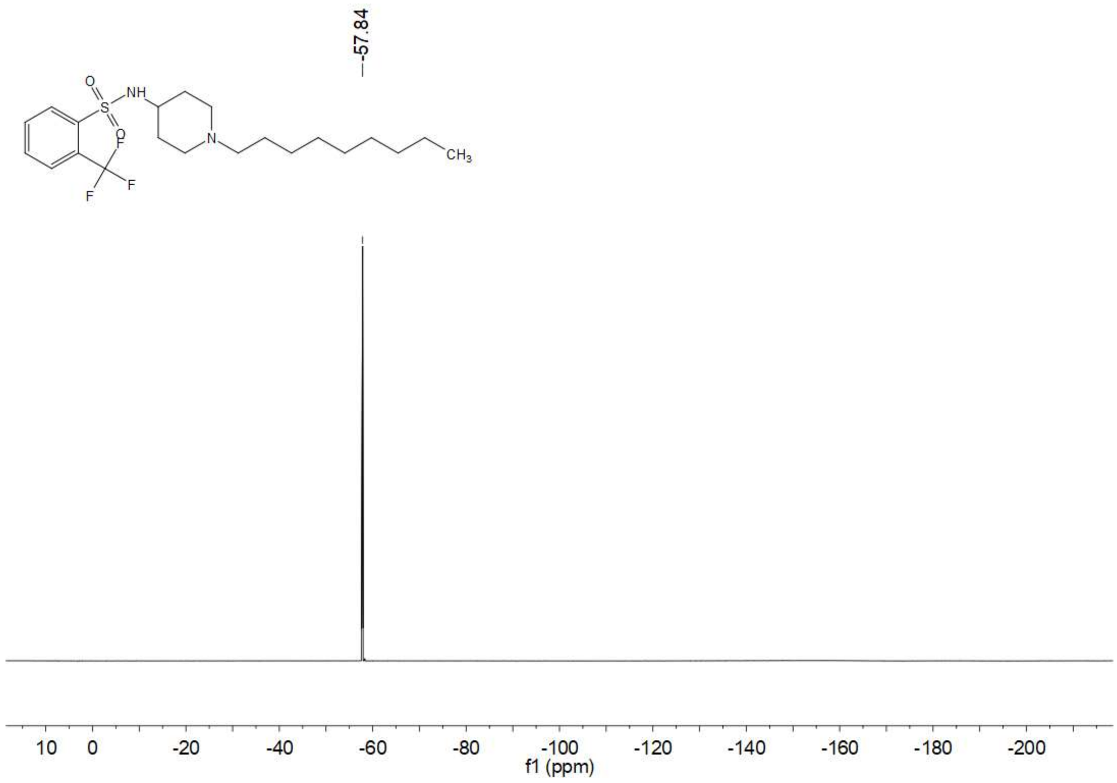

Figure S115. ^{19}F NMR spectrum ($CDCl_3$, 376 MHz) of **C4**.

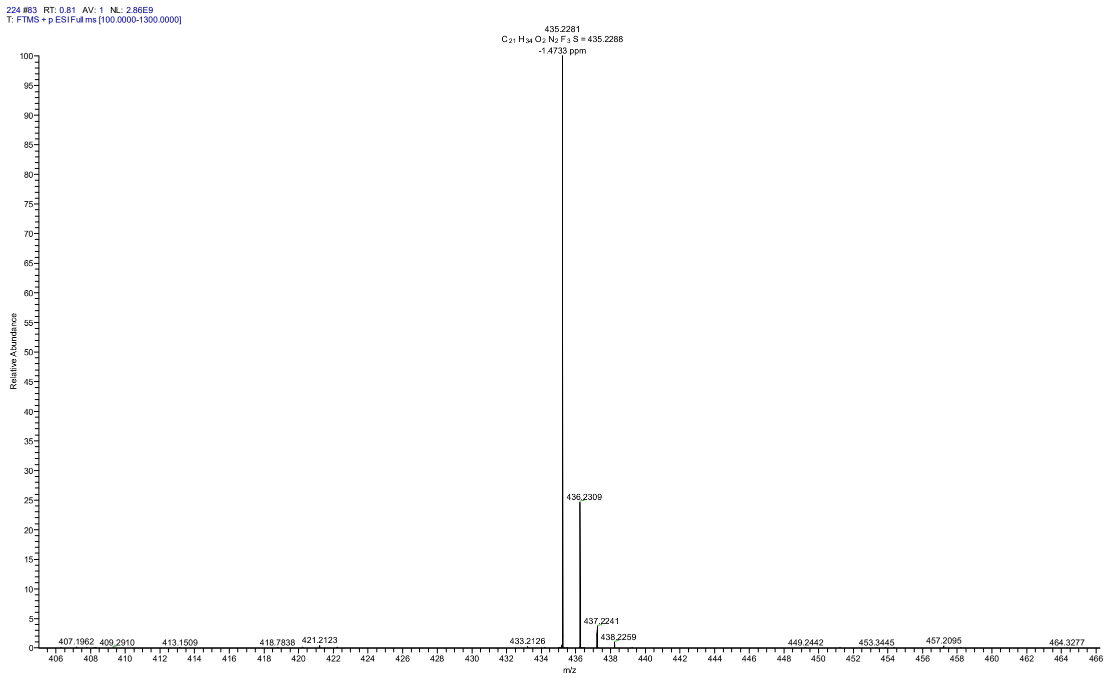

Figure S116. HRMS spectrum of **C_4**.

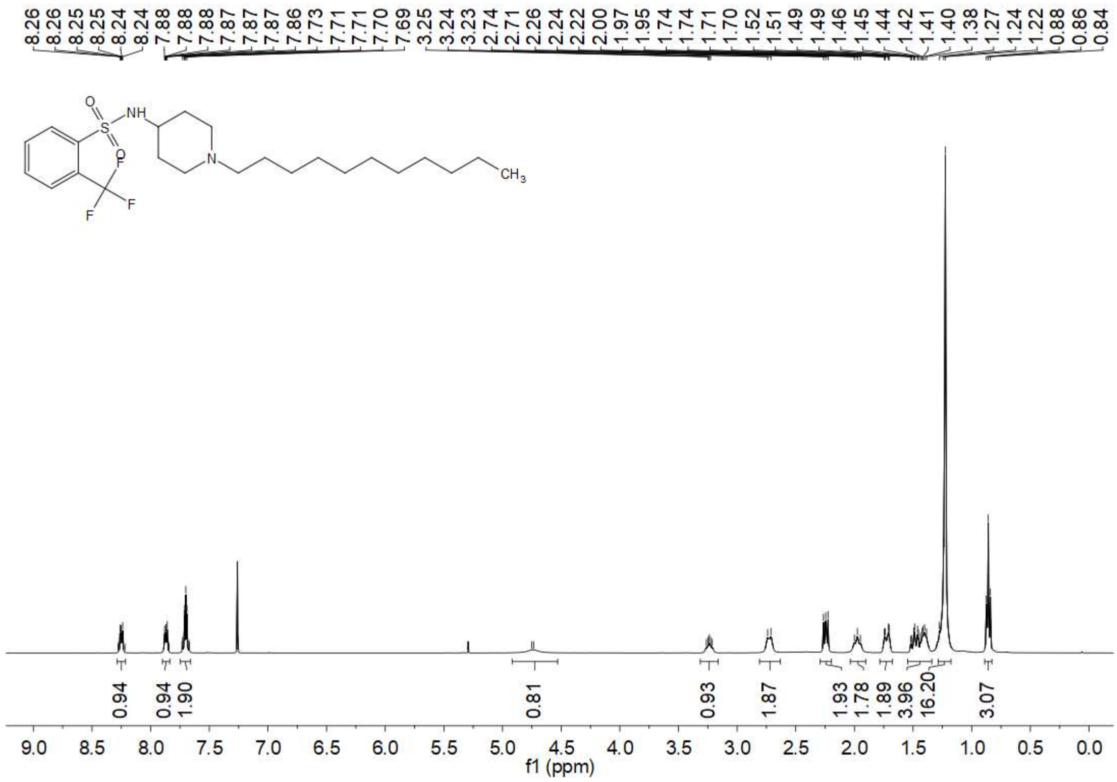

Figure S117. ^1H NMR spectrum ($CDCl_3$, 400 MHz) of **C_5**.

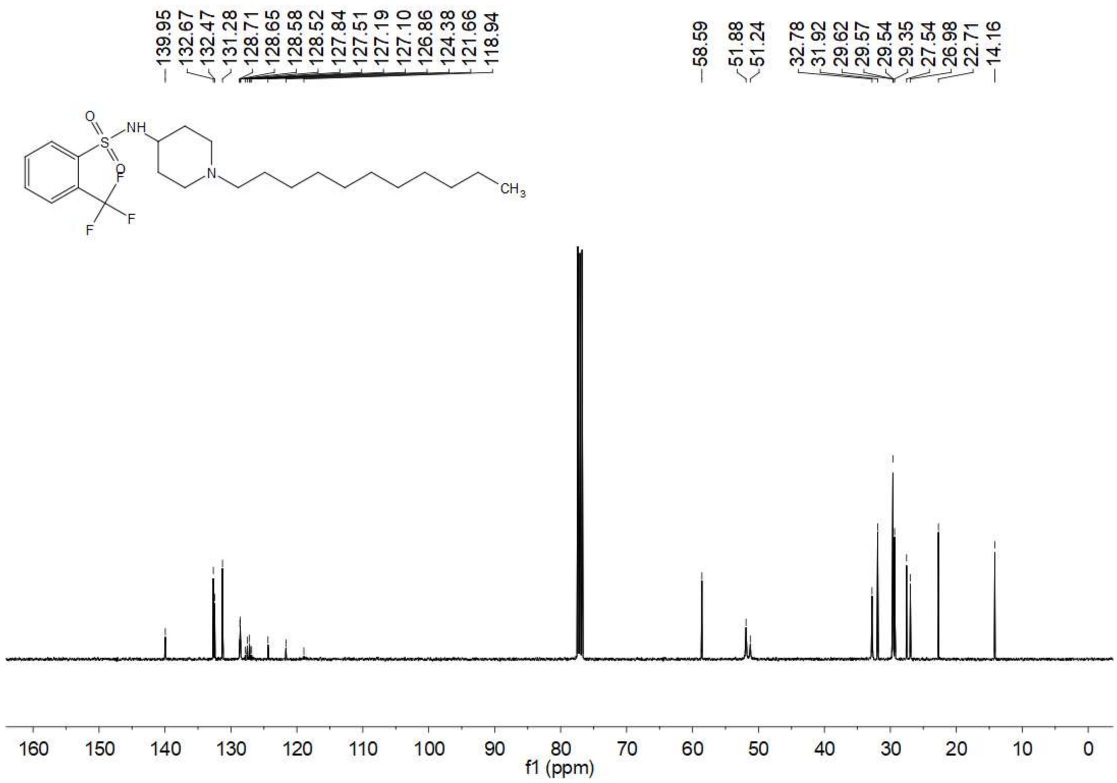

Figure S118. ^{13}C NMR spectrum ($CDCl_3$, 101 MHz) of **C_5**.

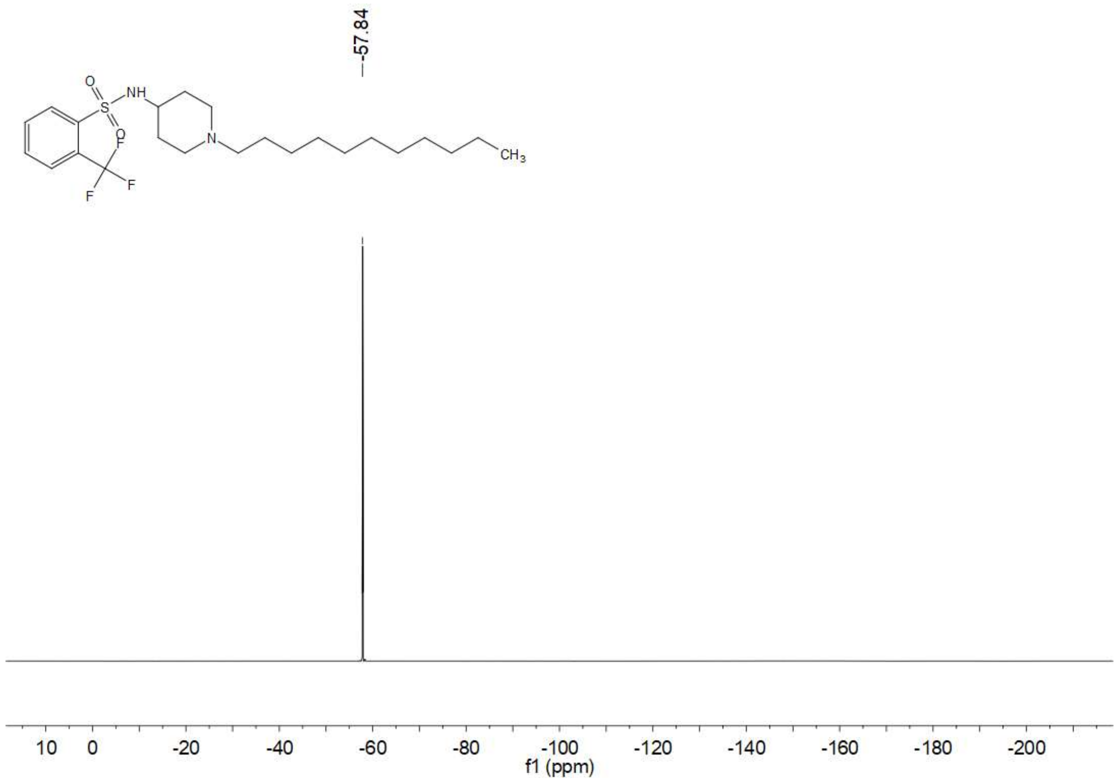

Figure S119. ^{19}F NMR spectrum ($CDCl_3$, 376 MHz) of **C_5**.

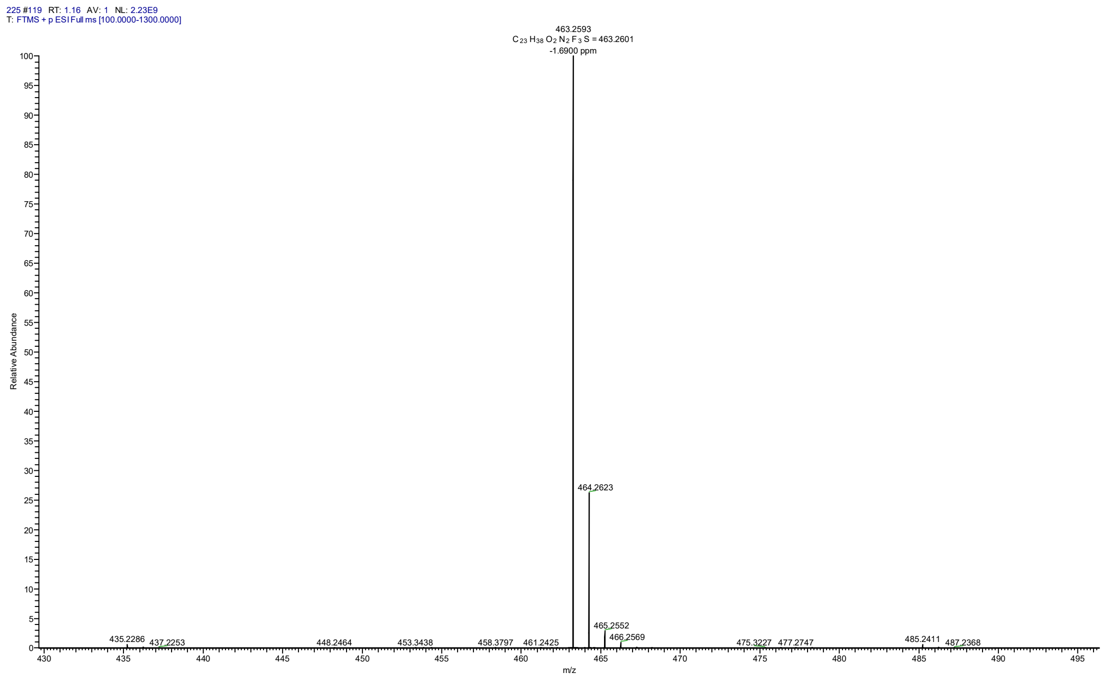

Figure S120. HRMS spectrum of **C_5**.

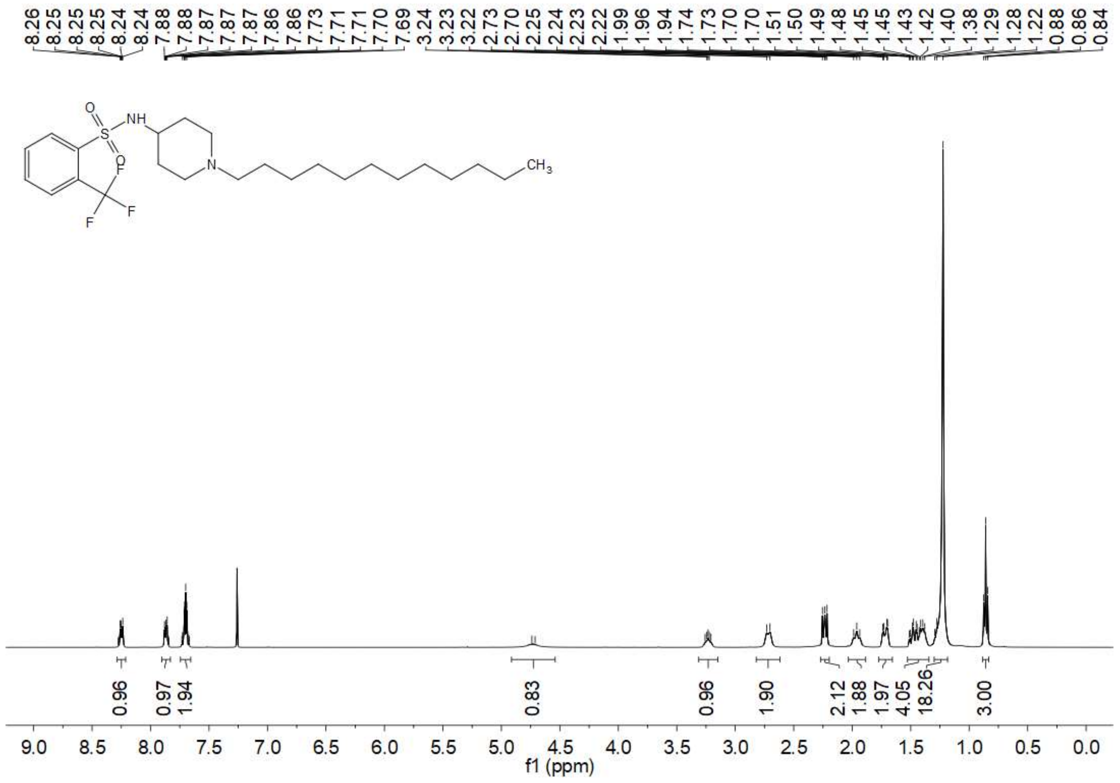

Figure S121. ^{1}H NMR spectrum ($CDCl_3$, 400 MHz) of **C_6**.

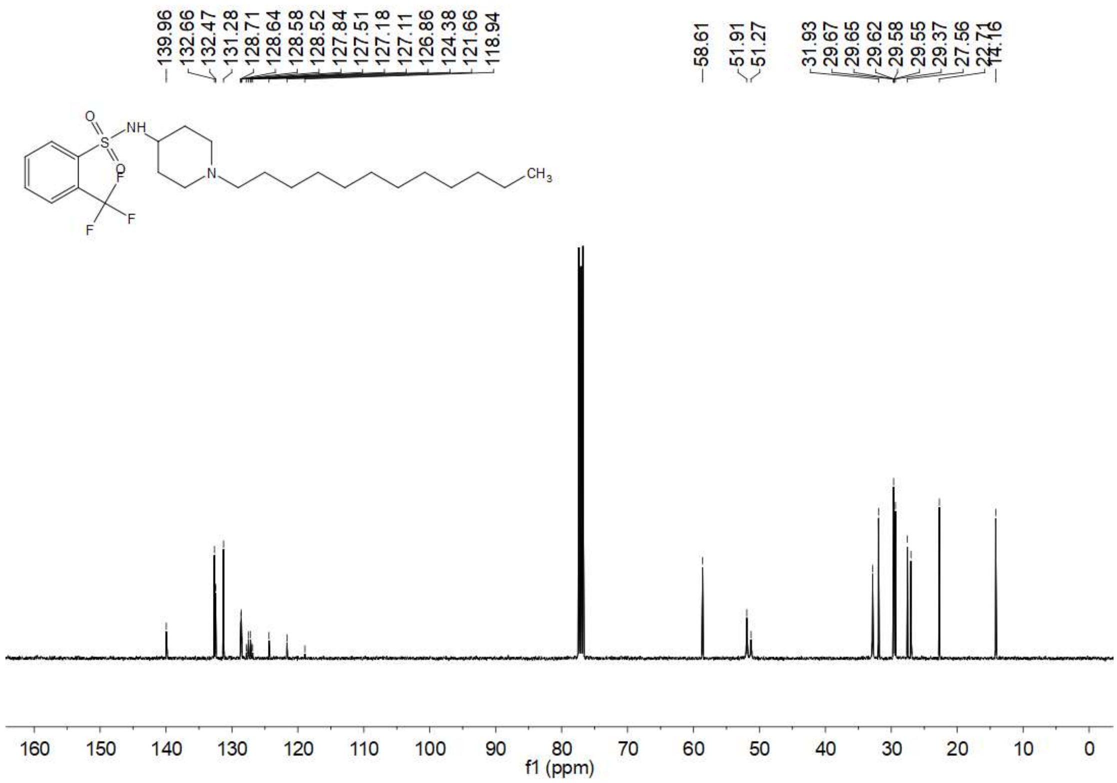

Figure S122. ^{13}C NMR spectrum ($CDCl_3$, 101 MHz) of **C_6**.

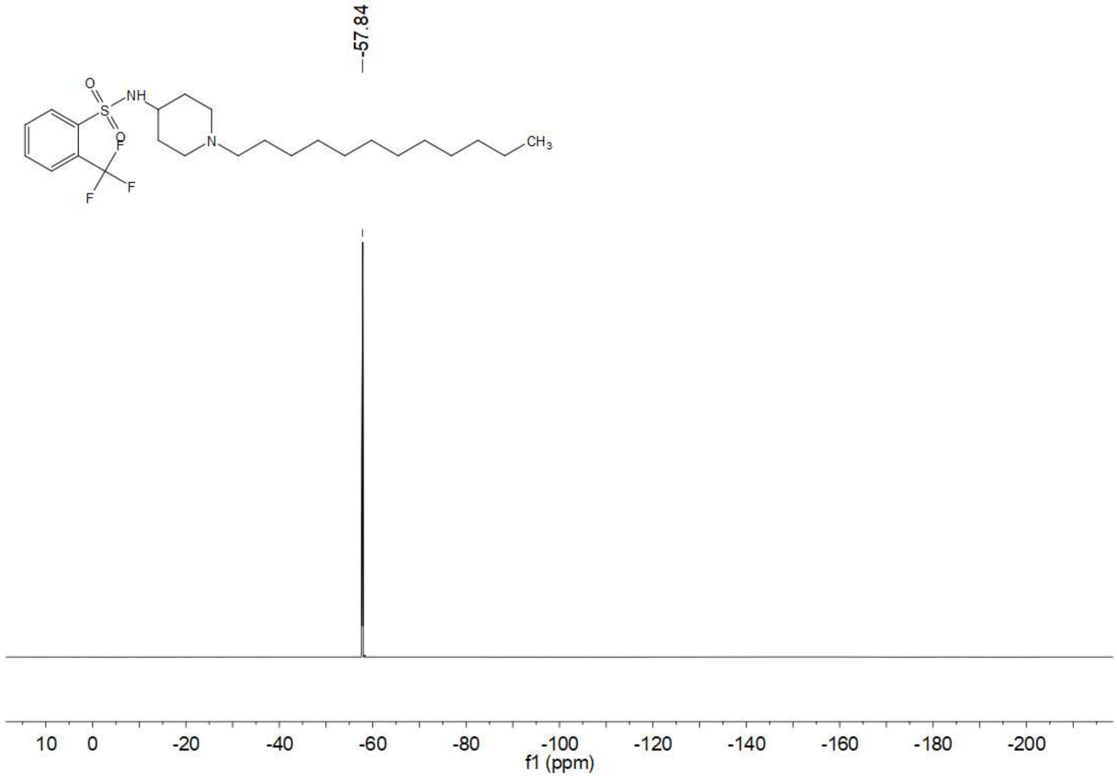

Figure S123. ^{19}F NMR spectrum ($CDCl_3$, 376 MHz) of **C_6**.

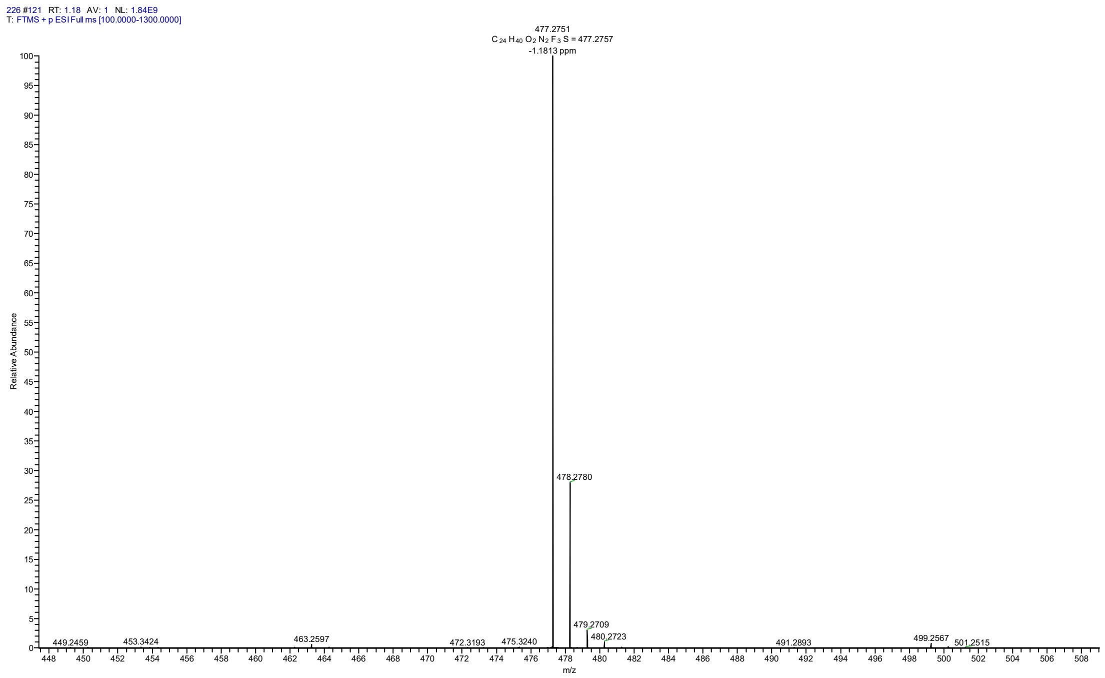

Figure S124. HRMS spectrum of $\mathbf{C_6}$.
